# Assessment of Population Genetic Diversity of Medicinal *Meconopsis integrifolia* (Maxim.) Franch. Using Newly Developed SSR Markers

**DOI:** 10.3390/plants13182561

**Published:** 2024-09-12

**Authors:** Jiahao Wu, Quanyin Yang, Wanyue Zhao, Xue Miao, Yuan Qin, Yan Qu, Ping Zheng

**Affiliations:** 1 Fujian Provincial Key Laboratory of Haixia Applied Plant Systems Biology, Haixia Institute of Science and Technology, College of Forestry, Fujian Agriculture and Forestry University, Fuzhou 350002, China; jiahao.mu@outlook.com (J.W.); smxsxl0521@163.com (Q.Y.); 2 Southwest Research Center for Engineering Technology of Landscape Architecture (State Forestry and Grassland Administration), Yunnan Engineering Research Center for Functional Flower Resources and Industrialization, College of Landscape Architecture and Horticulture Science, Southwest Forestry University, Kunming 650224, China; zhaowanyue0112@163.com (W.Z.); echo_miaoxue@163.com (X.M.)

**Keywords:** *Meconpsis integrifolia*, genetic diversity, populations, SSR markers

## Abstract

*Meconopsis integrifolia* is an endangered Tibetan medicinal plant with significant medicinal and ornamental value. Understanding its genetic diversity and structure is crucial for its sustainable utilization and effective conservation. Here, we develop a set of SSR markers based on transcriptome data to analyze the genetic diversity and structure of 185 individuals from 16 populations of *M. integrifolia*. The results indicate that *M. integrifolia* exhibits relatively high genetic diversity at the species level (the percentage of polymorphic bands PPB = 91.67%, Nei’s genetic diversity index He = 0.2989, Shannon’s information index I = 0.4514) but limited genetic variation within populations (PPB = 12.08%, He = 0.0399, I = 0.0610). The genetic differentiation among populations is relatively high (the coefficient of gene differentiation GST = 0.6902), and AMOVA analysis indicates that 63.39% of the total variation occurs among populations. This suggests that maintaining a limited number of populations is insufficient to preserve the overall diversity of *M. integrifolia*. Different populations are categorized into four representative subclusters, but they do not cluster strictly according to geographical distribution. Limited gene flow (Nm = 0.2244) is likely the main reason for the high differentiation among these populations. Limited seed and pollen dispersal abilities, along with habitat fragmentation, may explain the restricted gene flow among populations, highlighting the necessity of conserving as many populations in the wild as possible.

## 1. Introduction

**Figure 1:**
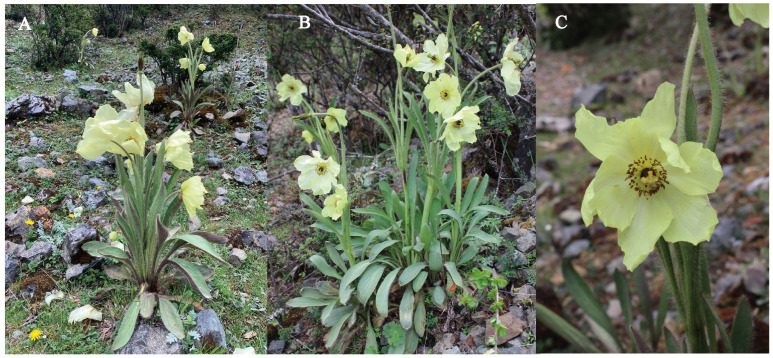
Habitat (**A**), whole plant morphology (**B**), and flower morphology (**C**) of *M. integrifolia* in the wild.

The genus *Meconopsis*, belonging to the Papaveraceae family, encompasses approximately 49 species, 38 of which are found in China [1,2]. Many species within the genus are traditionally used in Tibetan medicine for their properties such as heat-clearing, detoxification, diuresis, anti-inflammatory, and analgesic effects, as documented in the Pharmacopoeia of Traditional Tibetan Medicine of the People’s Republic of China, Volume I [3]. Additionally, *Meconopsis* is renowned for its large flowers, vibrant colors, and graceful appearance, making it one of the most striking mountain flowers [4]. It holds significance as the national flower of Bhutan and is recognized as one of the eight famous flowers of Yunnan province, representing a noteworthy genus of high-altitude wildflowers with substantial breeding potential. Among these, *M. integrifolia* (Maxim.) Franch. thrives in shrubs or forests at elevations ranging from 2700 to 5100 m. Its erect stature and its large and beautiful yellow flowers render it highly ornamental, showcasing considerable potential for landscaping applications (Figure 1). Moreover, it is classified as a first-level endangered Tibetan medicinal plant, underscoring its high economic value [5]. However, in recent years, changes in natural environments compounded by anthropogenic factors have led to alterations in the habitat of *Meconopsis*. Consequently, there has been a gradual decline in both the variety and quantity of these species, with many facing imminent endangerment.

Understanding genetic diversity is crucial for plant breeding programs and the preservation of genetic resources [6]. Numerous studies have investigated the genetic diversity of *Meconopsis* using various molecular markers. For instance, random amplified polymorphic DNA (RAPD) markers were used to assess the genetic diversity of *M. paniculata* and *M. simplicifolia*, revealing very low or no genetic polymorphism in both species among and within populations [7]. RAPD markers were also used to analyze the genetic diversity and population structure of 16 *M. quintuplinervia* populations, showing a relatively high rate of genetic variation at the species level (total genetic diversity Ht = 0.2954, Shannon’s information index I = 0.4371) and a relatively high level of genetic differentiation within populations (the coefficient of gene differentiation GST = 0.2320; analysis of molecular variance (AMOVA) revealed 78.3% of the variation within populations) [8,9]. Fluorescent amplified fragment length polymorphism (AFLP) markers were previously applied to analyze the genetic diversity and structure of *M. integrifolia*, revealing relatively high genetic variation at the species level (the percentage of polymorphic bands PPB = 82.0%, Nei’s genetic diversity index He = 0.2356, and I = 0.3695) but extremely low genetic diversity at the population level (He = 0.0317, I = 0.0480) [10]. Genetic structure analysis indicated that their genetic differentiation mainly existed among populations (GST = 0.8636), with three main genetic groups aligning with their geographical distribution [10]. Similarly, significant population differentiation was also observed in *M. integrifolia* populations based on the chloroplast DNA (cpDNA) analysis (GST = 0.843) [9]. Despite several investigations on genetic diversity in *Meconopsis* plants, considerable variation exists in the distribution and intraspecific differentiation of different species within the genus *Meconopsis*. Consequently, current genetic knowledge remains limited, impeding the genetic conservation and enhancement of these endangered yet economically and ornamentally significant Chinese medicinal herbs.

Simple Sequence Repeats (SSRs), also referred to as microsatellites, consist of short tandem repeats of 1–6 base pairs that are widely distributed across eukaryotic genomes [11]. SSR markers are characterized by their abundance, co-dominant inheritance, high polymorphism, high resolution, and transferability among closely related species, rendering them widely applicable in genetic diversity studies across diverse plant species [12,13]. The broad application of transcriptome sequencing has yielded abundant data for developing SSR markers in non-model species. SSR markers generated from transcriptome data have been widely deployed in numerous studies of non-model plants such as Siberian wildrye (*Elymus sibiricus* L.) [14], Perennial ryegrass (*Lolium perenne* L.) [15]. Our team previously investigated the mechanism of floral color formation in *Meconopsis* “Lingholm” using transcriptome data [16]. SSRs derived from one species can be used to discern diversity in related species and even in other genera within the same family [17]. Here, SSR markers were newly developed and used for the assessment of population genetic diversity and phylogenetic relationships among 16 populations comprising 185 individuals of wild-collected *M. integrifolia* (Table S1). Our work could provide valuable information for the systematic utilization and conservation of *M. integrifolia* germplasm resources.

## 2. Results

### 2.1. SSR Markers’ Development and Their Characterizations

Utilizing our transcriptome data obtained previously [16], a total of 91,615 unigenes were assembled for *Meconopsis* “Lingholm”. A total of 91,615 unigenes were scanned using the MISA software, and 15,165 SSR loci were detected in 12,455 unigenes (Table 1 and Table S2). The SSR locus in the transcriptome had six types, and the numbers of each repeat type varied greatly. Of all detected SSR loci, tri-nucleotide repeats with 10,022 loci accounting for 66.09% ranked as the most abundant type, followed by di-nucleotide repeats with 3360 loci (22.16%), hexa-nucleotide repeats with 806 loci (5.31%), penta-nucleotide repeats with 436 loci (2.88%), tetra-nucleotide repeats with 315 loci (2.08%), and mono-nucleotide repeats was the least abundant type with 226 loci (1.49%) (Table 1 and Table S2).

**Figure 2:**
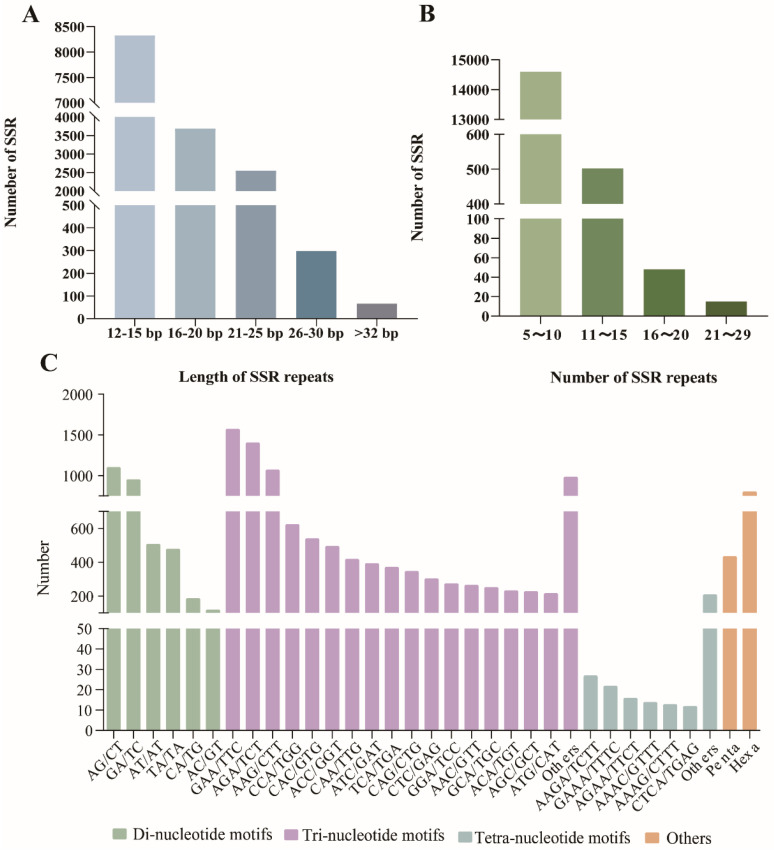
Characterizations of SSRs identified from transcriptome datasets obtained in our previous work of *Meconopsis* “Lingholm”. (**A**) Distribution of the length of repeats in SSR loci. (**B**) Distribution of the number of repeats in SSR loci. (**C**) Type distribution of SSRs identified in the assembled *Meconopsis* “Lingholm” unigenes.

The length of repeats in SSR loci ranged from 12 to 108 bp, with most of them (8327 loci, 54.9%) distributed between 12 and 15 bp (Figure 2A). The repeat unit frequencies of SSRs ranged from 5 to 29 times, with SSR loci having 5 to 10 repeats totaling 14,600 (96.27% of the total). Among these, SSR loci with 5 to 6 repeats were the most abundant, accounting for 67.56%. Only 15 SSR loci had repeats exceeding 21 times, representing a mere 0.09% (Figure 2B). The most abundant di-nucleotide repeats were AG/CT (1106; 32.92%) followed by GA/TC (956; 28.45%), AT/AT (509; 15.15%), and TA/TA (480; 14.29%). The most abundant tri-nucleotide repeats were GAA/TTC (1575; 15.72%) followed by AGA/TCT (1407; 14.04%) and AAG/CTT (1074; 10.72%). Meanwhile, the most affluent tetra-repeat motif types were AAGA/TCTT (27; 8.57%). The numbers of hexa- and penta-nucleotide motifs were 436 (2.88%) and 806 (5.31%), respectively (Figure 2C).

### 2.2. Assessment of Novel SSRs, Primer Design, and Genetic Diversity Statistics

**Figure 3:**
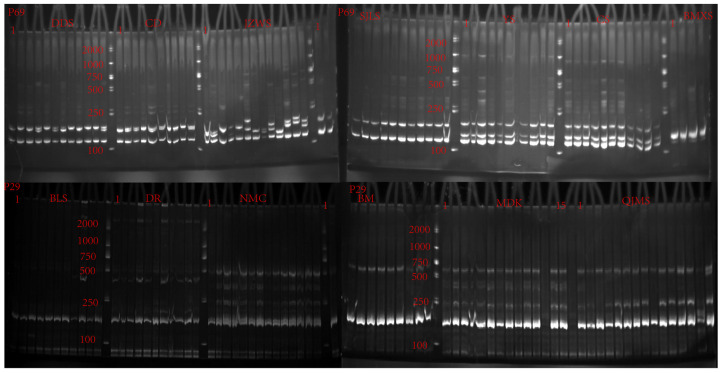
PCR amplification results using primers P69 and P29 in *M. integrifolia*. The gel image shows the amplification products of individuals from different populations, with bands separated by markers. The full names corresponding to the population abbreviations in the figure are provided in Table 2.

Initially, 96 pairs of non-repetitive SSR primers designed based on transcriptome data were selected for preliminary screening (Table S3), which were utilized to amplify DNA extracted from four individuals of *M. integrifolia*. Following screening, 23 pairs of primers producing clear and distinct bands were identified and employed for polymerase chain reaction (PCR) amplification on 185 individuals collected from 16 populations of *M. integrifolia* (Figure S1). Part of the amplification results are depicted in Figure 3. The amplification results show that the sizes of polymorphic bands in the amplified products varied from 100 to 220 bp, with a total of 84 polymorphic bands observed (Figure S1). On average, each primer pair generated 3.65 polymorphic bands.

At the species level, the genetic diversity indices were as follows: PPB = 91.67%, observed number of alleles (Na) = 1.9167, effective number of alleles (Ne) = 1.5037, He = 0.2989, and I = 0.4514. The differences in genetic diversity at the population level are shown in Table 2. The average value of Na was 1.1208, ranging from 1.0595 (Bomi Tianchi in Tibet, BM) to 1.4881 (Litang Jianziwan Mountain in Sichuan Province, JZWS); the average value of Ne was 1.0657, ranging from 1.0397 (BM) to 1.2683 (JZWS); the average value of He was 0.0399, ranging from 0.0226 (BM) to 0.1603 (JZWS); and the average value of I was 0.0610, ranging from 0.0334 (BM) to 0.2436 (JZWS). The consistent ranking results of different genetic diversity indicators including Na, Ne, He, I, and PPB indicated that three populations from BM, Yaoshan Mountain in Yunnan Province (QJYS), and Maidika in Tibet (MDK) showed the lowest genetic diversity, while populations from JZWS, Zogang Dongda Mountain in Tibet (DDS), Qamdo in Tibet (CD), and Baima Snow mountain in Yunnan Province (BMXS) had the highest rankings for each genetic diversity indicator (Table 2). A previous study has shown that altitude significantly impacts the plant traits and floral characteristics of *M. integrifolia* [18]. The correlation analysis results show that He is significantly correlated with altitude (r = 0.302, *p* = 0.033 < 0.05) (Figure S2A), while I (r = 0.295, *p* = 0.160 > 0.05) and PPB (r = 0.275, *p* = 0.529 > 0.05) did not show significant correlations with altitude changes (Figure S2B,C), suggesting that altitude had some influence on the genetic diversity of *M. integrifolia*.

### 2.3. Genetic Differentiation and Phylogenetic Relationship

**Figure 4:**
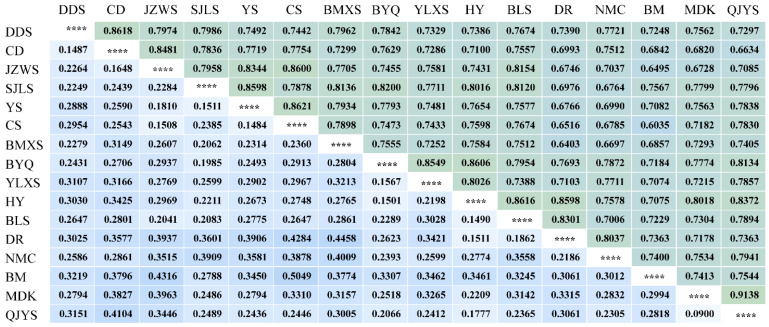
Genetic distance (below diagonal) and genetic similarity (above diagonal) among 16 populations of *M. integrifolia* based on SSR analysis. “****” indicates the comparison of populations with themselves, which naturally results in perfect similarity and theoretically a genetic distance of zero.

Nei’s method revealed high genetic differentiation among *M. integrifolia* populations (GST = 0.6902). The distribution of genetic variability among and within populations, as estimated by AMOVA, yielded consistent results. Specifically, AMOVA revealed that 63.39% of the total genetic variation was attributed to diversity among populations, with only 36.61% occurring within populations (Table 3). The gene flow (Nm) among populations was estimated to be 0.2244 (<1). The genetic similarity coefficient (GS) values among the populations ranged from 0.6035 to 0.9138 (Figure 4). Specifically, *M. integrifolia* from population BM and the *M. integrifolia* population from Cangshan Mountain in Yunnan Province (CS) had the greatest genetic distance and the lowest genetic similarity. Conversely, *M. integrifolia* from population QJYS and *M. integrifolia* from population MDK exhibited the smallest genetic distance and the highest genetic similarity. Mantel tests based on SSR markers revealed that genetic differentiation among populations (Nei’s genetic distance) did not show a significant correlation with geographic distance (Figure S2D). These results indicated that the genetic variation in *M. integrifolia* populations may be influenced by multiple factors.

**Figure 5:**
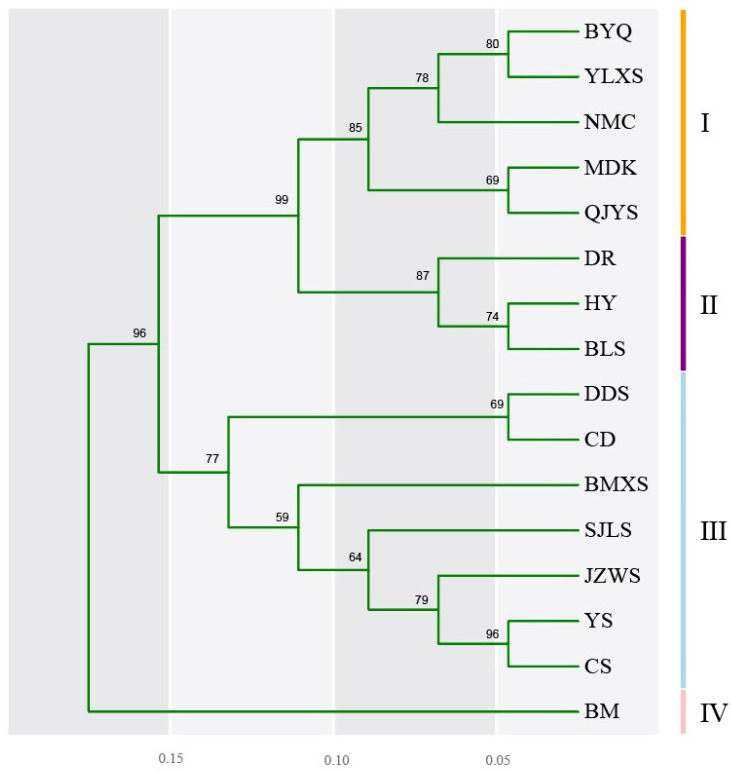
UPGMA dendrogram based on Nei’s genetic diversity coefficient among 16 populations of *M. integrifolia* using SSR marker analysis. Bootstrap analysis was performed with 1000 replicates, and the corresponding bootstrap values (%) were indicated.

To further elucidate the relationships among 16 populations of *M. integrifolia*, we employed unweighted pair-group method average (UPGMA) cluster analysis based on Nei’s genetic distance to generate a population dendrogram (Figure 5). The results indicated that the *M. integrifolia* population BM first separated into a distinct branch, forming subcluster IV, which showed a relatively distant genetic relationship with other populations. The population from Bayan Har Mountain in Qinghai Province (BYQ), Yulong Snow Mountain in Yunnan Province (YLXS), Namtso in Tibet (NMC), MDK, and QJYS clustered together and formed subcluster I, indicating closer genetic relationships among these populations. The population from Hongyuan in Sichuan Province (HY) and Xiaojin Balang Mountain in Sichuan Province (BLS) clustered together closely to form subcluster II with the population from Dari in Qinghai Province (DR). The remaining subcluster III contained most populations including DDS, CD, BMXS, Shergyla Mountain in Tibet (SJLS), JZWS, Yushu in Qinghai Province (YS), and CS. Among these, subclusters II and III each contain several geographically proximate populations. However, the overall clustering results do not entirely align with the geographical distribution. For instance, the population CS clusters closely with the distant population YS, while the geographically closer population YLXS is grouped into a different subcluster.

**Figure 6:**
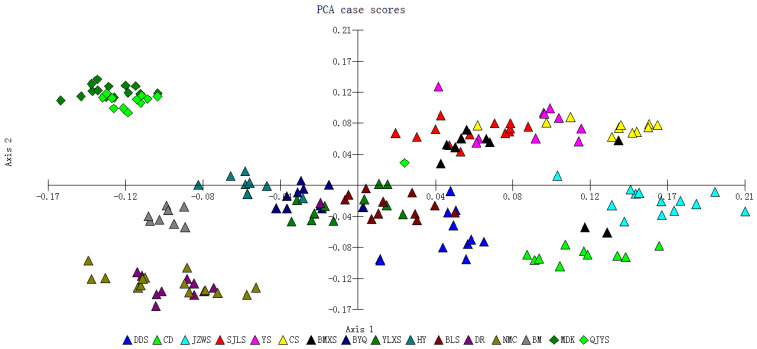
PCA cluster analysis of 185 individuals from 16 populations of *M. integrifolia*.

The principal component analysis (PCA) clustering results for all individuals from the 16 populations were generally consistent with the UPGMA cluster analysis (Figure 6). Individuals from the population BM clustered separately from those of other populations. Individuals from populations that clustered within the same branch did not group strictly according to their respective populations but were intermixed with individuals from other populations in the same subcluster (Figure 5 and Figure 6).

## 3. Discussion

### 3.1. Genetic Diversity of M. integrifolia

Intraspecific genetic variation is crucial for adaptation to environmental changes and the long-term survival of species [19]. In this study, we used previously obtained transcriptome data to screen a set of SSR markers suitable for the genus *Meconopsis*. The identified SSR markers were applied for the genetic diversity and structure of 16 populations of *M. integrifolia*, which could allow us to evaluate genetic variation between different populations and investigate potential factors influencing this variation. The SSR marker analysis revealed relatively high genetic diversity at the species level for *M. integrifolia* (PPB = 91.67%, He = 0.2989, and I = 0.4514) (Table 2). These results are obviously higher than those obtained by Guo et al. (2016) using AFLP markers for *M. integrifolia* (He = 0.2356, I = 0.3659) [10] and slightly higher than those reported by Yang et al. (2010) using RAPD markers for *M. quintuplinervia* (He = 0.2408, I = 0.3347) [8]. In contrast to the high genetic diversity at the species level, *M. integrifolia* showed relatively low genetic diversity at the population level (PPB = 12.08%, He = 0.0399, and I = 0.0610) (Table 2). This finding is consistent with the results of Guo et al. (2016) using AFLP markers, which also showed very low genetic diversity at the population level (He = 0.0317, I = 0.0480) [10]. Specifically, two sampling sites (Balang Mountain and Jianziwan Mountain) in this work overlap with the study conducted by Guo et al. (2016). Their AFLP analysis showed higher diversity at Jianziwan Mountain (YJJZW, PPB = 9.2%, He = 0.0320, I = 0.0475) compared to Balang Mountain (XJ, PPB = 5.9%, He = 0.0169, I = 0.0259) [10]. Similarly, our SSR marker analysis also revealed higher genetic diversity at Jianziwan Mountain (JZWS, PPB = 48.81%, He = 0.1603, I = 0.2436) than at Balang Mountain (BLS, PPB = 28.57%, He = 0.1051, I = 0.1565) (Table 2). Moreover, the higher level of polymorphism detected in *M. integrifolia* populations by SSR markers compared with AFLPs highlights the discriminating capacity of the former. The reason why SSR markers exhibit higher polymorphism compared to AFLP markers may be due to the high variability of SSR regions in the genome. These regions, due to their repetitive nature, are susceptible to mutations, which can lead to higher polymorphism. In addition, SSR markers are usually located in non-coding regions and may be subject to less selective pressure, which may also promote the development of their polymorphism. This finding aligns with previous studies that have demonstrated similar results when comparing SSRs with other marker systems in species such as Fig (*Ficus carica* L.) [20], tropical maize (*Zea mays* L.) [21], and olive tree (*Olea europaea* L.) [22].

### 3.2. Genetic Differentiation and Structure of M. integrifolia Populations

The genetic structure of plant populations reflects the interplay of various processes, including distributional changes [23], habitat fragmentation and/or population isolation [24], genetic drift [25], reproductive systems [26], gene flow [27], and life history traits [28] All these factors are related to the total amount of genetic variation and its distribution within and among populations. This study found that the genetic differentiation among *M. integrifolia* populations was relatively high (GST = 0.6902), and AMOVA analysis showed a similar pattern of genetic structure with 63.39% of the total variation among populations. Previous studies using AFLP markers also indicated high genetic differentiation among *M. integrifolia* populations (GST = 0.8636), with AMOVA analysis showing 88.9% of the total variation among populations [10]. Similarly, results based on cpDNA analysis showed high genetic differentiation among *M. integrifolia* populations (GST = 0.843), with AMOVA analysis indicating 80.18% of the total variation among populations [9]. Consistently, these results suggest significant genetic differentiation among *M. integrifolia* populations. Although the UPGMA clustering results (Figure 5) and PCA clustering analysis (Figure 6) show that some geographically proximate populations cluster together according to their terrain, the genetically similar groups are intermixed in their geographical distribution, suggesting that geographical distribution has some influence on the genetic structure of *M. integrifolia*. However, there was no significant correlation between genetic distance and geographical distance among populations (Figure 4 and Figure S2D). This may imply that geographical isolation is not the primary factor driving genetic differentiation, or its effects might be obscured by other factors. The gene flow Nm value among populations based on SSR markers in our work was 0.2244 (less than 1), indicating relatively low gene flow among *M. integrifolia* populations, similar with that estimated by AFLP markers (Nm = 0.0395) [10]. These results imply that limited gene flow may be the primary reason for the low genetic variation within populations and high differentiation among populations of *M. integrifolia*.

The level of gene flow in a species is influenced by its biological characteristics and the behavior of its pollinators, and the breeding system significantly impacts population differentiation [29,30]. Typically, self-pollinating species retain more genetic diversity among populations rather than within populations, compared to outcrossing species [31]. It has been reported that *M. integrifolia* has a mixed mating system, with approximately 65.0% of seeds set through autonomous self-pollination [32], potentially promoting high genetic differentiation among populations. Additionally, the primary pollinators of *M. integrifolia* are flies and thrips with limited flight capabilities [32], restricting their movement and thus reducing pollen exchange among populations, contributing to the high genetic differentiation. According to our field observations, the *M. integrifolia* plant often produces many small, lightweight, smooth seeds that lack obvious dispersal adaptations. This kind of seeds are likely dispersed over short distances near the parent plant through gravity. Therefore, gene flow via seeds or pollen within and among *M. integrifolia* populations is limited.

Besides biological characteristics, limited gene flow is also restricted by fragmented and heterogeneous habitats [33]. *M. integrifolia* grows at elevations of 2700–5100 m in shrubs or under forests, distributed in the Eastern Himalayas and Hengduan Mountain regions. The parallel mountain ranges and deep river valleys extending from north to south in this area may act as physical barriers, hindering effective gene flow between *M. integrifolia* populations and thereby enhancing population differentiation. The wild populations of *M. integrifolia* are small and unable to form large populations, which occurs in various ecological environments, including different altitudes, climate conditions, and habitat types. Environmental heterogeneity may lead to differences in natural selection, which could, in turn, affect the genetic structure among populations [34]. Therefore, the limited seed and pollen dispersal capability of *M. integrifolia* and its habitat fragmentation likely explain the current limited gene flow (Nm = 0.2244), resulting in increased genetic erosion within populations and genetic differentiation among populations.

Understanding the genetic diversity and structure of natural populations of endangered species is essential for effective conservation measures [31,35]. Our study indicates that *M. integrifolia* exhibits a high degree of genetic differentiation (GST = 0.6902), with most genetic variation occurring among populations. Maintaining only a limited number of populations is insufficient to preserve the overall genetic diversity. Therefore, conservation efforts should focus on increasing population numbers to enrich the gene pool [36]. To protect the genetic diversity of *M. integrifolia*, it is essential to preserve as many wild populations as possible. Moreover, *M. integrifolia* populations can be classified into four representative groups (Figure 5), and representative populations from different groups should be prioritized for monitoring and management under limited conditions. Moreover, our findings suggest that genetic drift plays a significant role in the genetic differentiation of *M. integrifolia* populations. Conservation strategies should include delineating development areas to allow populations time for self-renewal and expansion. In situ conservation should be the primary approach, complemented by ex situ conservation when necessary. In situ efforts should establish protected areas to prevent trampling and damage by humans and livestock. For populations with very few individuals, severely degraded habitats, or those unable to reproduce independently in the wild, ex situ conservation and scientific management are recommended as alternative strategies. Additionally, artificial propagation should be considered when necessary to supplement *M. integrifolia* populations. Scientific methods to enhance seed germination rates, improve water and nutrient management, and promote plant growth should be employed. Research on rapid propagation techniques through tissue culture could also help increase the number of *M. integrifolia* clonal populations [37].

## 4. Materials and Methods

### 4.1. Samples Collection and DNA Extraction

**Figure 7:**
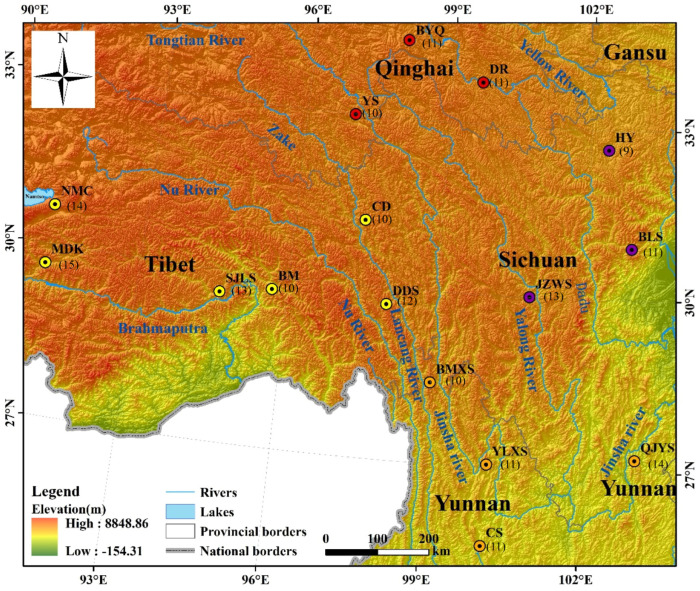
Location distributions of the 16 populations of *M. integrifolia* sampled for this study. Six of the sixteen populations were collected from Tibet, four from Yunnan Province, and three each from Qinghai Province and Sichuan Province. The number of samples collected from each population is also indicated below the corresponding population name. Details of the populations are provided in Table S1.

The plant material utilized here in studying the genetic diversity and population structure of *M. integrifolia* was collected from sixteen landraces (16 sites) with 185 individuals (Figure 7, Table S1). These sampling sites were strategically chosen to cover a wide geographic range, primarily sourced from four provinces: Yunnan, Sichuan, Tibet, and Qinghai. Among them, there were 4 populations in Yunnan, 3 in Sichuan, 6 in Tibet, and 3 in Qinghai, with 10–15 individuals collected per sampling point. The average elevation of the 16 sampling sites was 4329 m, with the highest altitude at Namtso in Tibet (NMC) reaching 5200 m and the lowest at Cangshan Mountain in Yunnan (CS) at 3700 m. The barriers between sampling sites were primarily mountain ranges or rivers. Tender fresh leaf tissue from each individual was collected for genomic DNA extraction, which was performed using the Qiagen DNA extraction kit (Qiagen, Hilden, Germany) method.

### 4.2. SSRs Identification and Primer Design

SSRs were identified based off our transcriptome data obtained in previous work (NCBI: PRJNA1151241) [16] using MISA 1.0 software [38]. Primer design for SSRs was conducted using Primer5 software, with parameters set as follows: primer length of 18–22 bp, GC content of 40–60%, annealing temperature of 50–70 °C, Tm of 55–65 °C, and product size of 100–300 bp. Measures were taken to avoid secondary structures such as hairpins, dimers, mismatches, and primer–dimers. A total of 96 pairs of non-repetitive SSR primers were selected as preliminary screening primers, synthesized by Shuoqing Biotechnology Co., Ltd. in Kunming, China, and tested on the DNA samples from four individuals of *M. integrifolia*. Primers producing clear, non-streaky, highly polymorphic, and reproducible bands were selected, resulting in 23 primer pairs with good polymorphism that were kept for genetic diversity analysis of 185 individuals from 16 landraces of *M. integrifolia* (Table 4). PCR reactions were conducted in a 25 μL volume containing 60 ng template DNA, 2 mM Mg^2+^, 0.2 mM dNTPs, 0.5 μM primer, and 1.6 U Taq DNA polymerase. PCR cycling conditions included an initial denaturation step of 5 min at 94 °C, followed by 35 cycles of 30 s at 94 °C, 30 s at the specific annealing temperature (ranging from 51 to 63 °C depending on the primers used), and 20 s at 72 °C, with a final extension step of 1 min at 72 °C. Polymorphic bands of PCR products were detected using 12% SDS-PAGE separation gel, and the amplified products were observed and photographed, and gel images were saved under a UVP gel imaging system.

### 4.3. Data Processing and Analysis

The GENE MAPPER 4.1 software was utilized for precise data point analysis [39]. The core base repeat number, corresponding to primer pairs, was instrumental in determining the exact size of the loci. The polymorphism detected for the primer pairs was ascertained based on the locus information analyzed, leading to the selection of highly specific and polymorphic loci for further research endeavors. Given that SSRs are co-dominant genetic markers, a binary matrix (0, 1) was constructed to represent the presence (1) and absence (0) of bands [40]. This matrix, derived from SSR data, was then used to evaluate genetic variability, employing a suite of metrics including the percentage of polymorphic bands (PPB), the observed number of alleles per locus (Na), the effective number of alleles per locus (Ne), Nei’s genetic diversity index (He), the Shannon’s information index (I), the coefficient of gene differentiation (Gst), gene flow (Nm), Nei’s standard genetic distance (D), standard genetic identity (J), and genetic similarity (GS), with the aid of POPGENE 1.32 software [41].

For the analysis of molecular variance (AMOVA), the Arlequin v3.5.2.2L [42] software was employed, conducting 1000 simulations to ascertain the significance of differences in data components between inter-population and intra-population variations and to calculate the respective percentages of variation. The UPGMA method was applied in both MEGA 11 and MVSP 3.22 software to analyze the amplification results of the 16 populations of *M. integrifolia*, and each software was used to construct a dendrogram [43]. Additionally, a principal component analysis (PCA) was performed to delineate the genetic relationships among these populations. Furthermore, the TFPGA 1.3 software was leveraged to execute Mantel tests, aiming to assess the correlation between genetic distances calculated from SSR markers and geographical distances [44].

## 5. Conclusions

*M. integrifolia*, a member of the Papaveraceae family, is an endangered valuable Tibetan medicinal plant with significant medicinal and ornamental value. Understanding genetic diversity and structure is crucial for its sustainable utilization and implementing effective conservation measures. In this study, we developed a set of SSR markers based on transcriptome data to analyze the genetic diversity and structure of 185 individuals from 16 populations of *M. integrifolia*. The results indicate that *M. integrifolia* exhibits relatively high genetic diversity at the species level (PPB = 91.67%, Nei’s genetic diversity index He = 0.2989, Shannon’s information index I = 0.4514) but limited genetic variation within populations (PPB = 12.08%, He = 0.0399, I = 0.0610). The genetic differentiation among populations is relatively high (GST = 0.6902), and AMOVA analysis also indicates that 63.39% of the total variation occurs among populations. This suggests that maintaining a limited number of populations is insufficient to preserve the overall diversity of *M. integrifolia*. All the collected populations can be categorized into four representative subclusters, but they do not cluster strictly according to geographical distribution, implying that geographical isolation might not be the primary factor driving genetic differentiation. Limited gene flow (Nm = 0.2244) is likely the main reason for high differentiation among these populations. Limited seed and pollen dispersal abilities, along with habitat fragmentation, may explain the restricted gene flow among populations, highlighting the necessity of conserving as many populations in the wild as possible.

## Figures and Tables

**Table 1:** Prediction of SSRs out of our previous transcript datasets of *Meconopsis* “Lingholm” [16].

Parameter	Number
Total number of unigenes examined	91,615
Total number of identified SSRs	15,165
Number of SSR containing unigenes	12,455
Mono nucleotide	226
Di nucleotide	3360
Tri nucleotide	10,022
Tetra nucleotide	315
Penta nucleotide	436
Hexa nucleotide	806

**Table 2:** Genetic diversity within populations of *M. integrifolia* based on SSR analysis.

Population	Sampling Location	PPB	Na	Ne	He	I
DDS	Zogang Dongda Mountain (Tibet)	40.48%	1.4048	1.2443	0.1425	0.2132
CD	Qamdo (Tibet)	32.14%	1.3214	1.2129	0.1219	0.1800
JZWS	Litang Jianziwan Mountain (Sichuan Province)	48.81%	1.4881	1.2683	0.1603	0.2436
SJLS	Shergyla Mountain (Tibet)	30.95%	1.3095	1.1714	0.1026	0.1556
YS	Yushu (Qinghai Province)	28.57%	1.2857	1.1822	0.1043	0.1549
CS	Cangshan Mountain (Yunnan Province)	28.57%	1.2857	1.1424	0.0874	0.1352
BMXS	Baima Snow Mountain (Yunnan Province)	30.95%	1.3095	1.1911	0.1119	0.1672
BYQ	Bayan Har Mountain (Qinghai Province)	26.19%	1.2619	1.1868	0.1043	0.1523
YLXS	Yulong Snow Mountain (Yunnan Province)	25.00%	1.2500	1.1254	0.0779	0.1206
HY	Hongyuan (Sichuan Province)	23.81%	1.2381	1.1385	0.0817	0.1233
BLS	Xiaojin Balang Mountain (Sichuan Province)	28.57%	1.2857	1.1791	0.1051	0.1565
DR	Dari (Qinghai Province)	22.62%	1.2262	1.1300	0.0779	0.1177
NMC	Namtso (Tibet)	27.38%	1.2738	1.1457	0.0879	0.1342
BM	Bomi Tianchi (Tibet)	5.95%	1.0595	1.0397	0.0226	0.0334
MDK	Maidika (Tibet)	17.86%	1.1786	1.0989	0.0599	0.0911
QJYS	Yaoshan Mountain (Yunnan Province)	11.90%	1.1190	1.0634	0.0381	0.0583
Mean		12.08% (±0.16)	1.1208 (±0.13)	1.0657 (±0.06)	0.0399 (±0.12)	0.0610 (±0.05)
Total		91.67%	1.9167	1.5037	0.2989	0.4514

**Table 3:** Genetic differences among the 16 populations of *M. integrifolia* using AMOVA analysis.

	Degrees of Freedom	Sum of Squares of Deviations	Variance Component	Variation Ratio	*p* Value
Among populations	15	1551.684	8.53261	63.39%	<0.001
Within populations	169	832.932	4.92859	36.61%	<0.001

**Table 4:** SSR primer characteristics used for genetic diversity analysis.

Number	Primer Sequences (5′-3′)	Repeat Motif	Tm	Size
P5	TTGCCTTGAAATTGAACATTCTT	(ATT)5	59.997	148
	AAGCAAACCCAAGATAAAACACA			
P11	TATGGATATGGAATTGGAATTGG	(TGC)6	59.769	157
	AGACTTCGACCTAACACGCTTTT			
P14	TCATTCACTGCTAGTACTCCAACTC	(TCA)6	59.037	135
	TGATGCTGAGAGTTTCTGTTTGA			
P17	TTTGGTTCCTTCTCCTCCTCTTA	(TC)7	60.567	151
	GATTGATGAACTGAAAAGCATCC			
P20	AGCAACAACAACATCAACATCAG	(GGT)5	60.088	132
	GCATGTTAGACTGAGATGGAAGG			
P23	ATGTCTCTGGAGTAATGGGTGTG	(TA)6	60.295	142
	CCGACAATGAAATGTAAAACCAT			
P24	GGTGAGAAGGGAGGATTTATGTC	(GGT)6	60.196	140
	ACTTCAGTAGTCAATCCGAACCC			
P27	AACACATGTAGCAATCTCGTCCT	(TCA)5	60.075	145
	GTTTCCTGGTTTTCTCATGGTT			
P29	GGTTTCTGCTCTTTGCTTCAGTA	(AT)7	60.069	155
	CACAACCCAACTAGAAAGACCAC			
P30	GAAAACAACACAAGAATCACCGT	(CAC)5	60.309	145
	GTTGACGTCTTCTTCTTCGTCTC			
P31	GTTGTCGATGATGAAAATGATTG	(ATG)6	59.333	143
	GCTAAAGACACCTTTTGAAGCAA			
P37	TGTAGGTAATAGCAGAGCCATTG	(TCT)5	58.492	151
	CTTGGGTAGTGCTTGATATGTCG			
P40	GTTACCCAACCATCGGTAGTTCA	(TGC)5	62.175	140
	GCTCGAGTTAATACATCCTCACG			
P42	ACTTTGCTAGGGAATGTCCAGAT	(GTG)5	60.373	148
	TTATGGTCTCCATTACGATCACC			
P57	CTTGAAAAGACGACTACACCCTTA	(TA)6	58.929	140
	CCTCAAGAGACTAGTCGGGAACT			
P58	TTCATGAATGAGGATGTGTATGC	(CAA)5	59.843	156
	AGAGAATGAGGATTGCATTACGA			
P65	CAAACATCAGTTTCCAACAACAA	(AAC)5	59.927	160
	GTTTACATTACCCGGAAGATGCT			
P69	CTTCTTCTTCGGCAATTGAAAAT	(AAT)5	60.898	138
	TACCAACACATCCTCTTACCGTT			
P76	CGAAGAATTAAGTGATGGAGAGG	(GAG)5	59.273	157
	TTAGGGCAACAGTGAATTTTGAT			
P84	AGAGATCATAGTTACAACCGCCA	(GTG)5	60.035	139
	CCTTGCAGTAAACCCTAACTGTG			
P91	GCTTATATGAAGATGCAACGAGG	(ACA)6	60.128	138
	CATTGTTTGACATTGAAAATGGA			
P94	TGGTGGTGGAGTAGGAGTTAGAG	(ATC)5	59.685	160
	TTGAAGAGCAAGAGAAAGTCCAG			

## Data Availability

The data presented in this study are available in the article, Supplementary Materials and online repositories.

## Supplementary Materials

The following supporting information can be downloaded at: https://www.mdpi.com/article/10.3390/plants13182561/s1. Figure S1: PCR amplification of all primers in *M. integrifolia*; Figure S2: Correlation analysis of altitude and He, PPB and I, and Nei’s genetic distance with geographic distance; Table S1: Location distributions of the 16 populations of *M. integrifolia*; Table S2: Summary of the different repeat units of identified SSRs in *M. integrifolia*; Table S3: 96 pairs of non-repetitive SSR primers selected for preliminary screening.
