# Supplementary material for: Assessment of Population Genetic Diversity of Medicinal Meconopsis integrifolia (Maxim.) Franch. Using Newly Developed SSR Markers
Source: Plants (Basel). 2024 Sep 12;13(18):2561. doi: 10.3390/plants13182561 (PMC11435270; doi:10.3390/plants13182561)
Supplement: Supplementary file 1 [file plants-13-02561-s001.zip › Figure S1.pdf]

PCR amplification of different primers among 16 populations of M. integrifolia. The amplification results of individuals from different populations were displayed in the gel image, separated by bands where the markers are located.

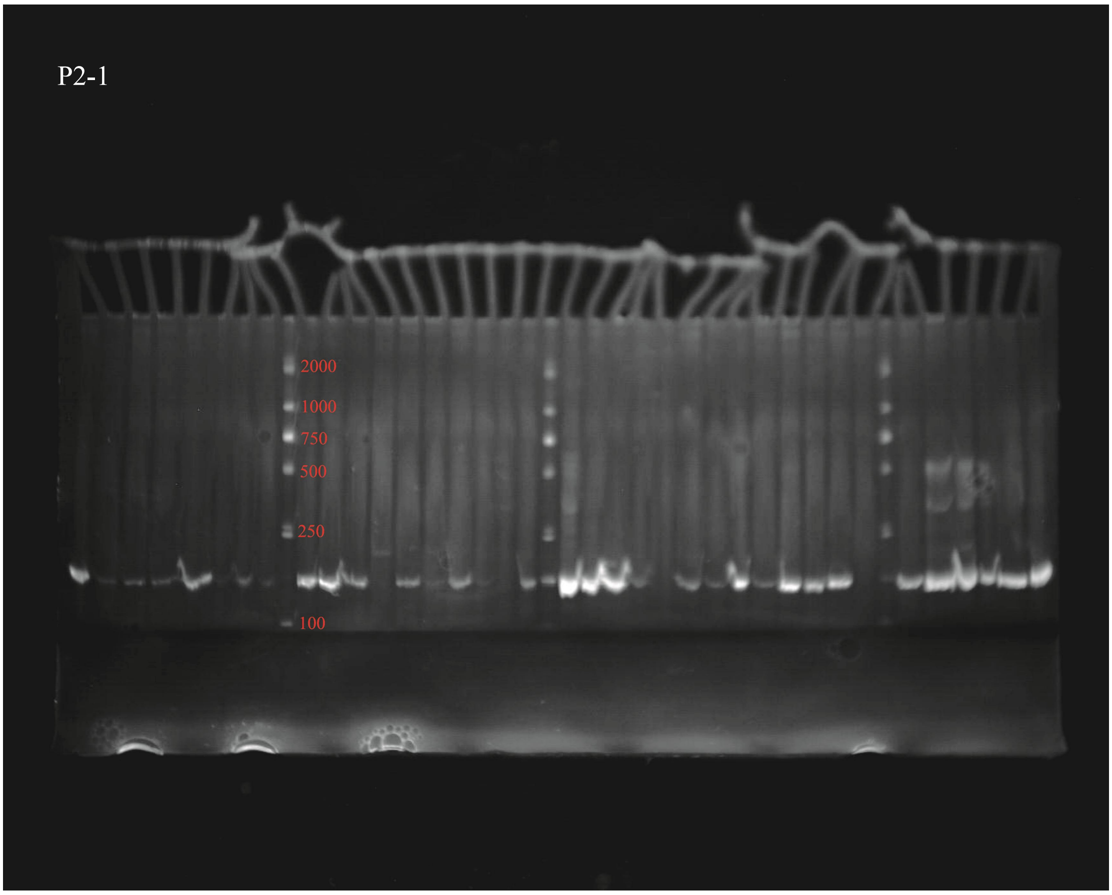

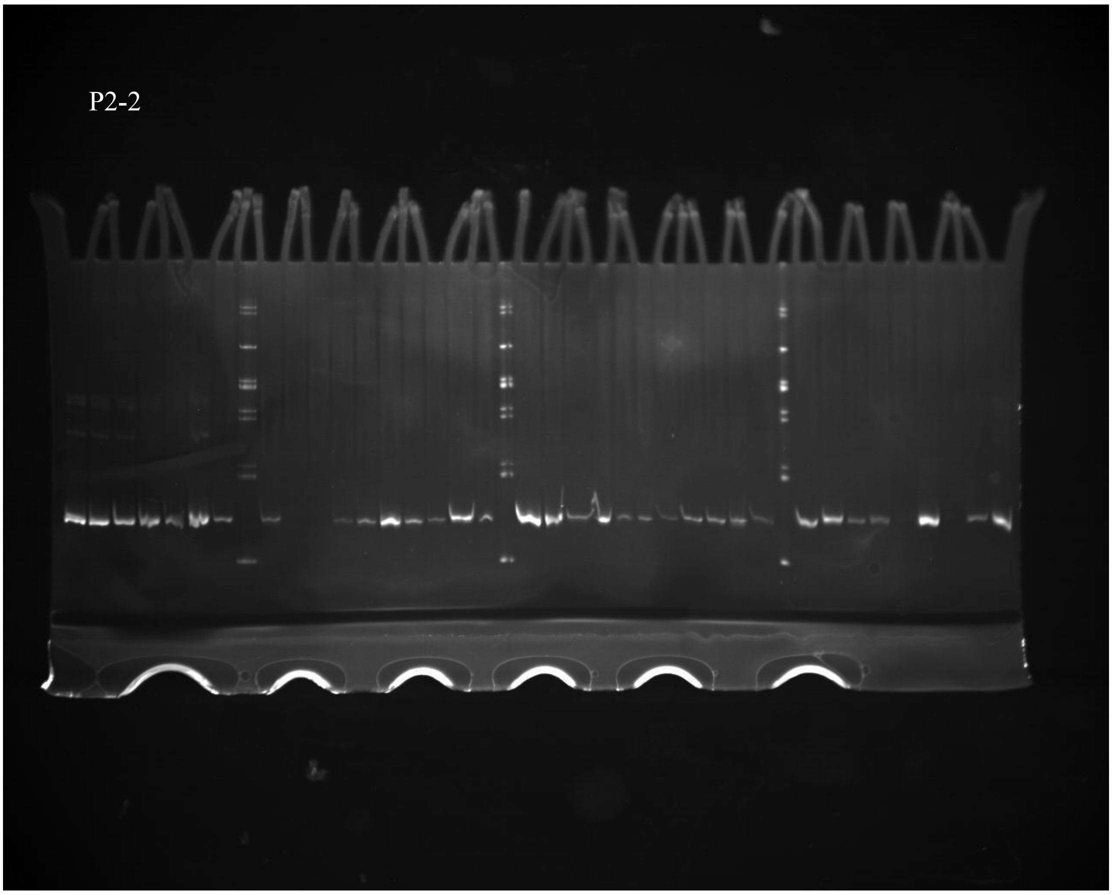
P2-2

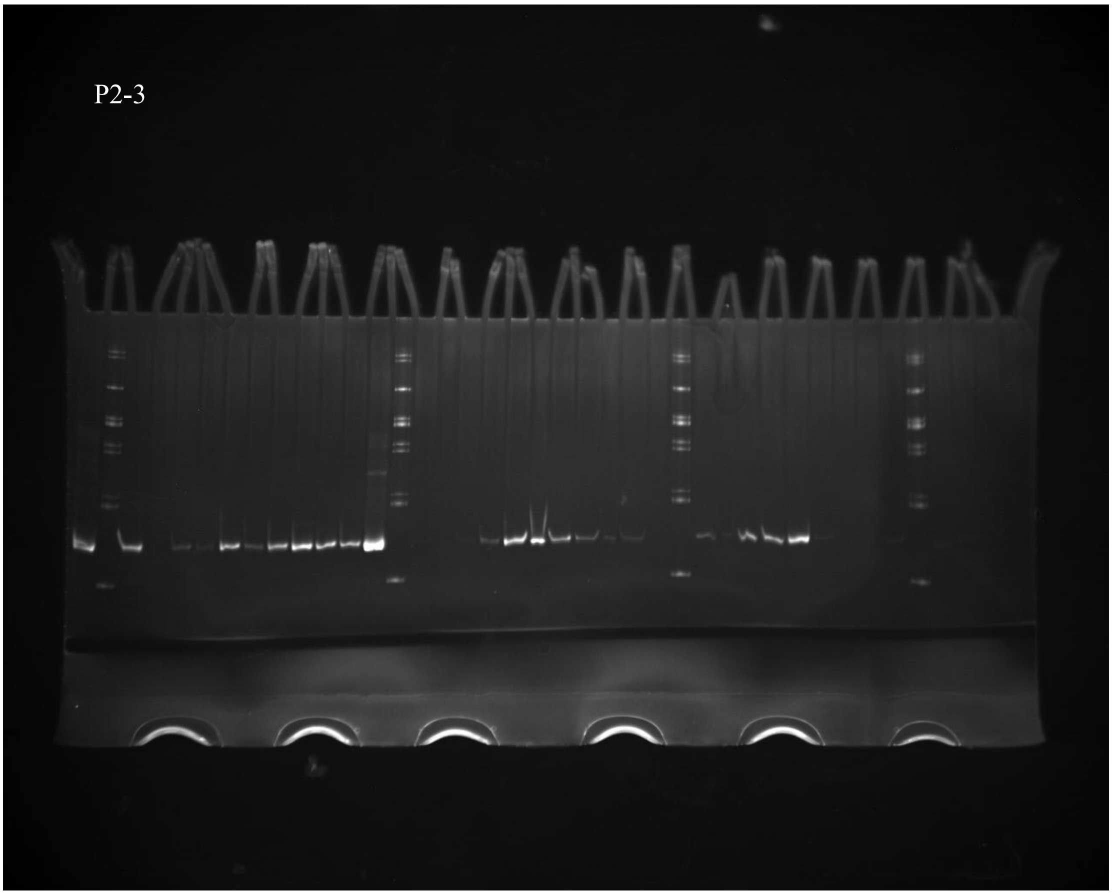
P2-3

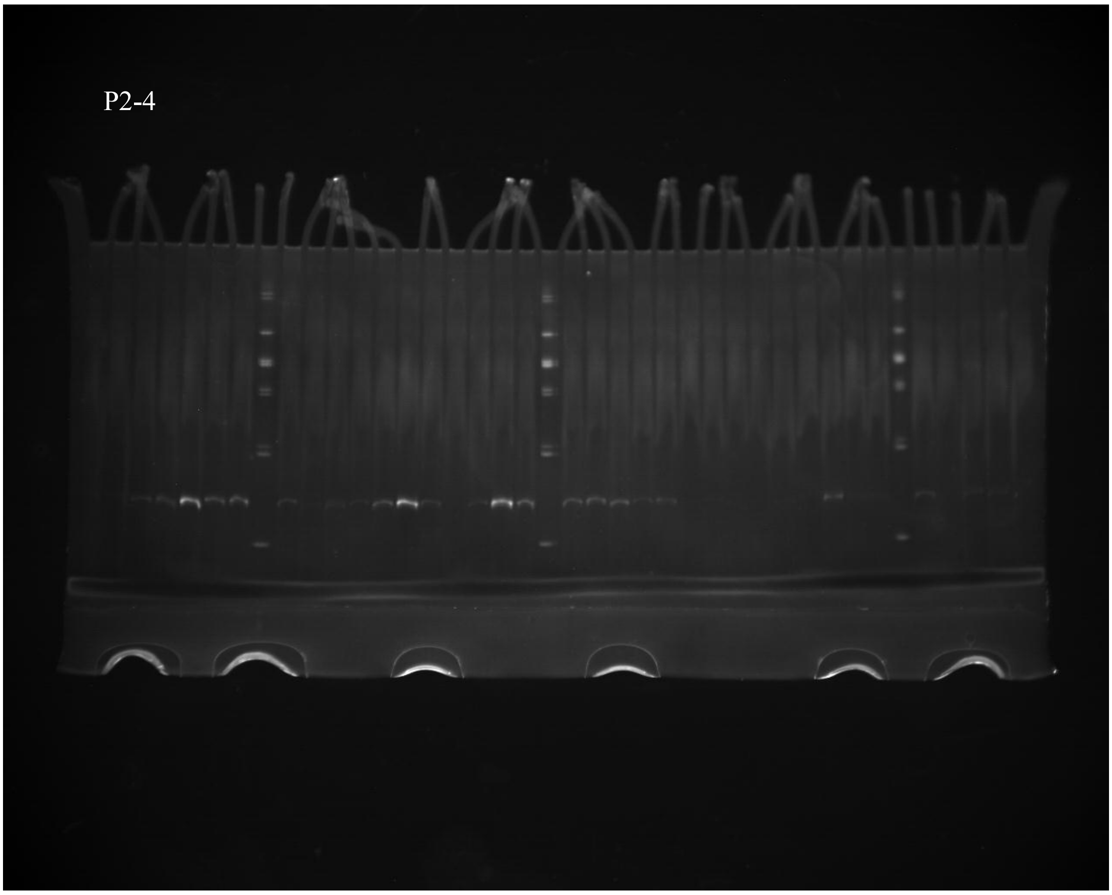
P2-4

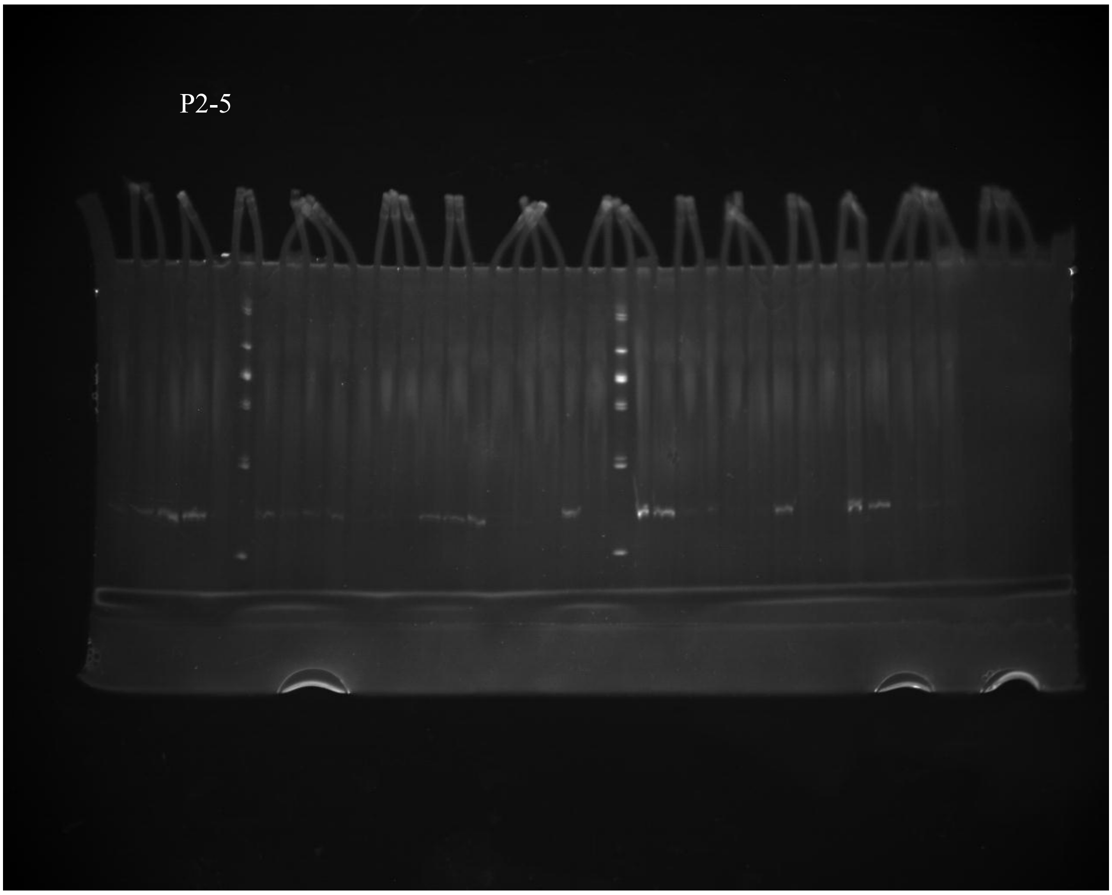
P2-5

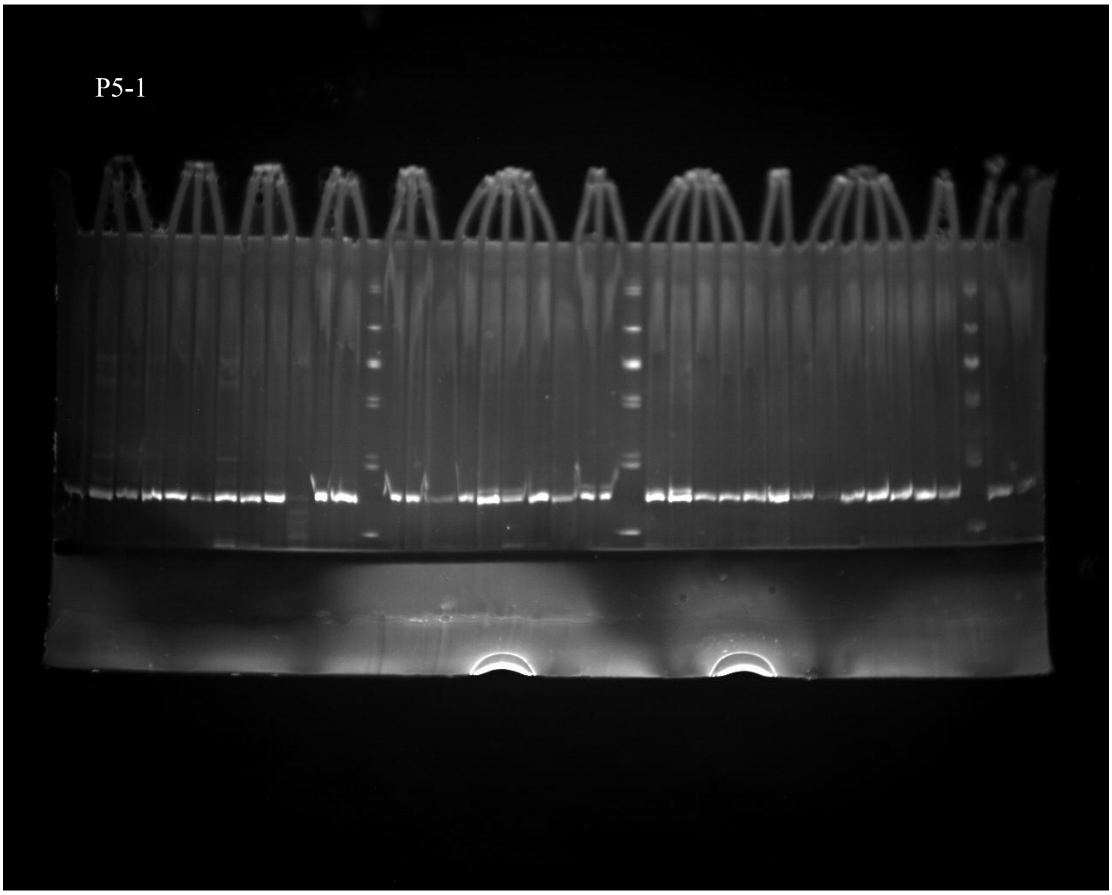
P5-1

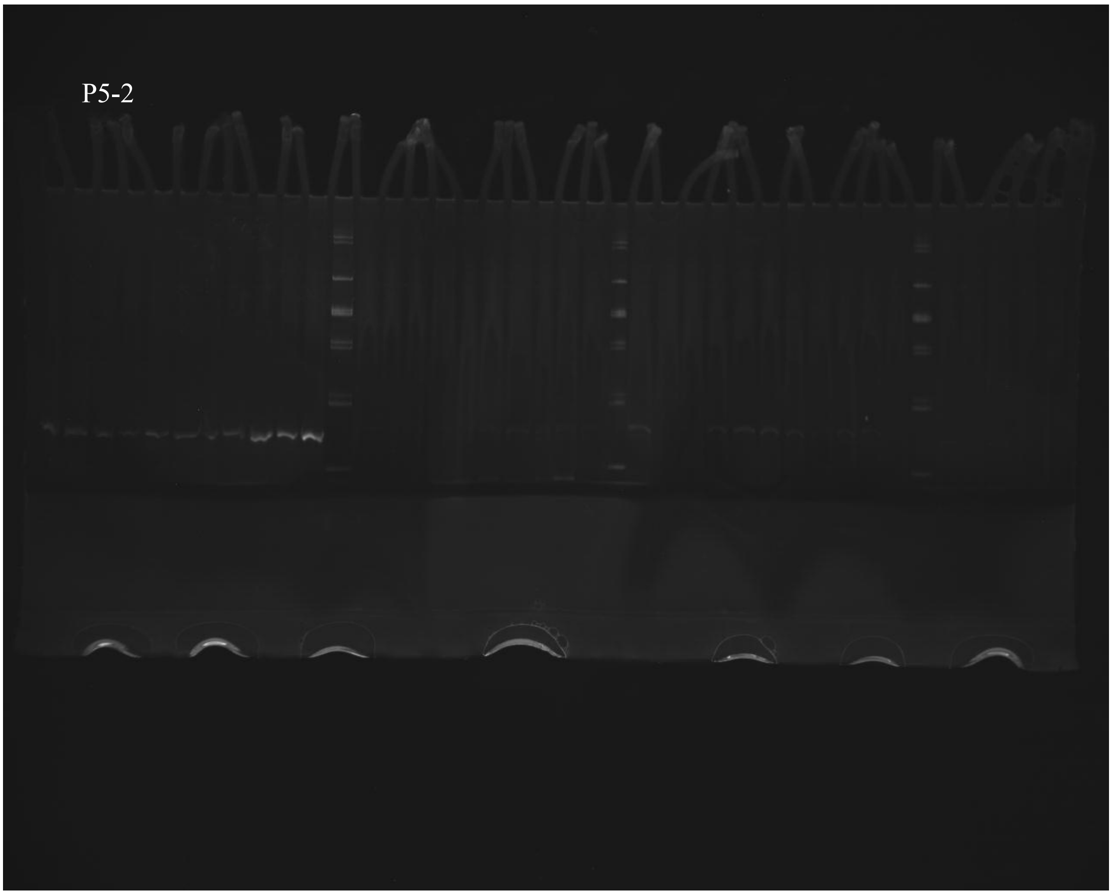
P5-2

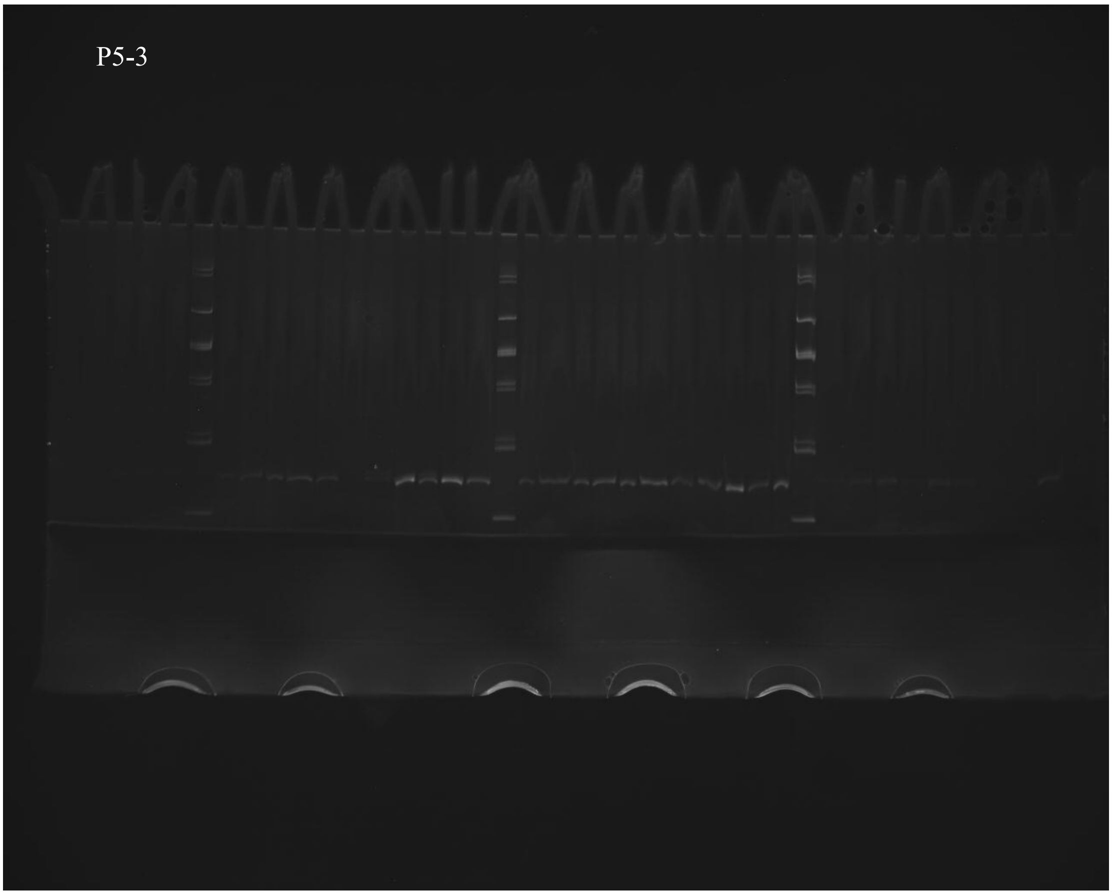
P5-3

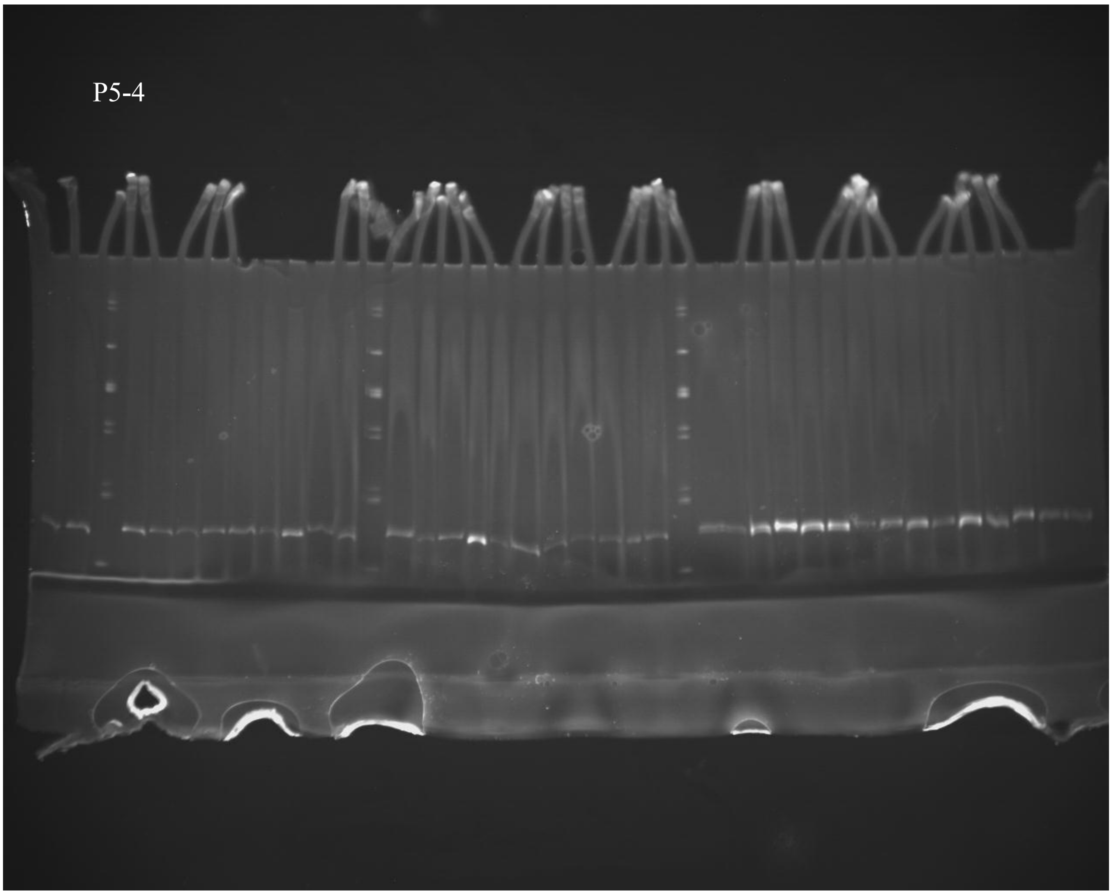
P5-4

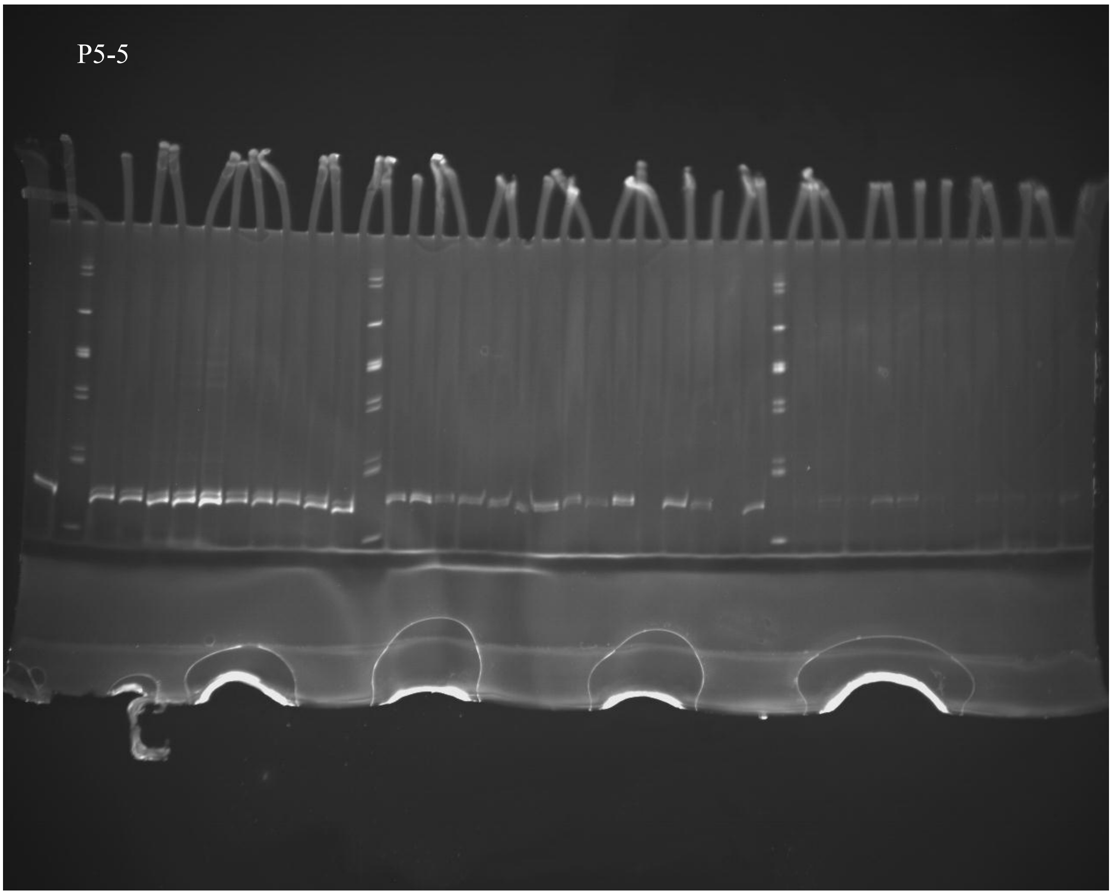
P5-5

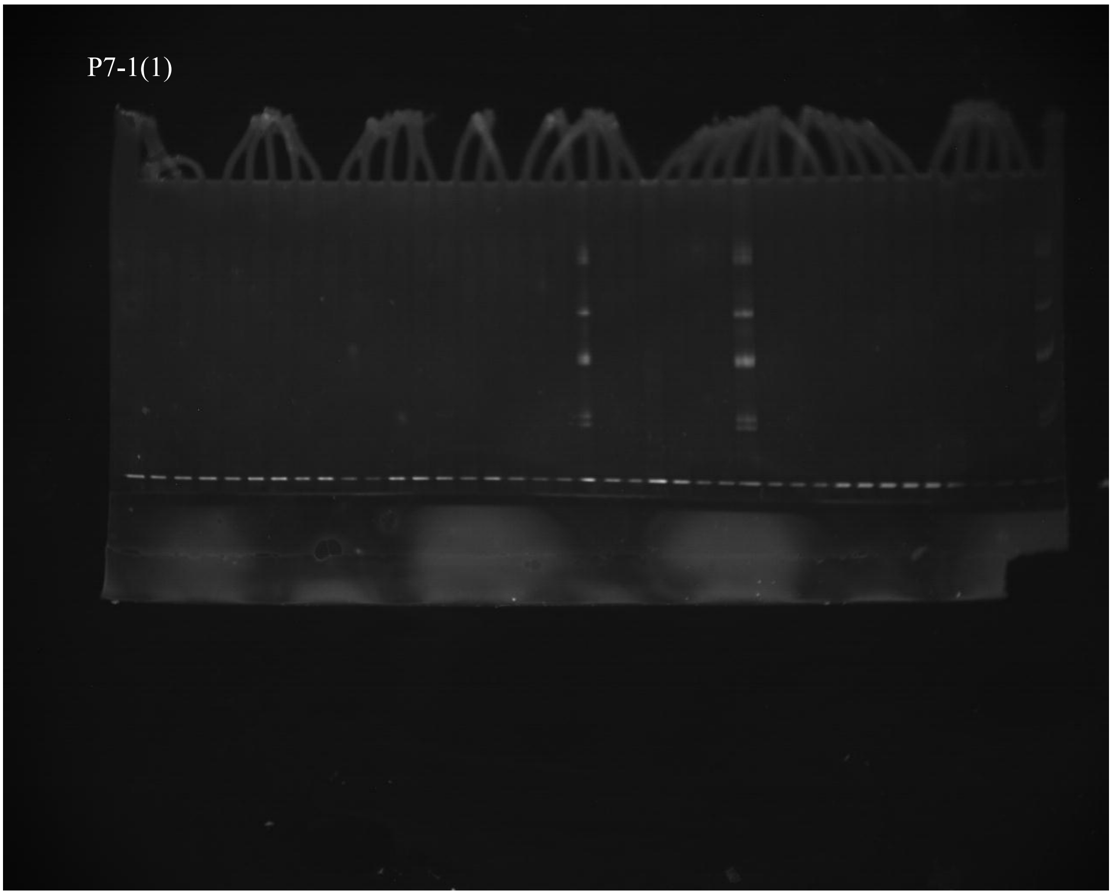
P7-1(1)

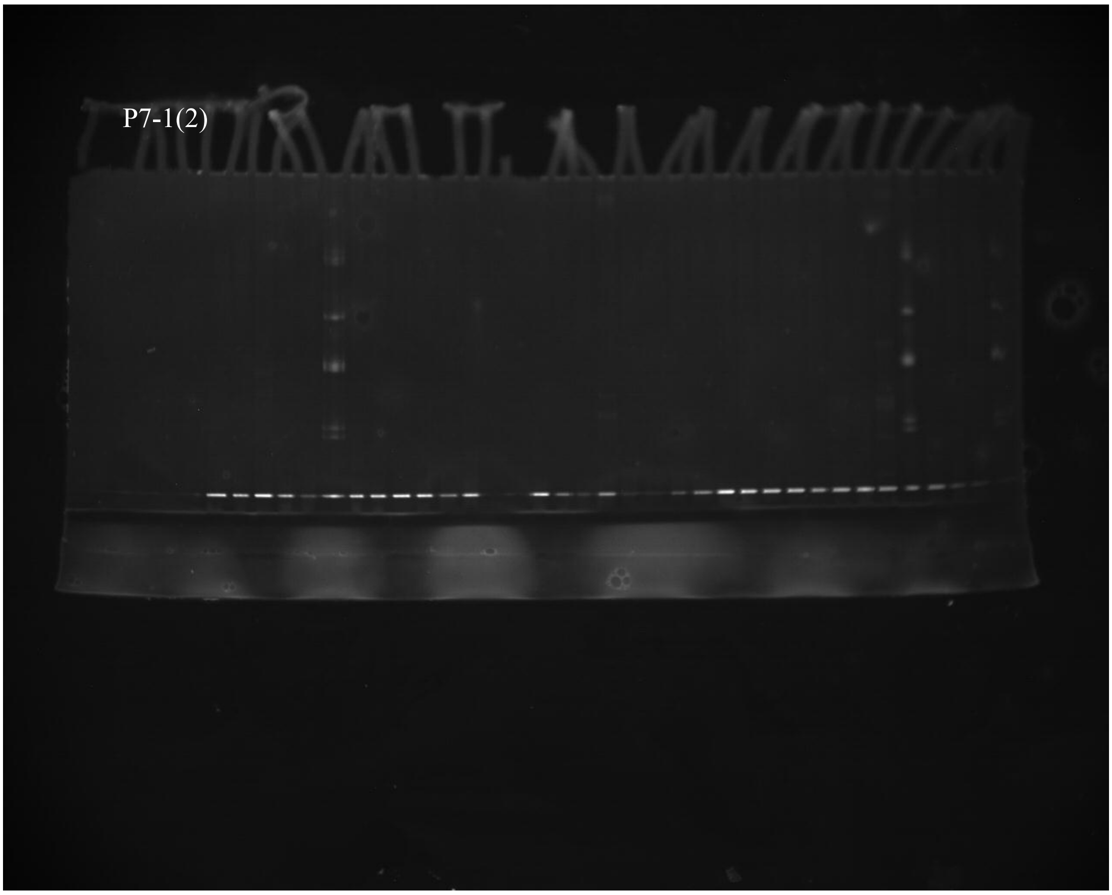
P7-1(2)

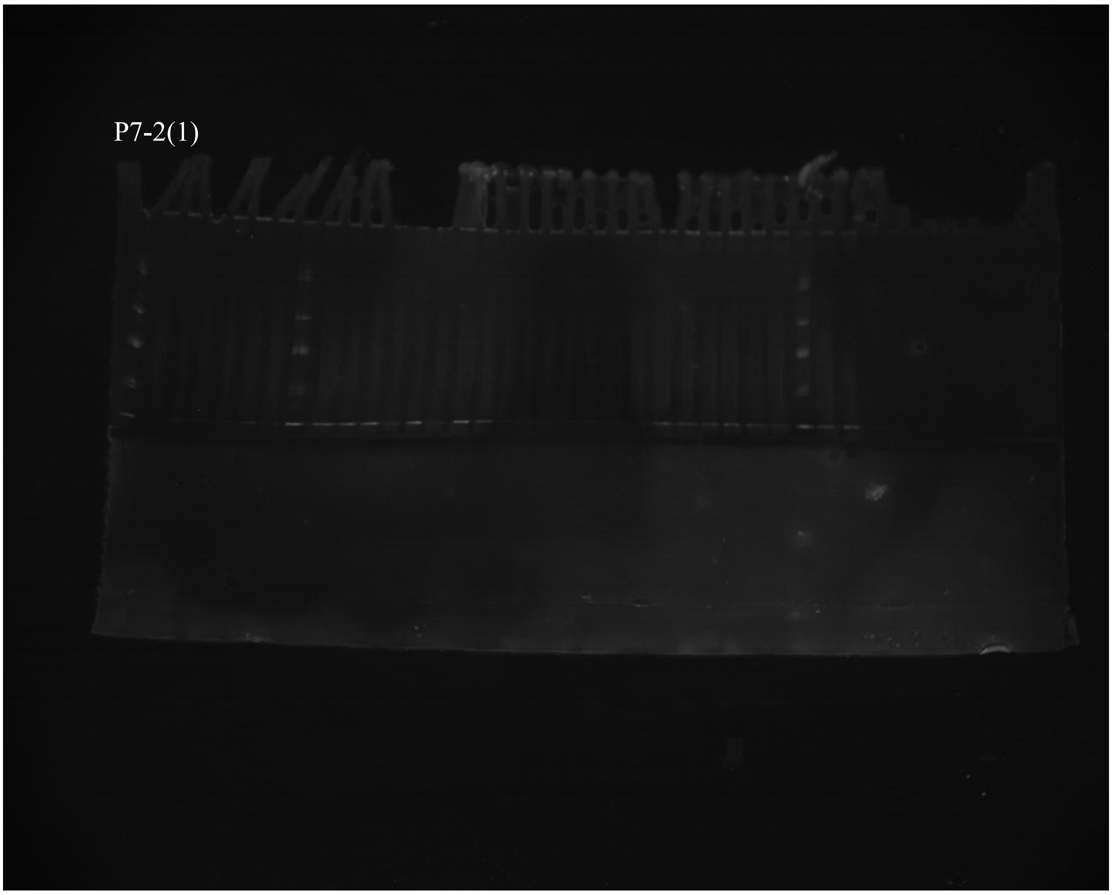
P7-2(1)

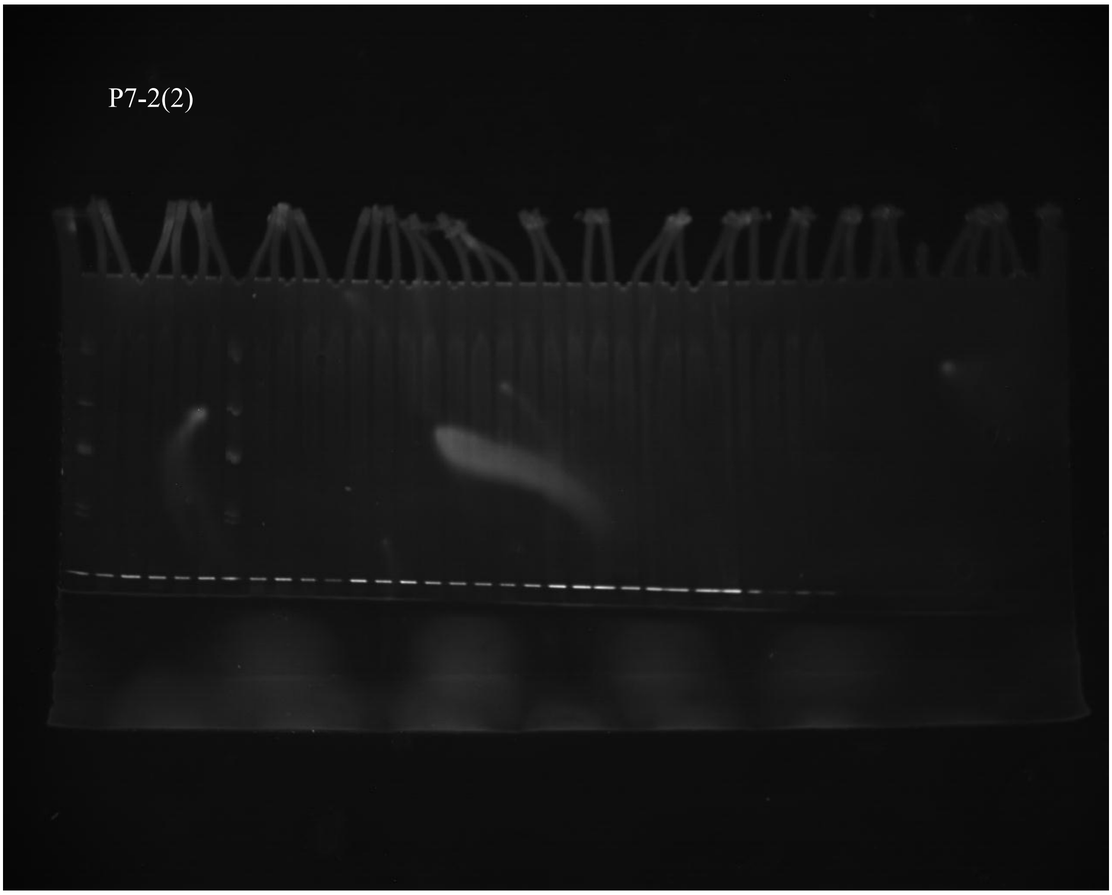
P7-2(2)

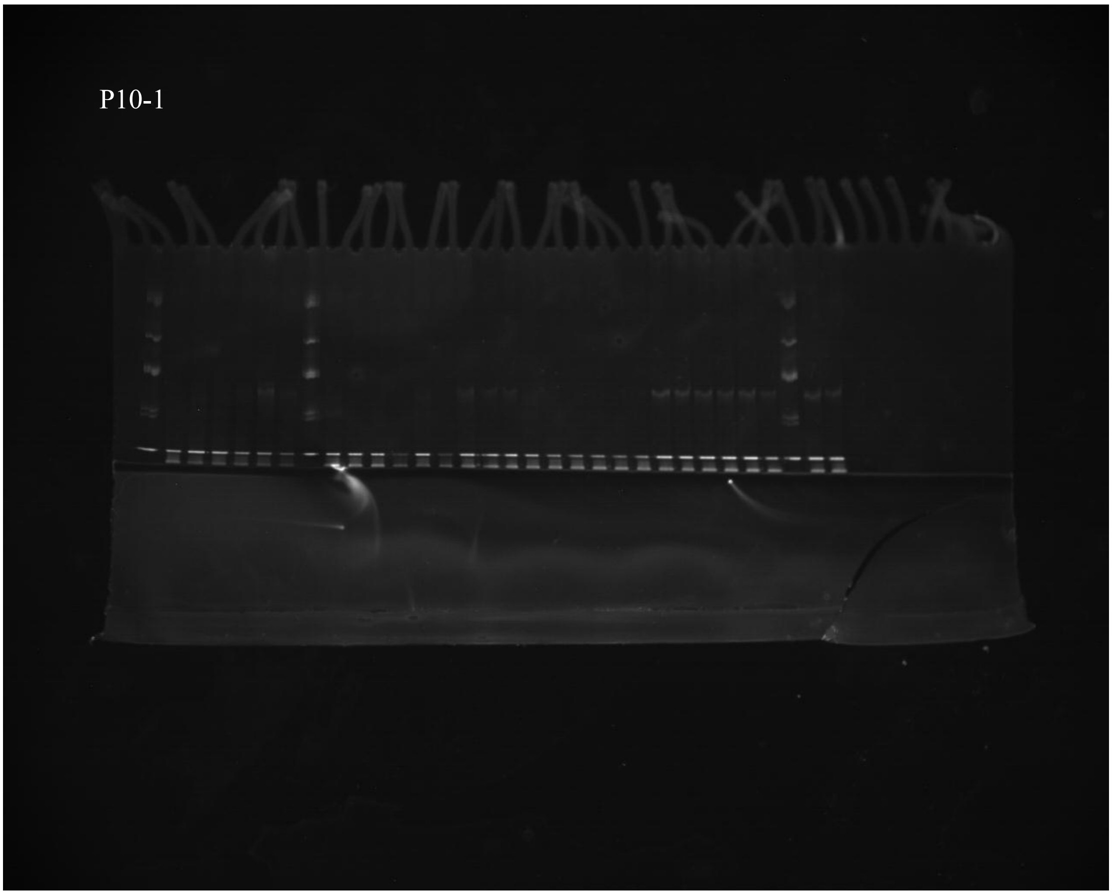
P10-1

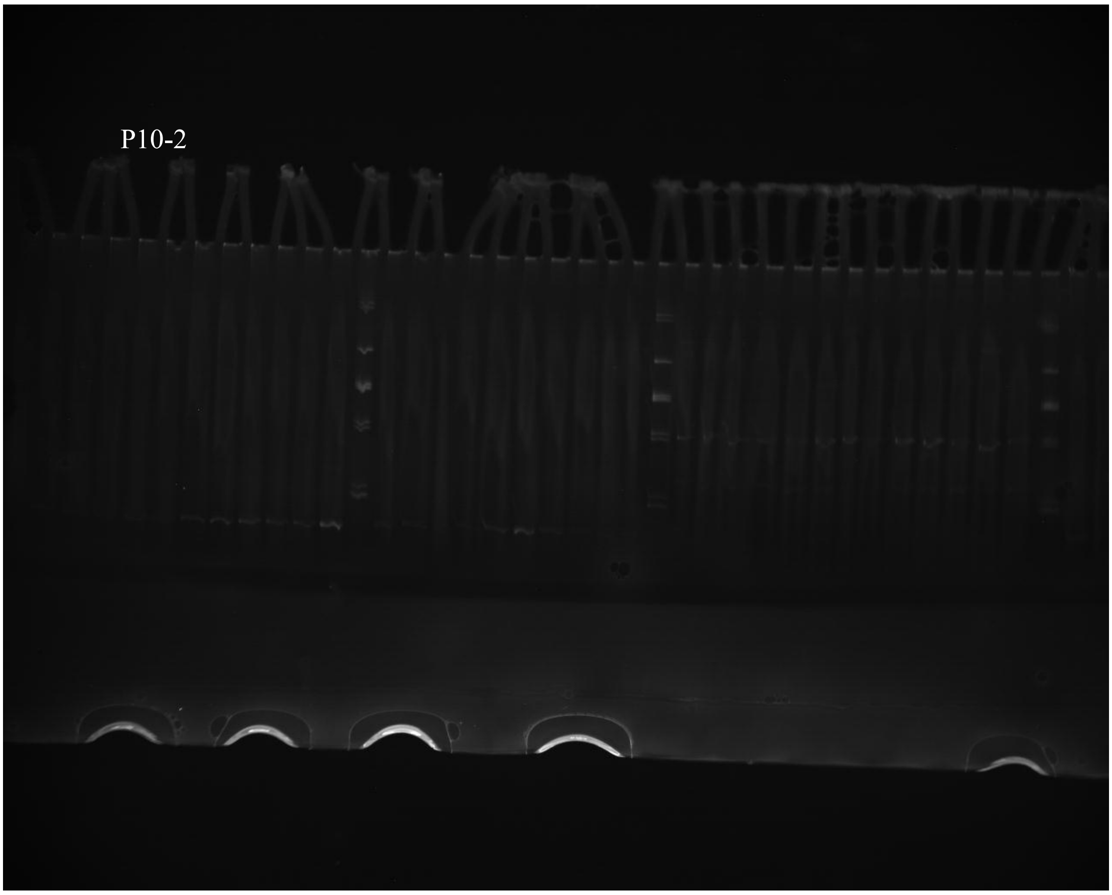
P10-2

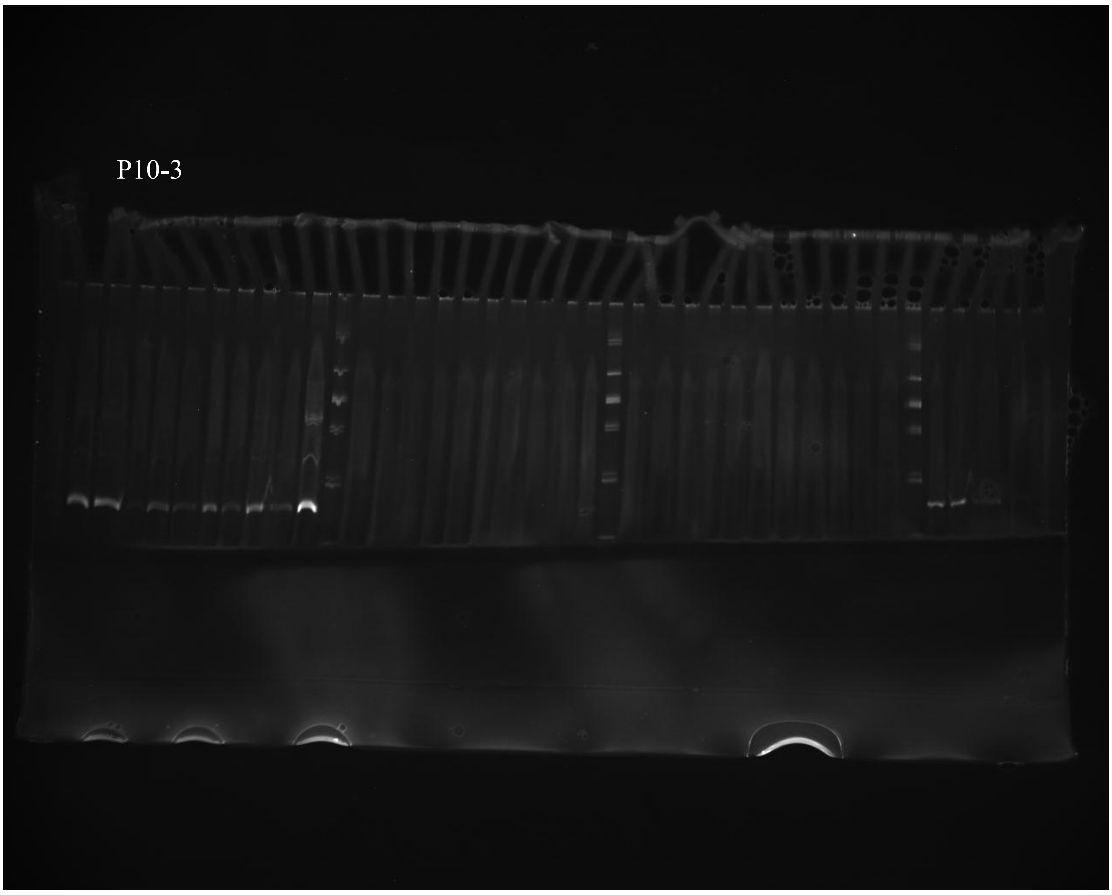
P10-3

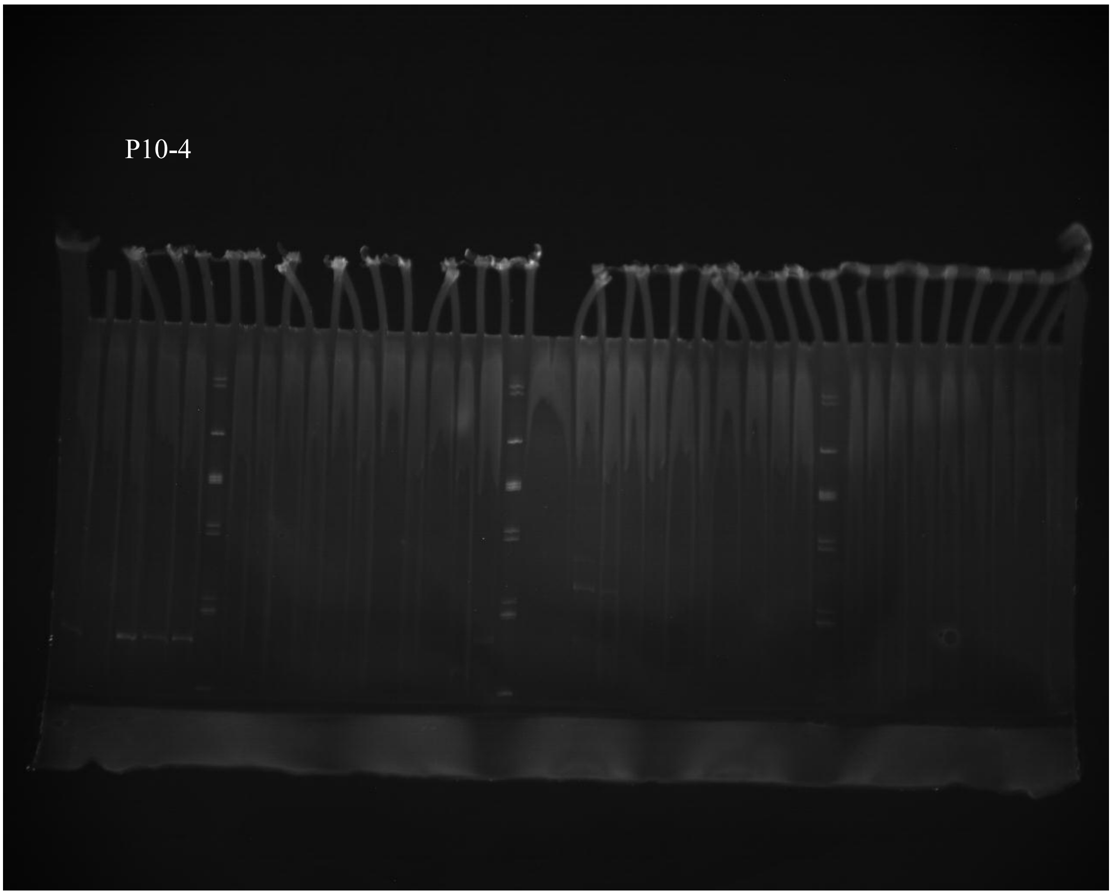
P10-4

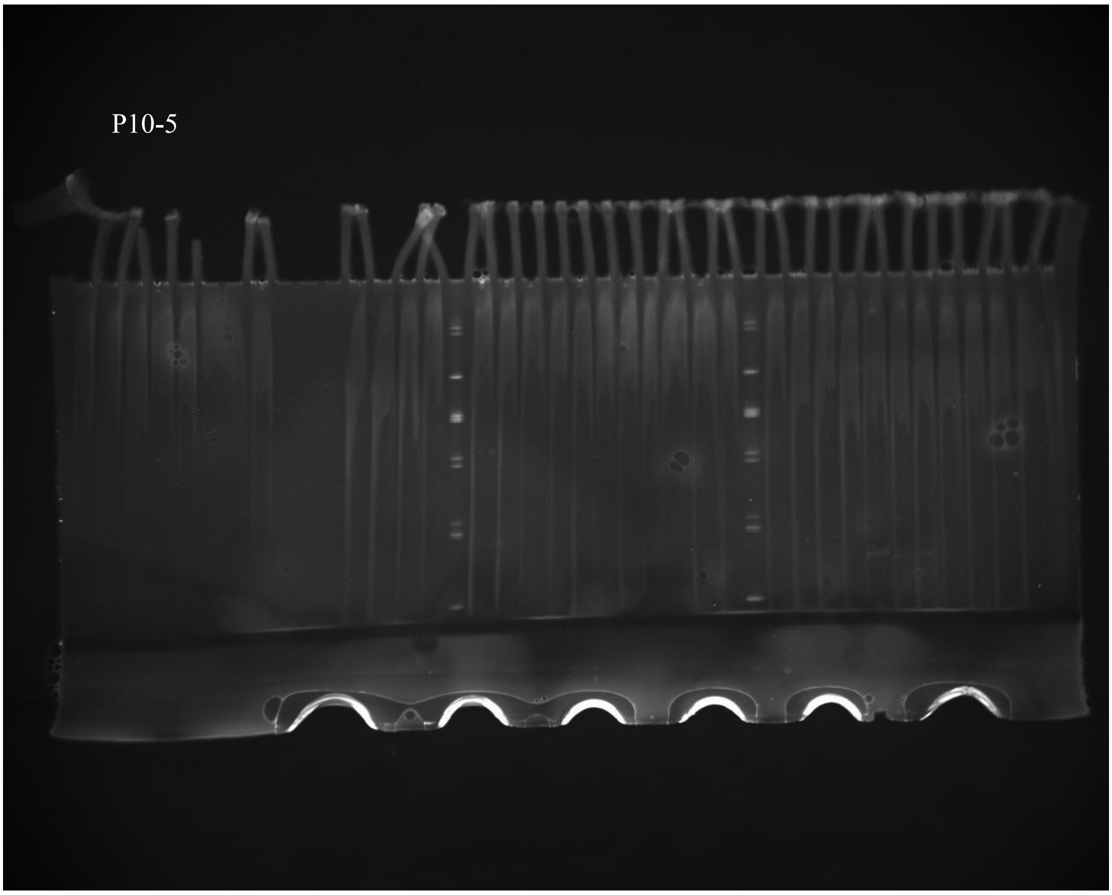
P10-5

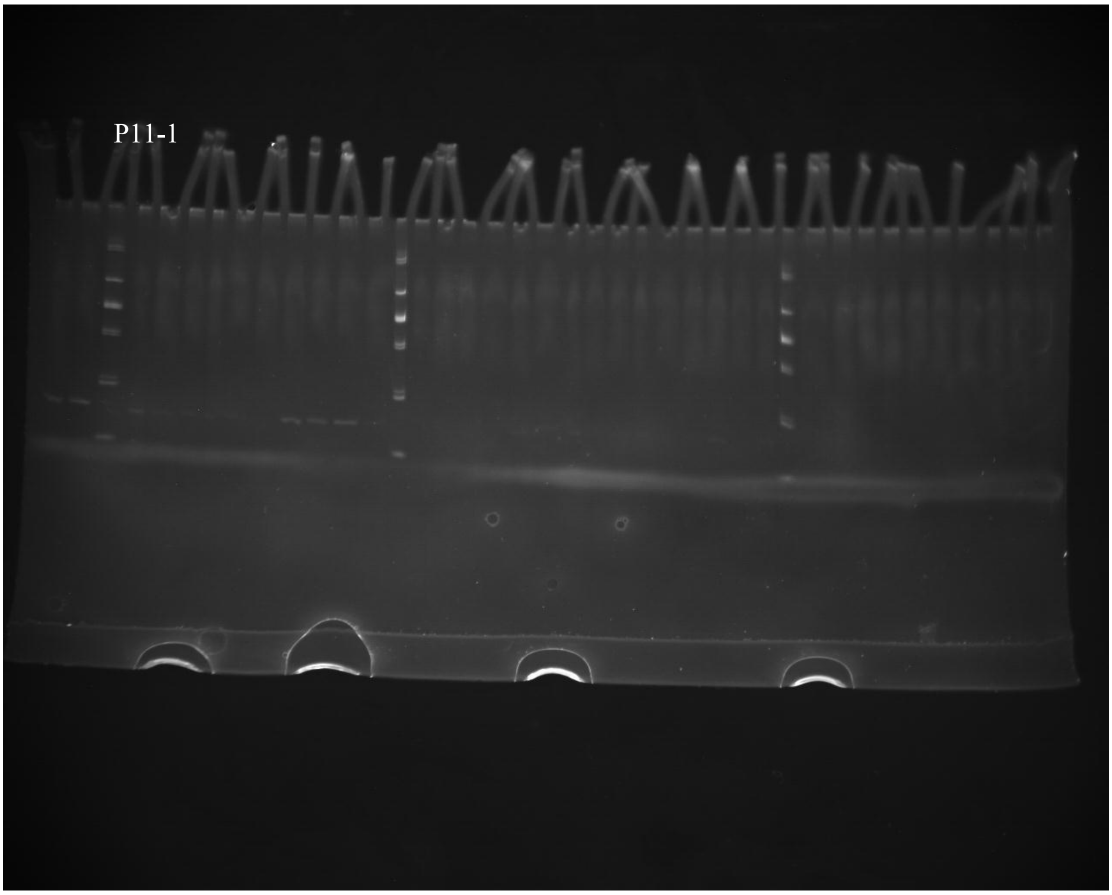
P11-1

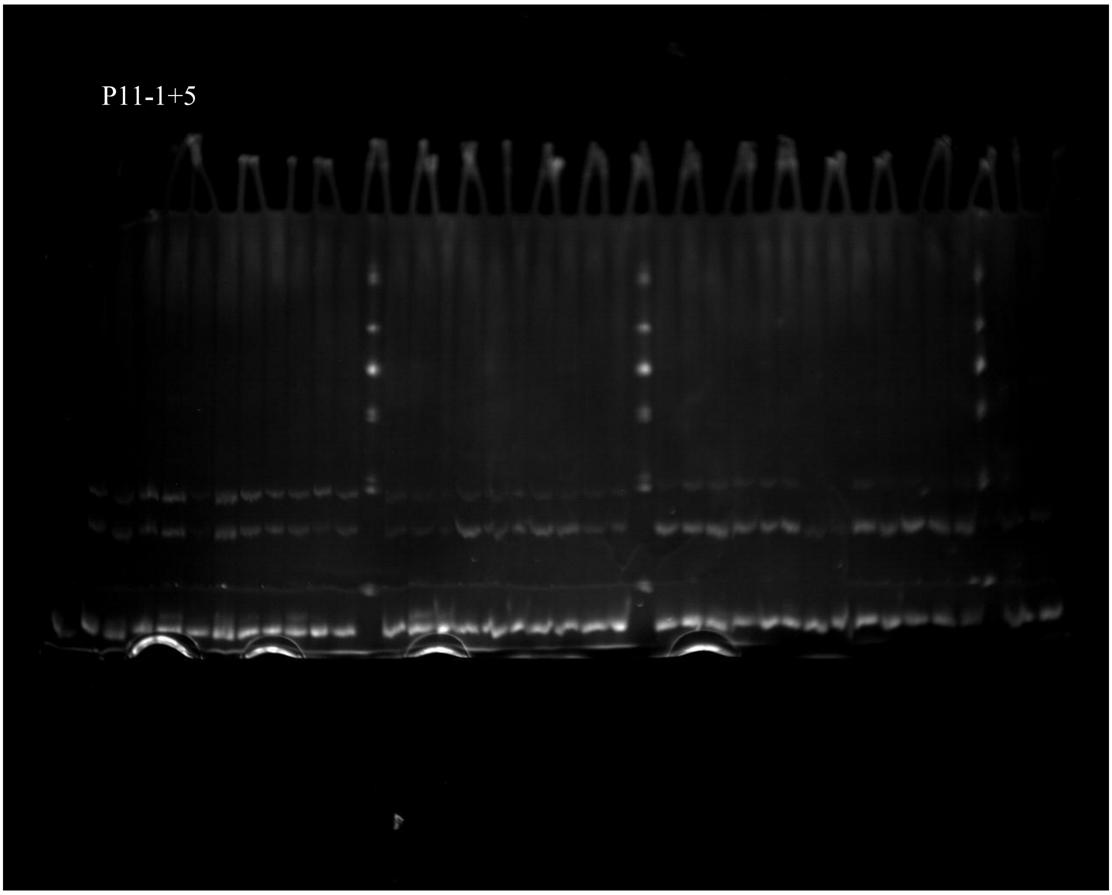
P11-1+5

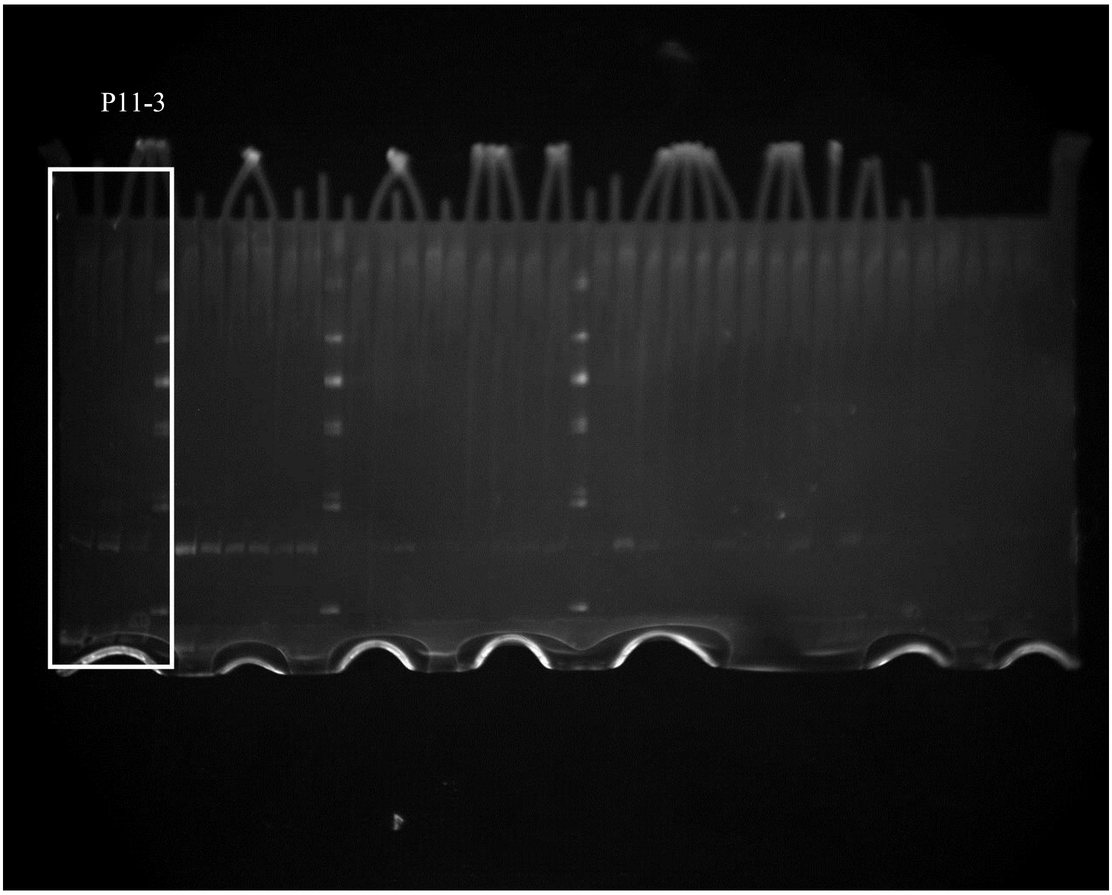
P11-3

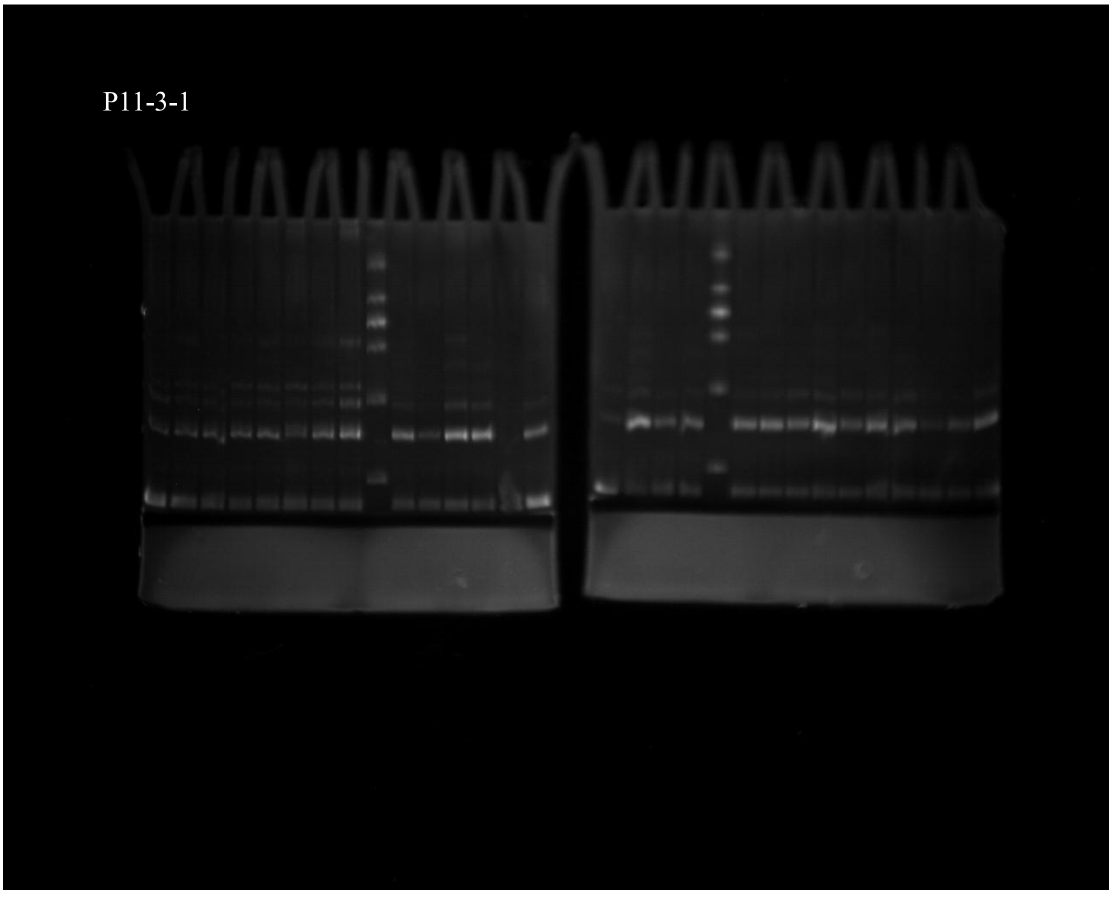
P11-3-1

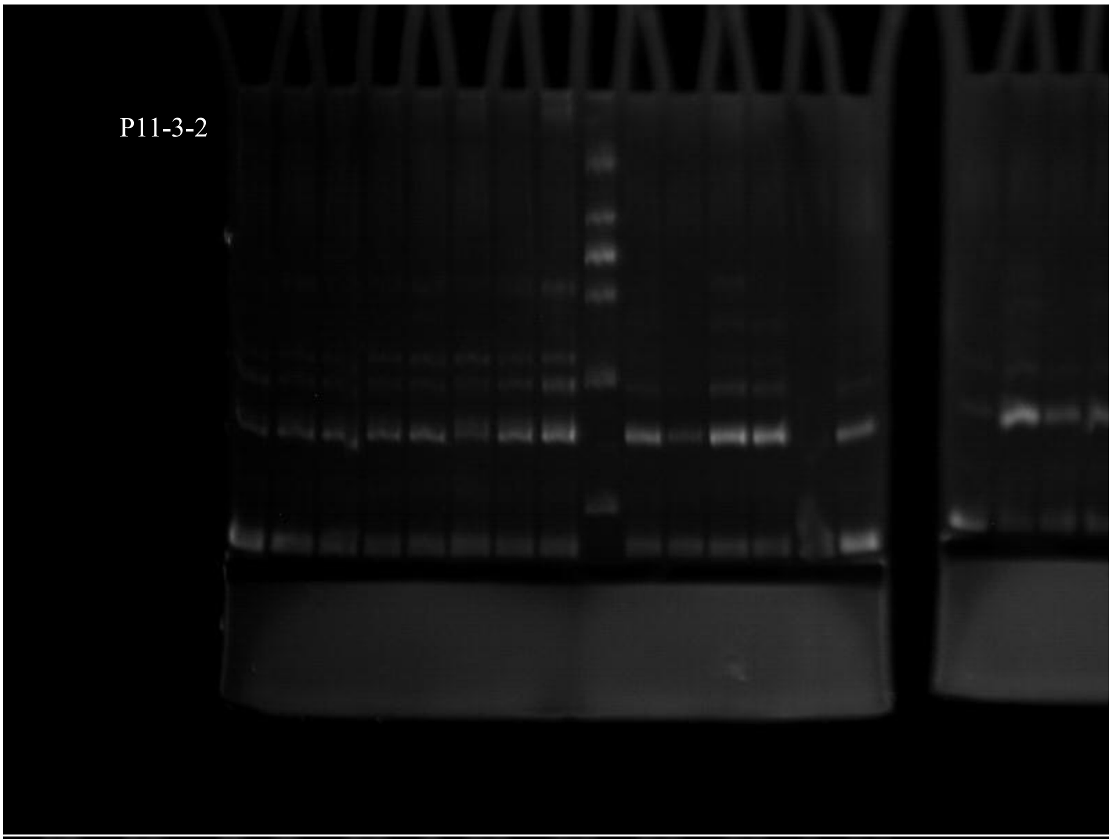
P11-3-2

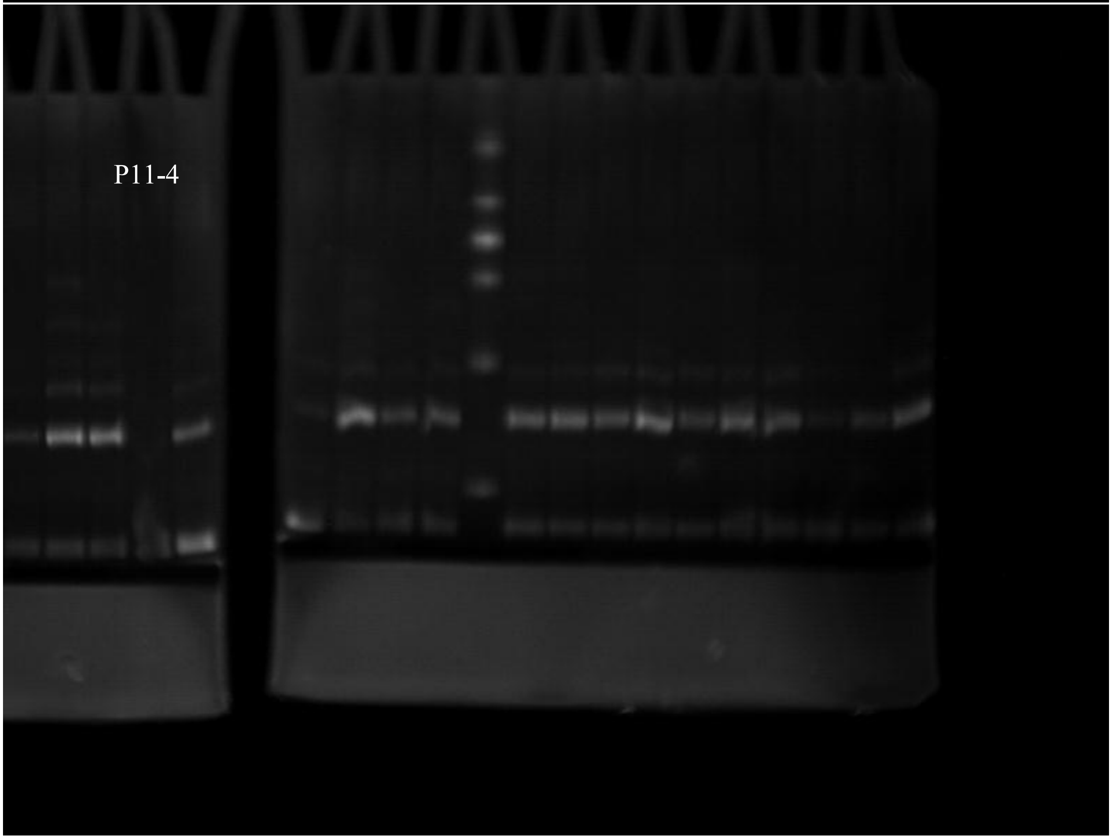
P11-4

P11-4-1

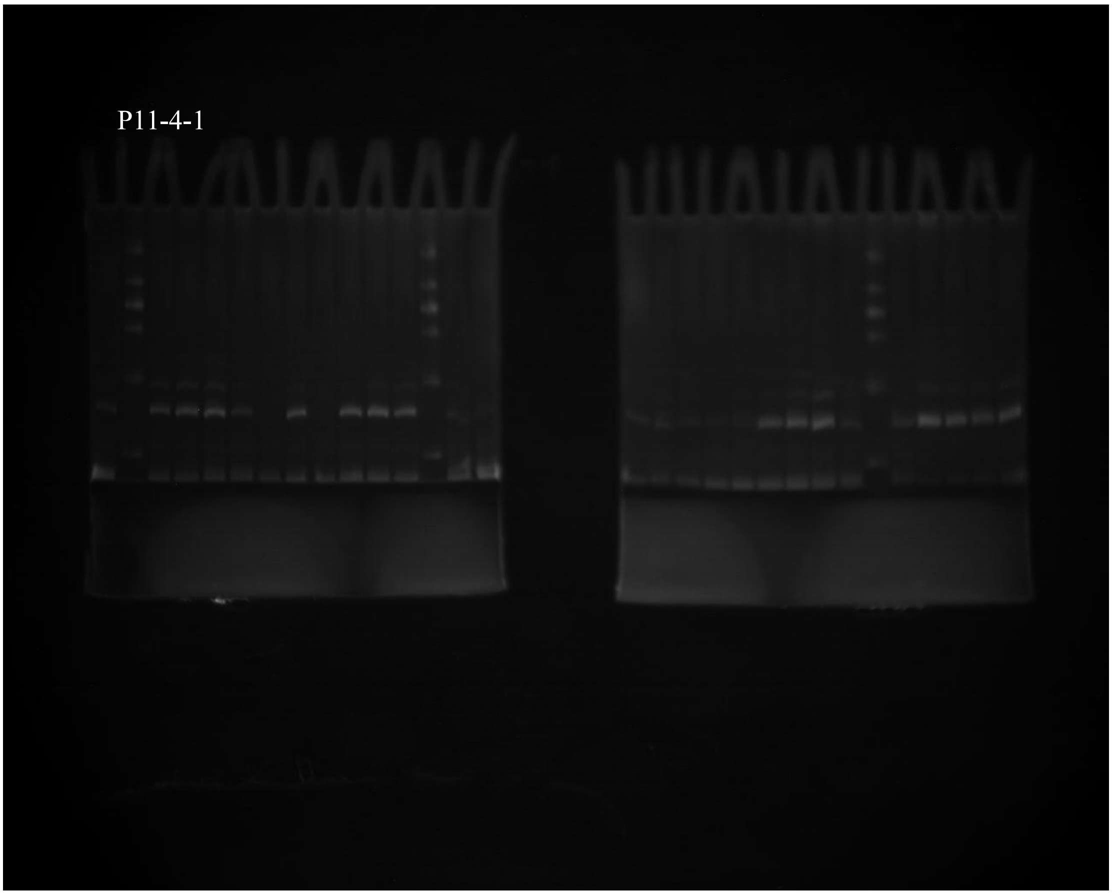

P11-4-2

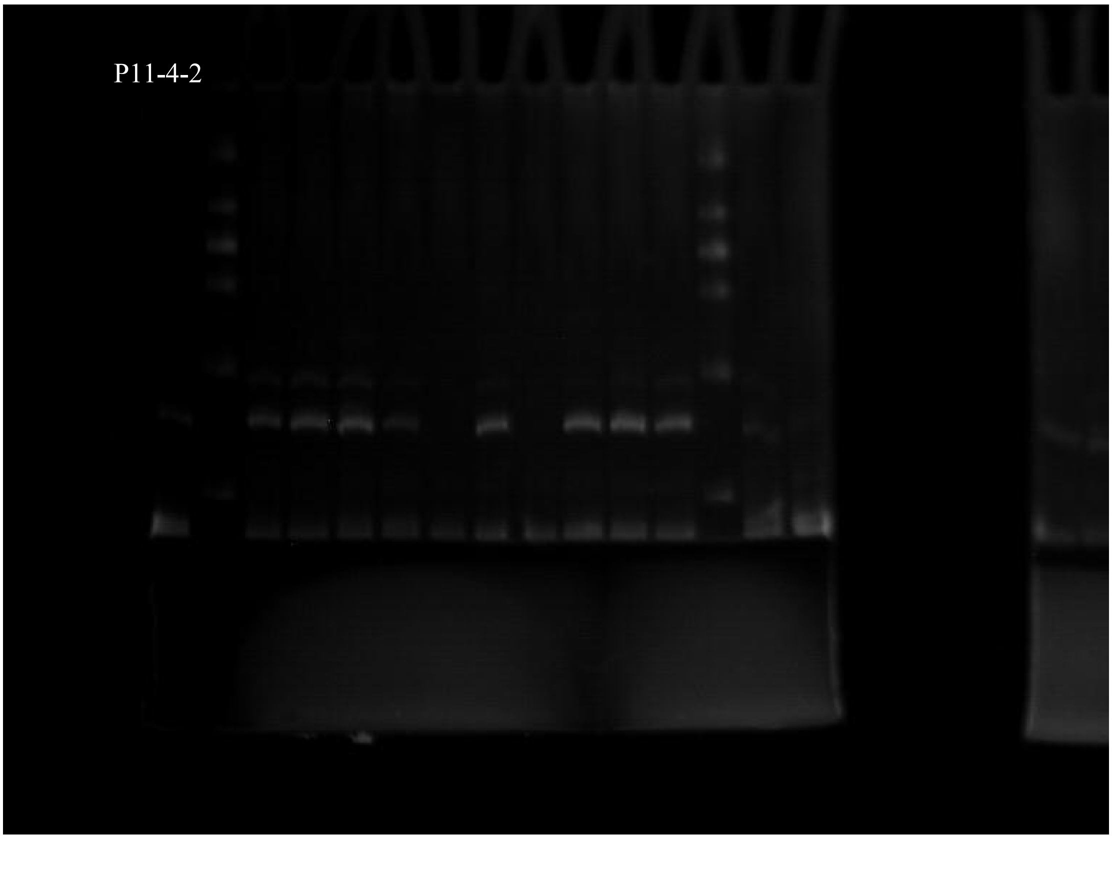

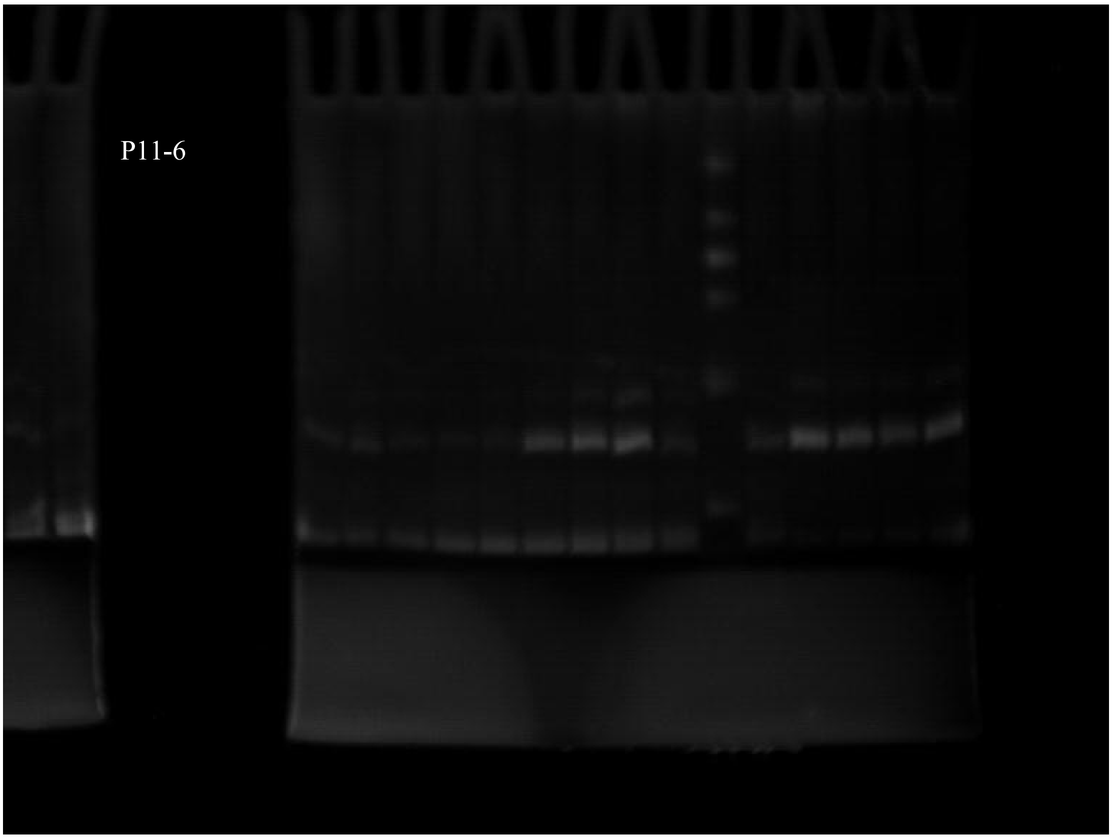
P11-6

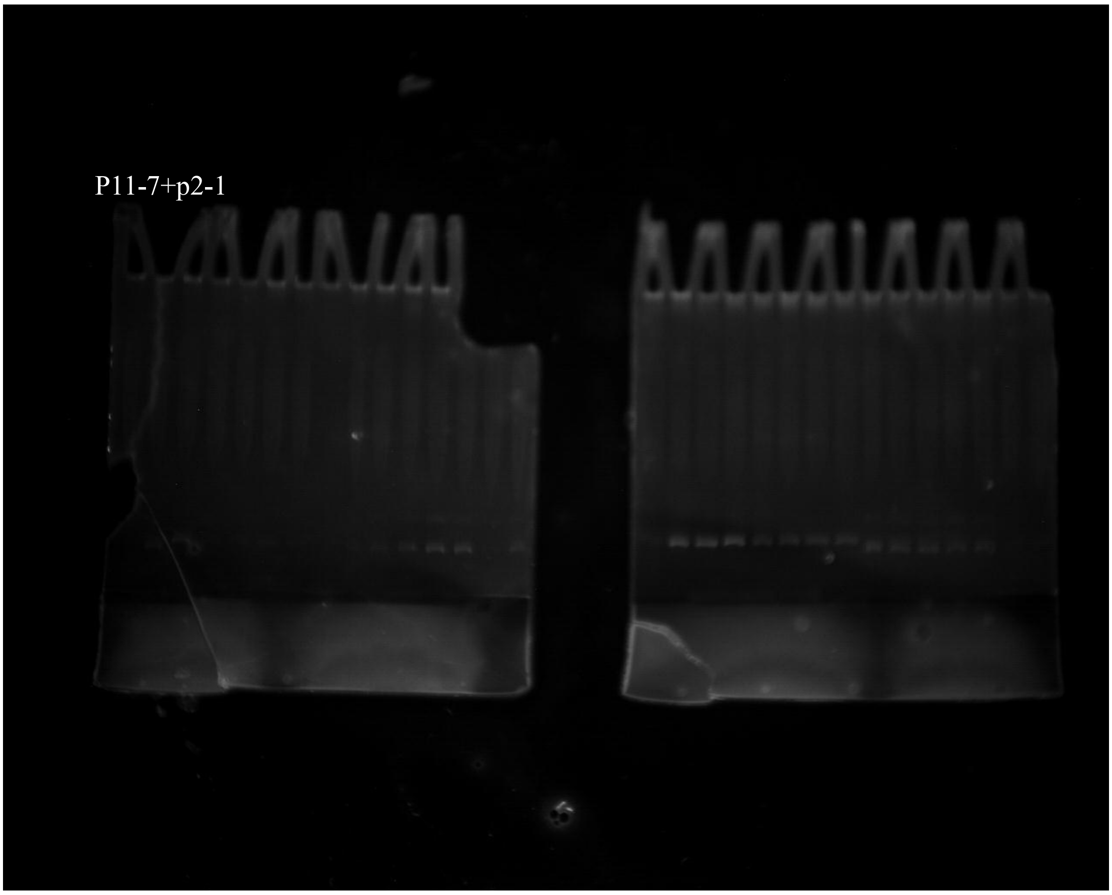
P11-7+p2-1

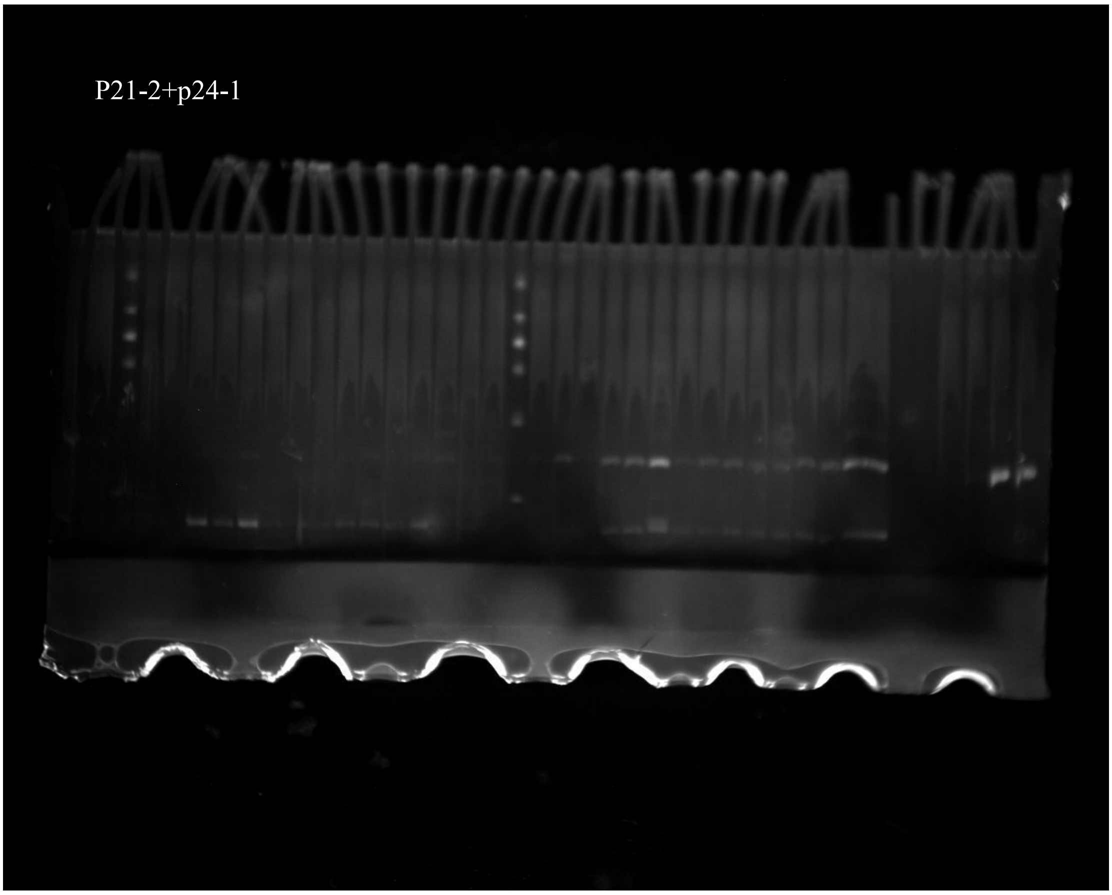
P21-2+p24-1

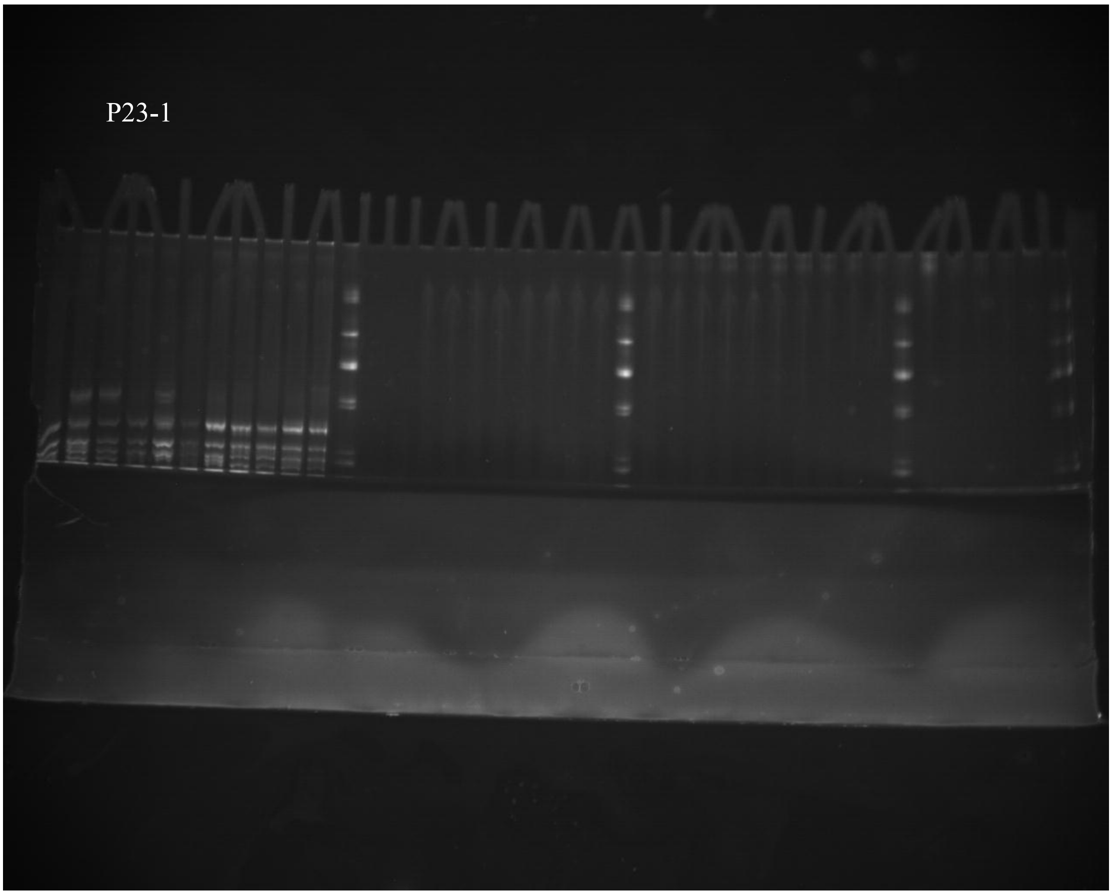
P23-1

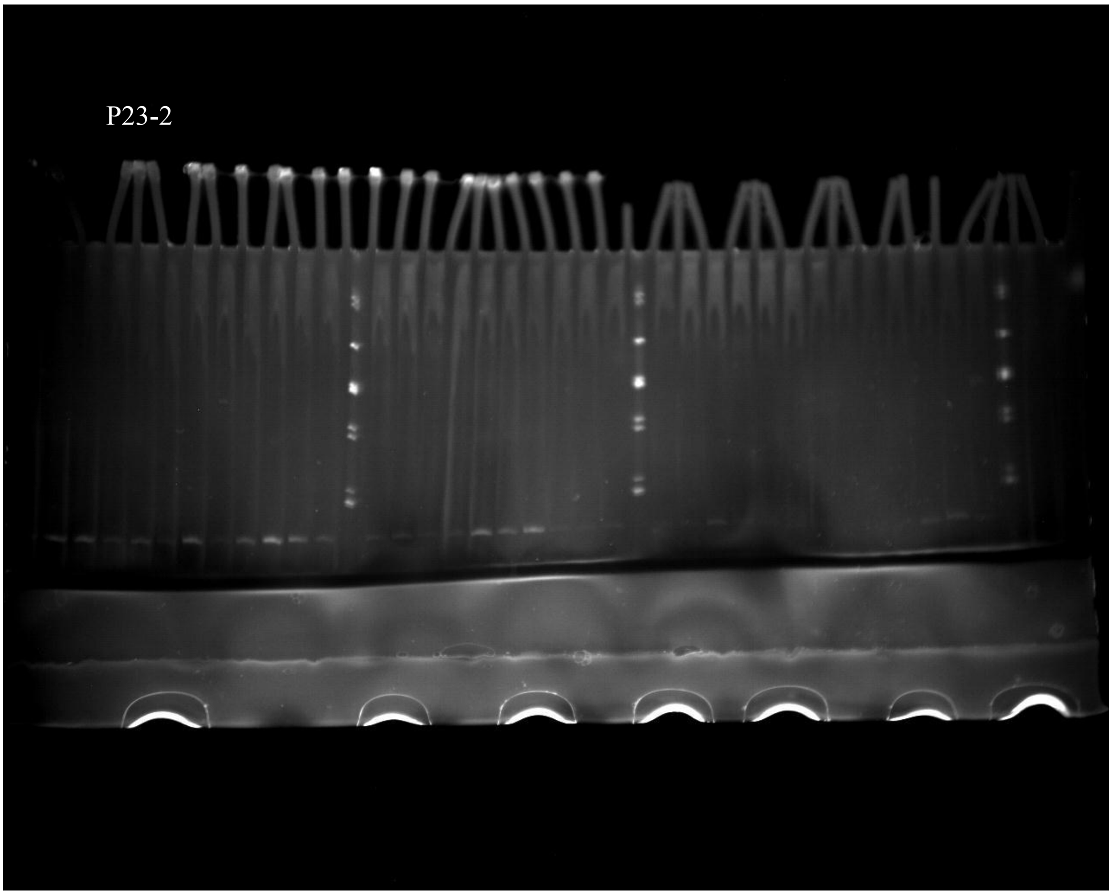
P23-2

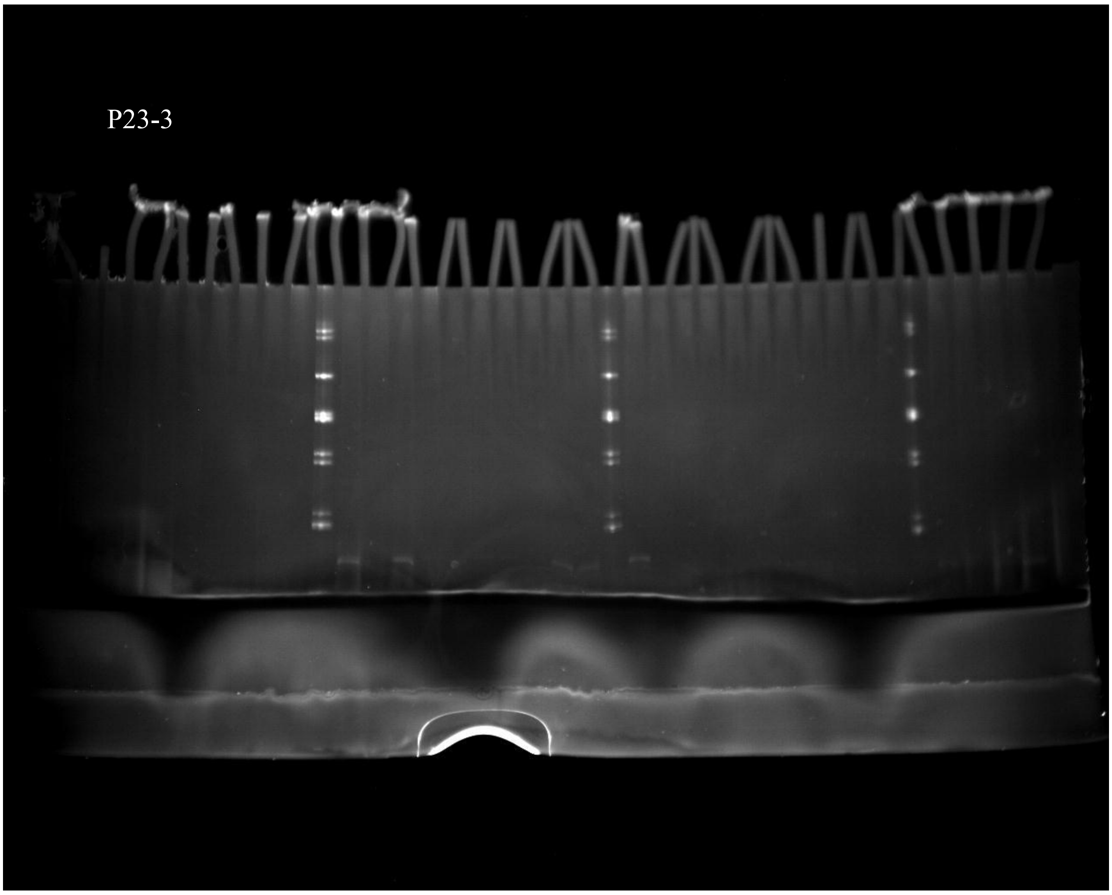
P23-3

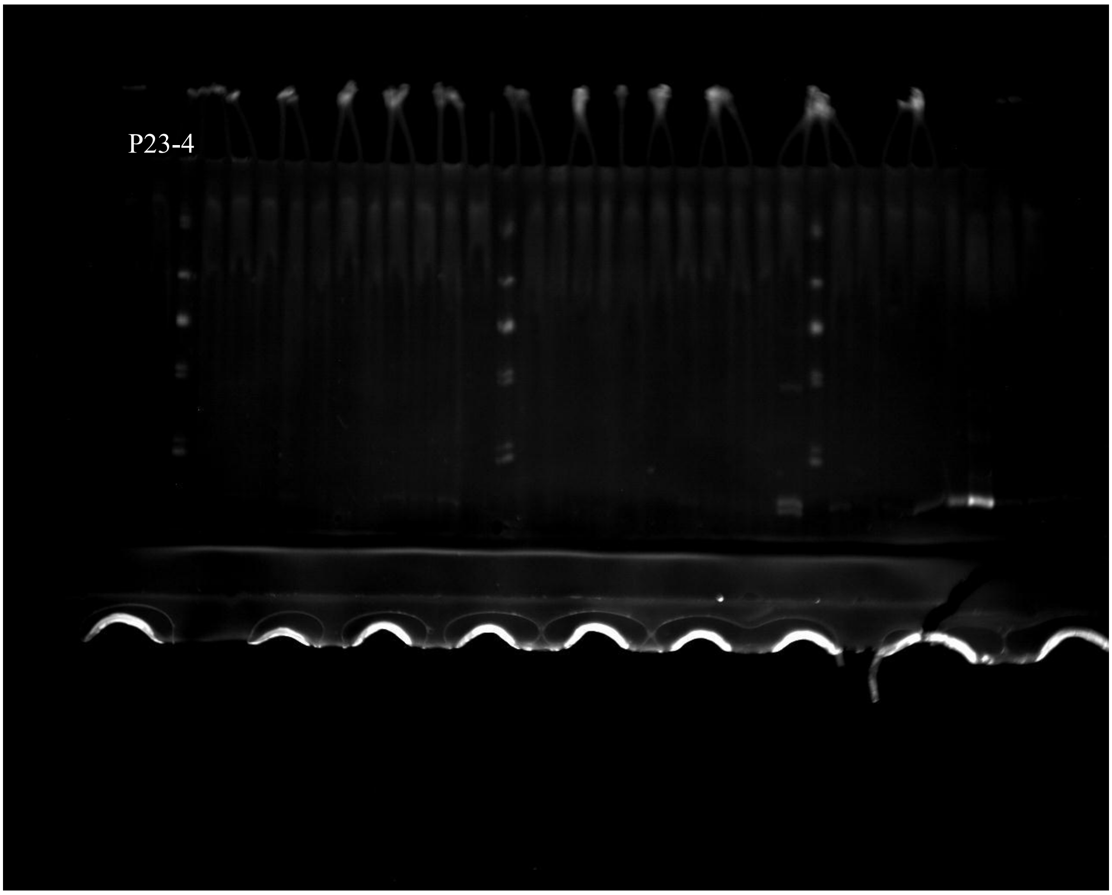
P23-4

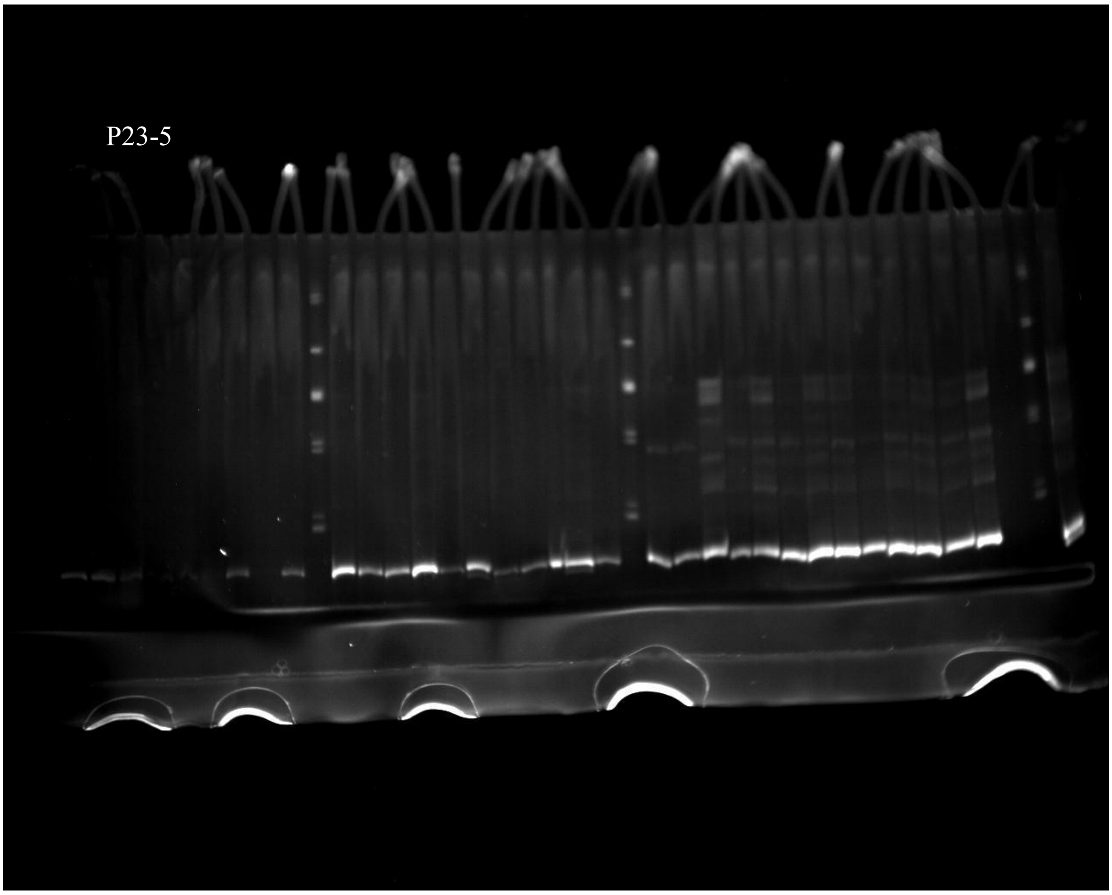
P23-5

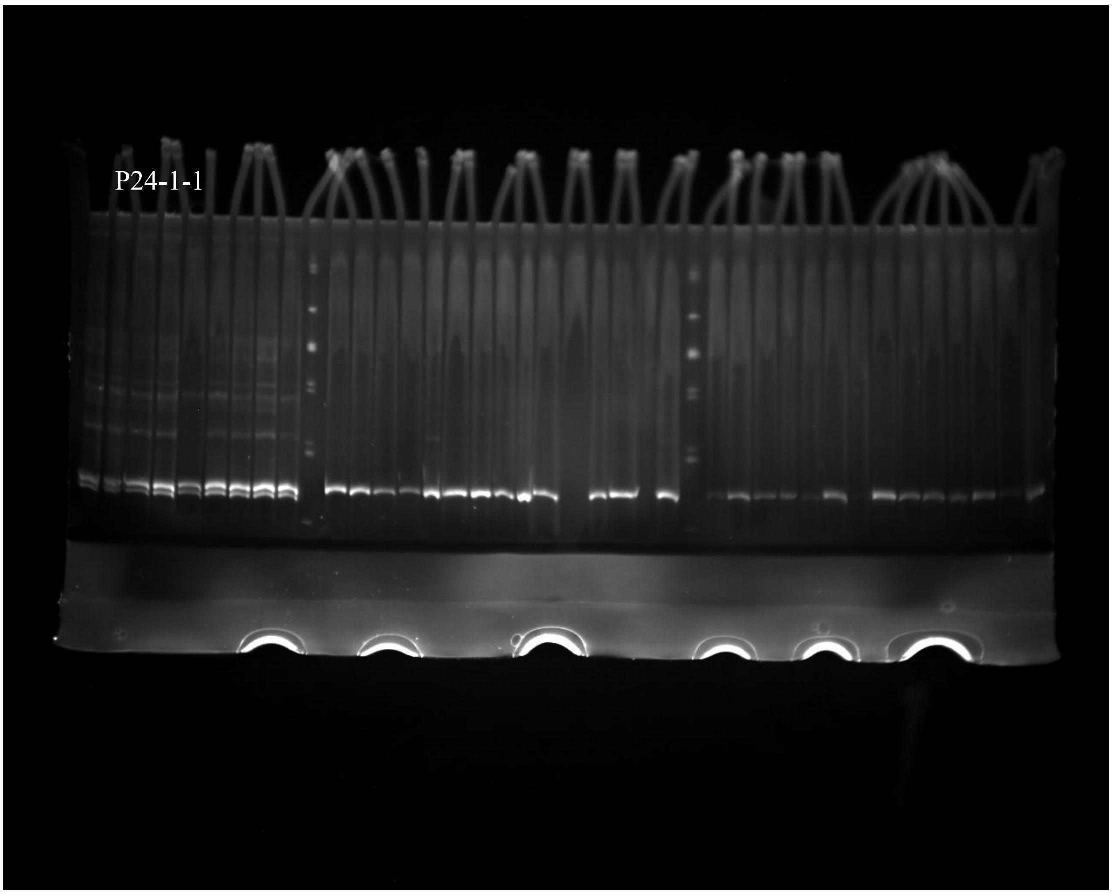
P24-1-1

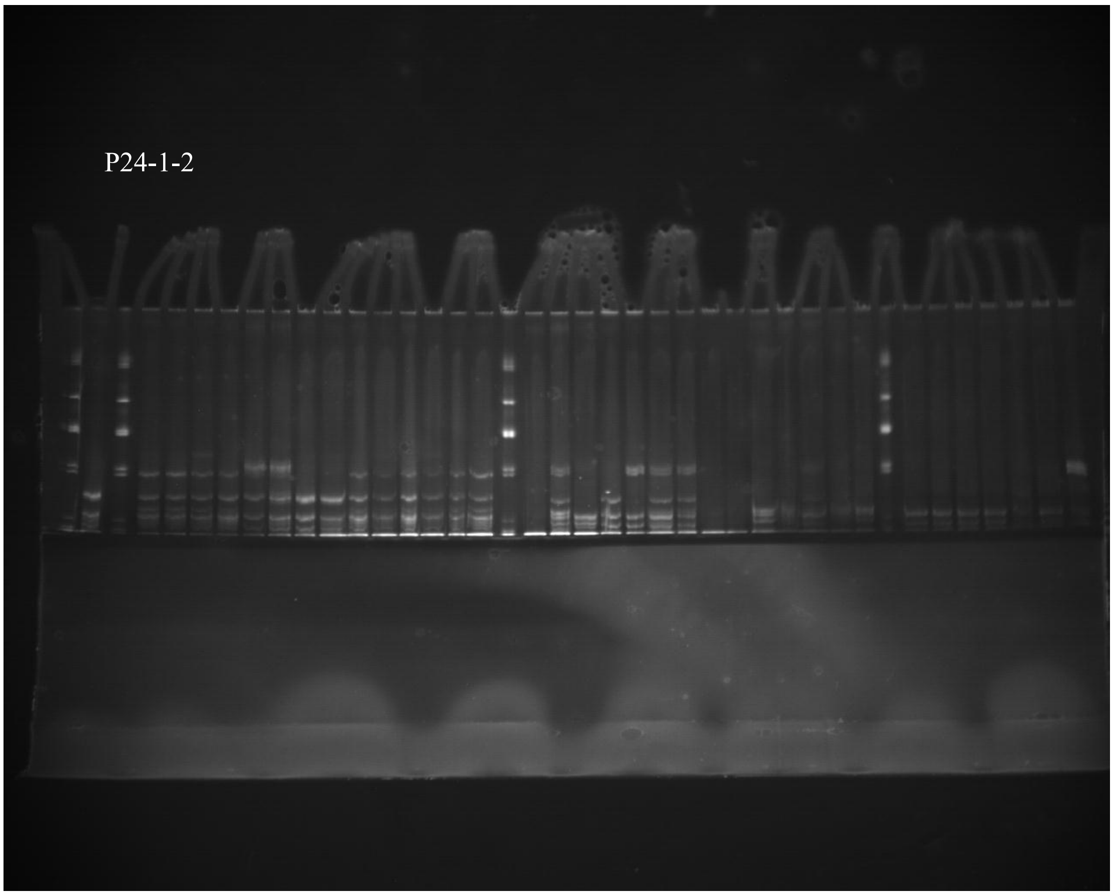
P24-1-2

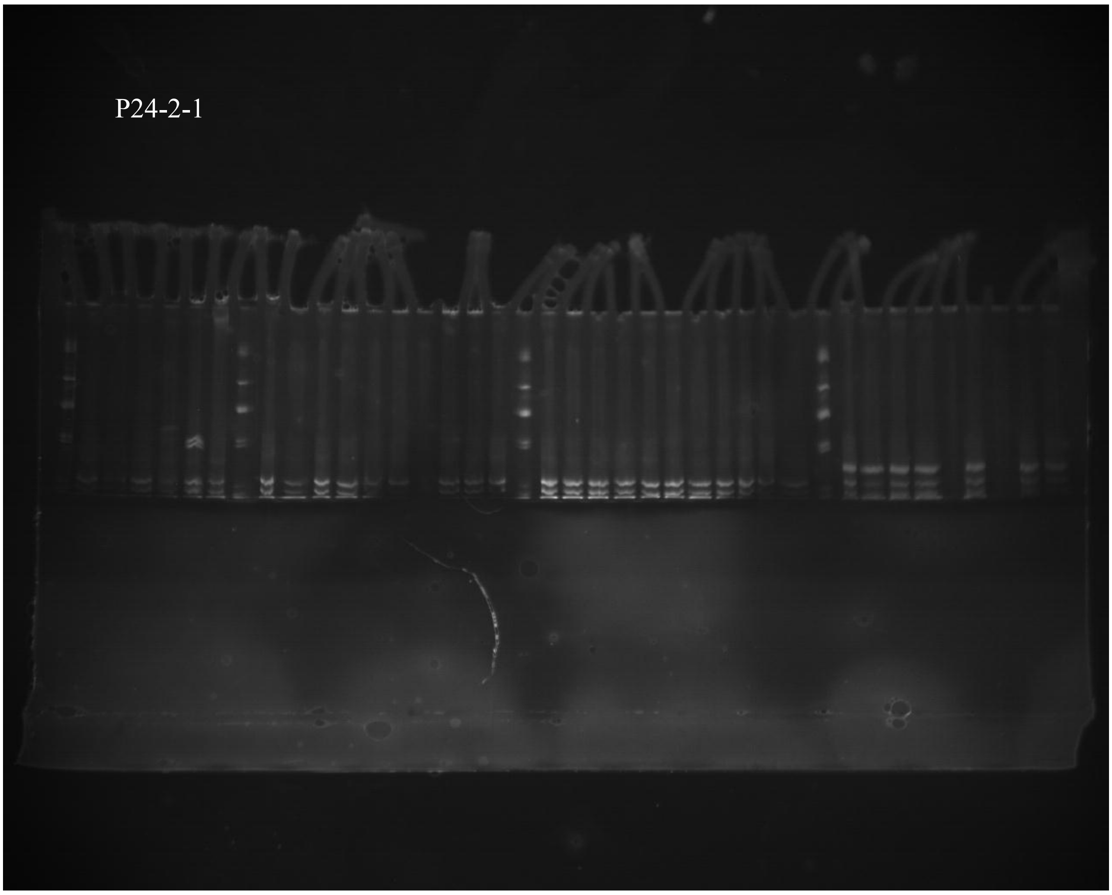
P24-2-1

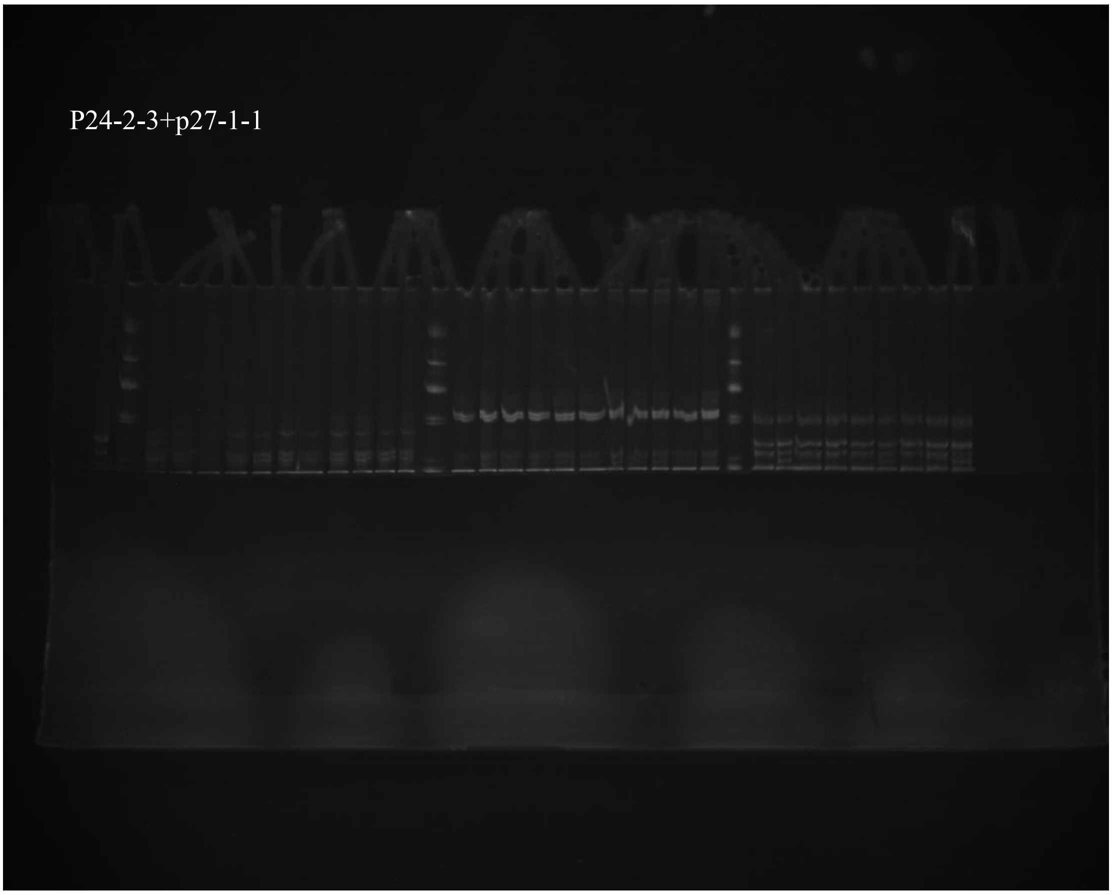
P24-2-3+p27-1-1

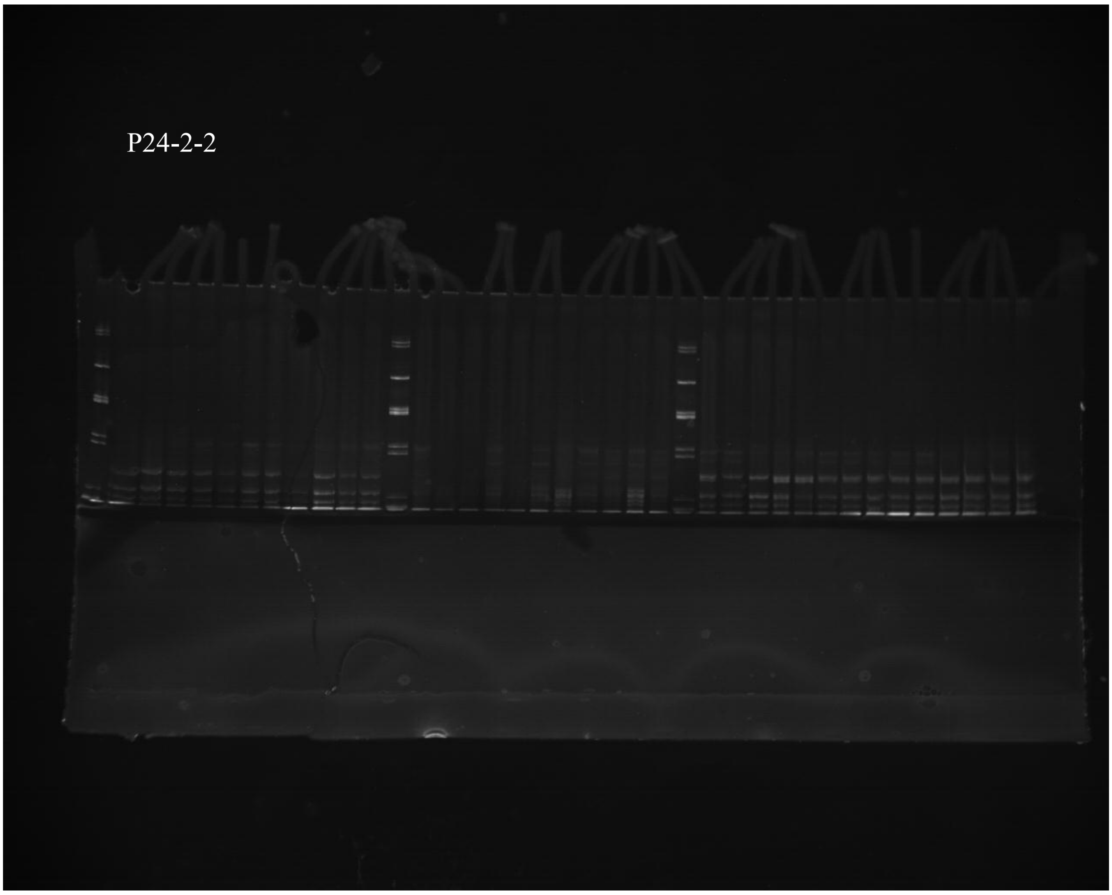
P24-2-2

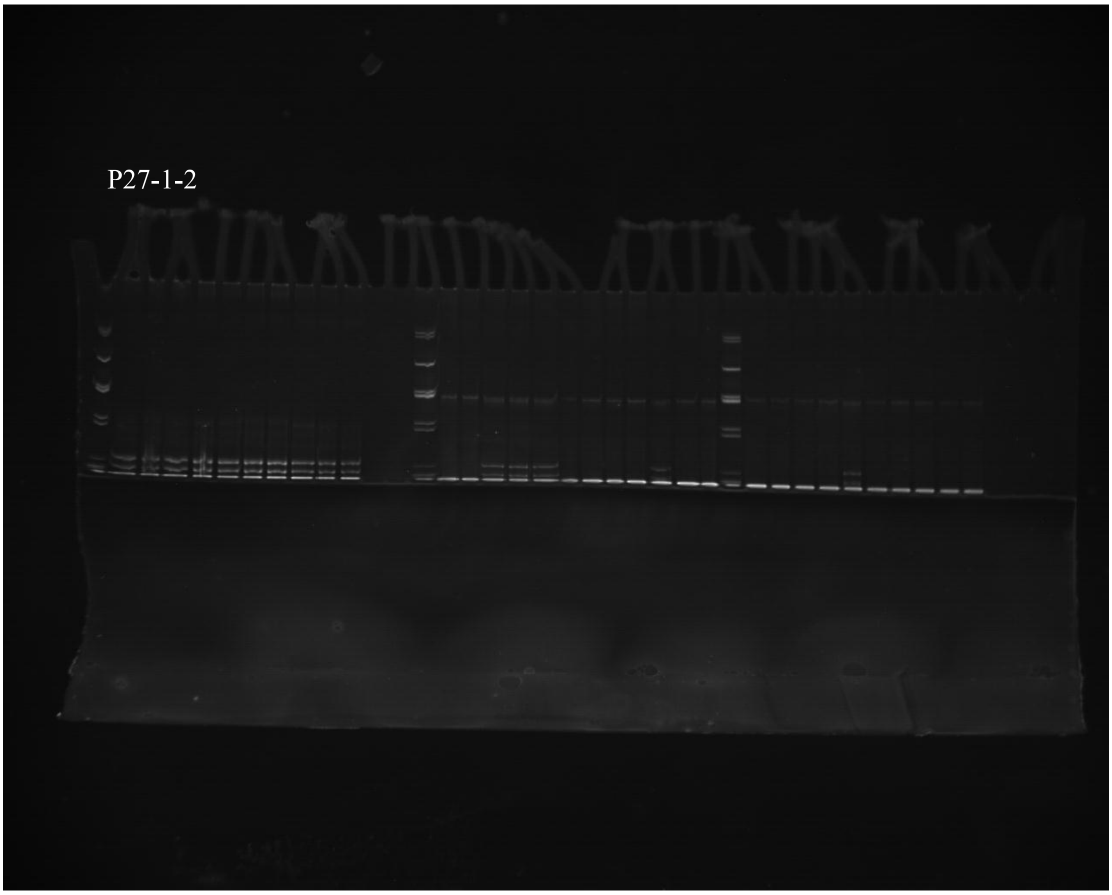
P27-1-2

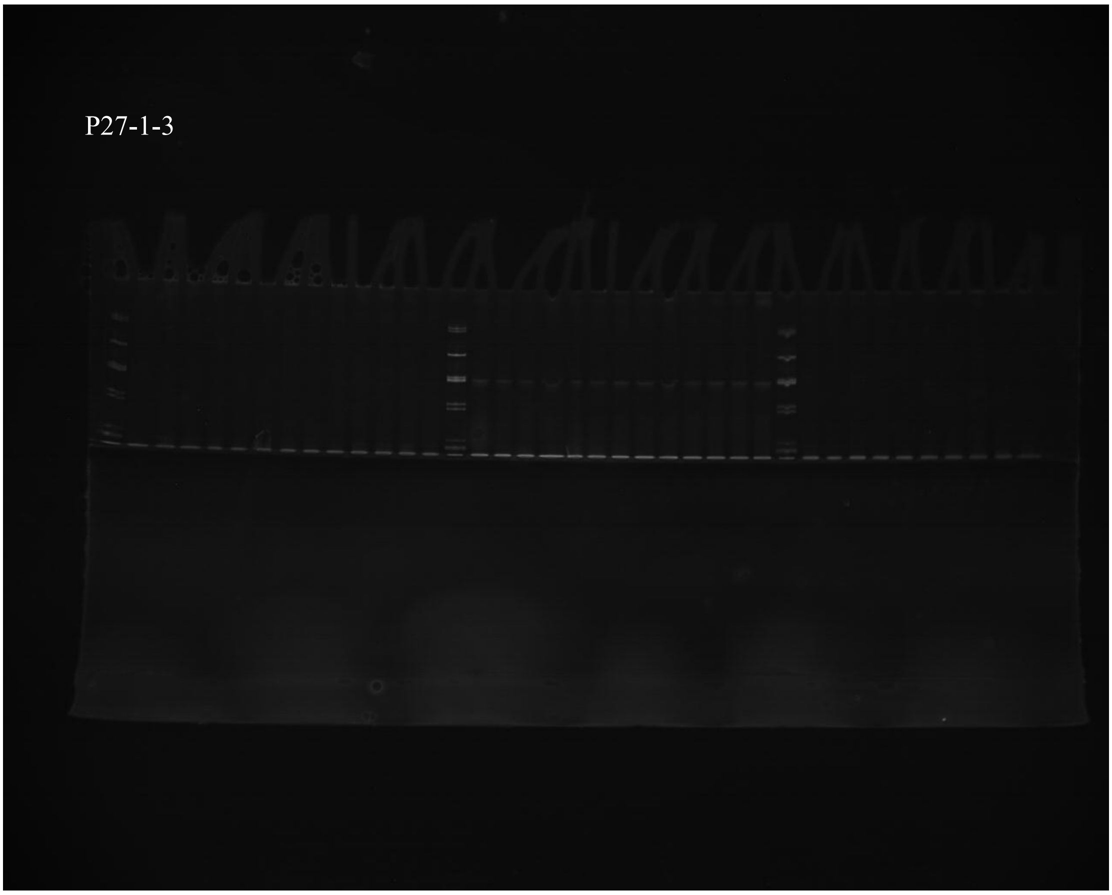
P27-1-3

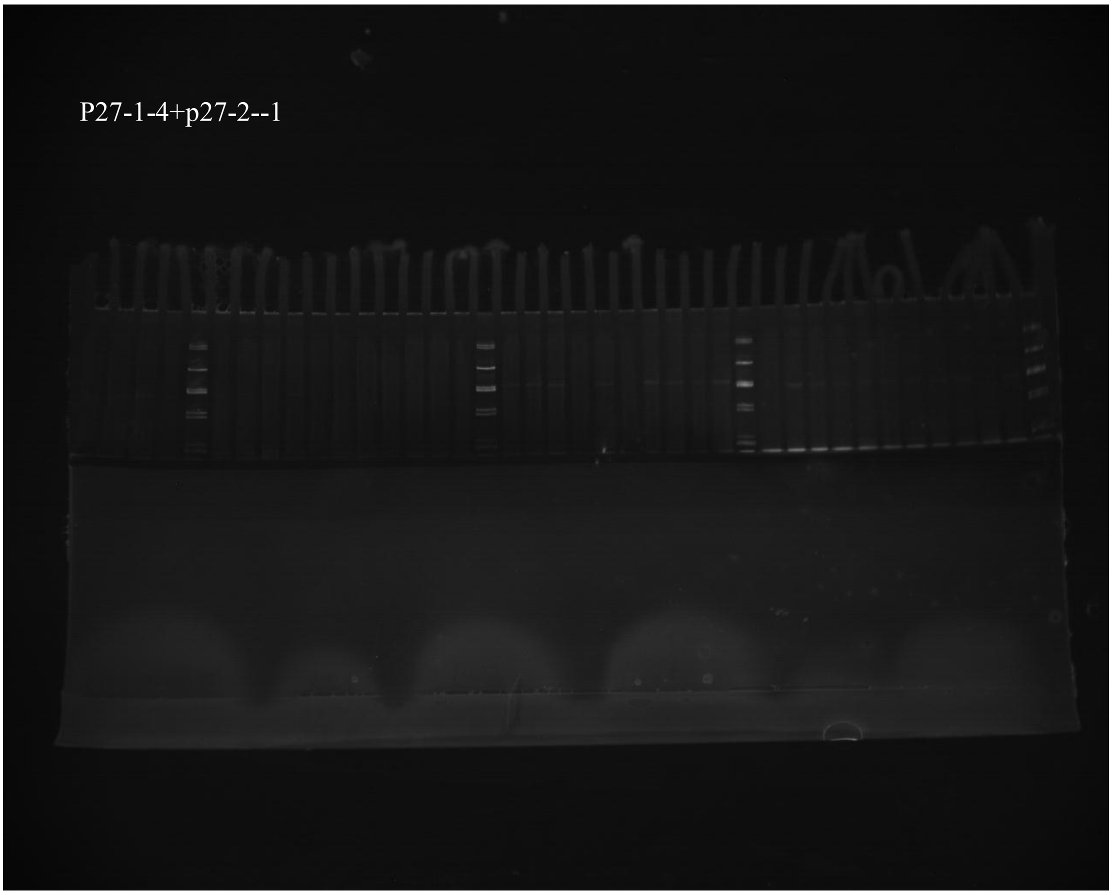
P27-1-4+p27-2--1

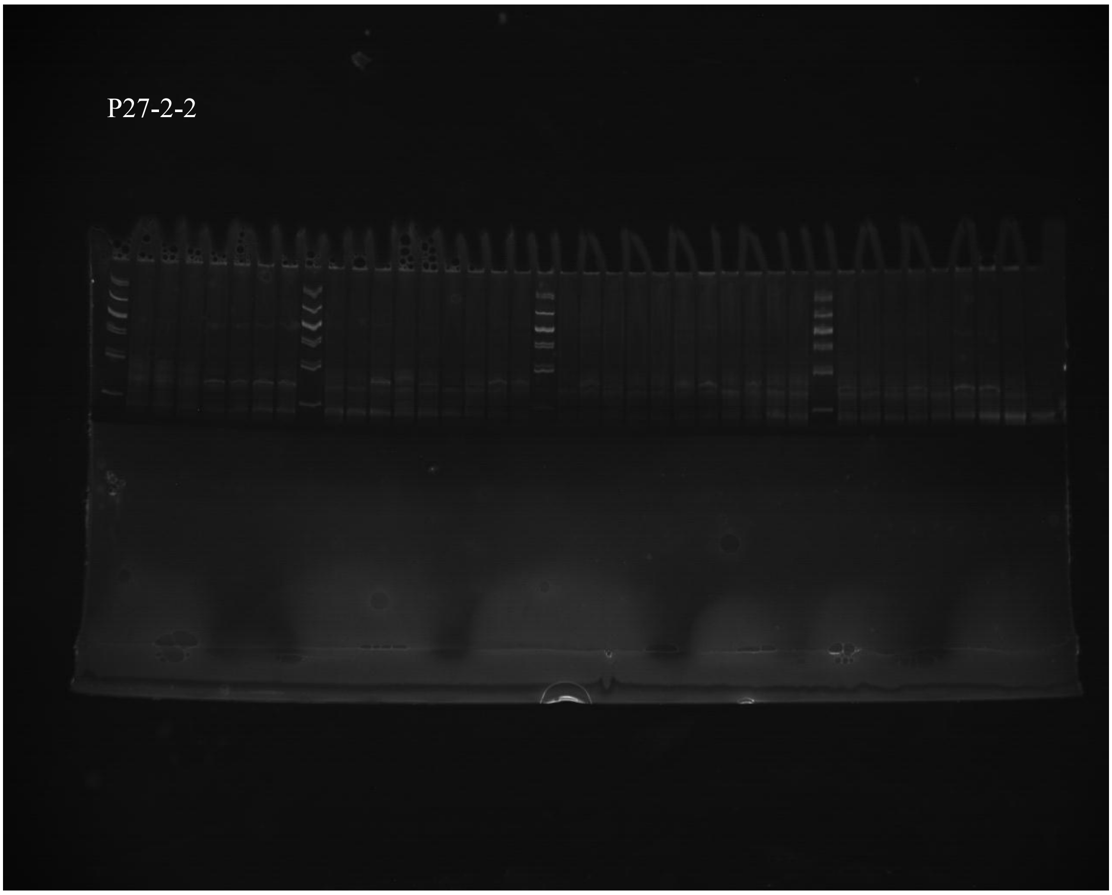
P27-2-2

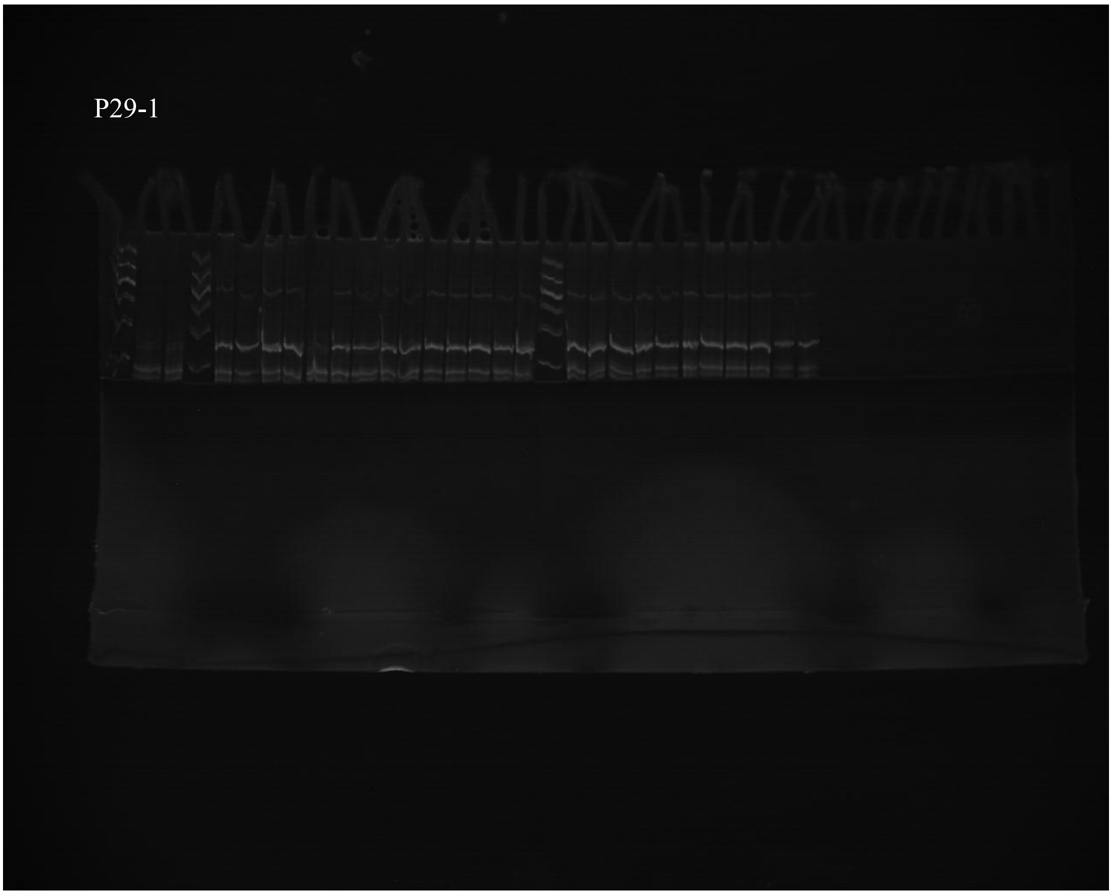
P29-1

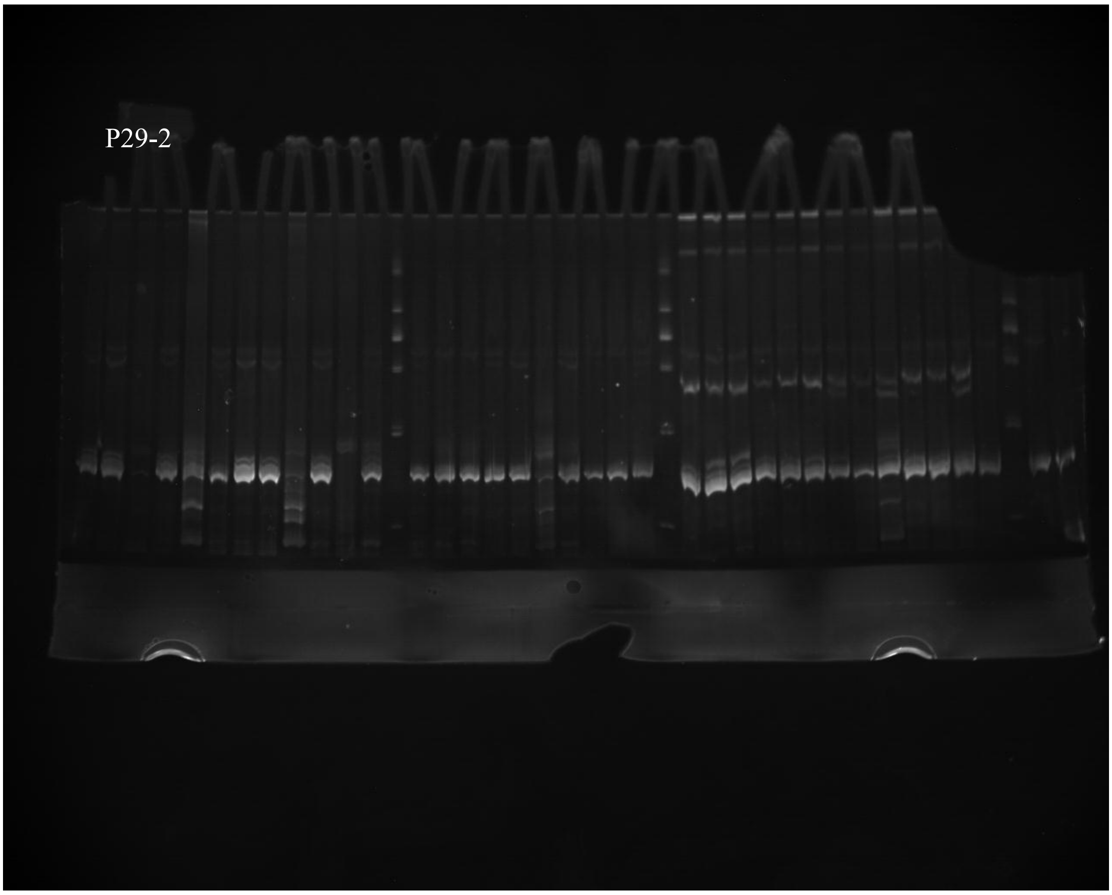
P29-2

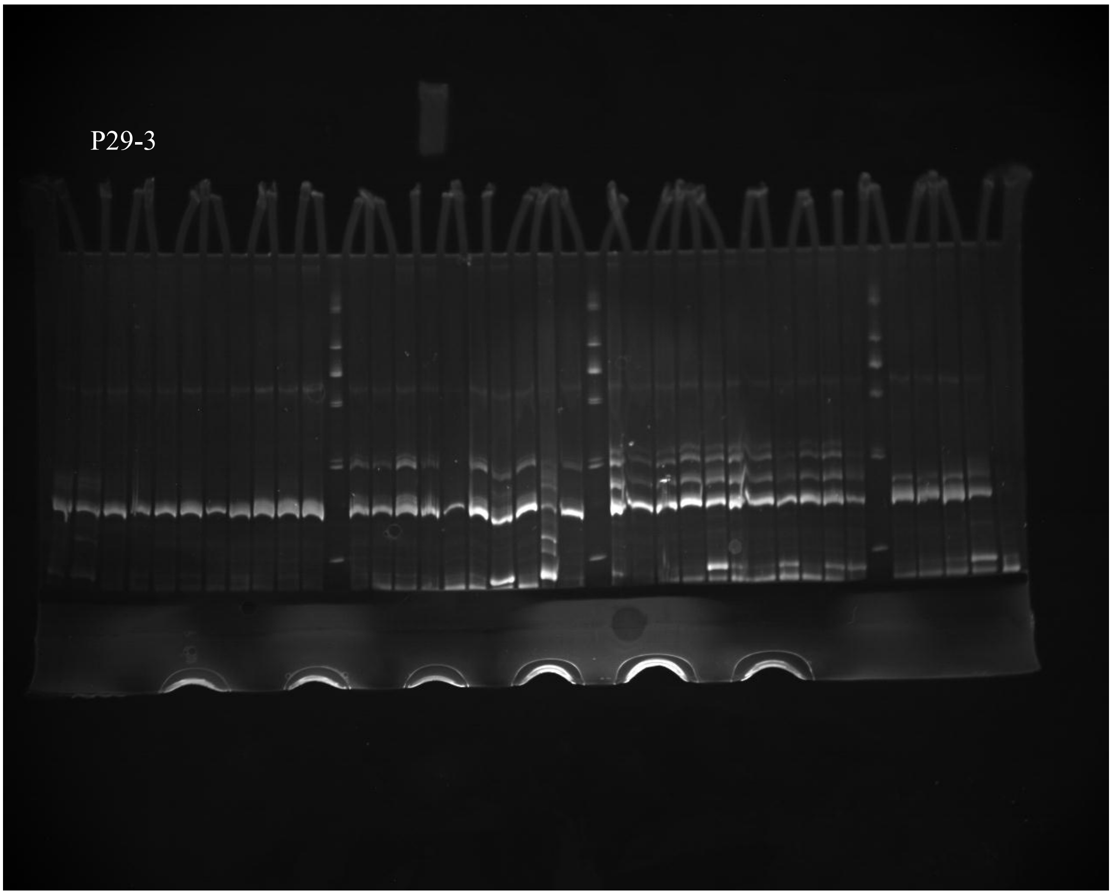
P29-3

P29-4

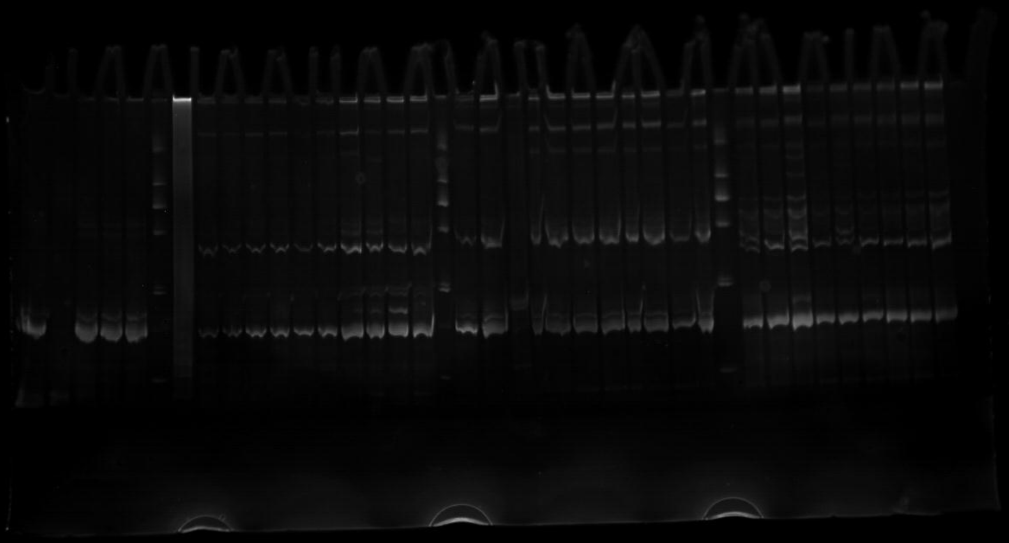

P29-5

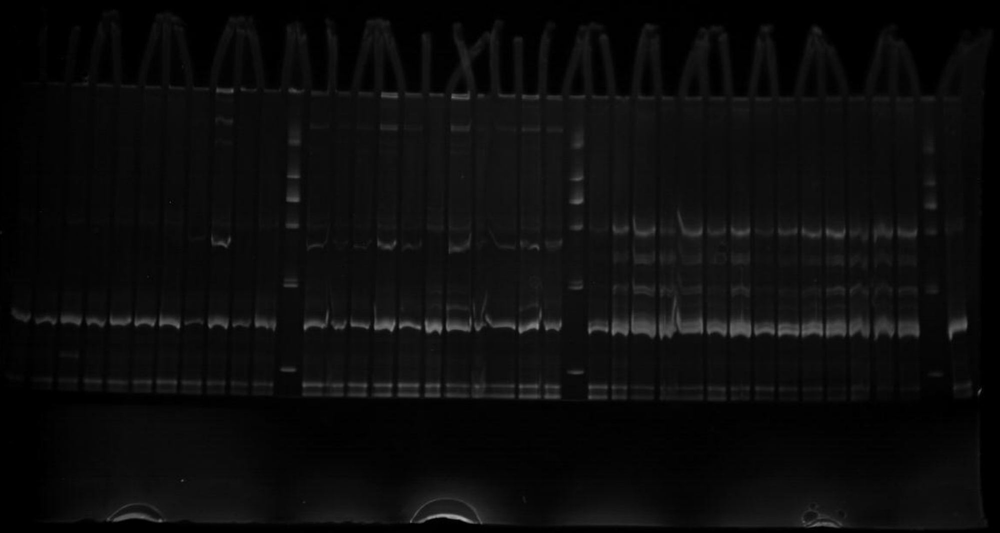

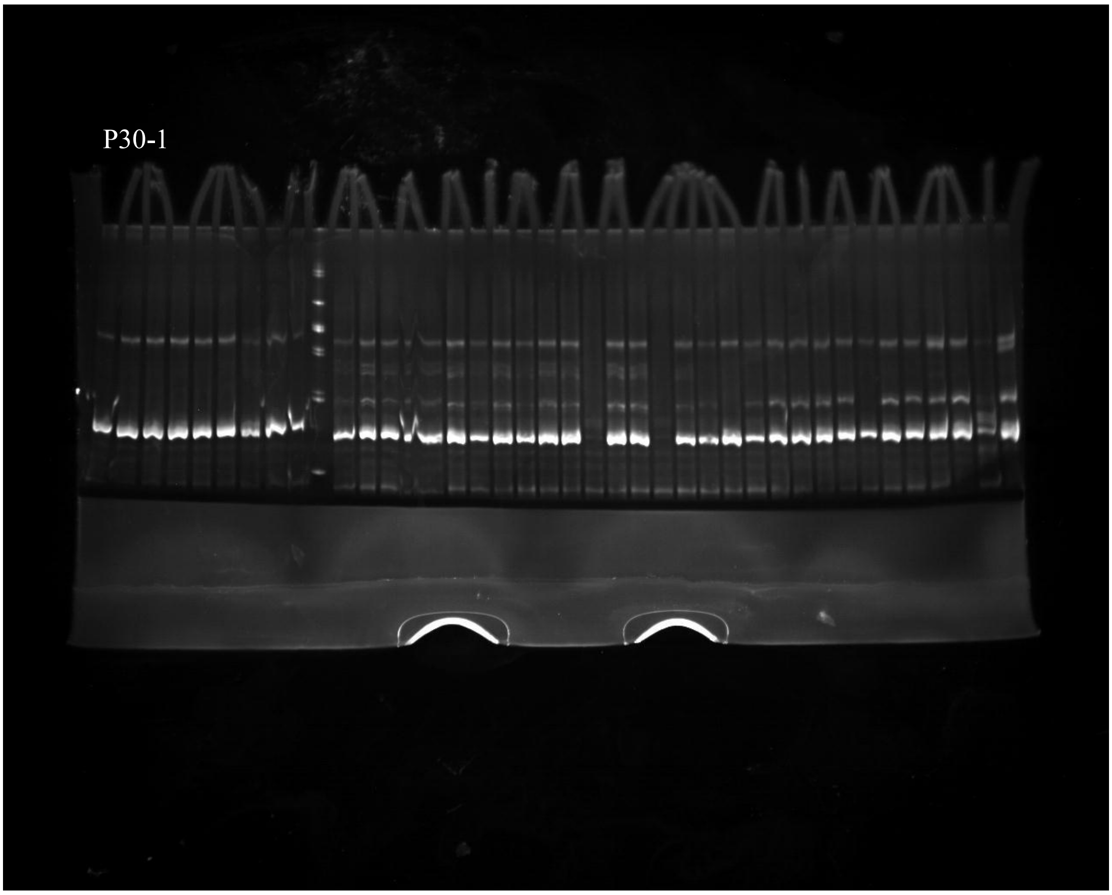
P30-1

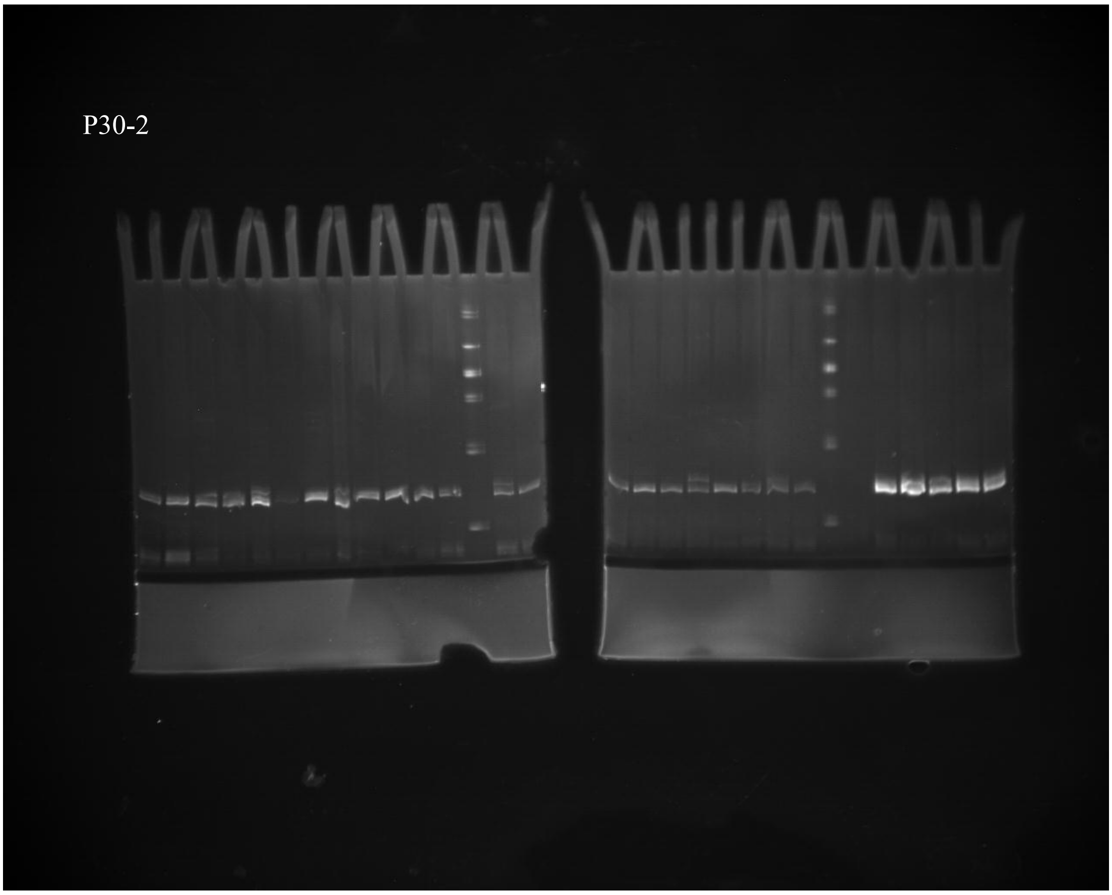
P30-2

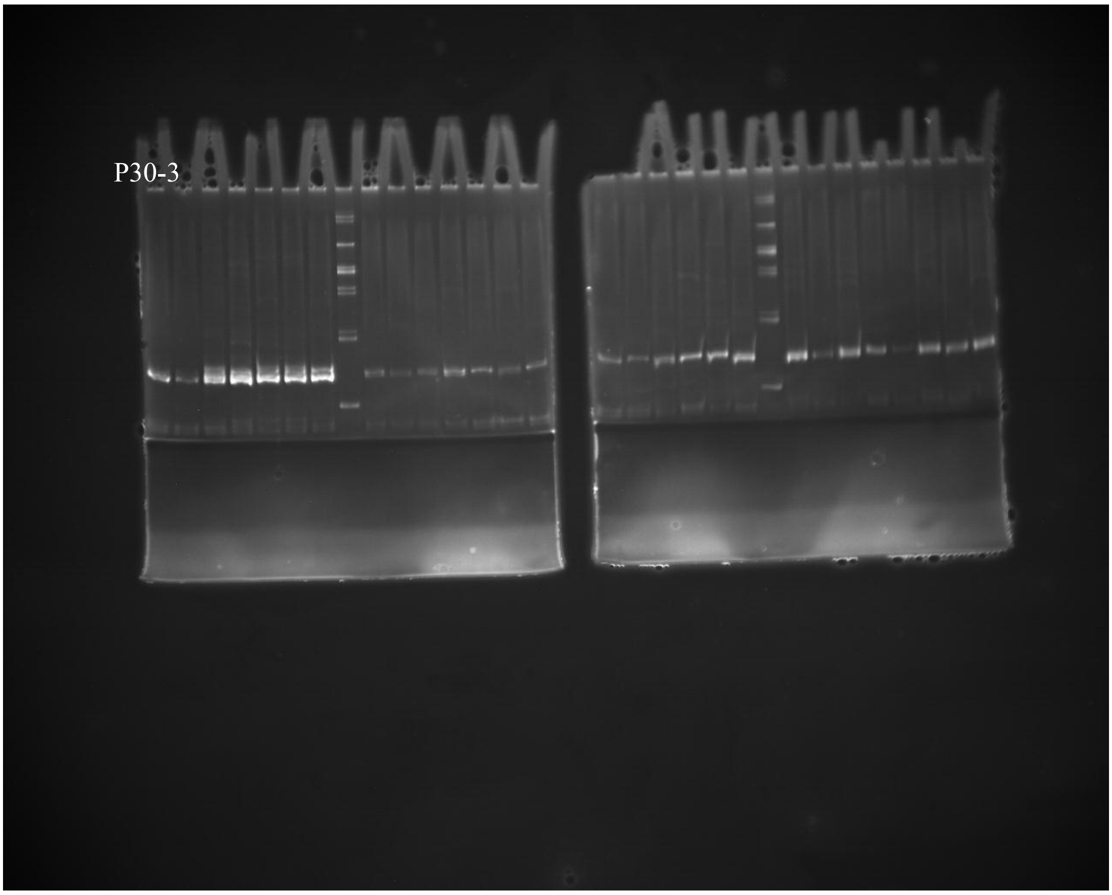
P30-3

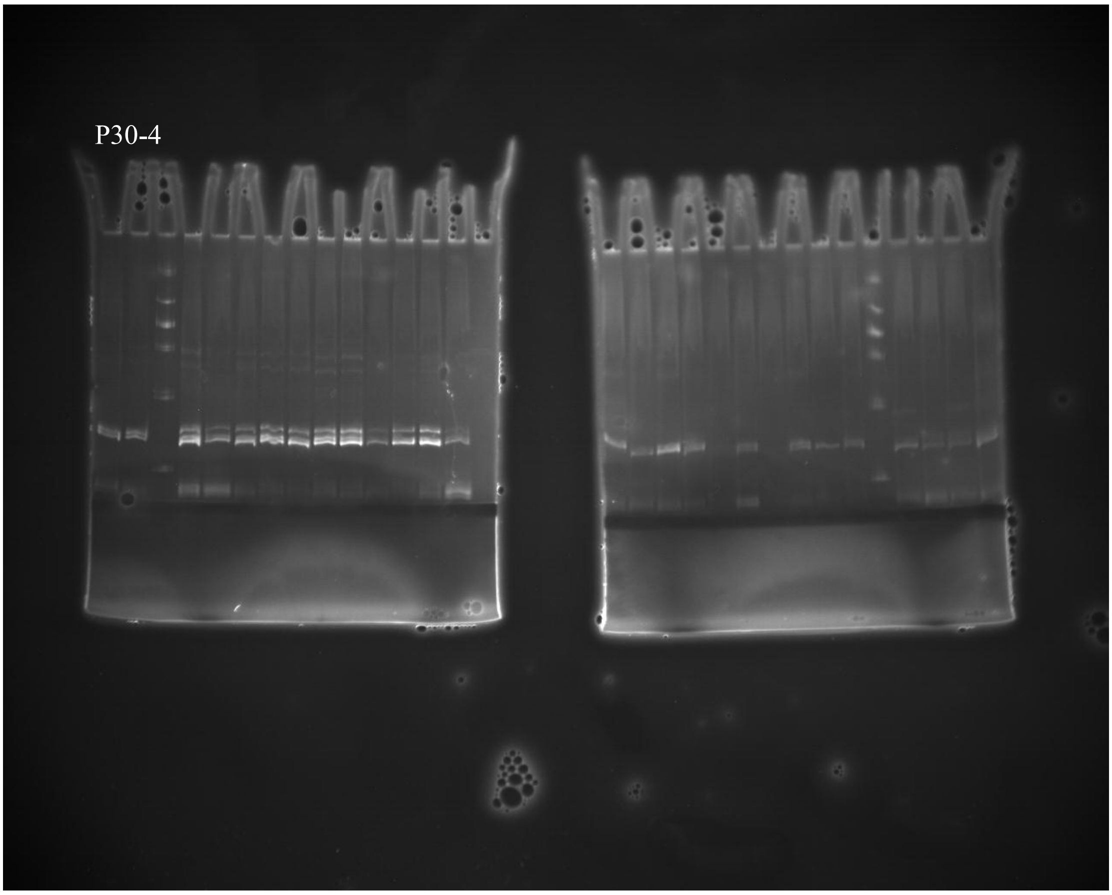
P30-4

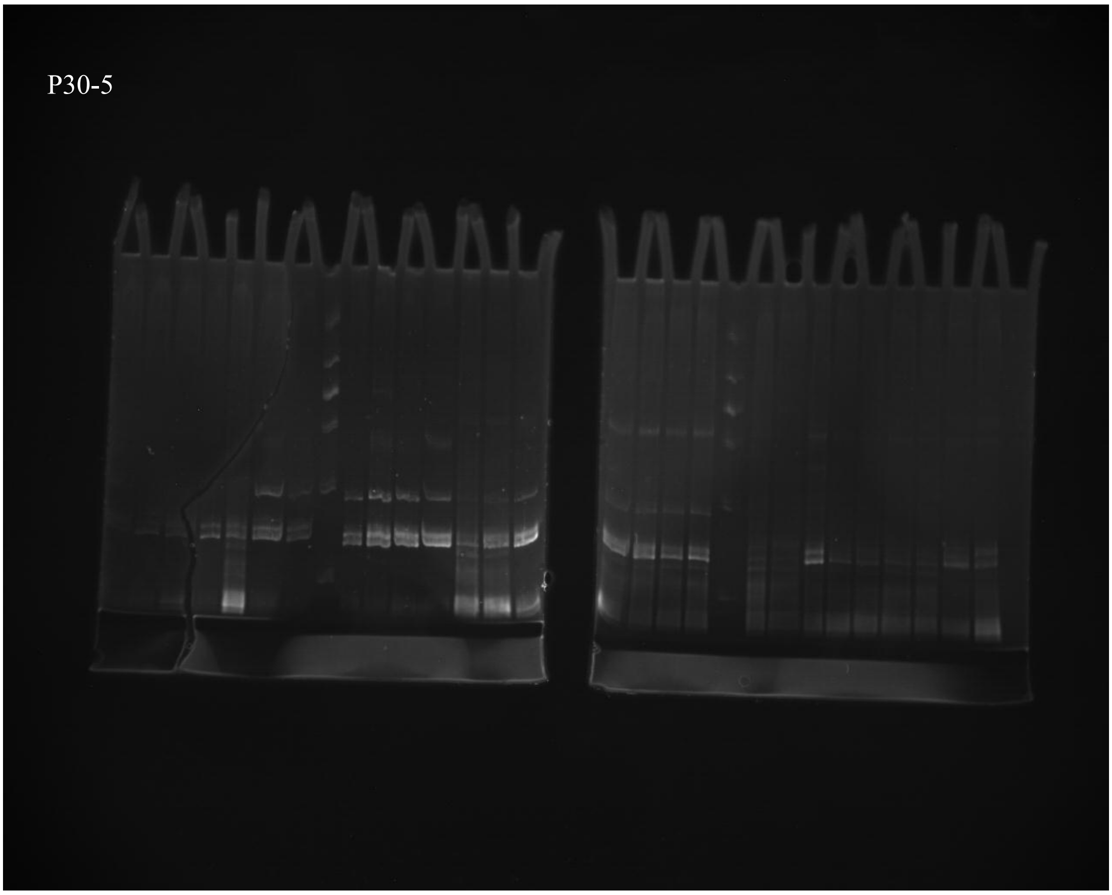
P30-5

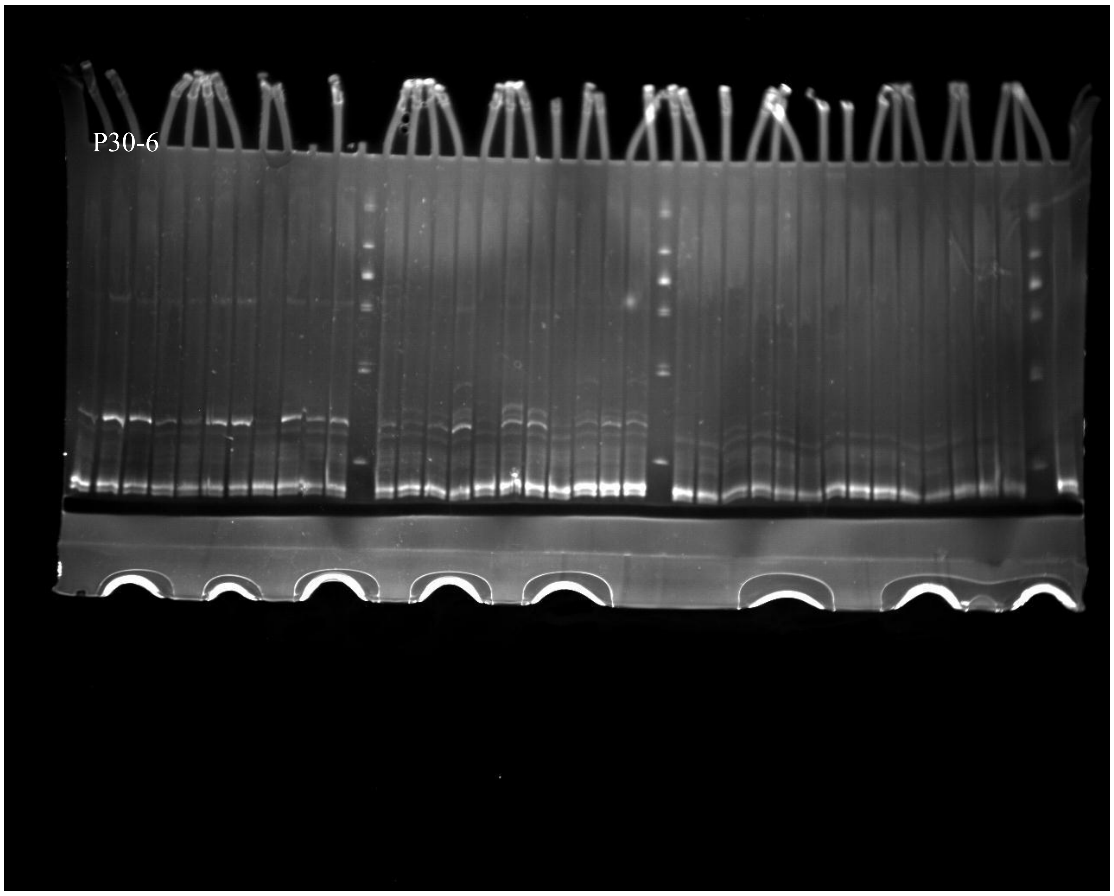
P30-6

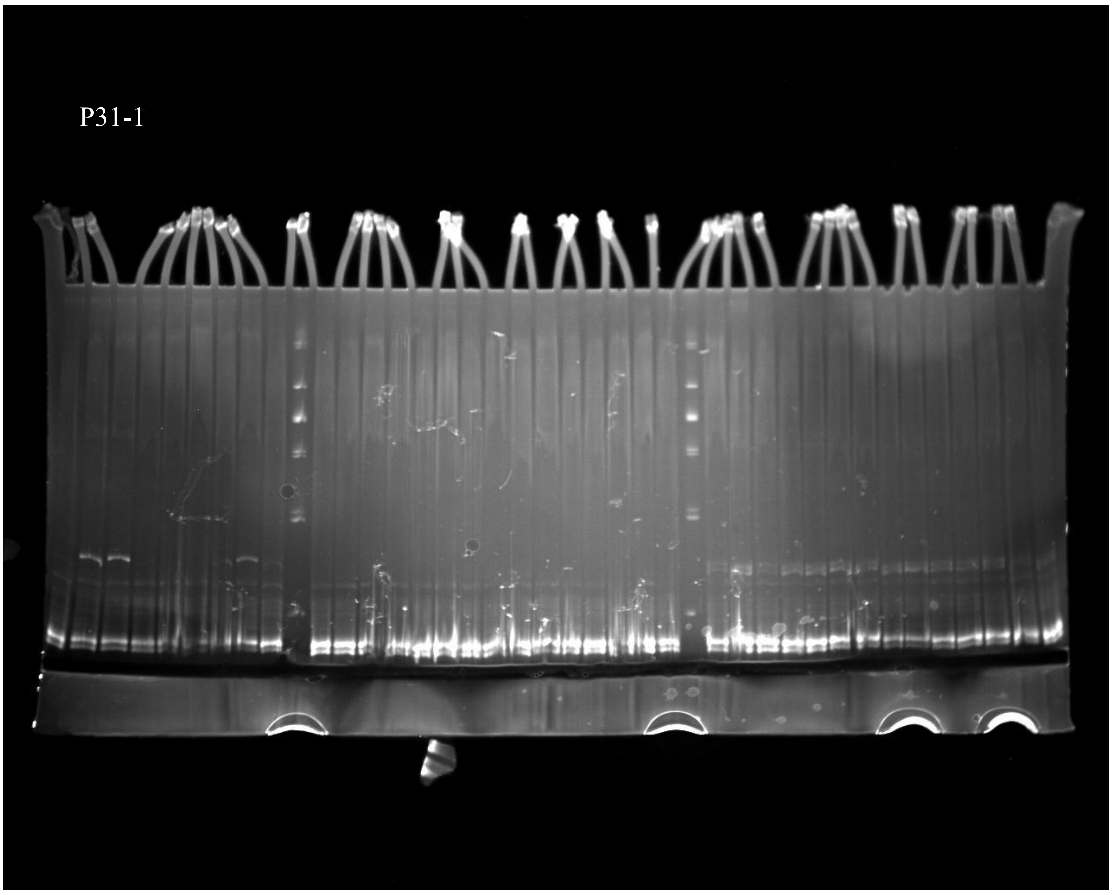
P31-1

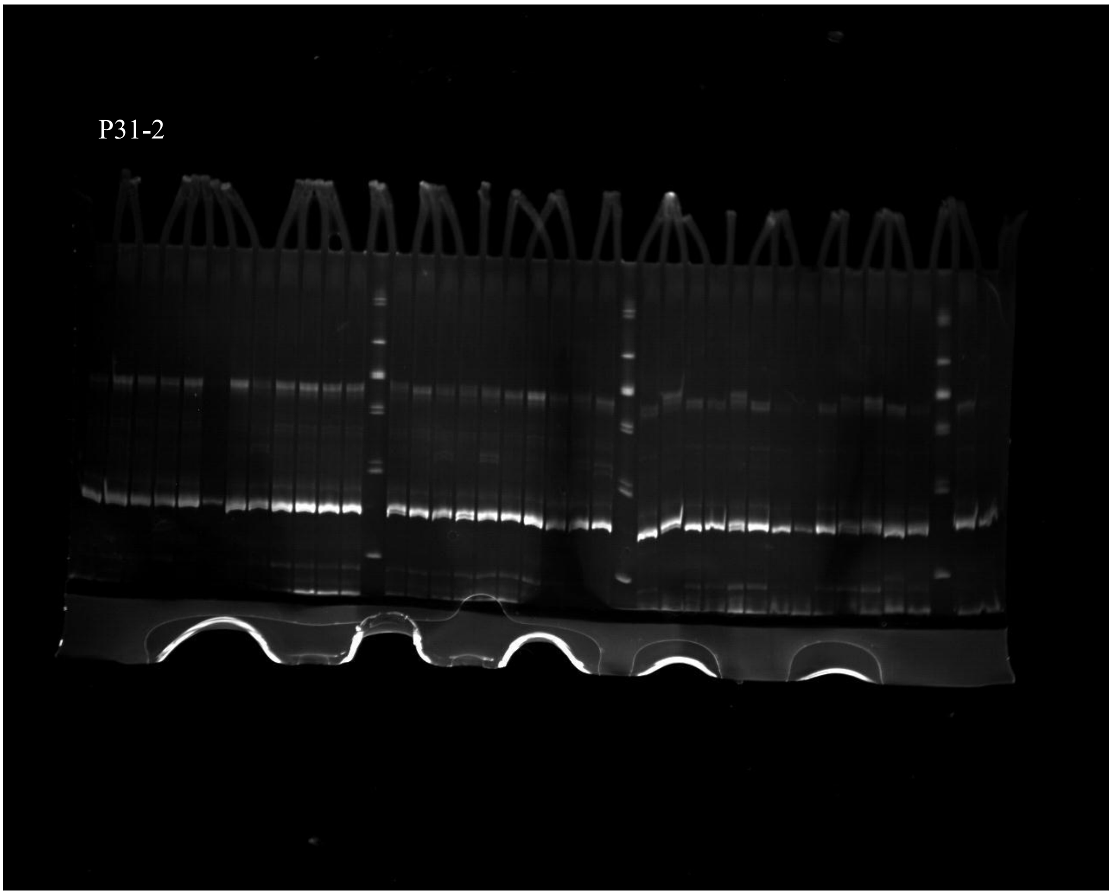
P31-2

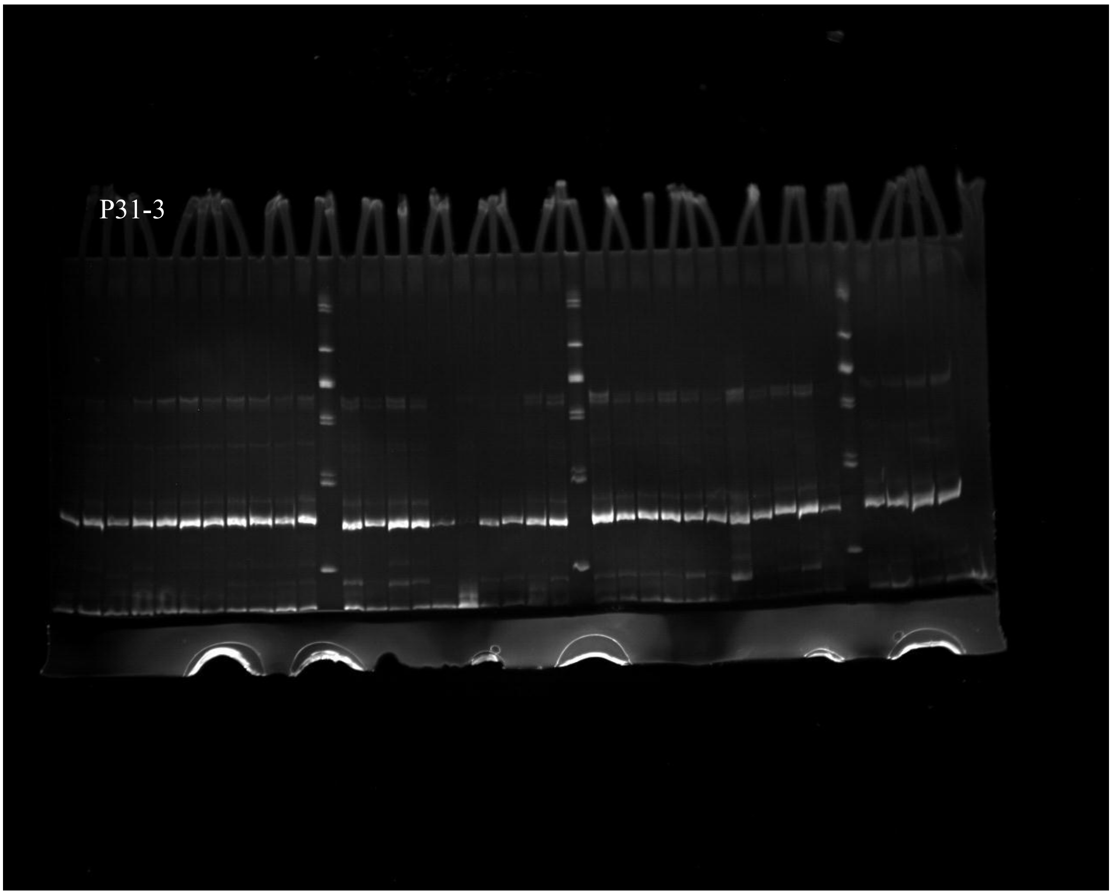
P31-3

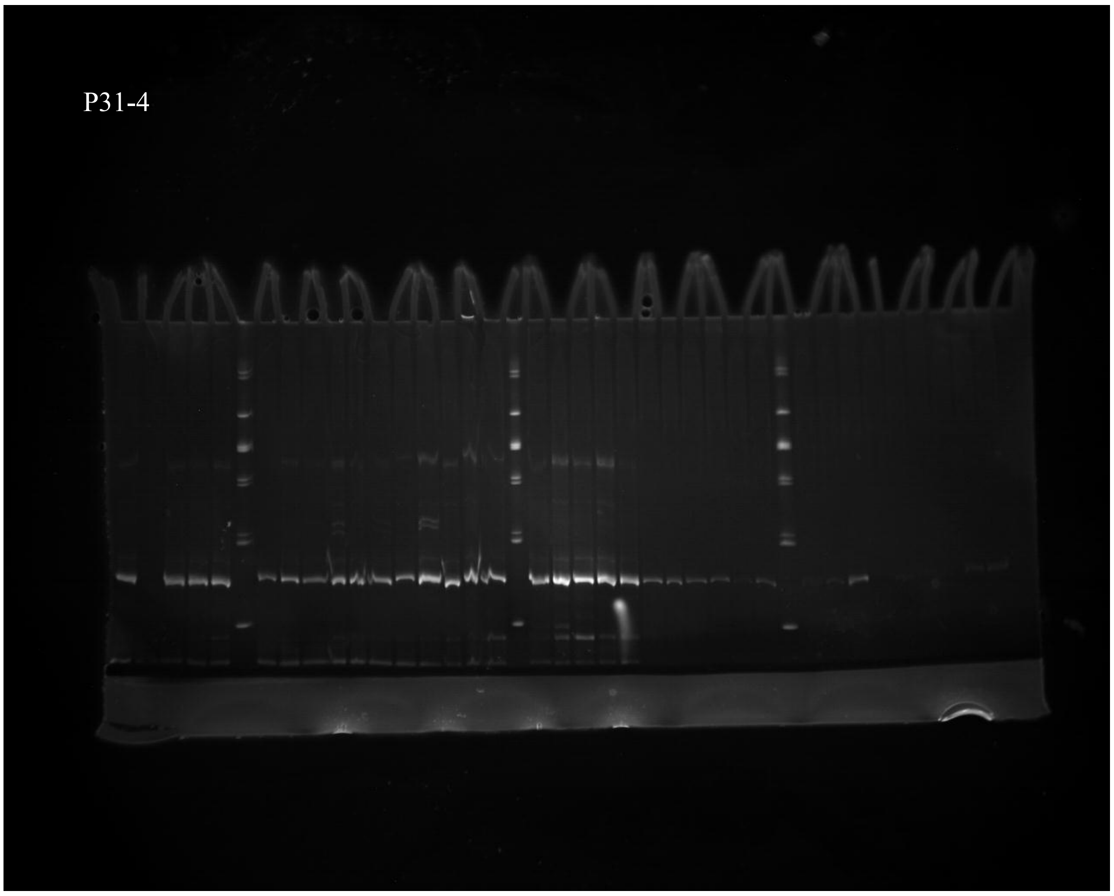
P31-4

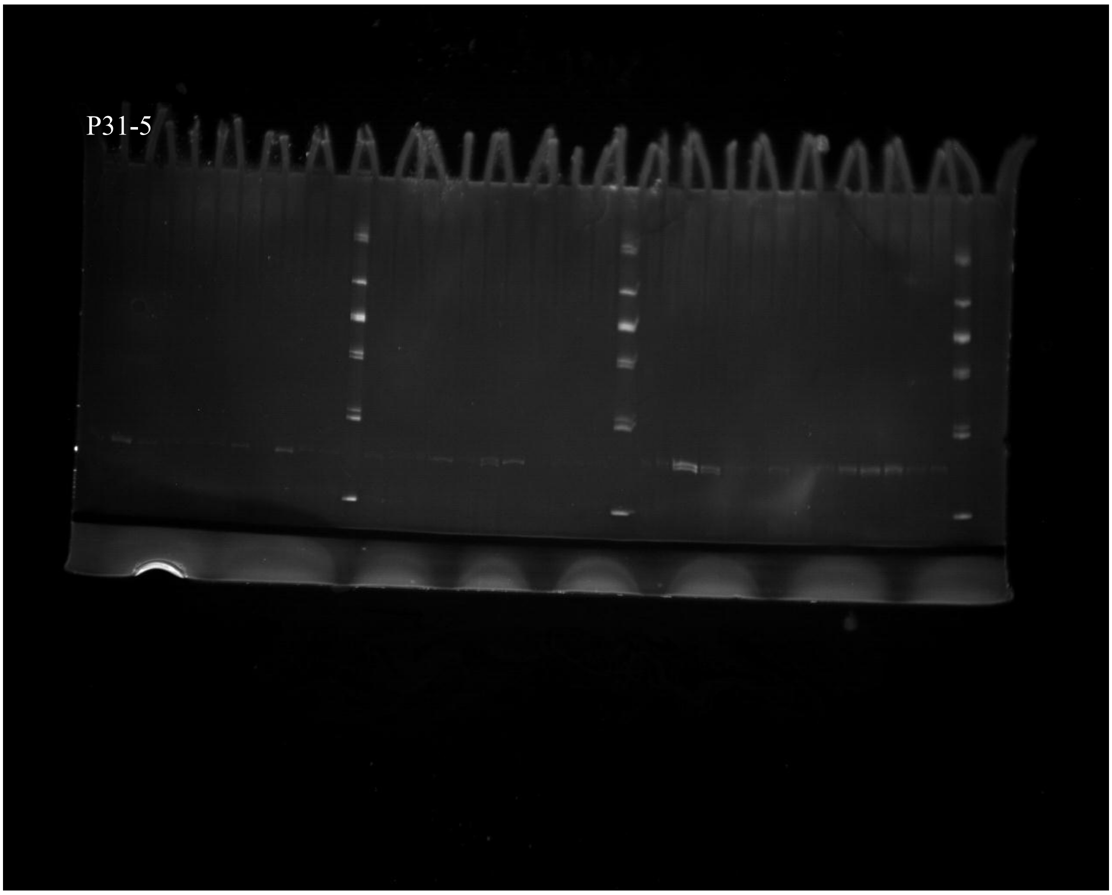
P31-5

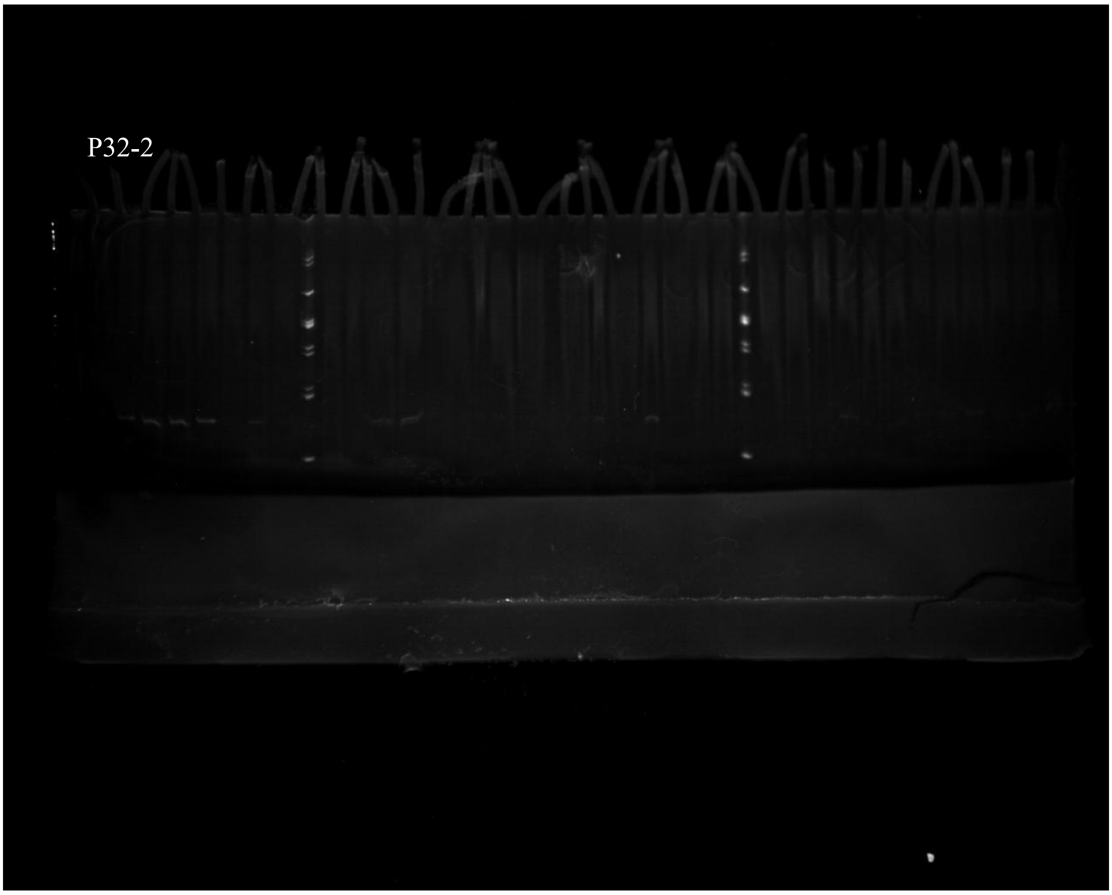
P32-2

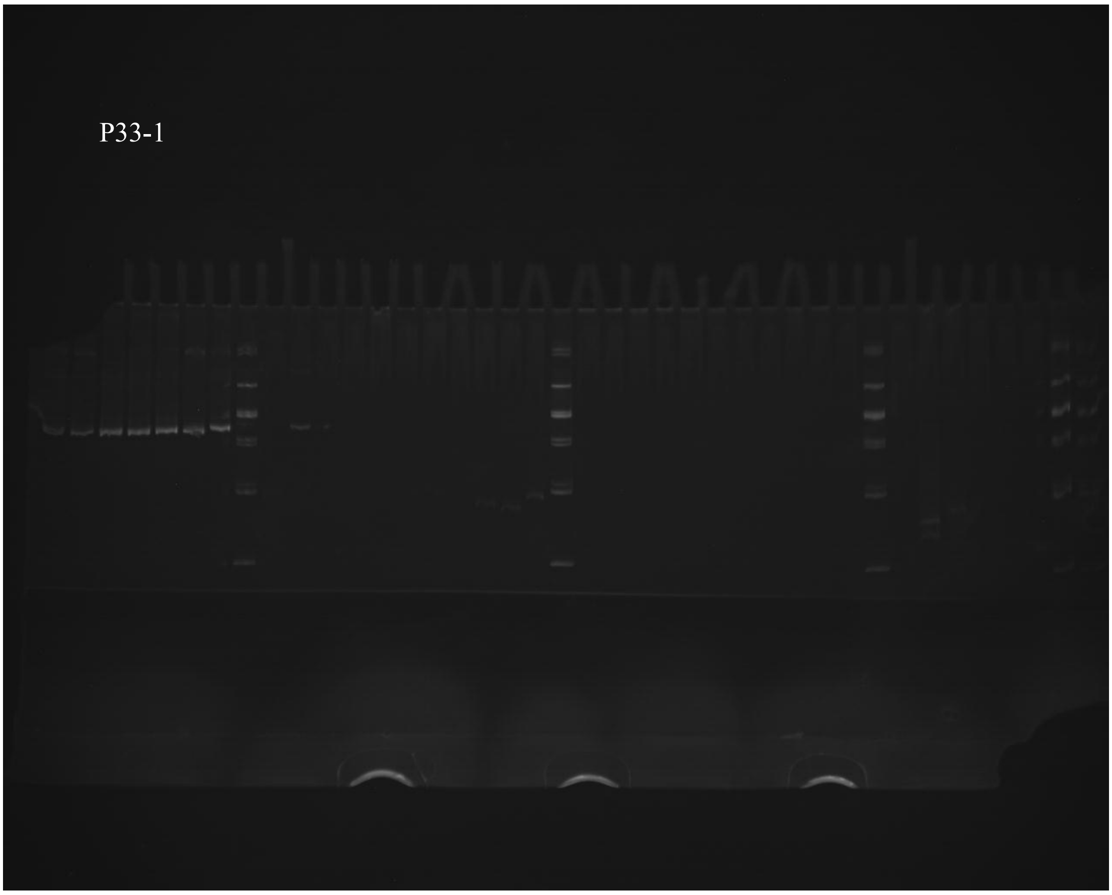
P33-1

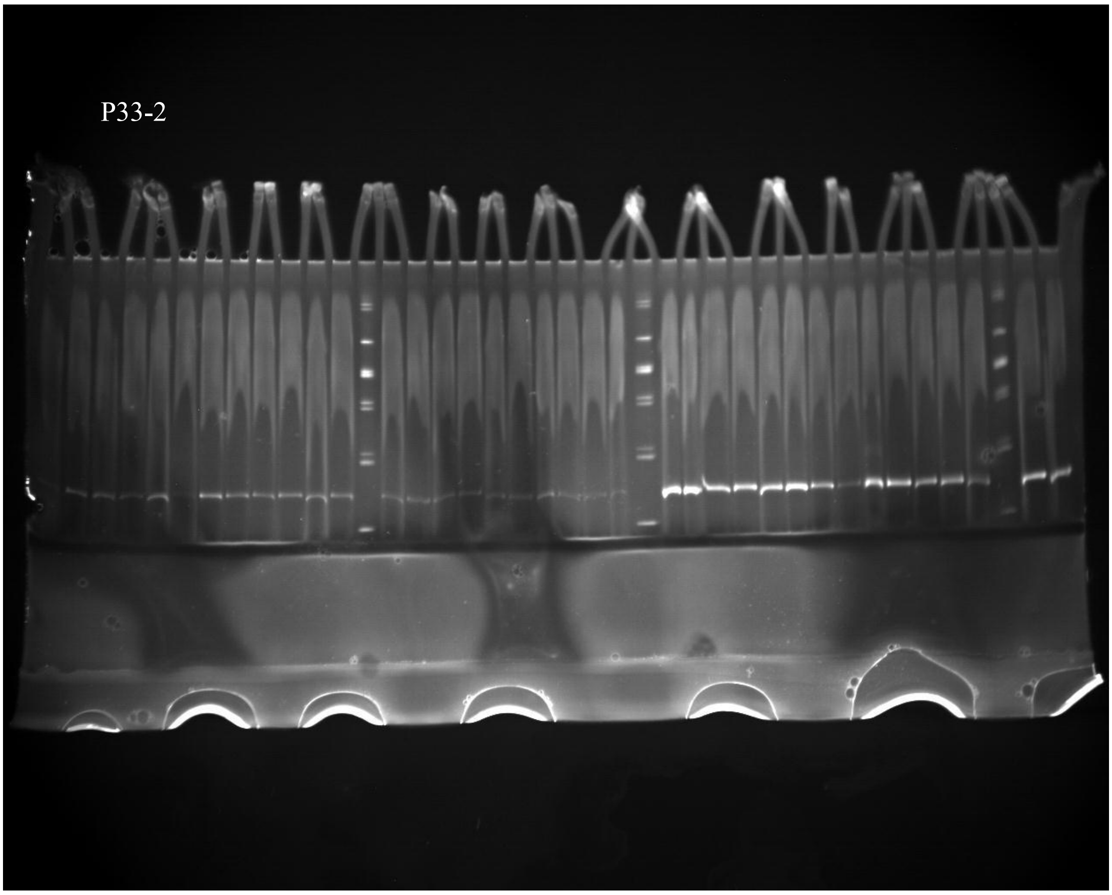
P33-2

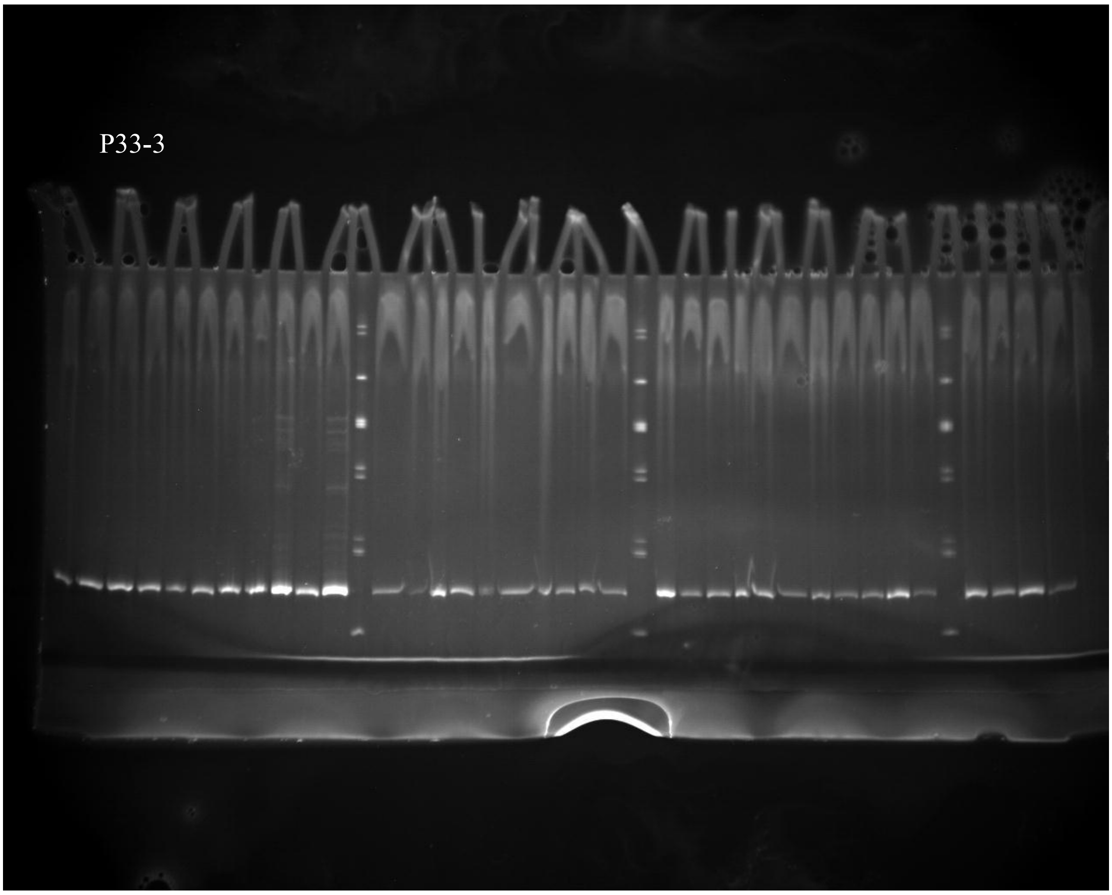
P33-3

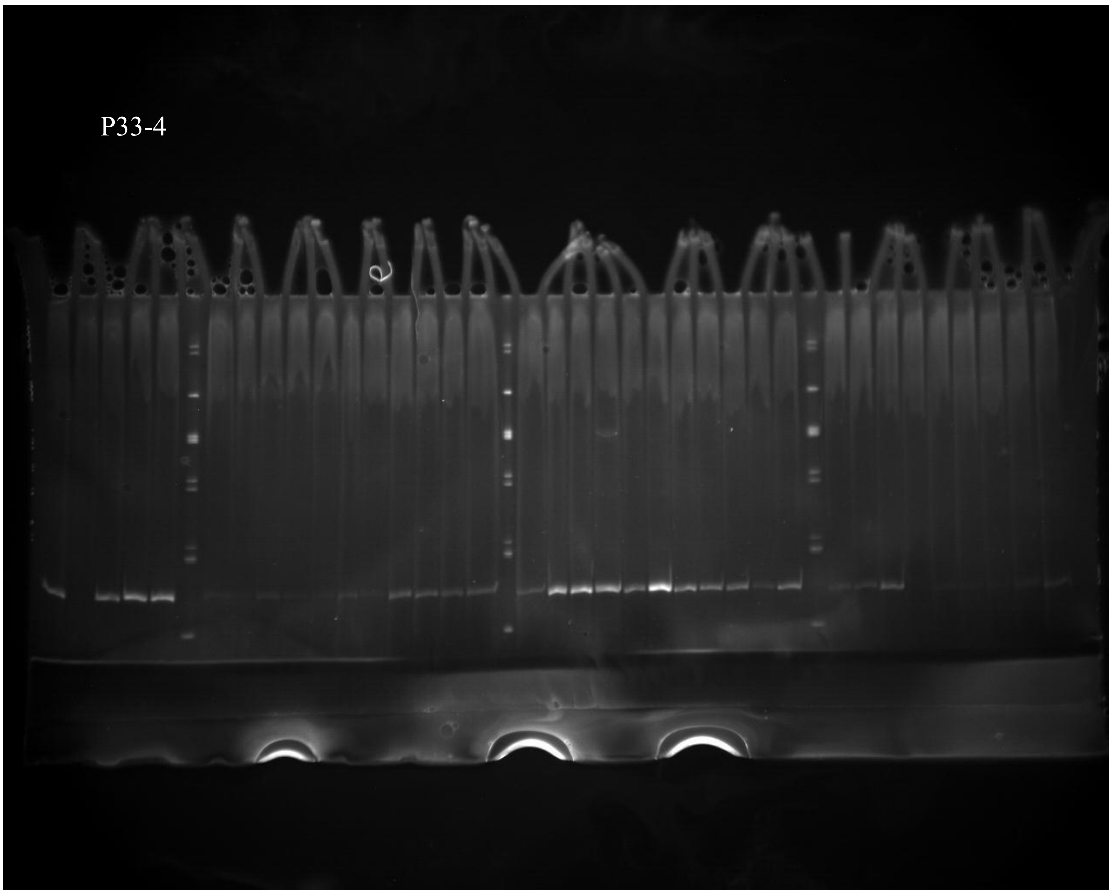
P33-4

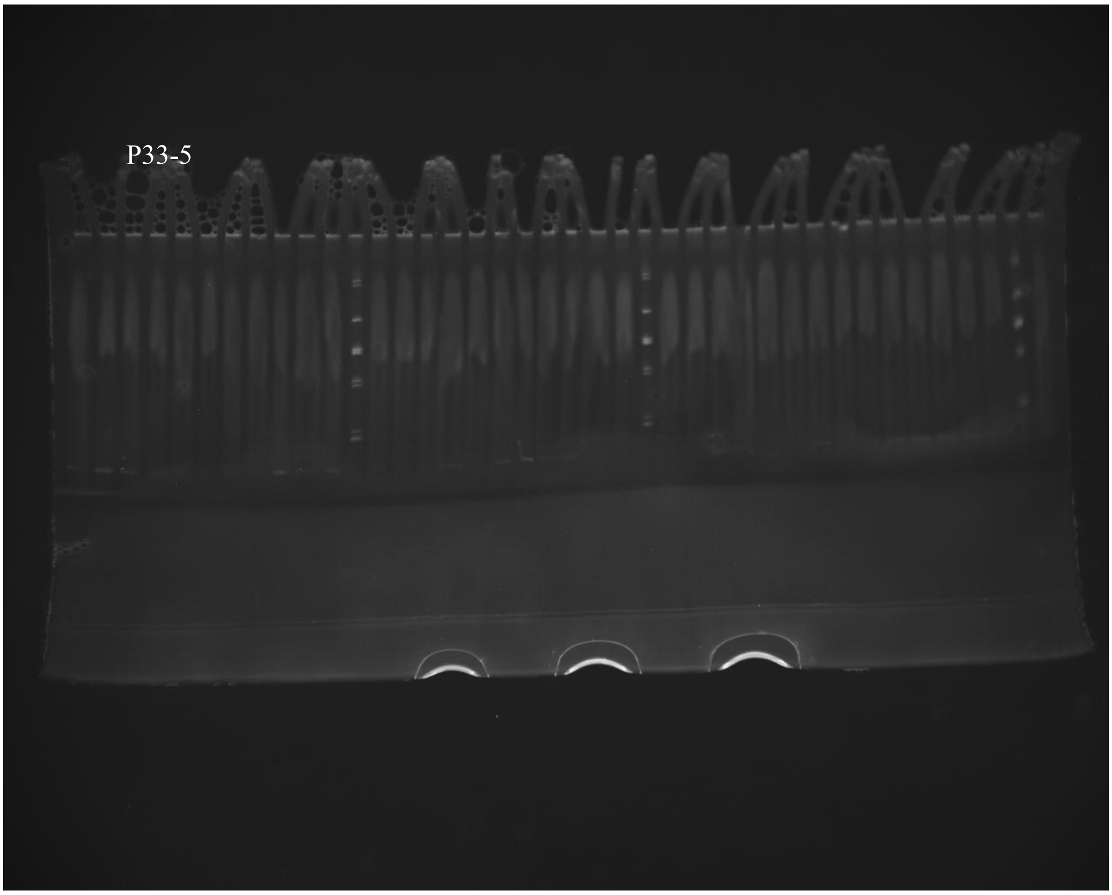
P33-5

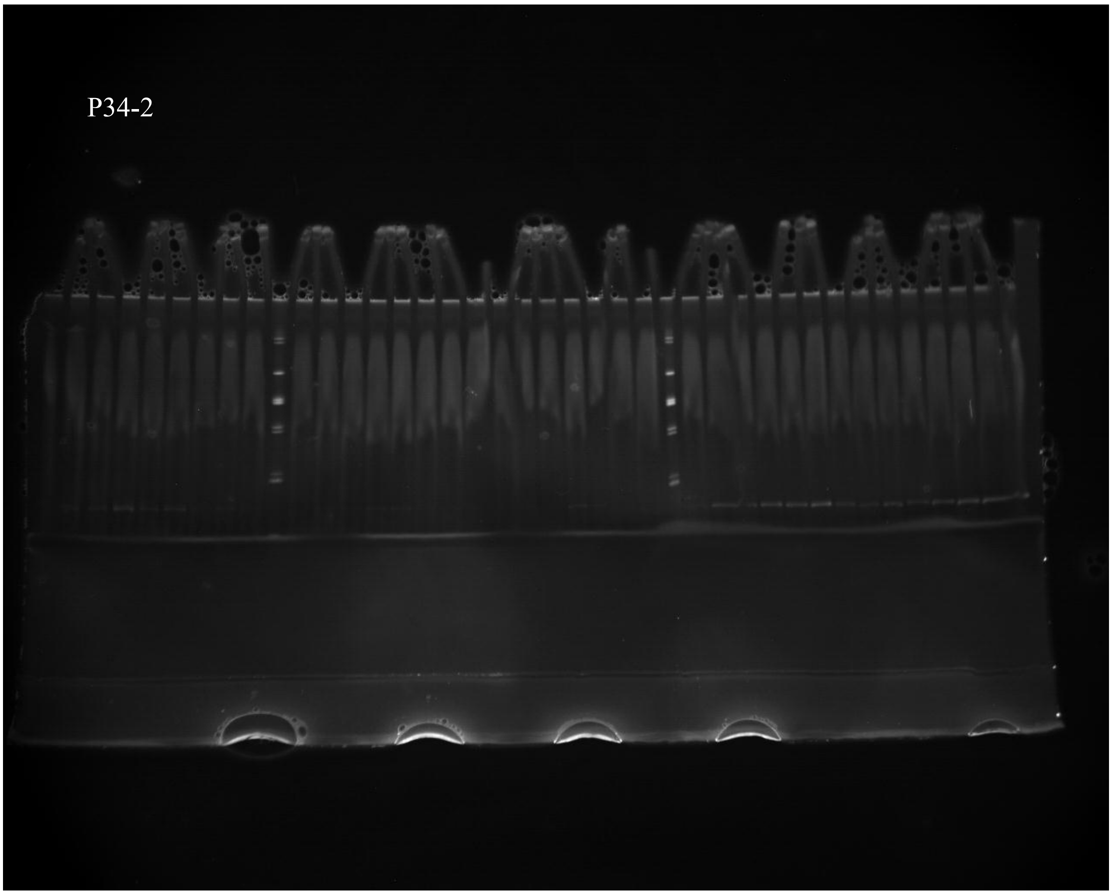
P34-2

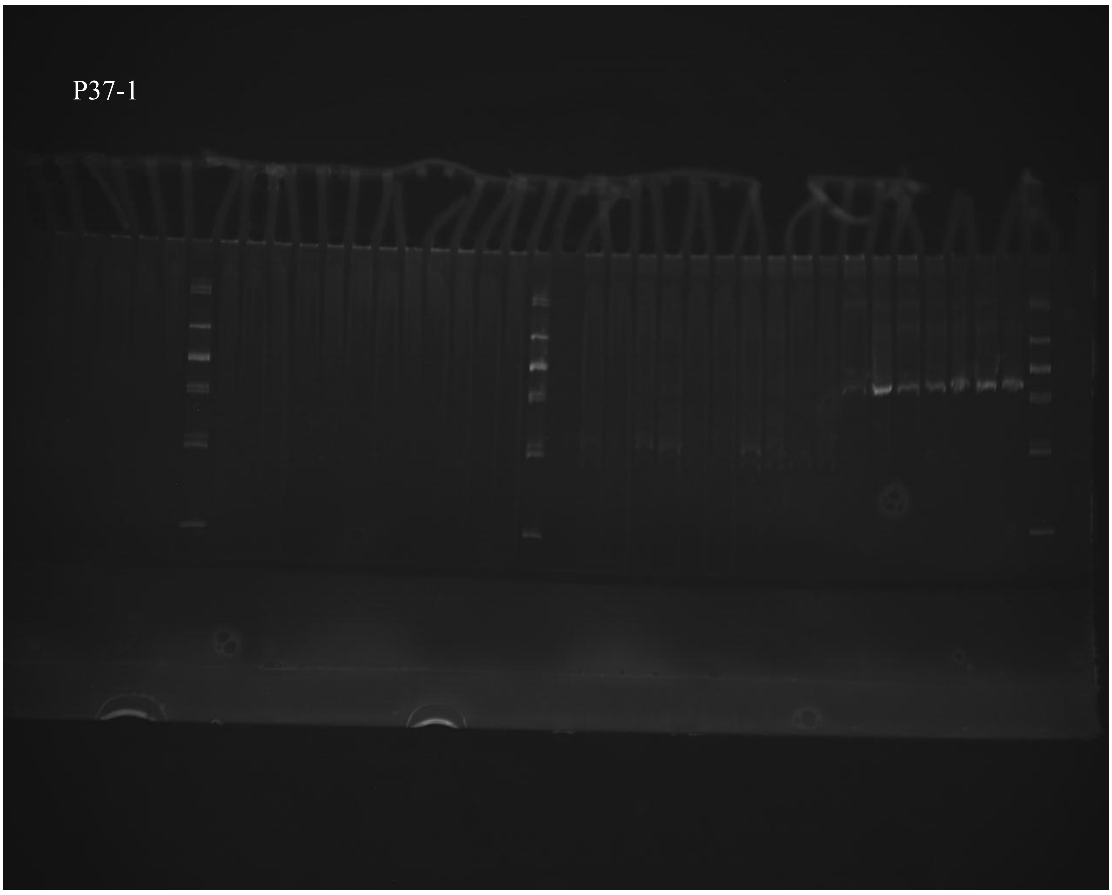
P37-1

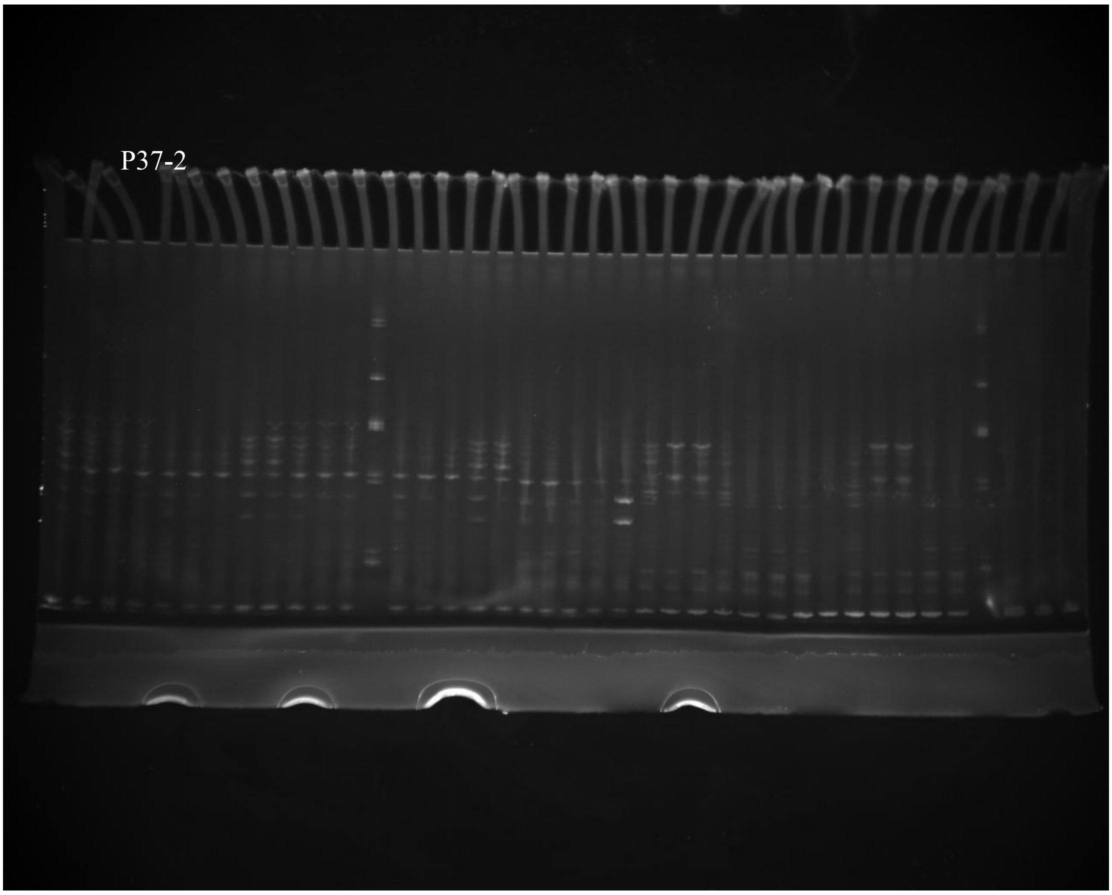
P37-2

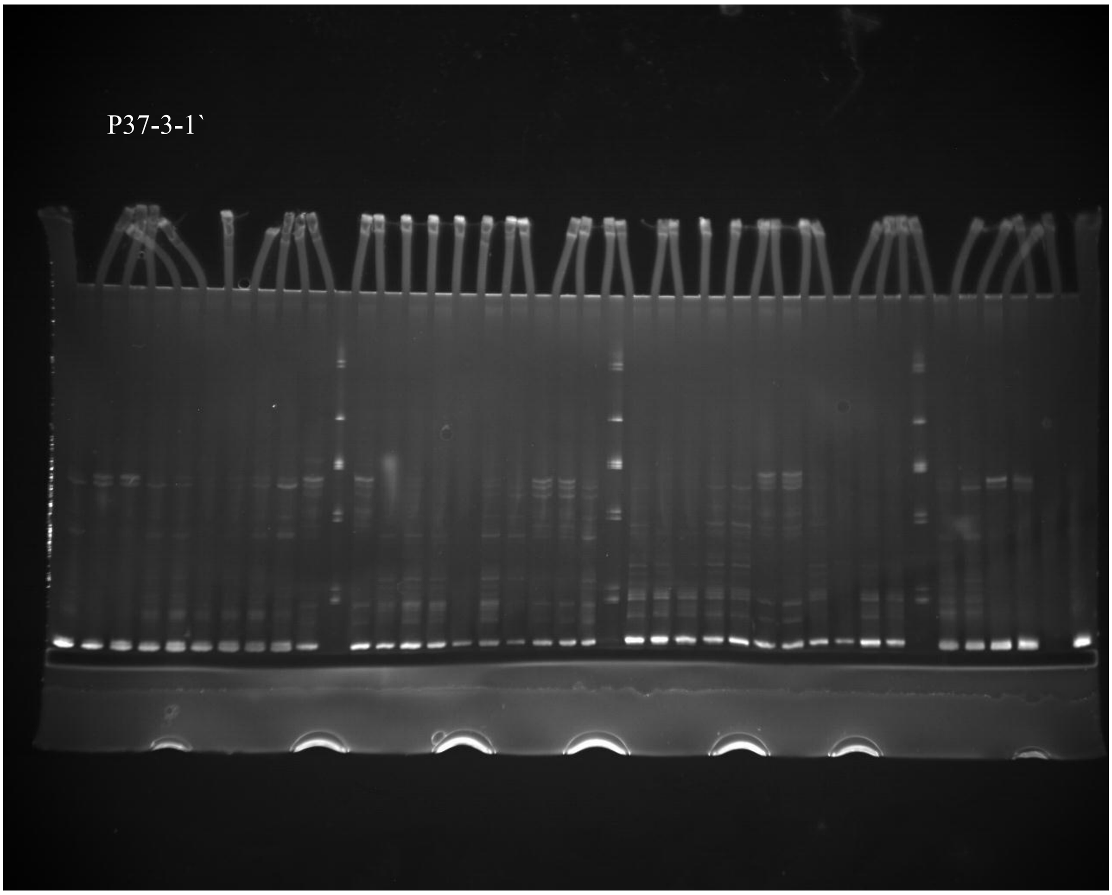
P37-3-1`

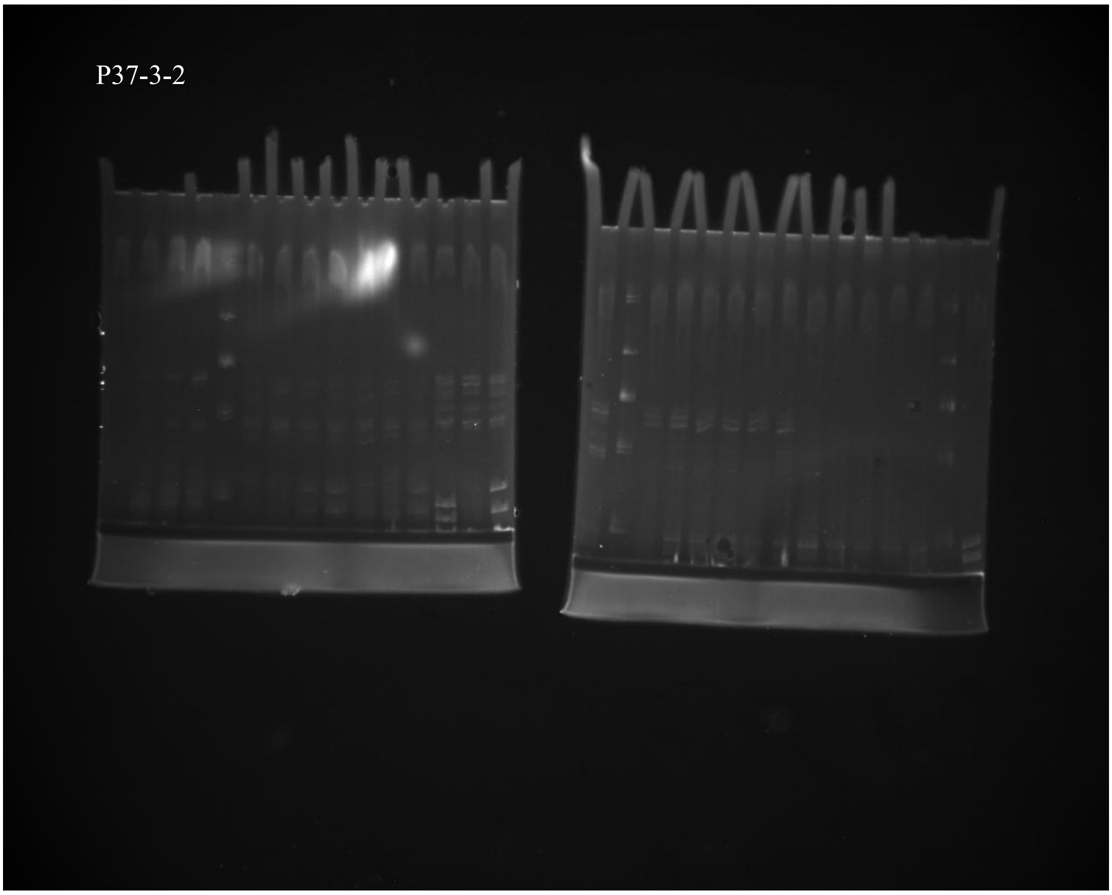
P37-3-2

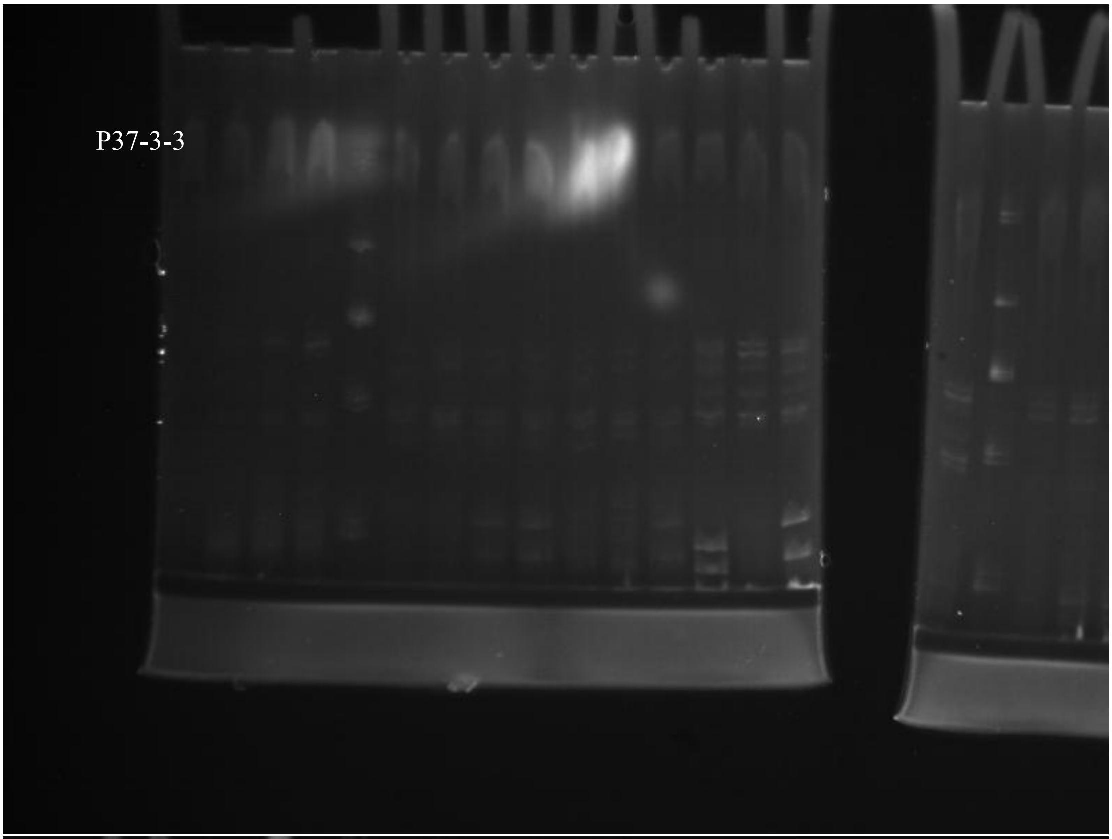
P37-3-3

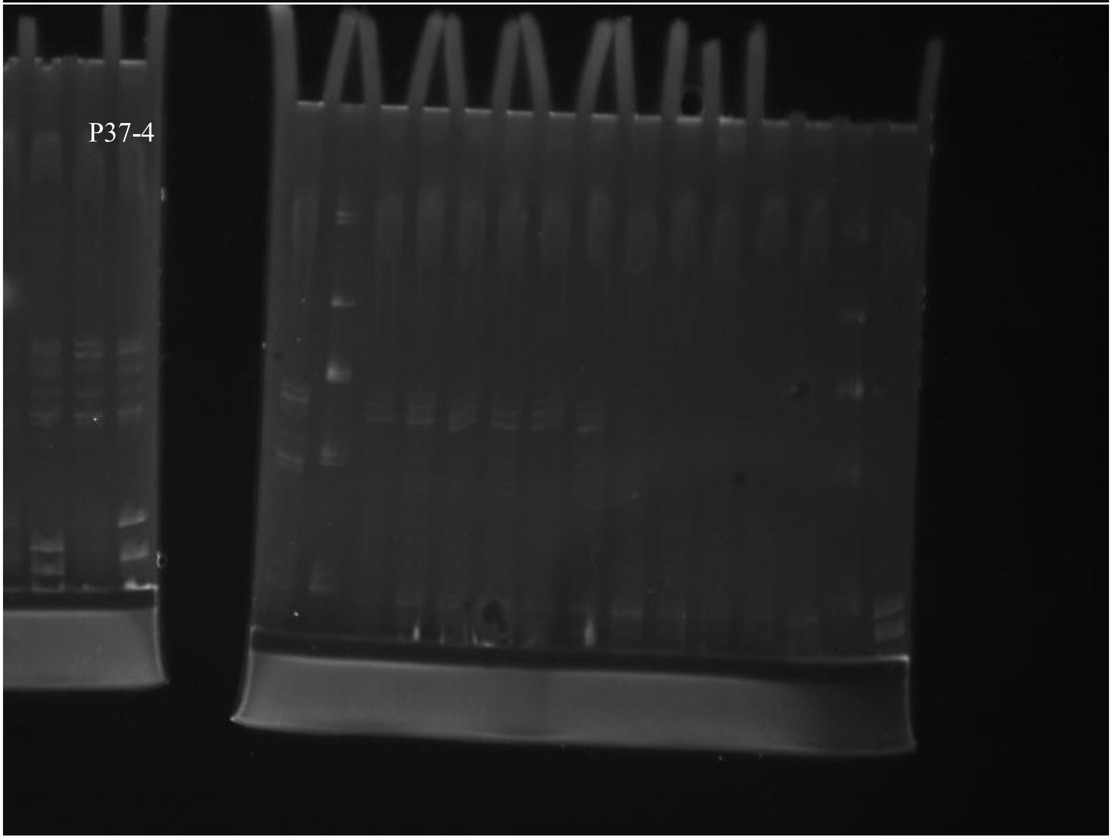
P37-4

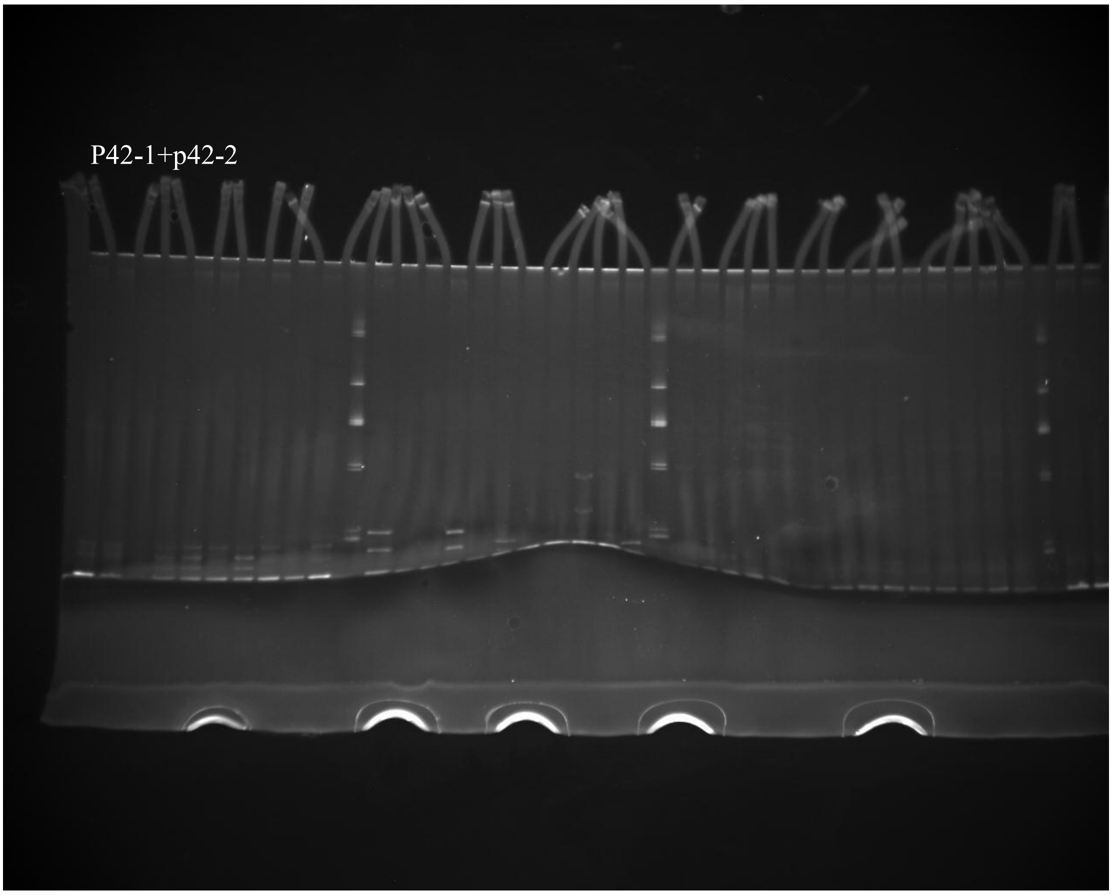
P42-1+p42-2

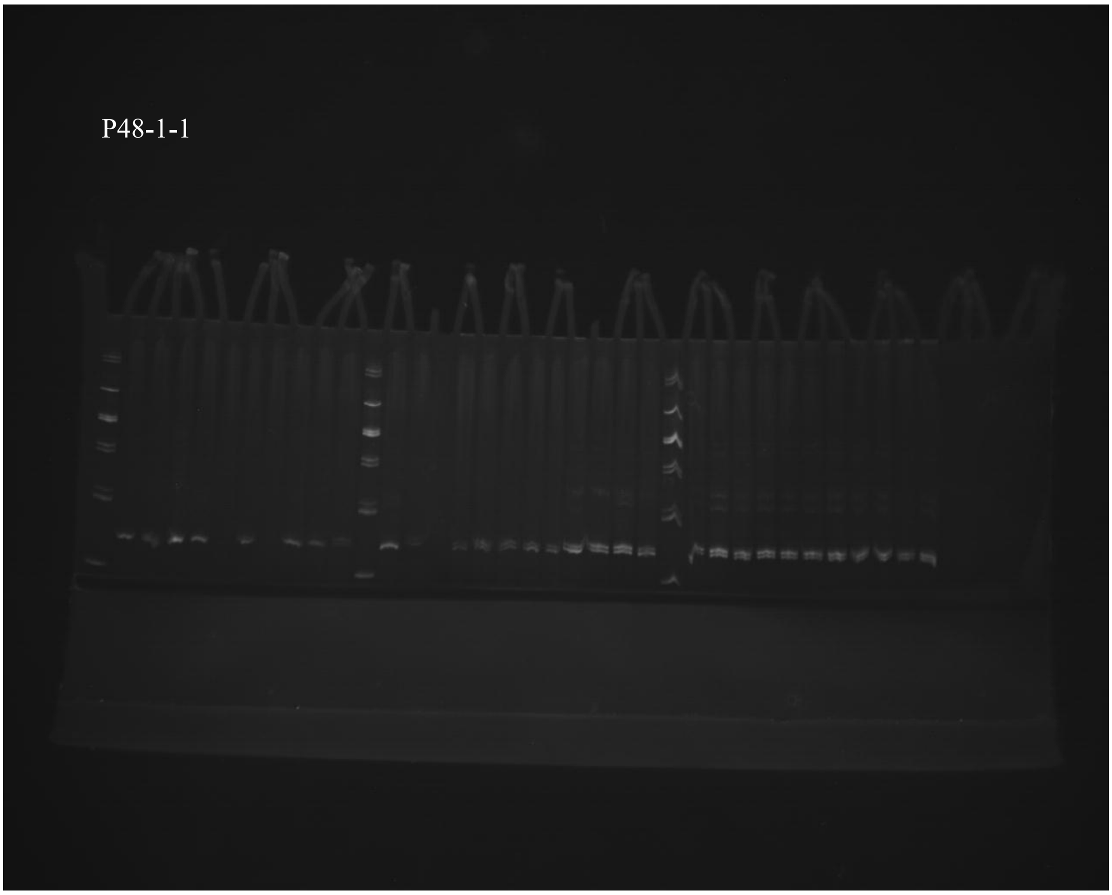
P48-1-1

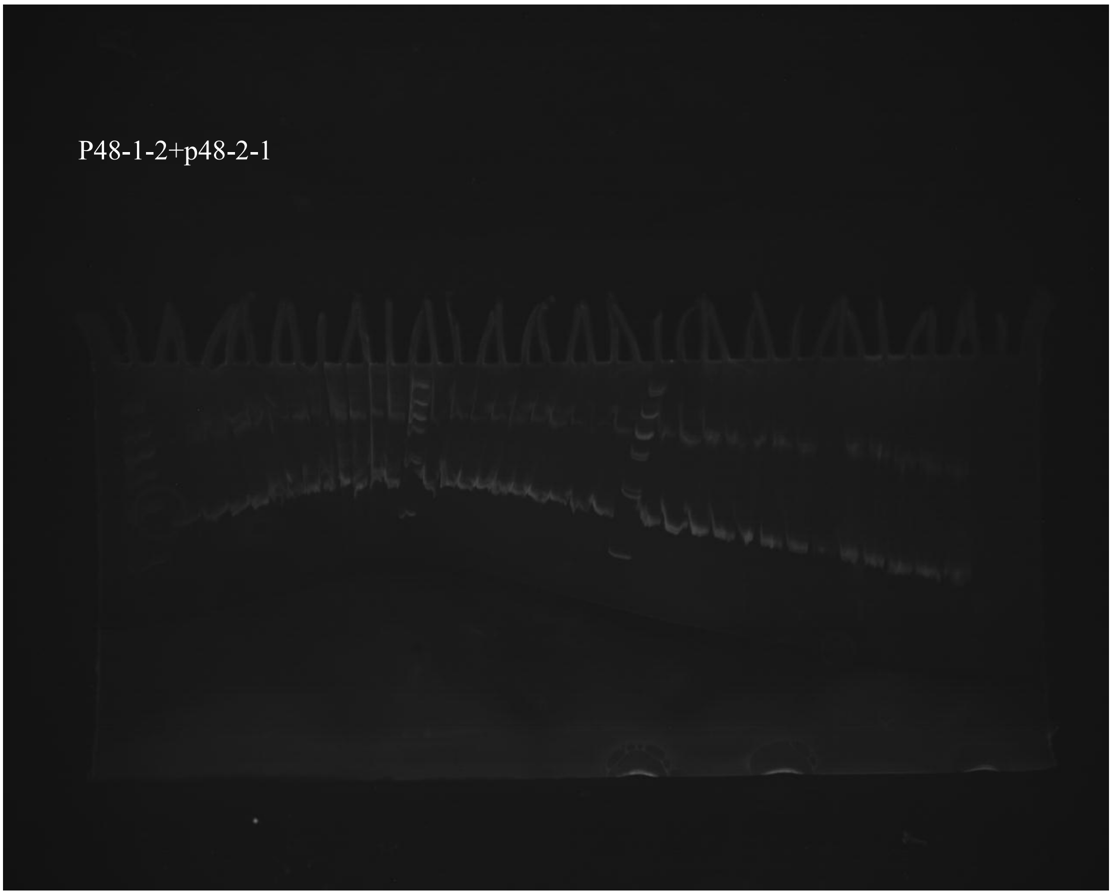
P48-1-2+p48-2-1

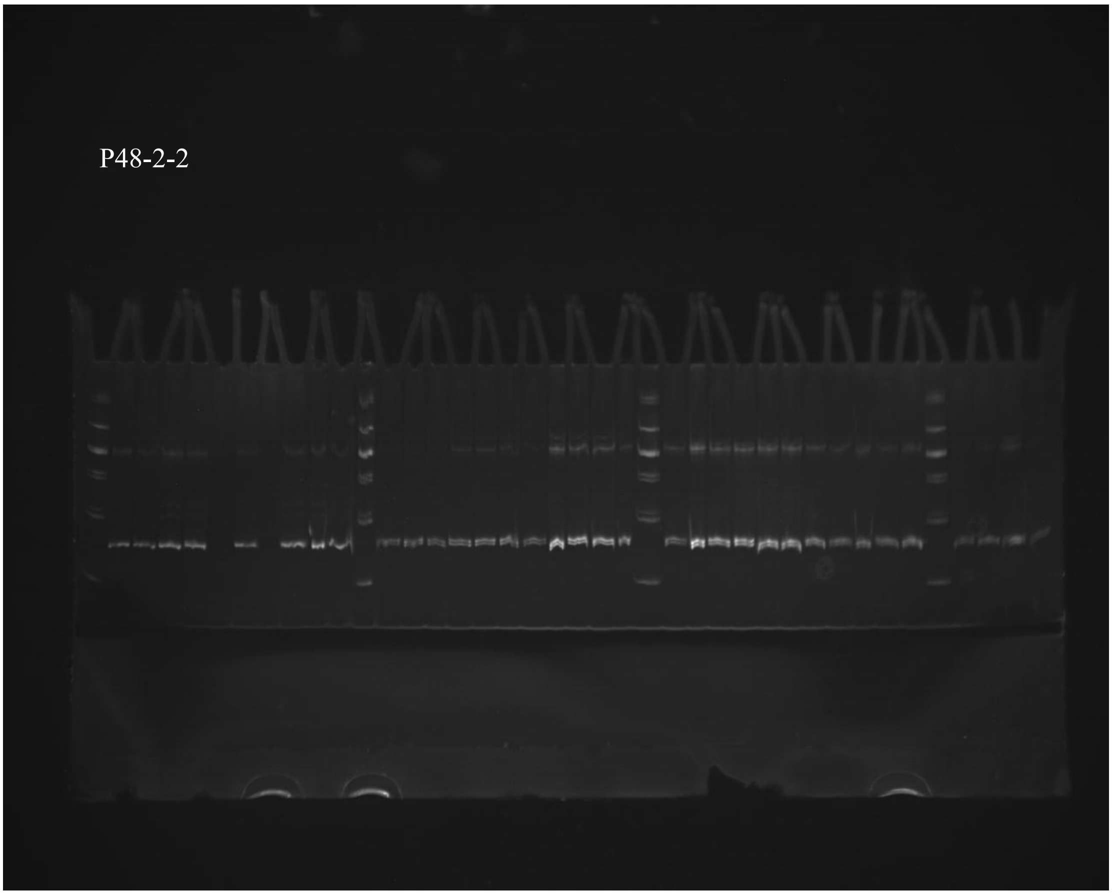
P48-2-2

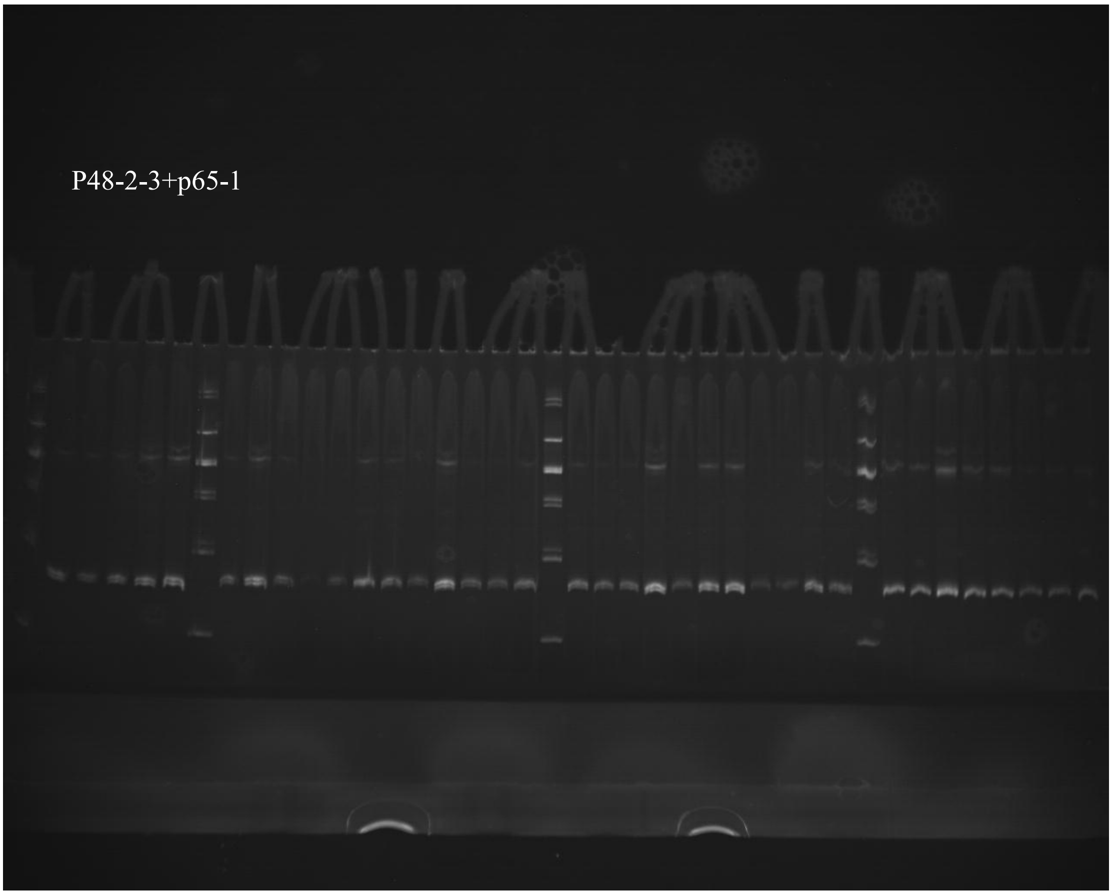
P48-2-3+p65-1

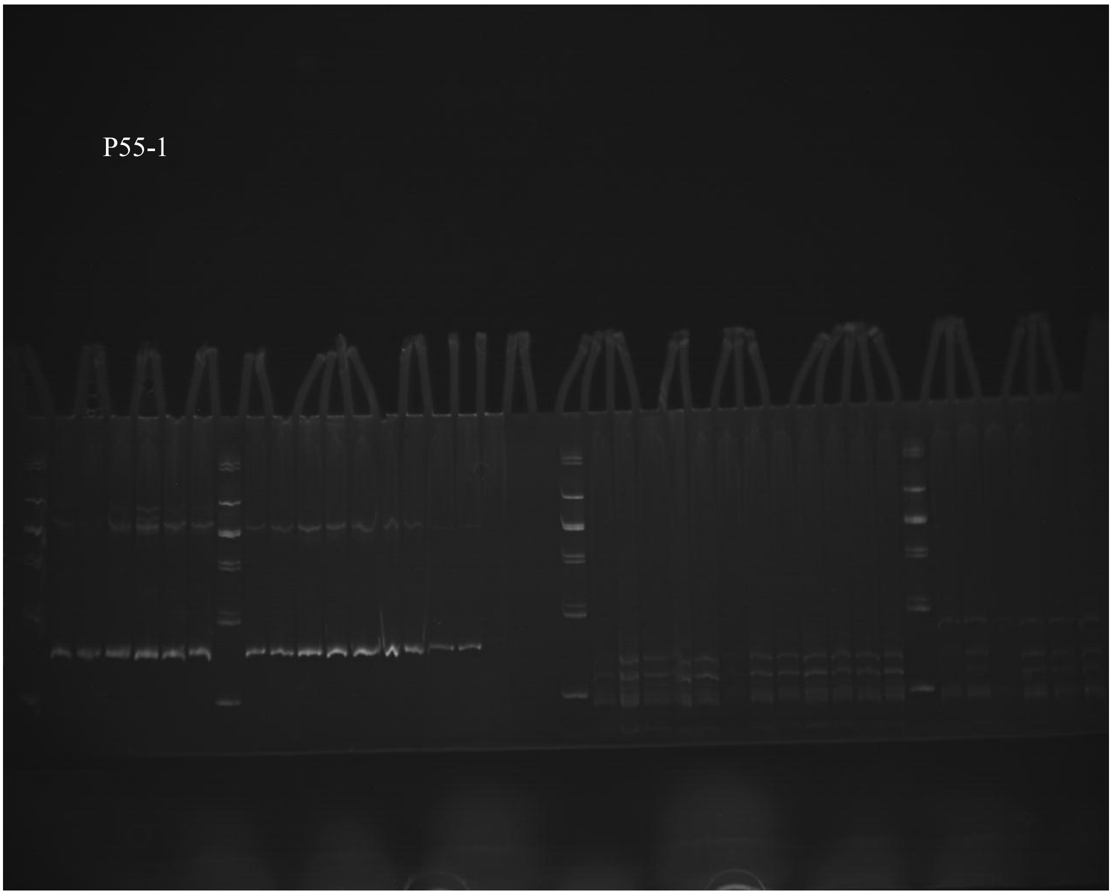
P55-1

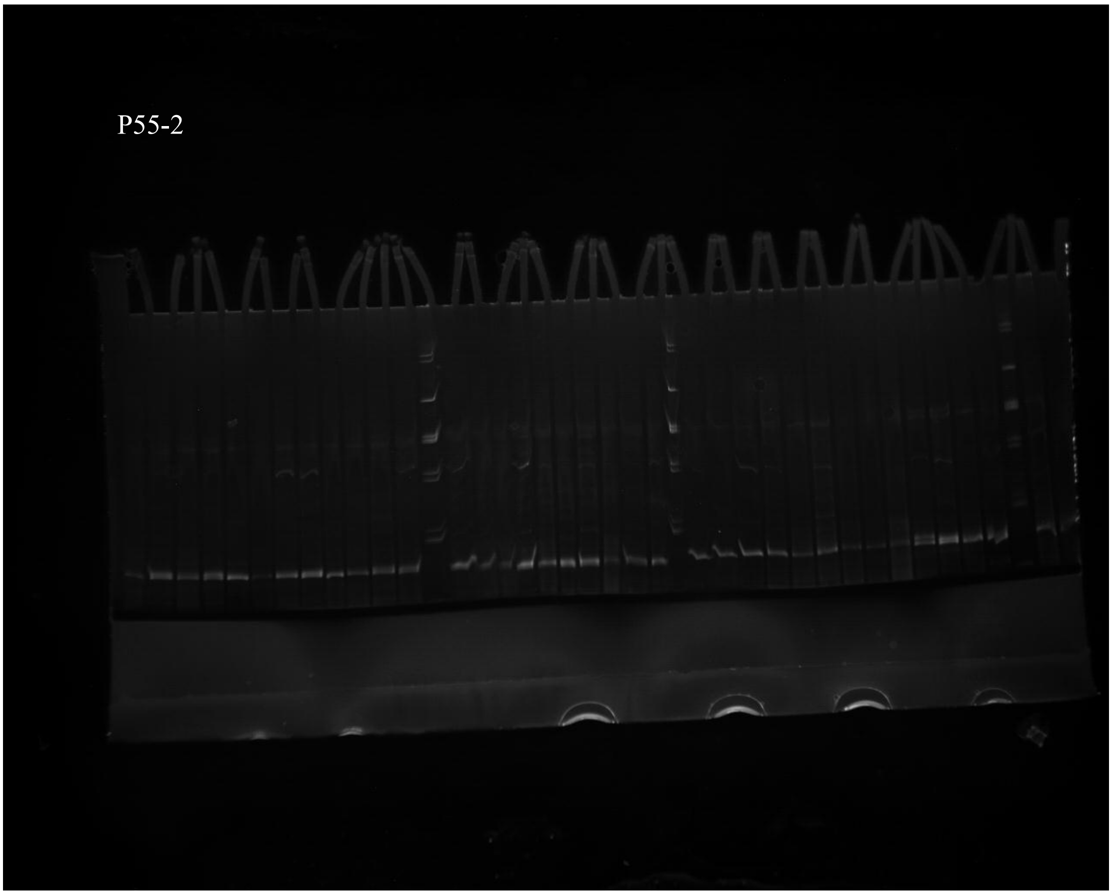

P55-2

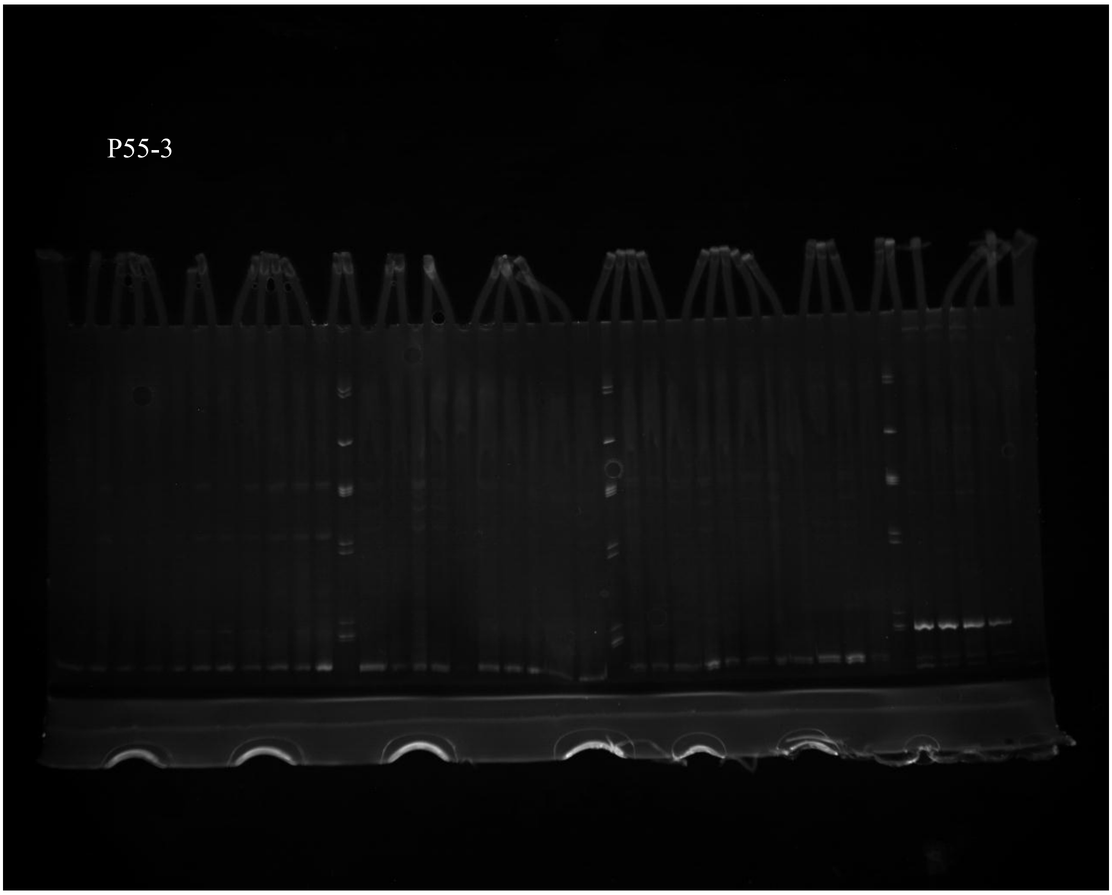

P55-3

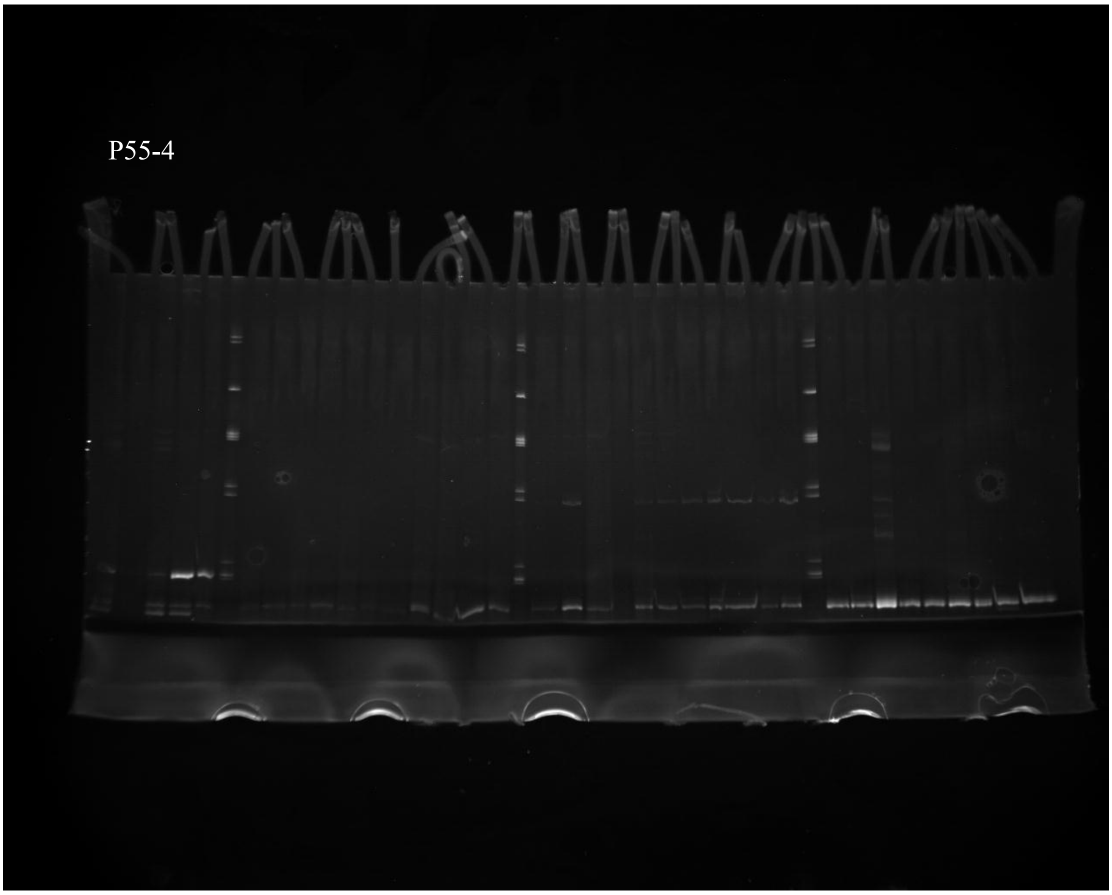
P55-4

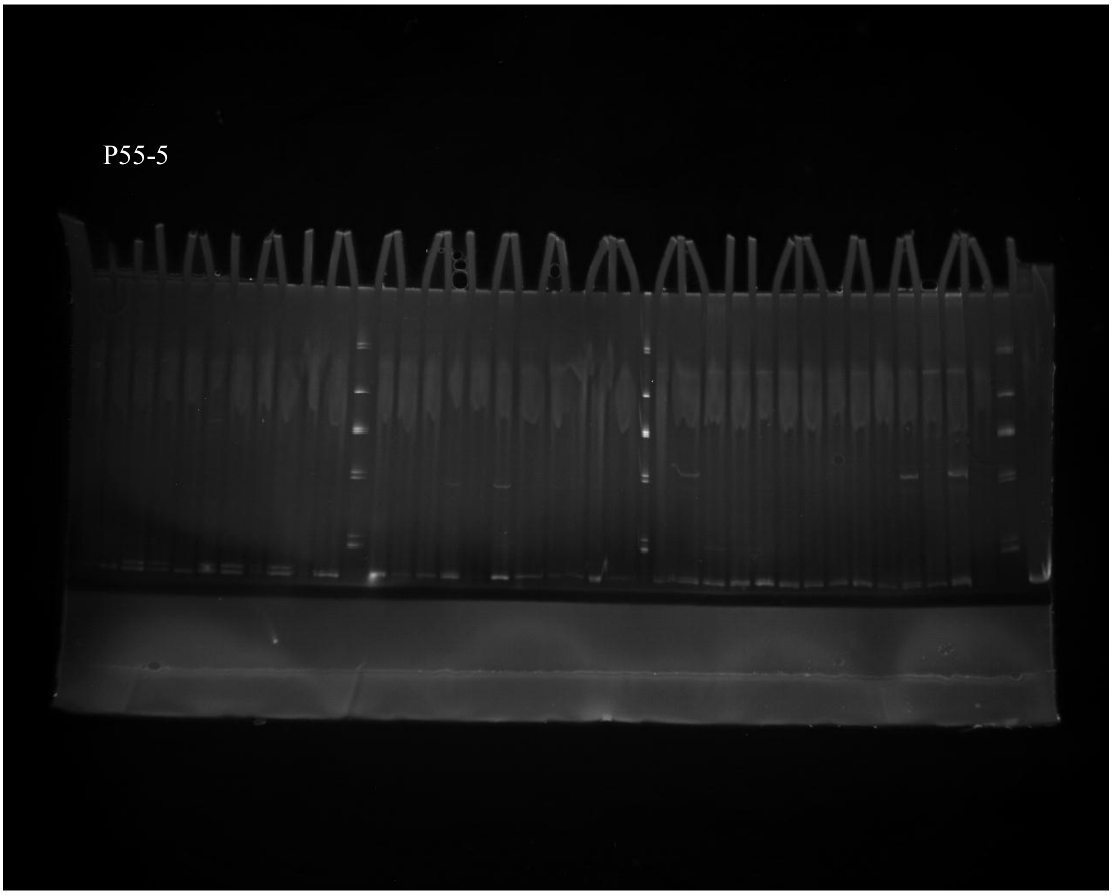
P55-5

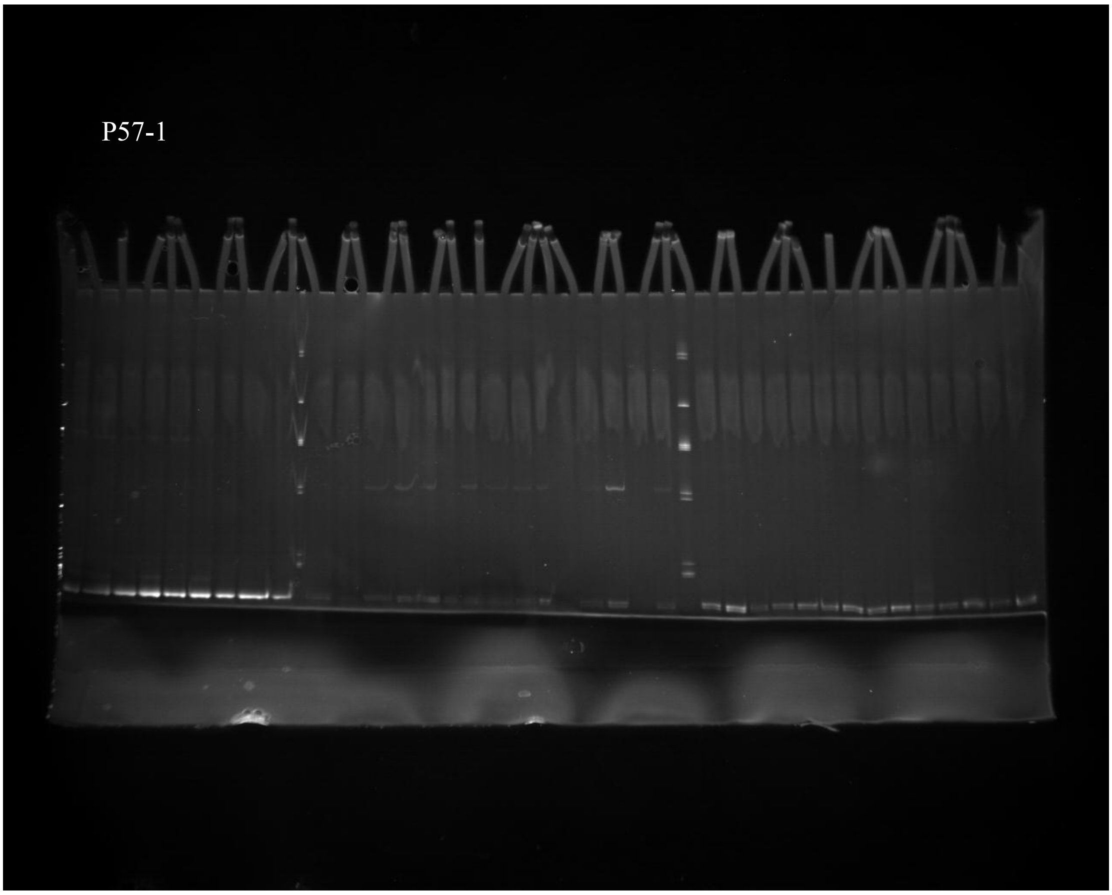
P57-1

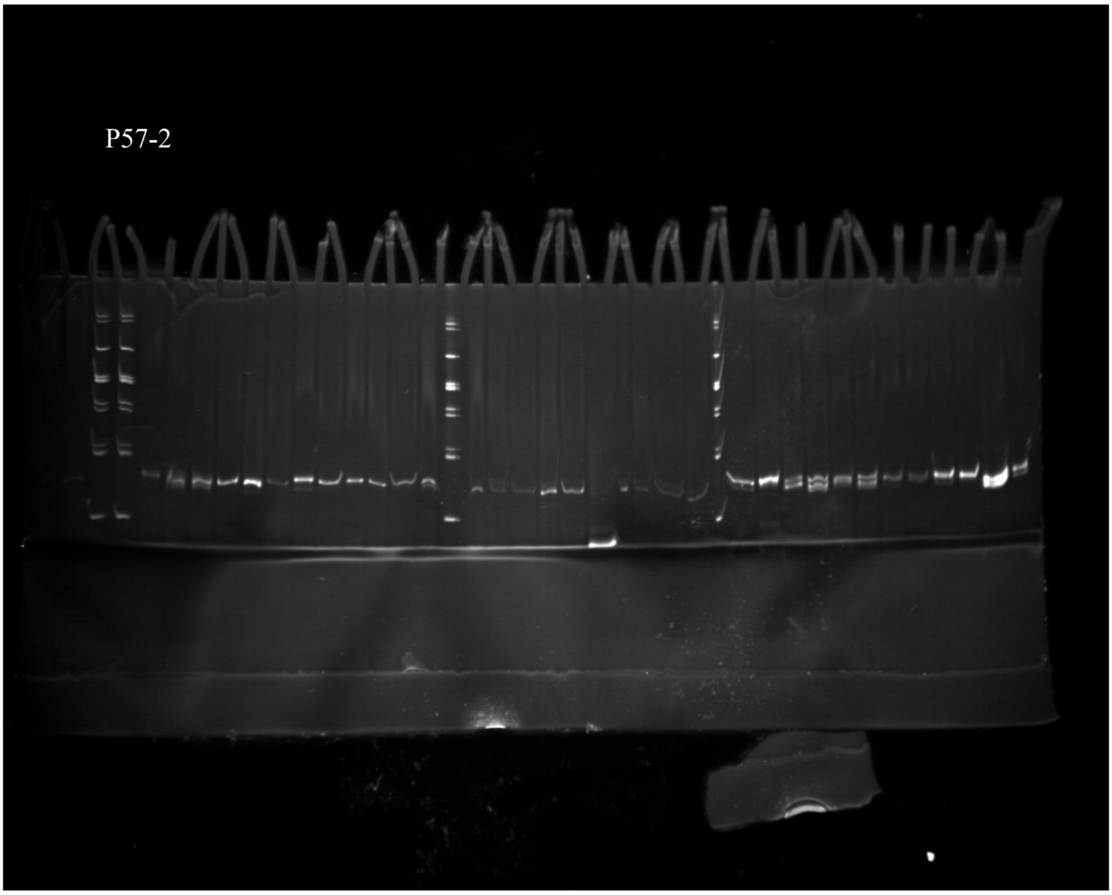
P57-2

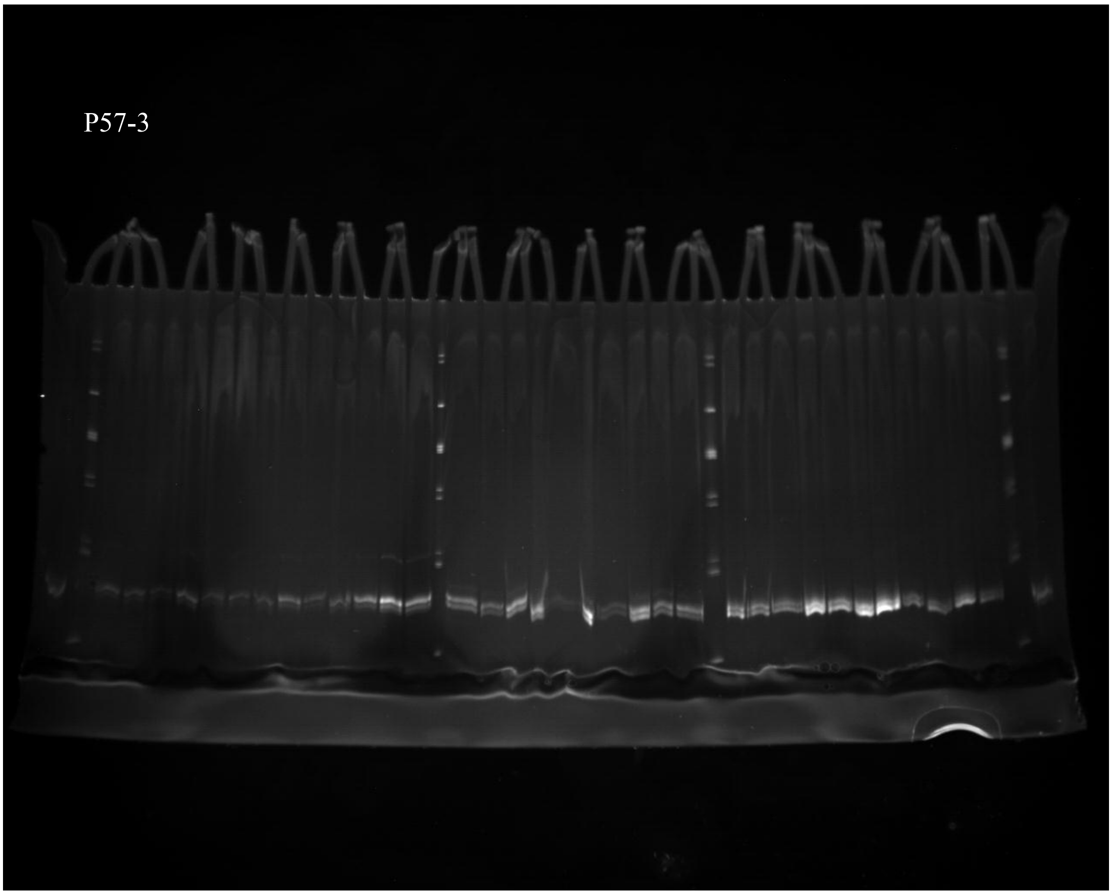
P57-3

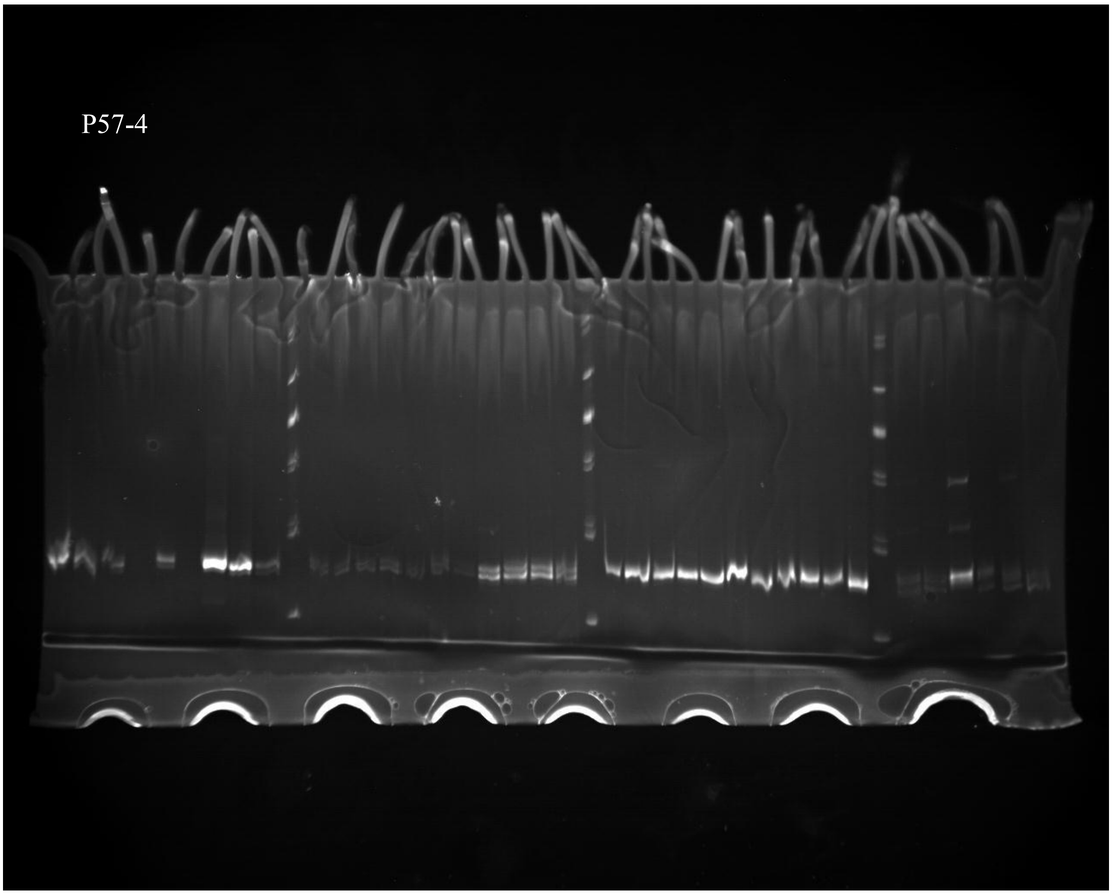
P57-4

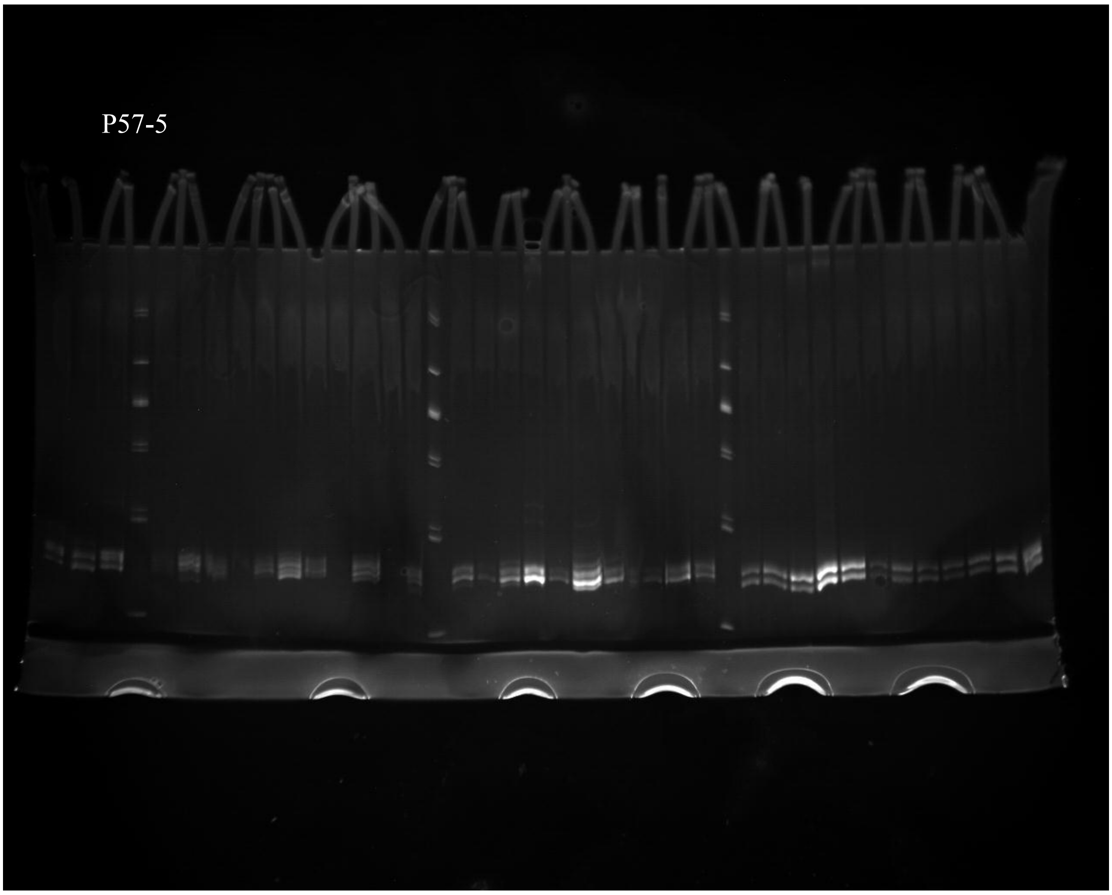
P57-5

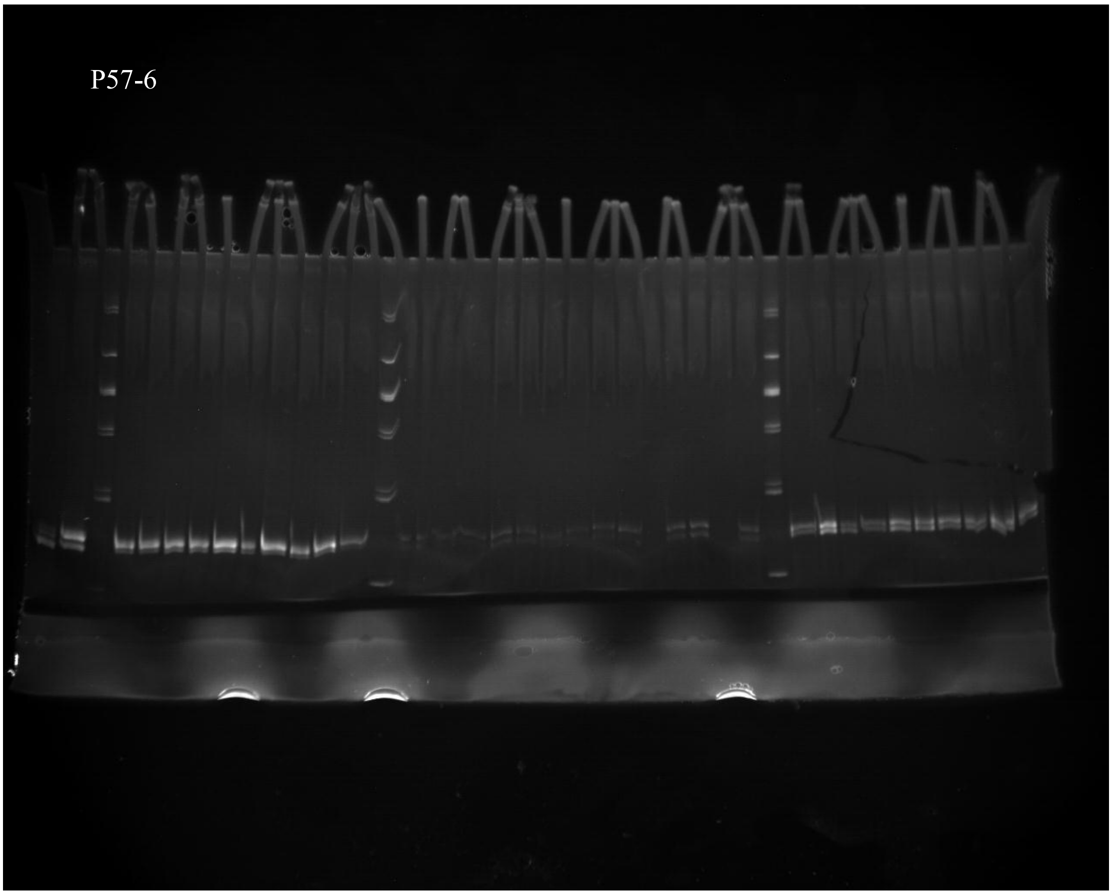
P57-6

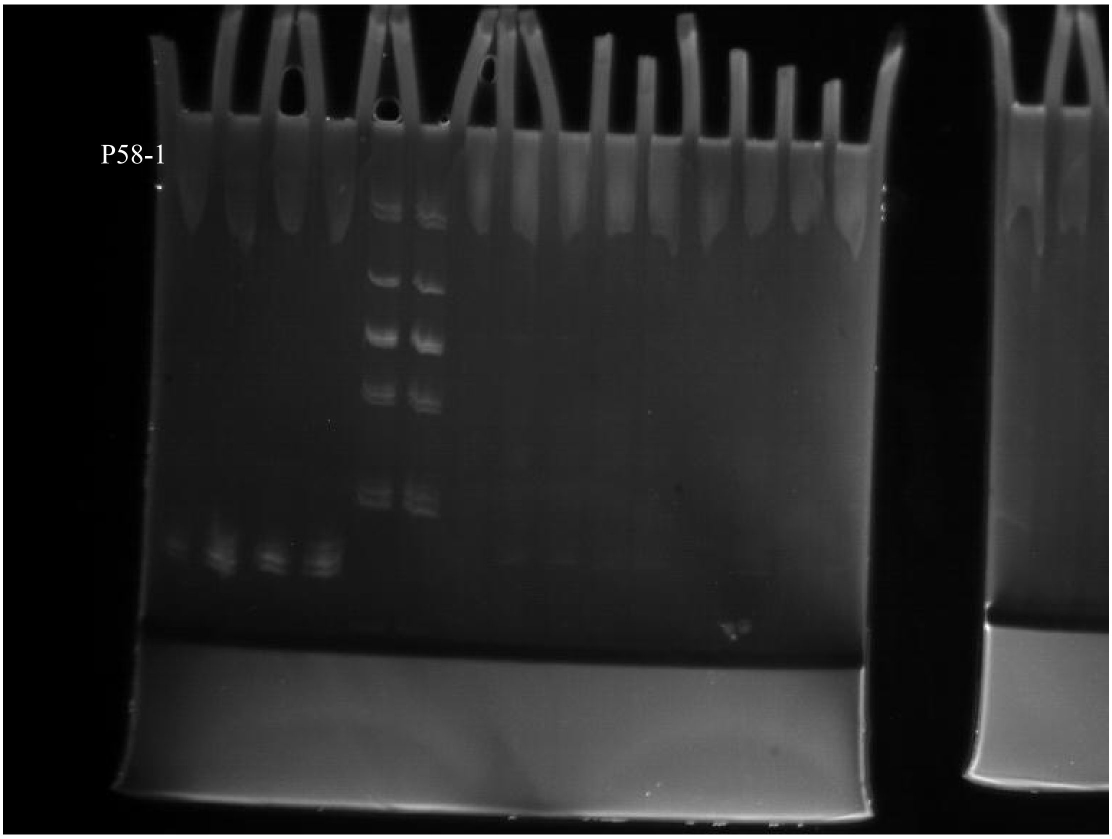
P58-1

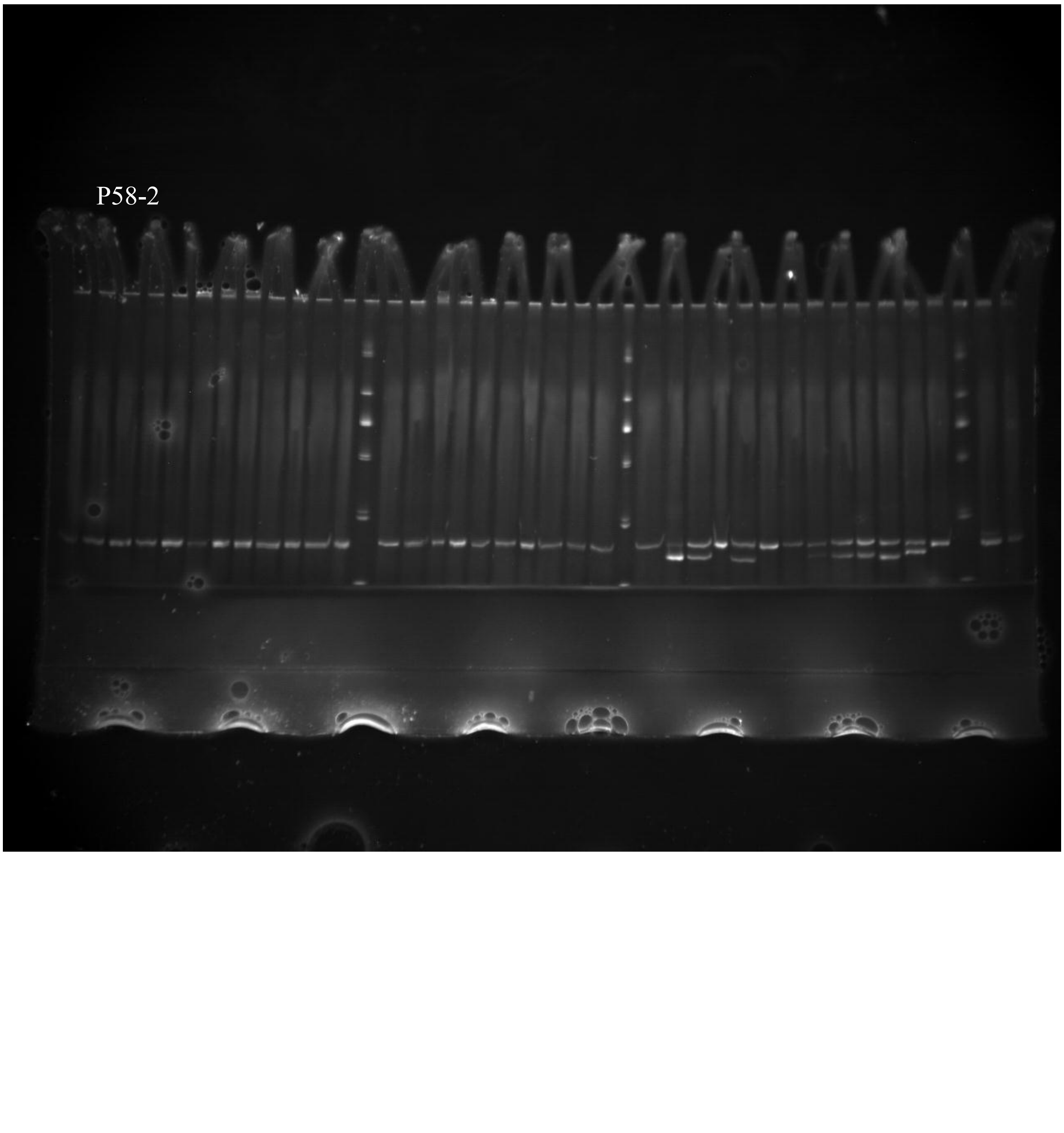
P58-2

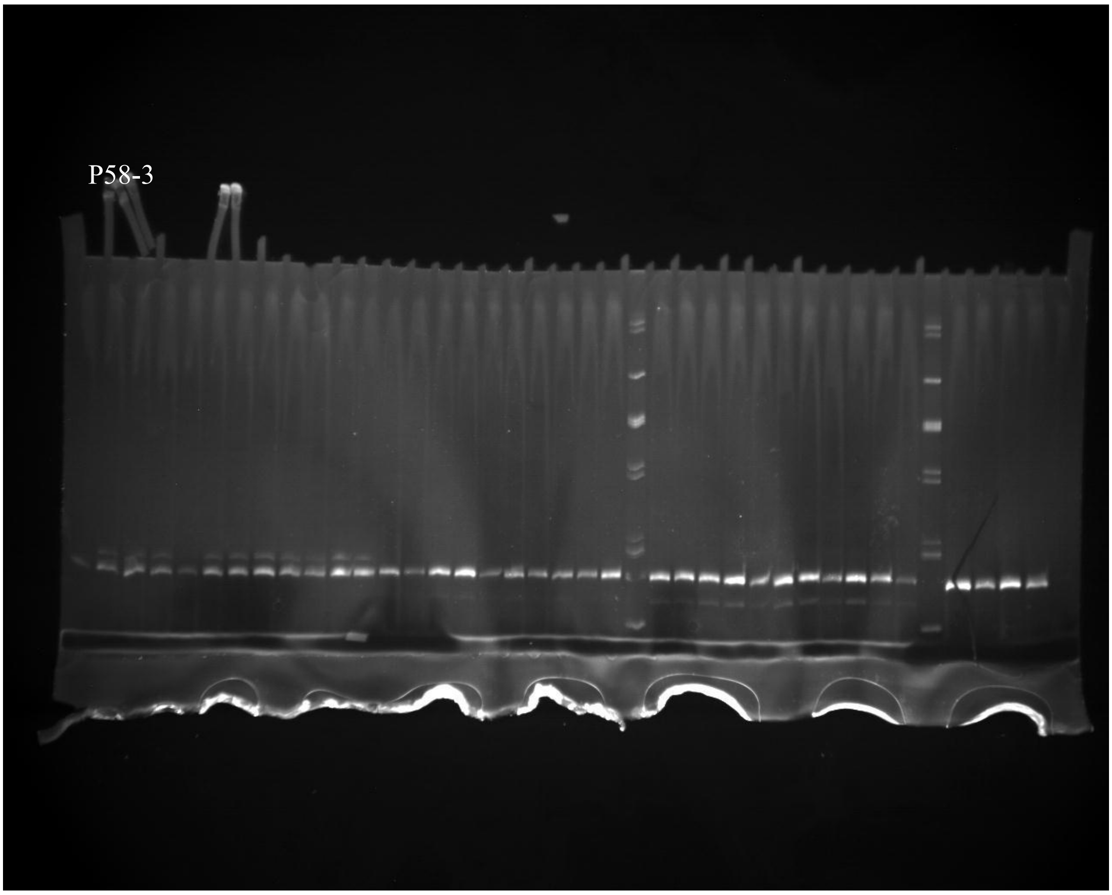
P58-3

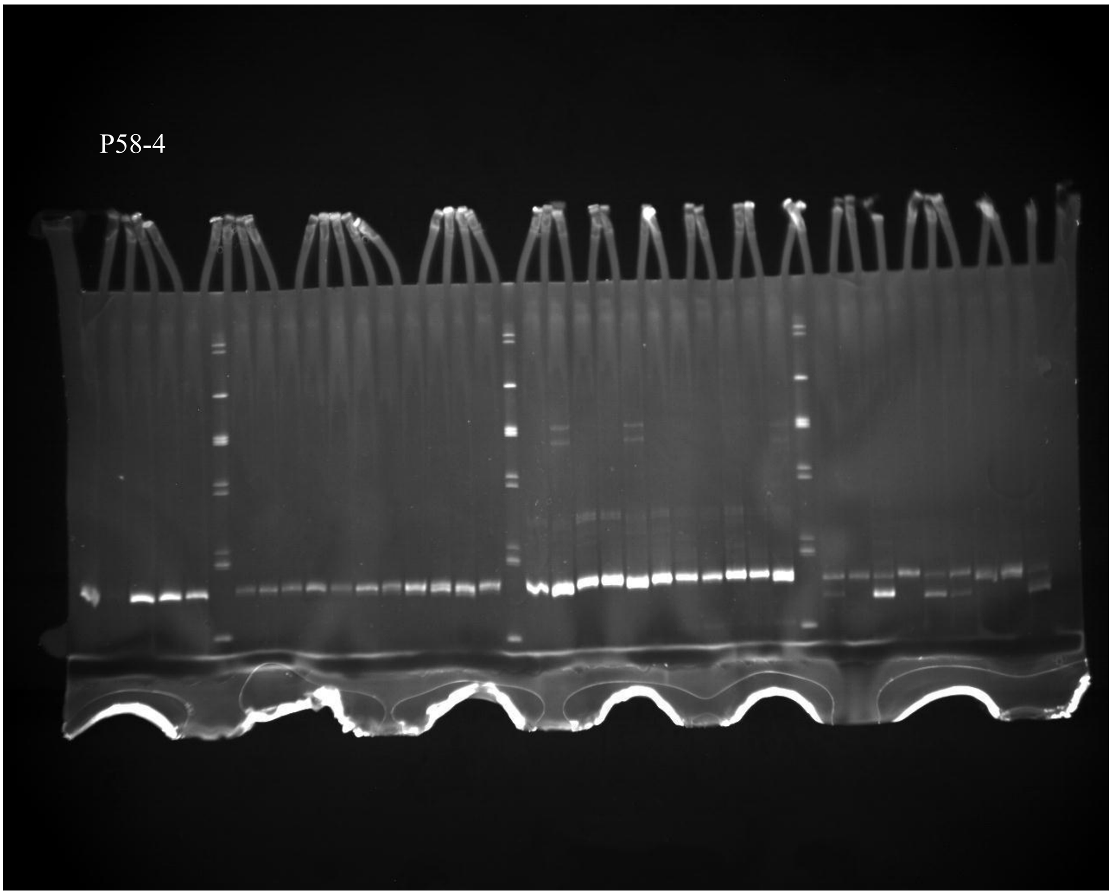
P58-4

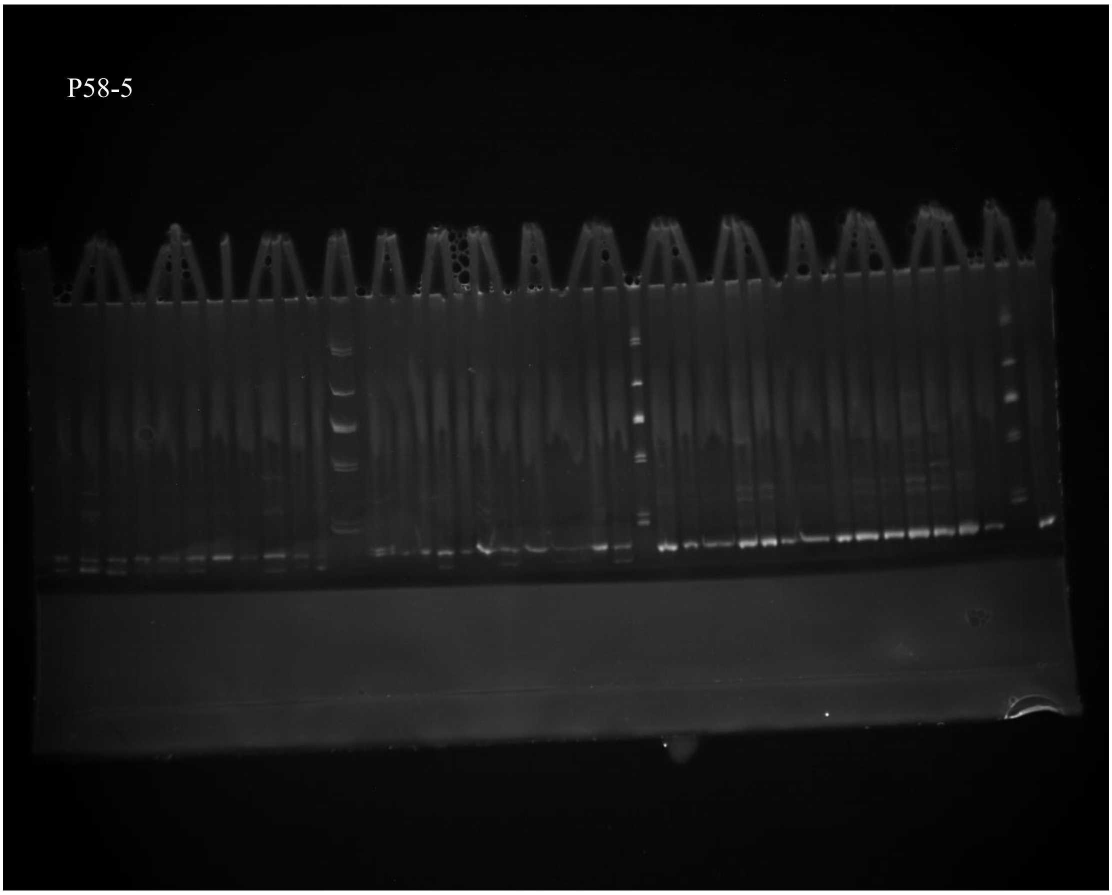
P58-5

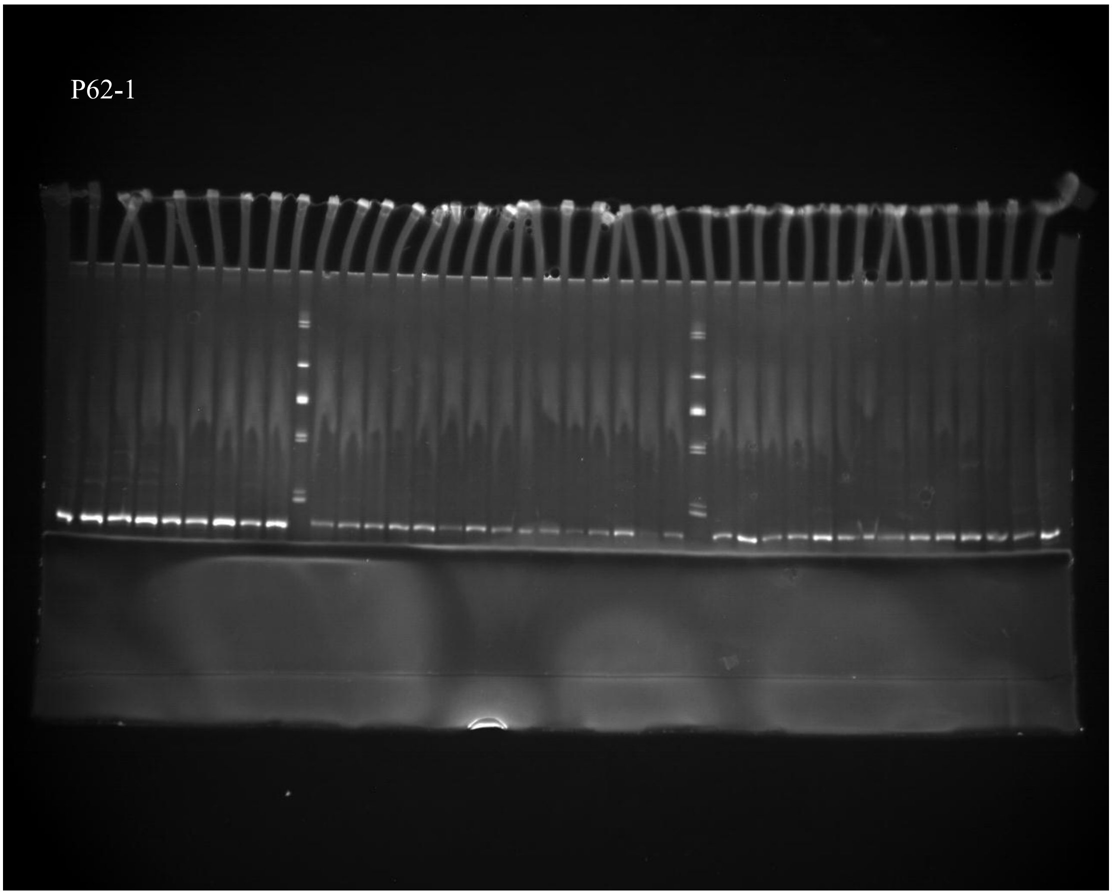
P62-1

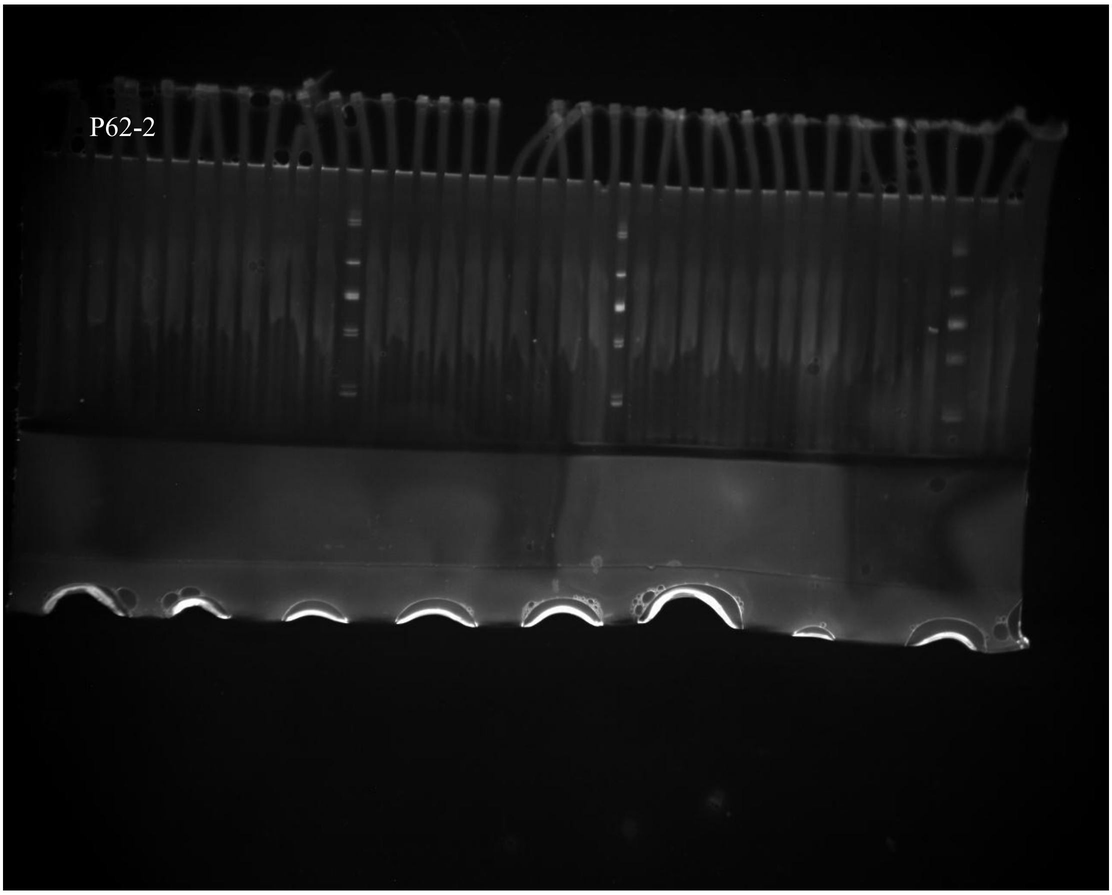
P62-2

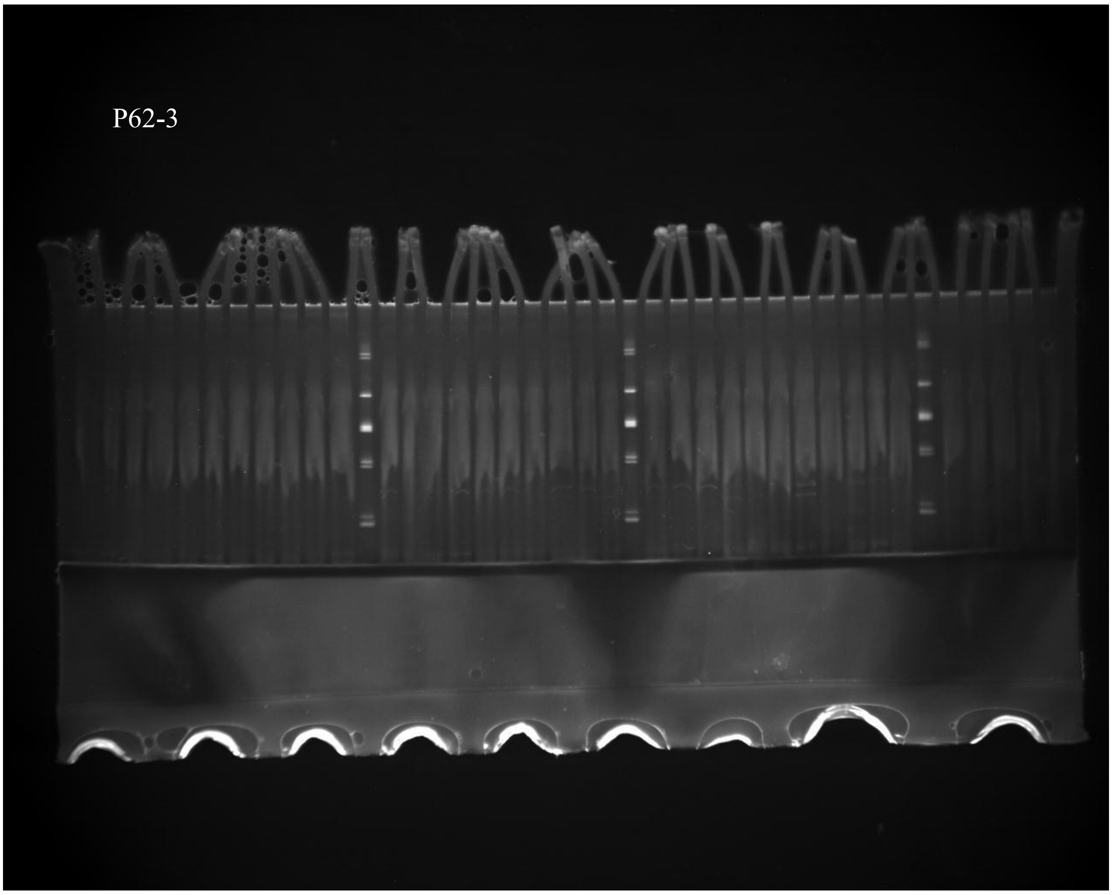
P62-3

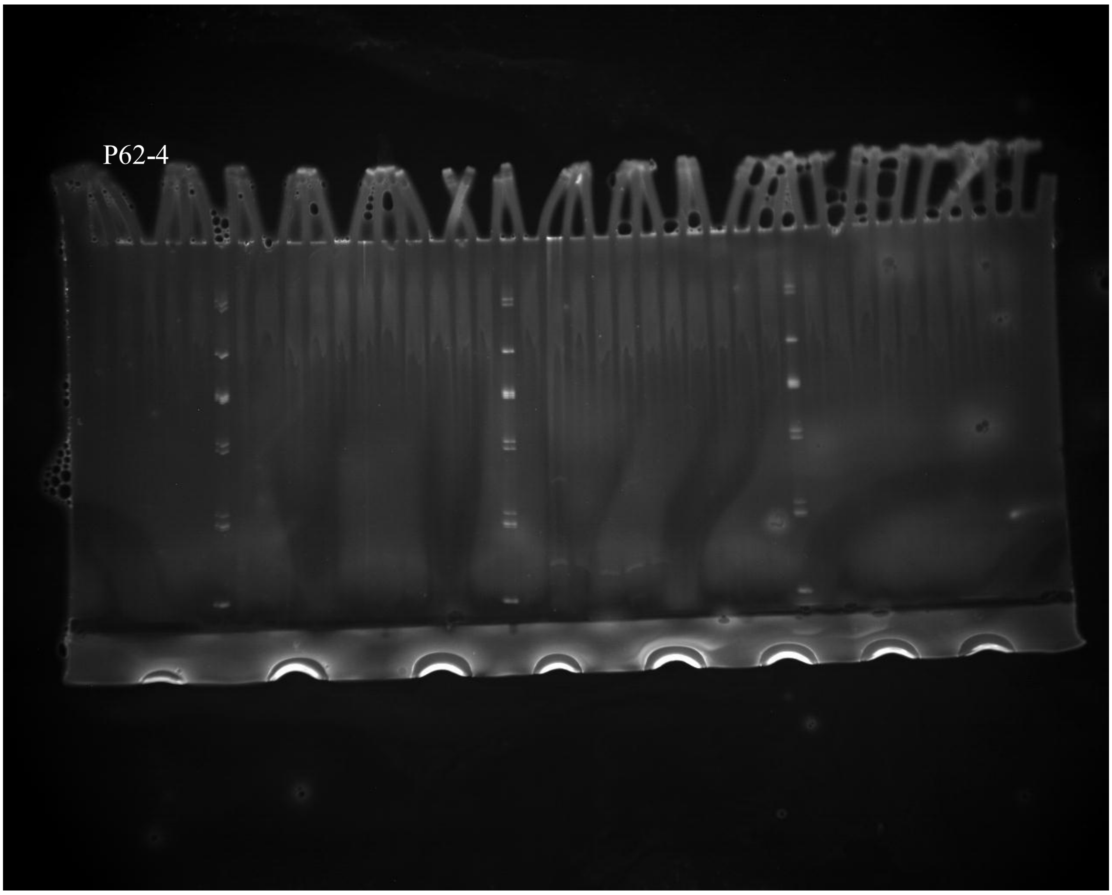
P62-4

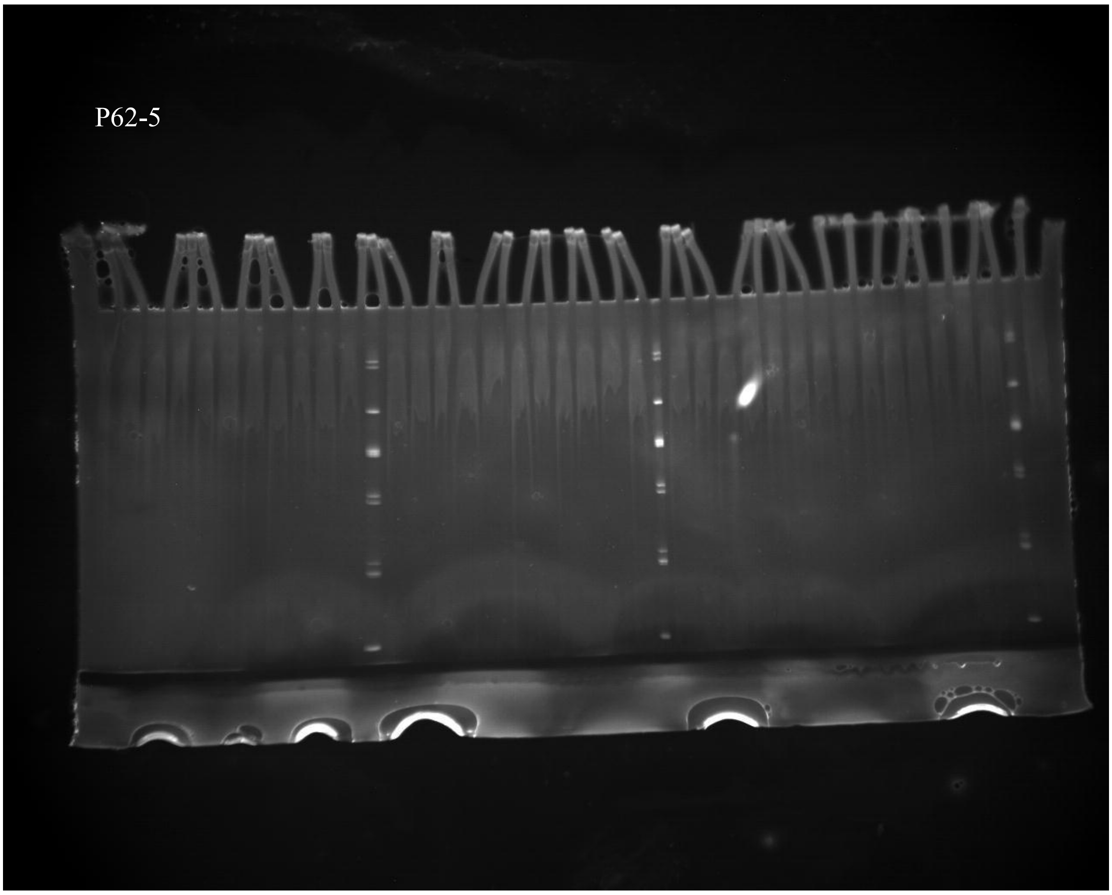
P62-5

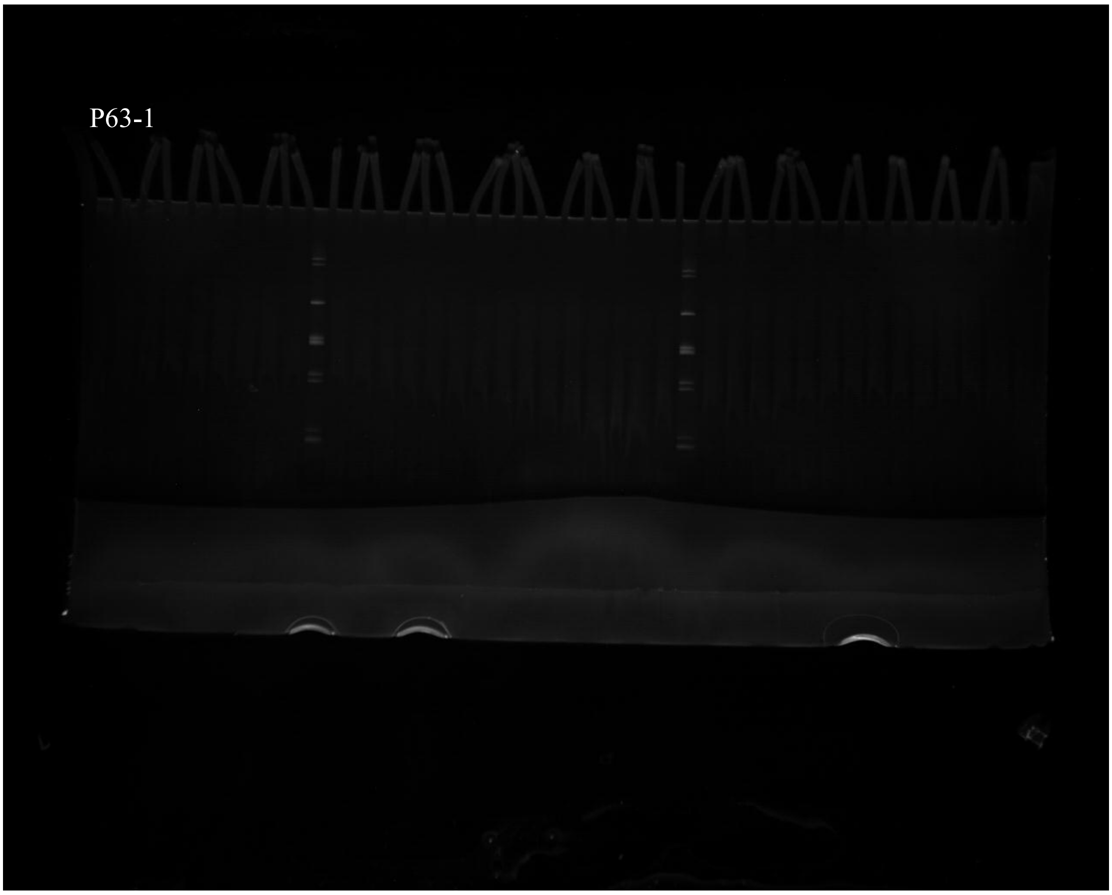
P63-1

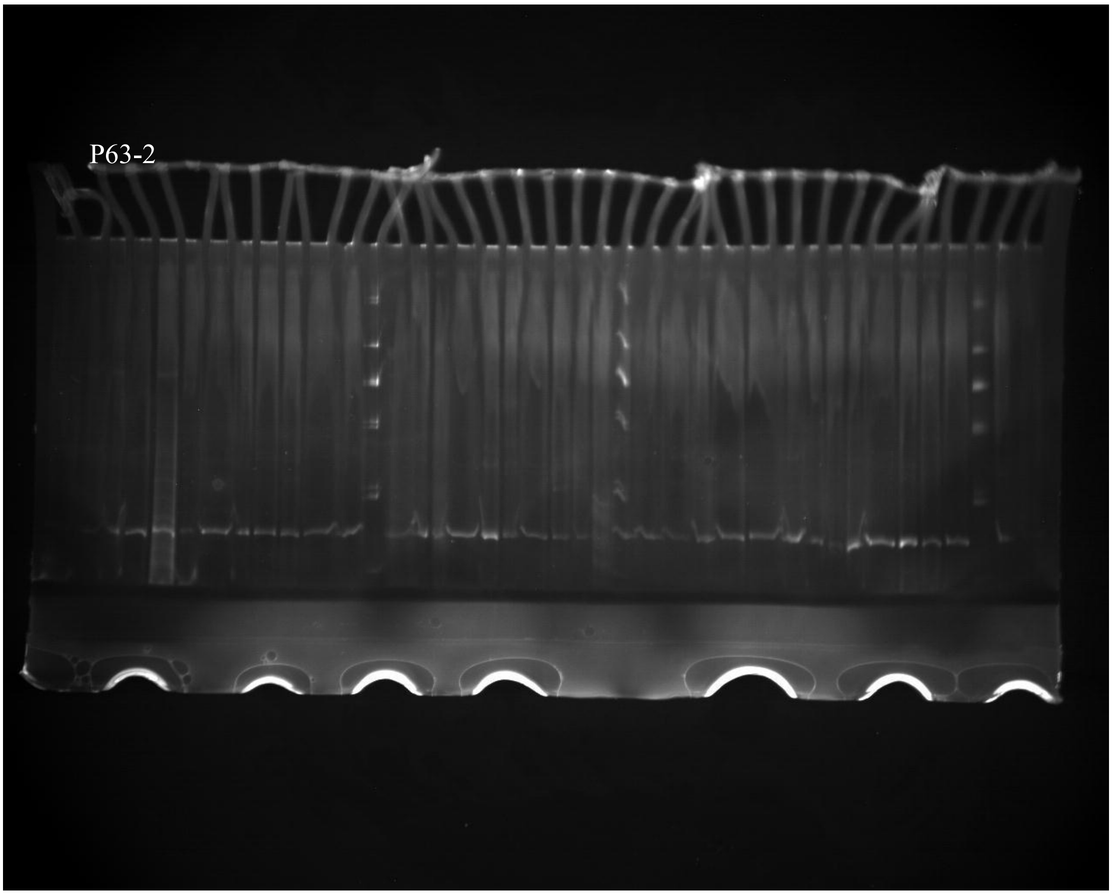
P63-2

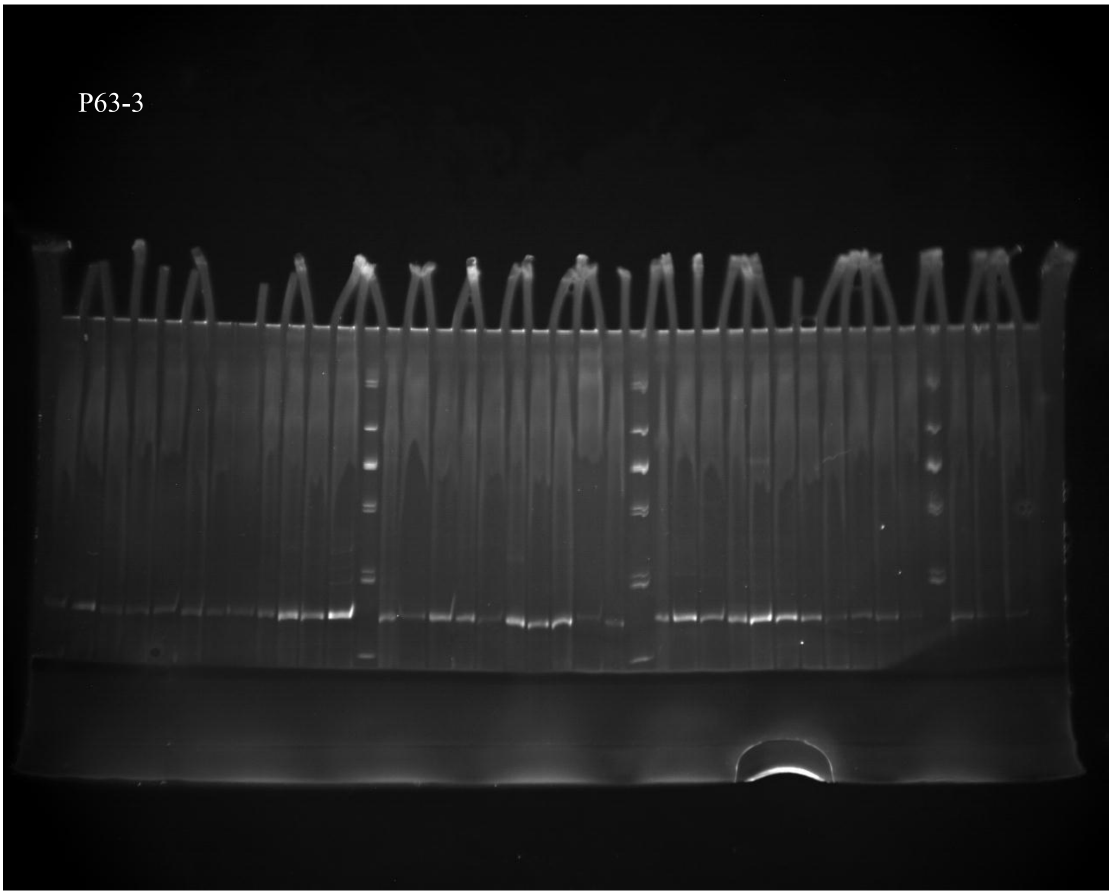
P63-3

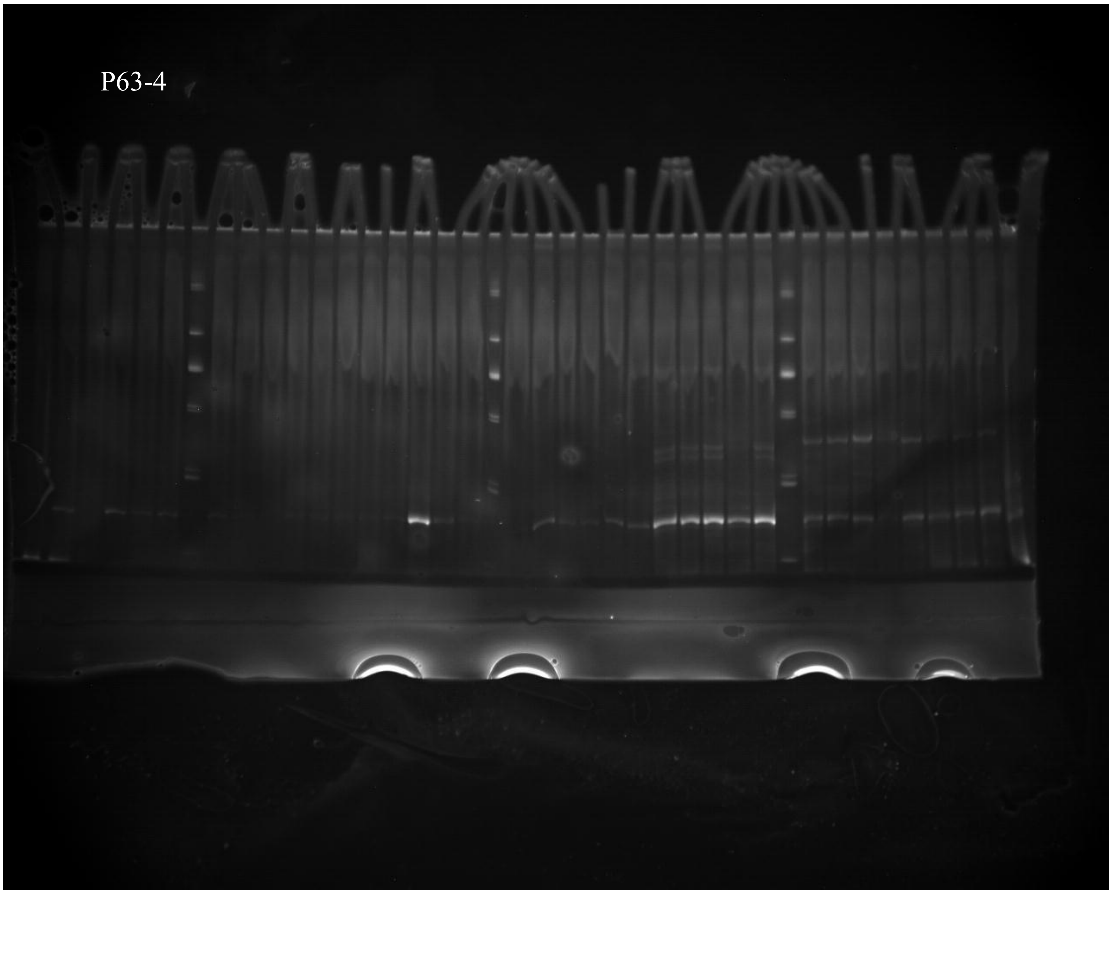
P63-4

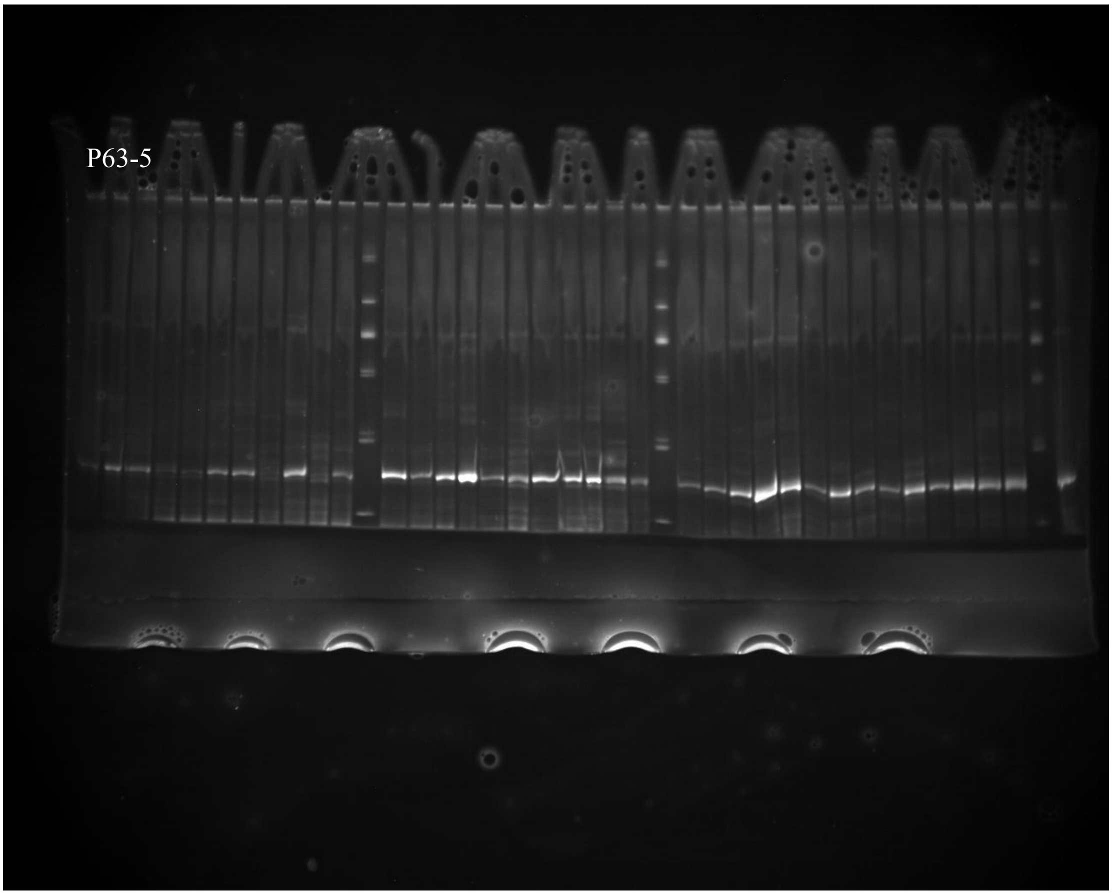
P63-5

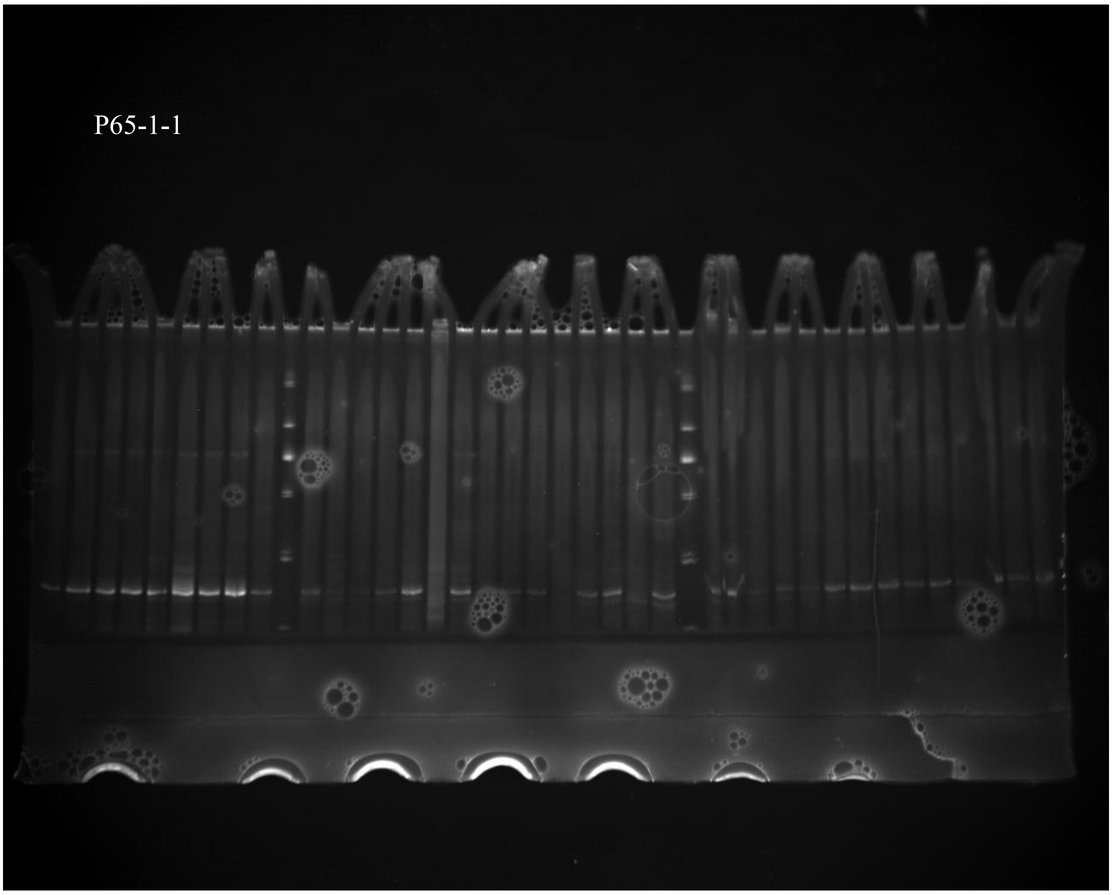
P65-1-1

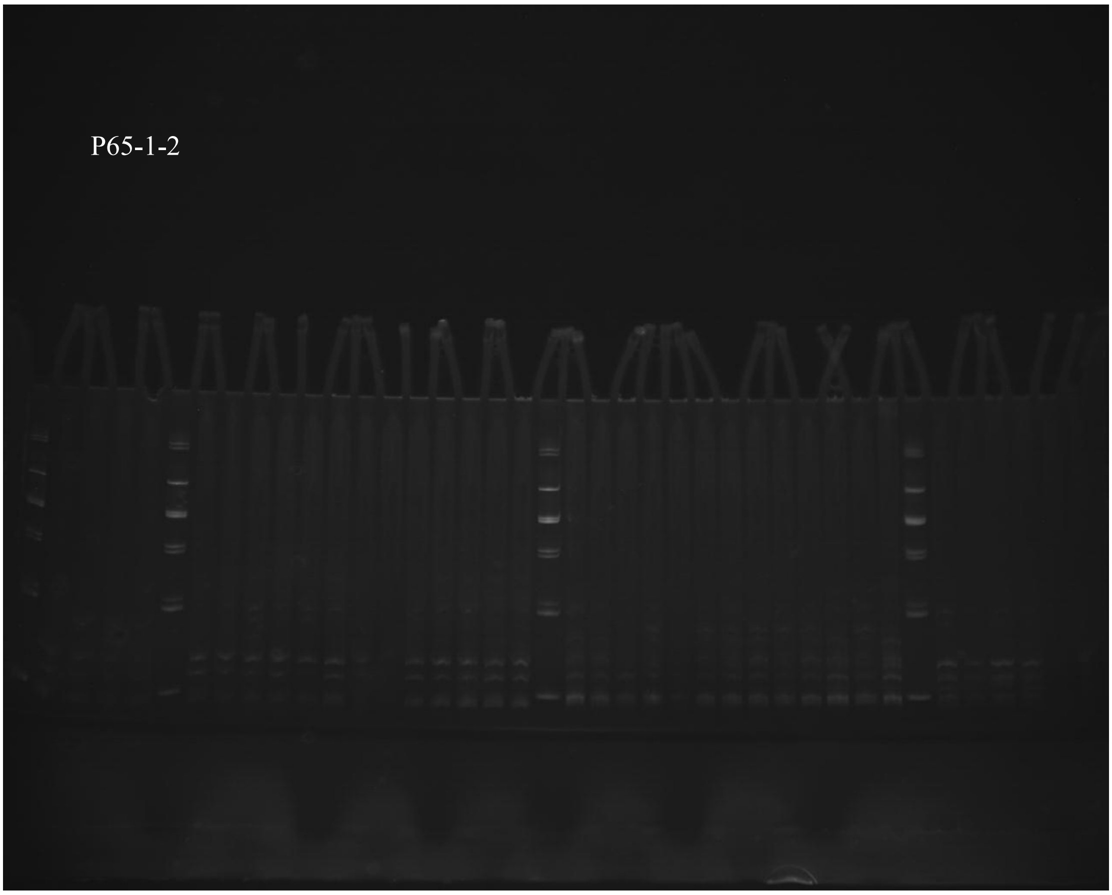
P65-1-2

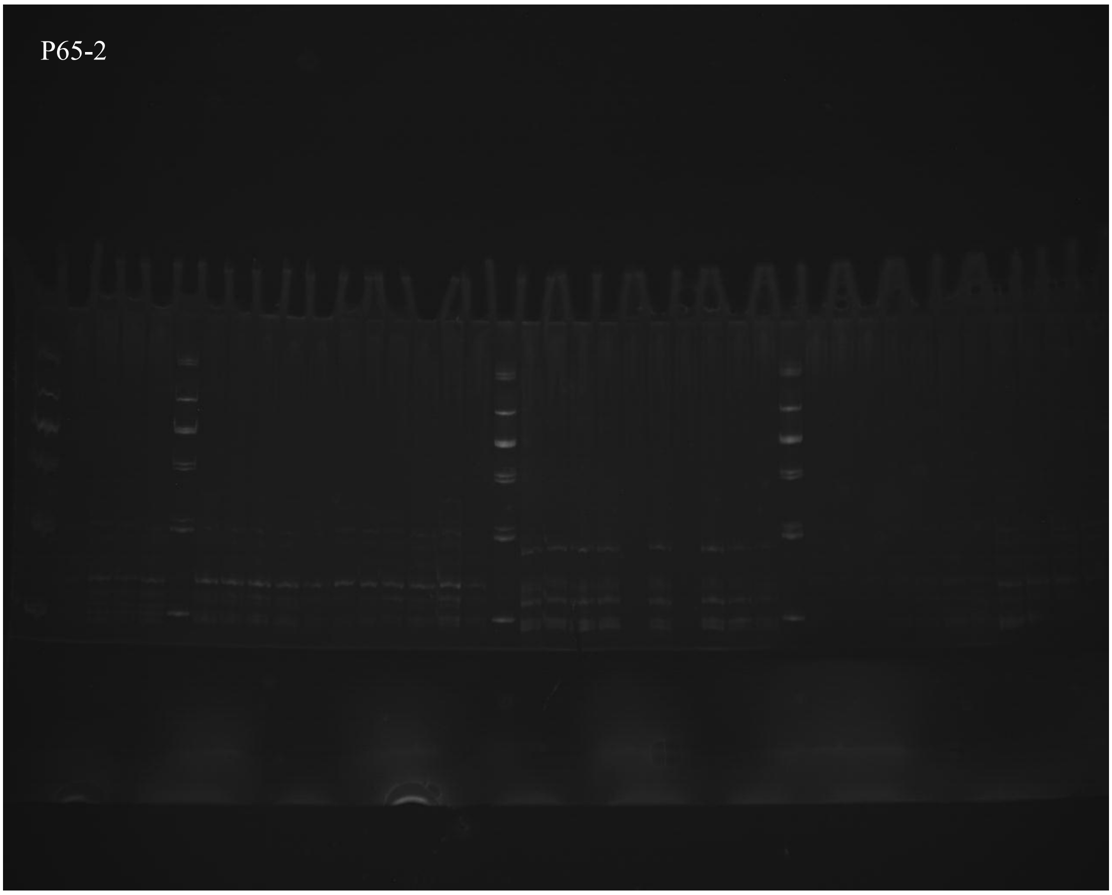
P65-2

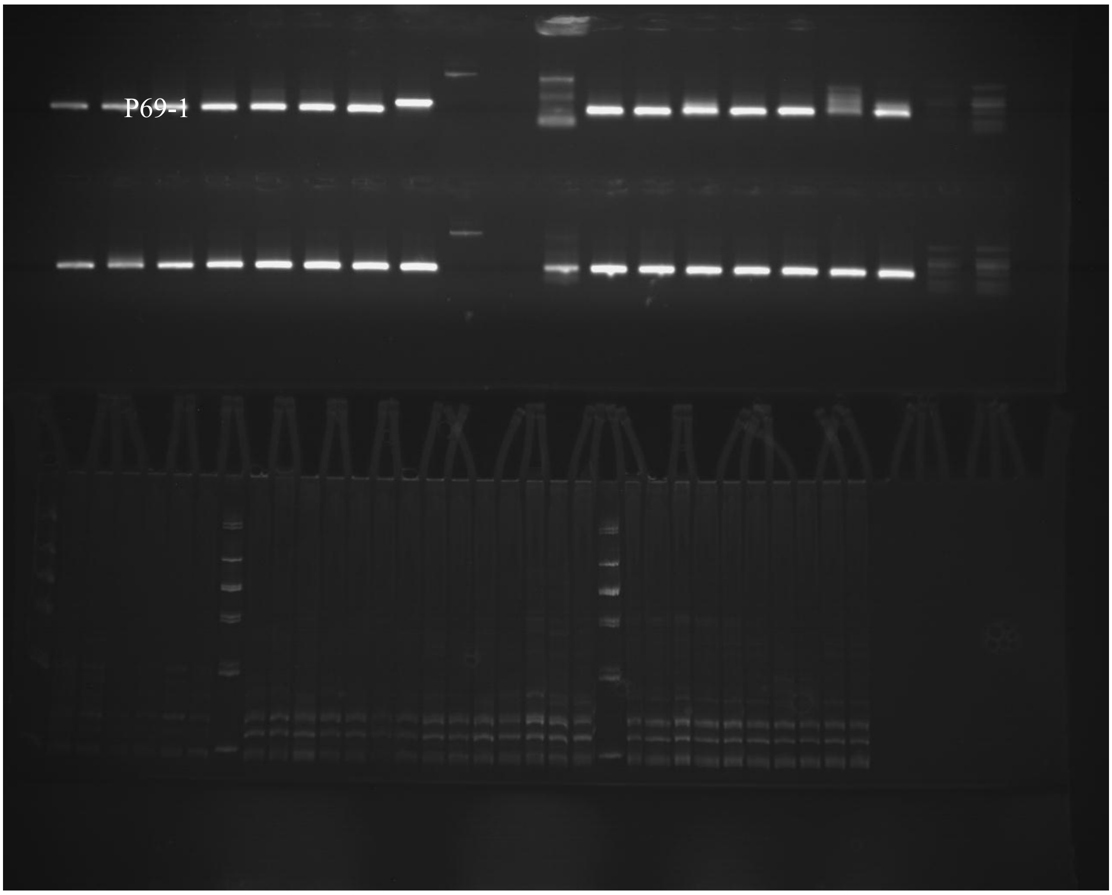
P69-1

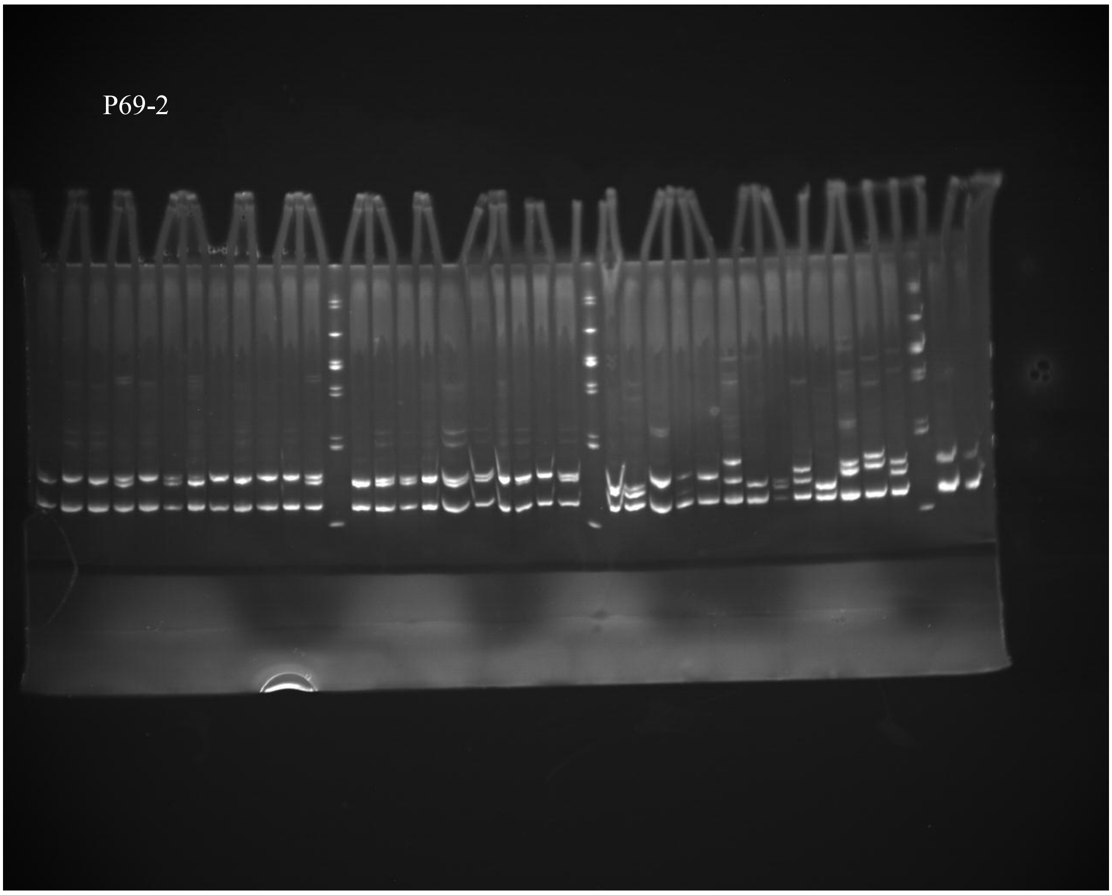
P69-2

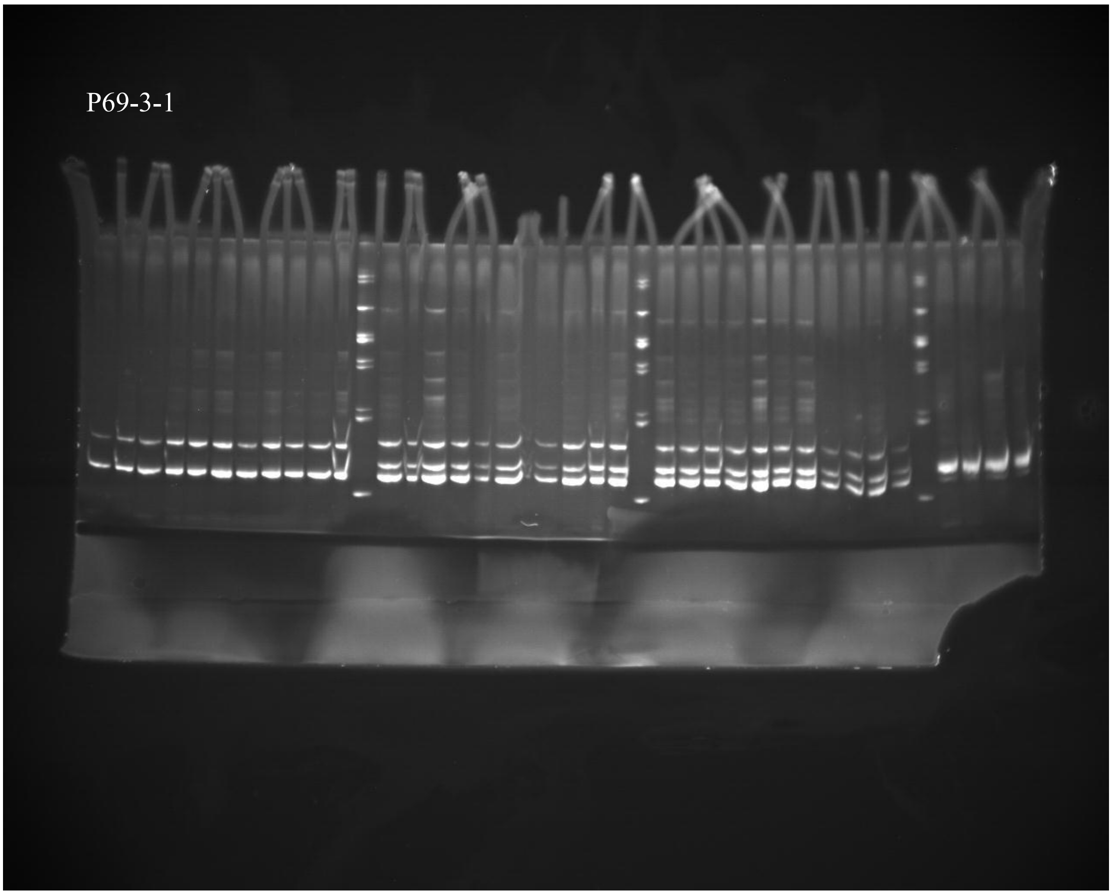
P69-3-1

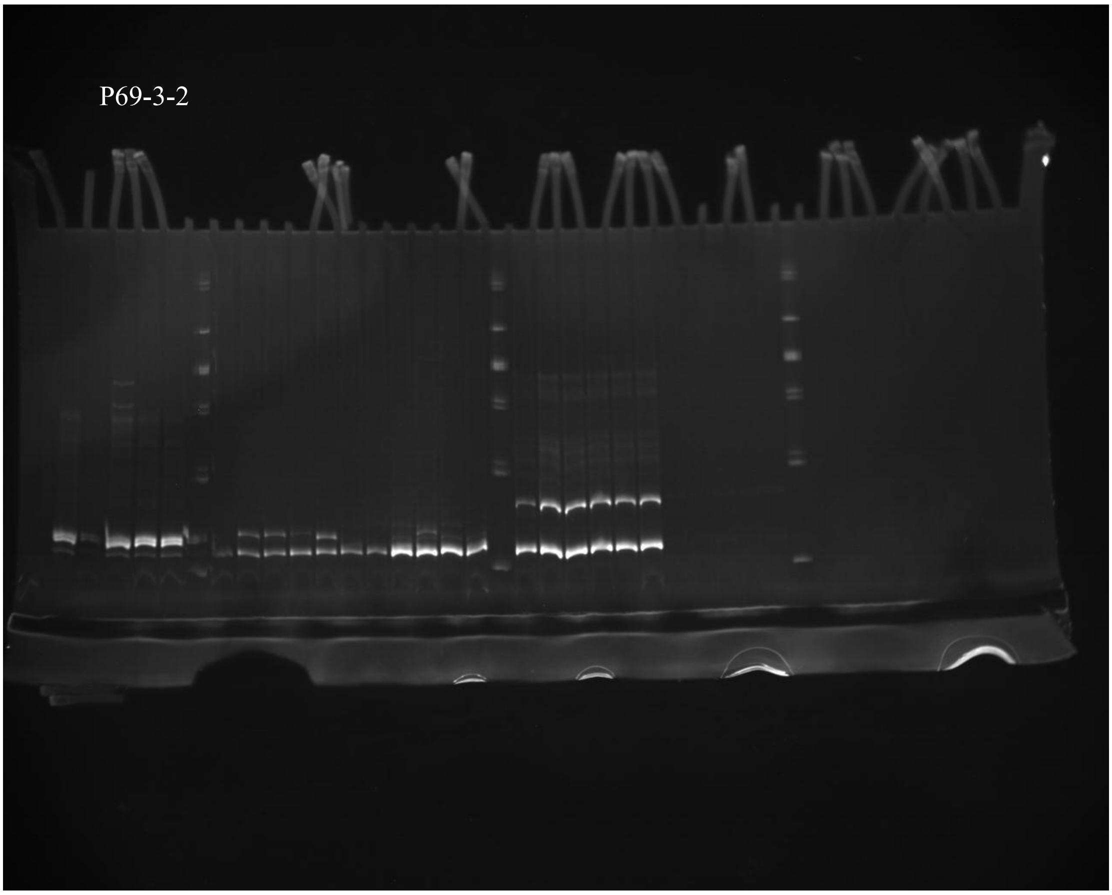
P69-3-2

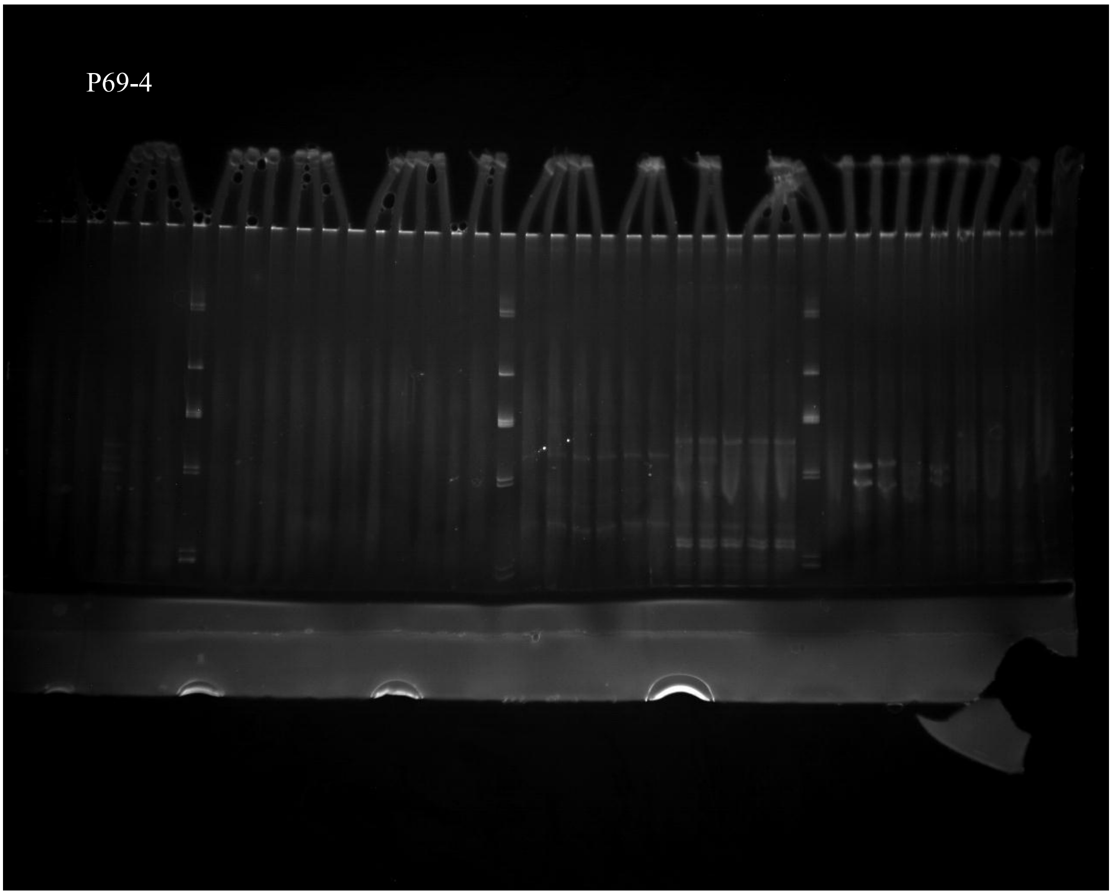
P69-4

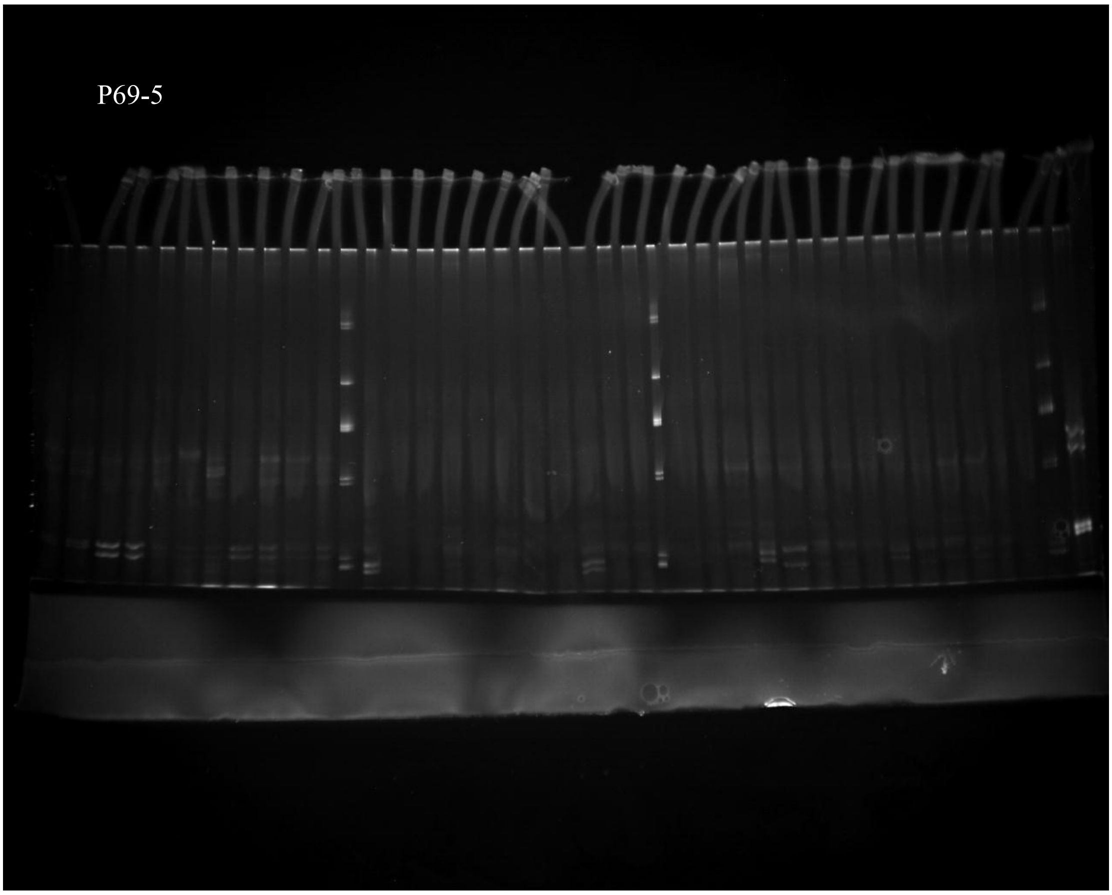
P69-5

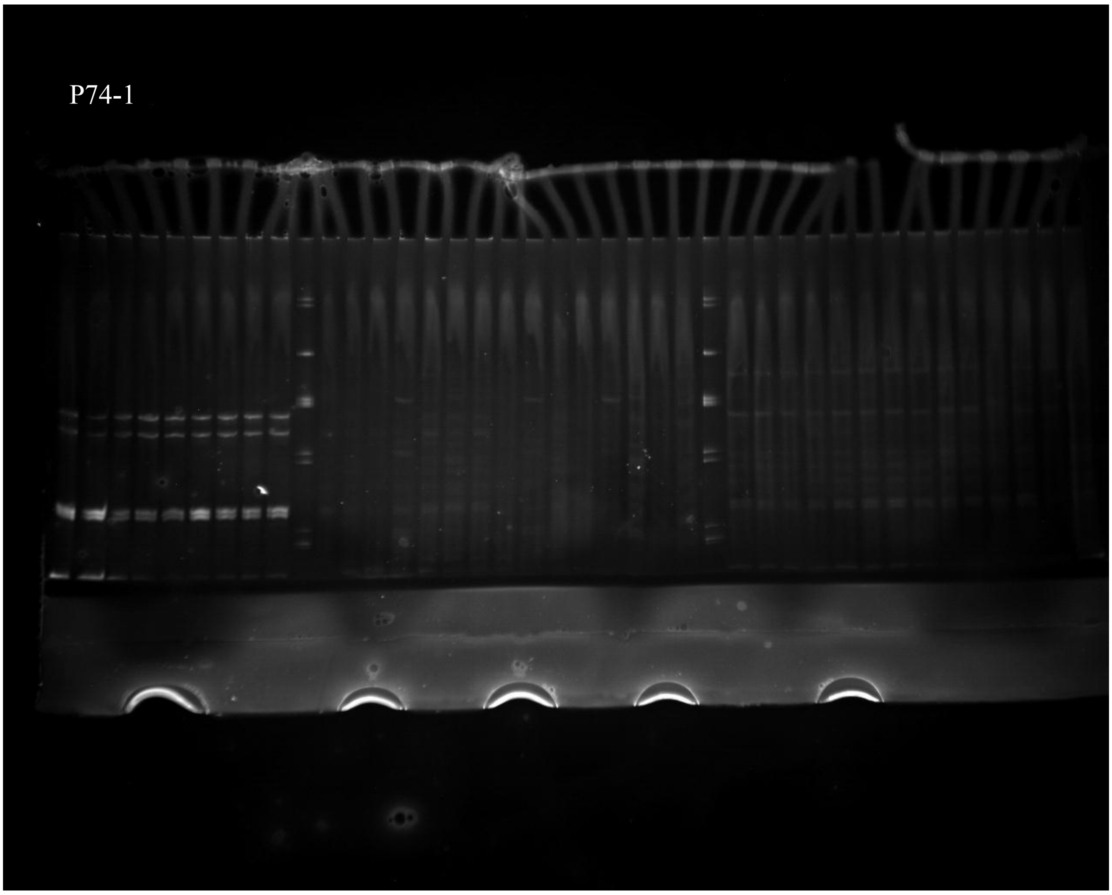
P74-1

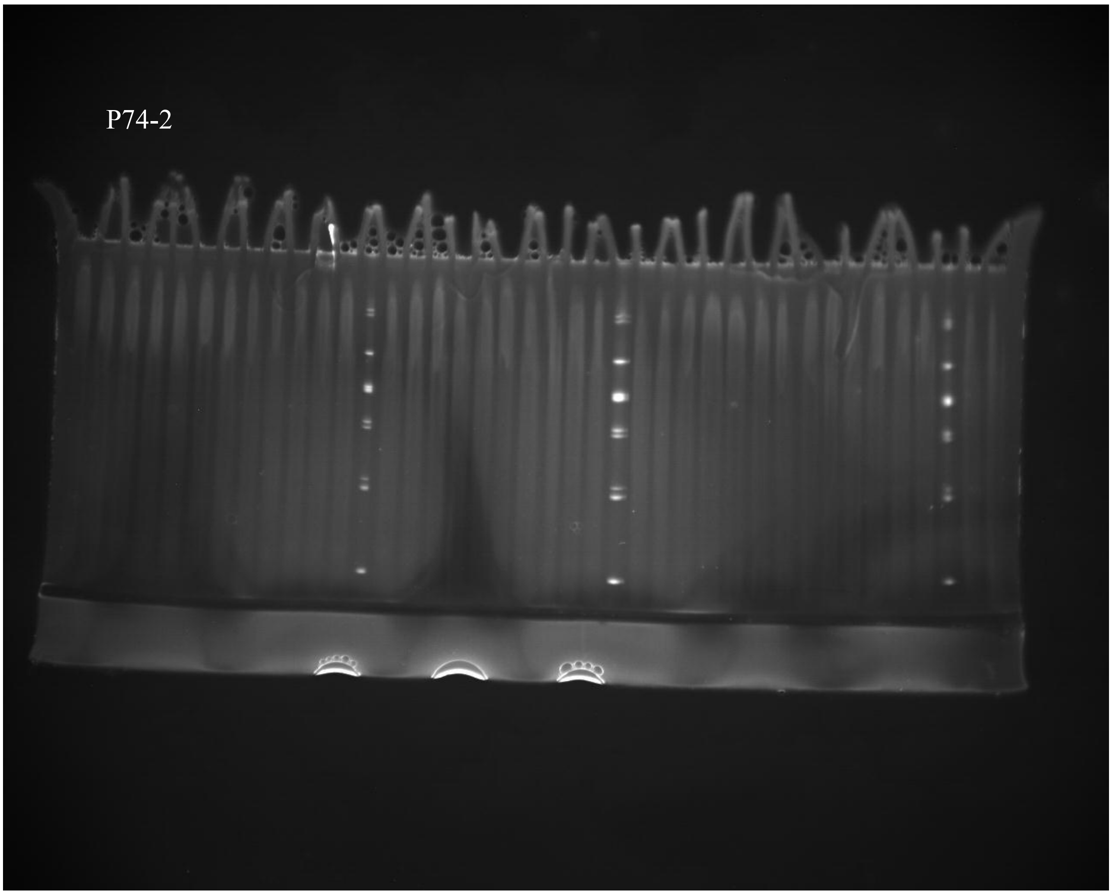
P74-2

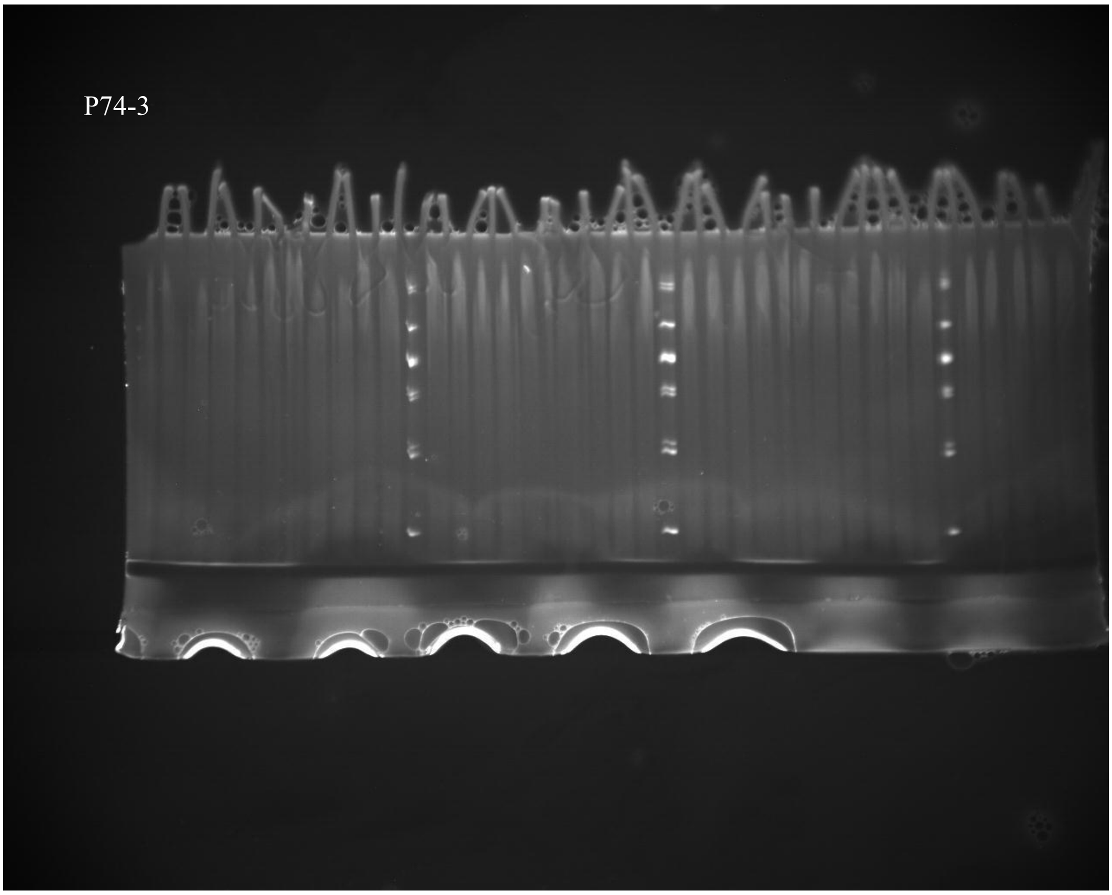
P74-3

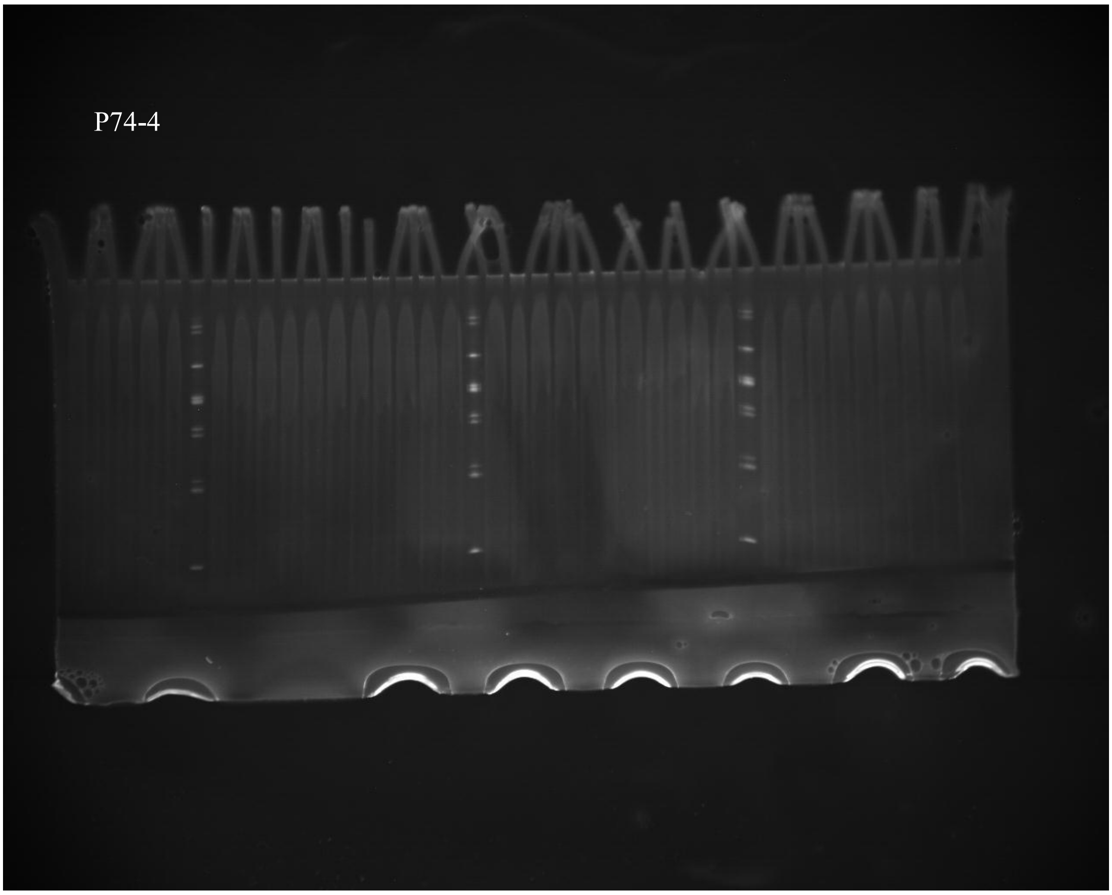
P74-4

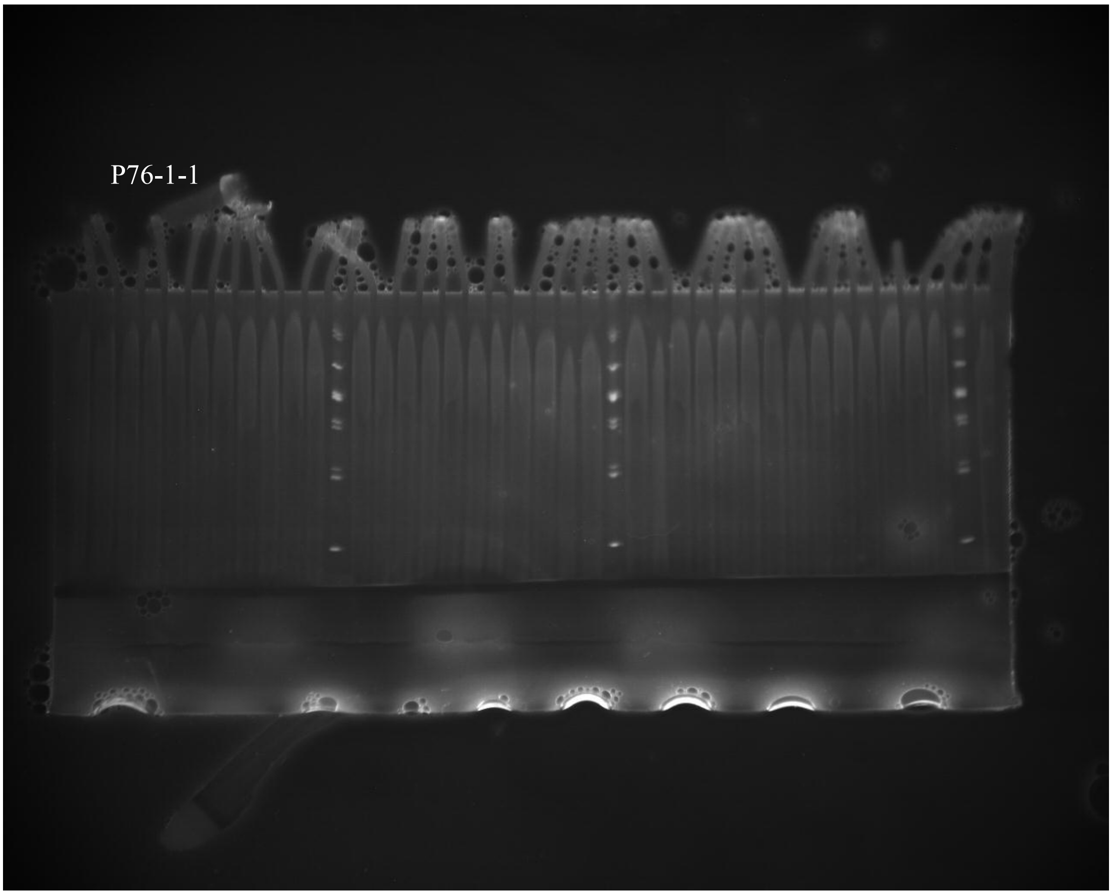
P76-1-1

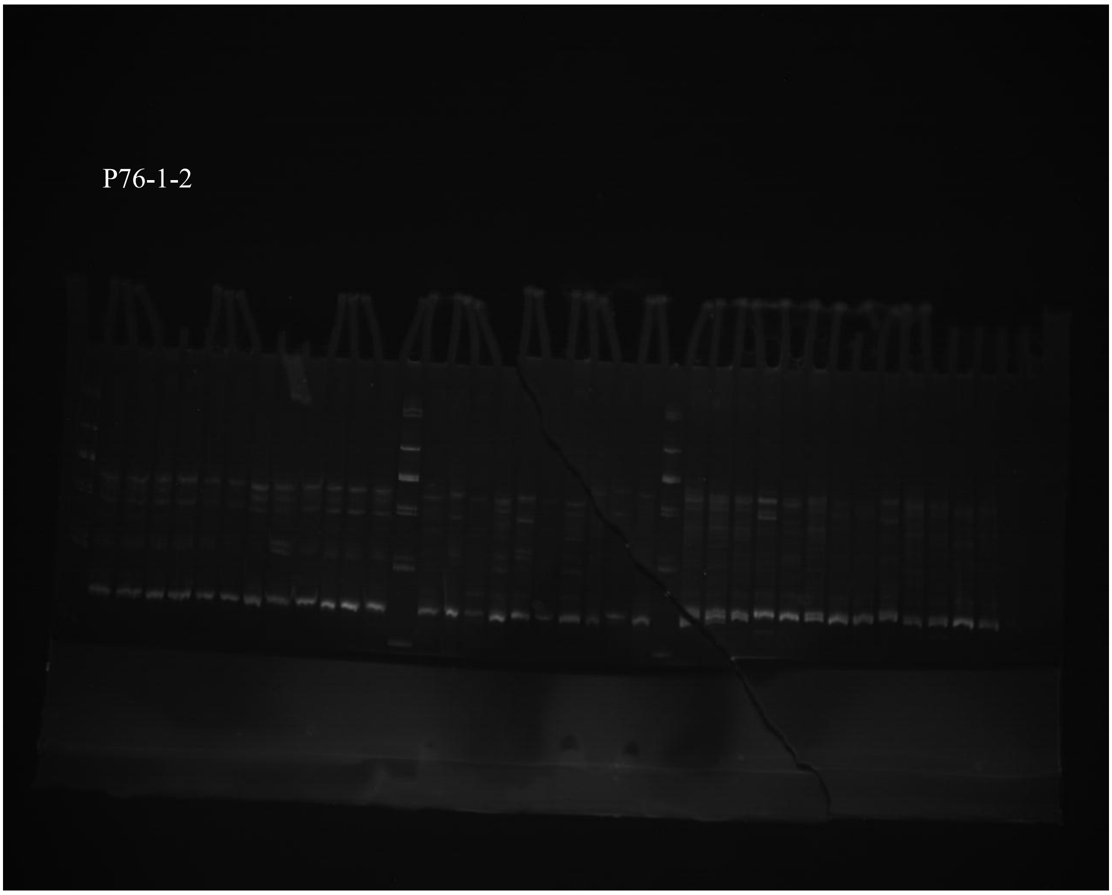
P76-1-2

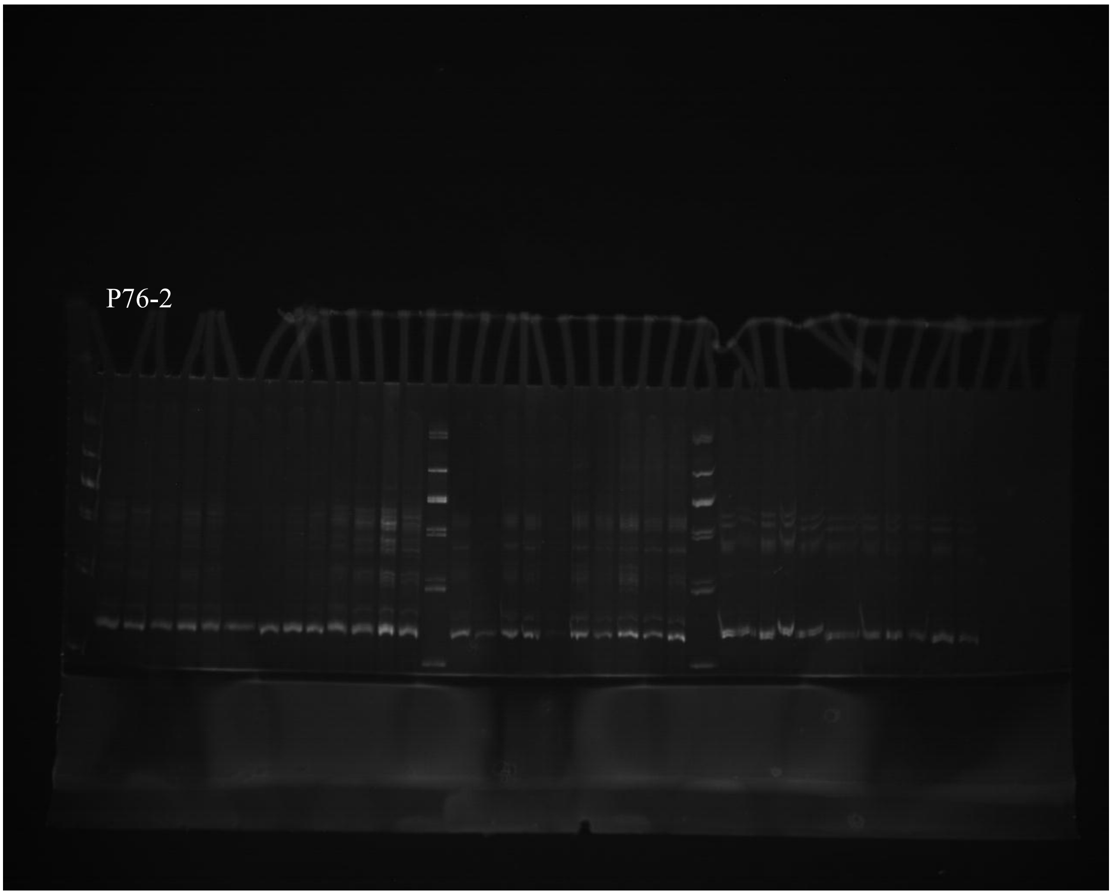
P76-2

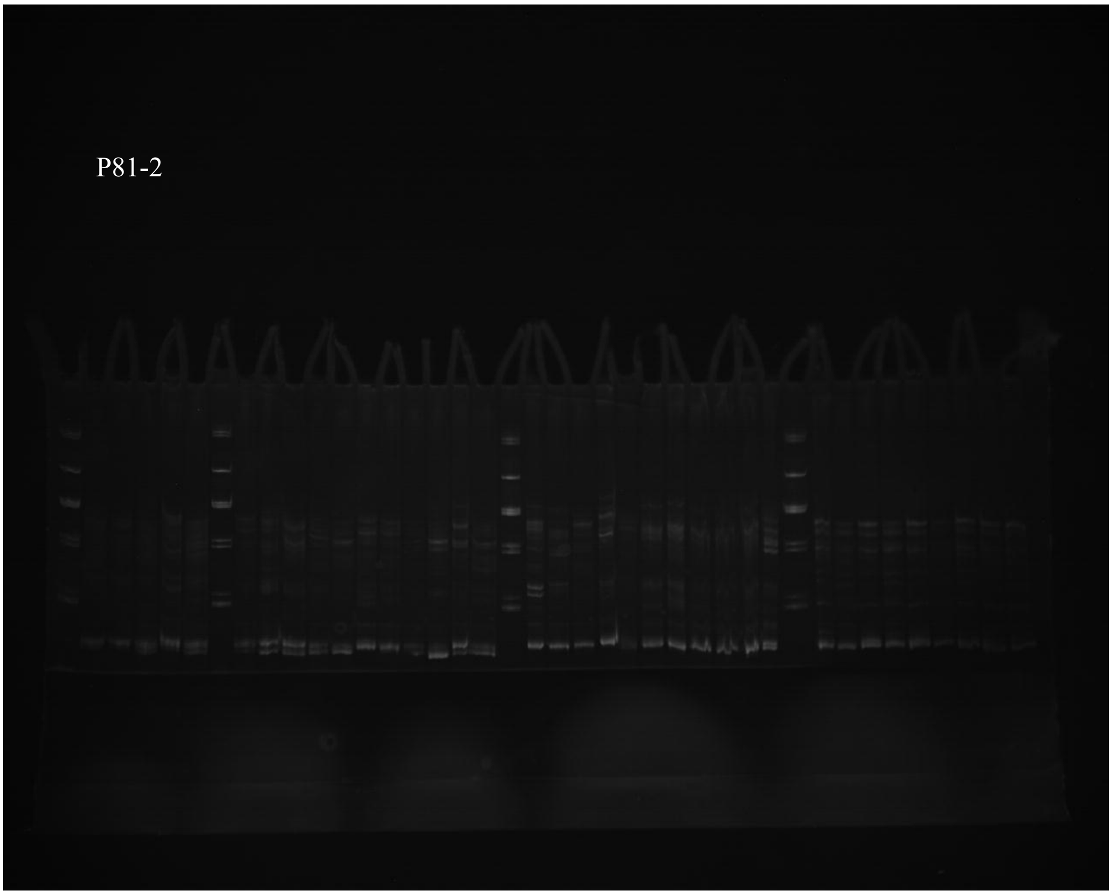
P81-2

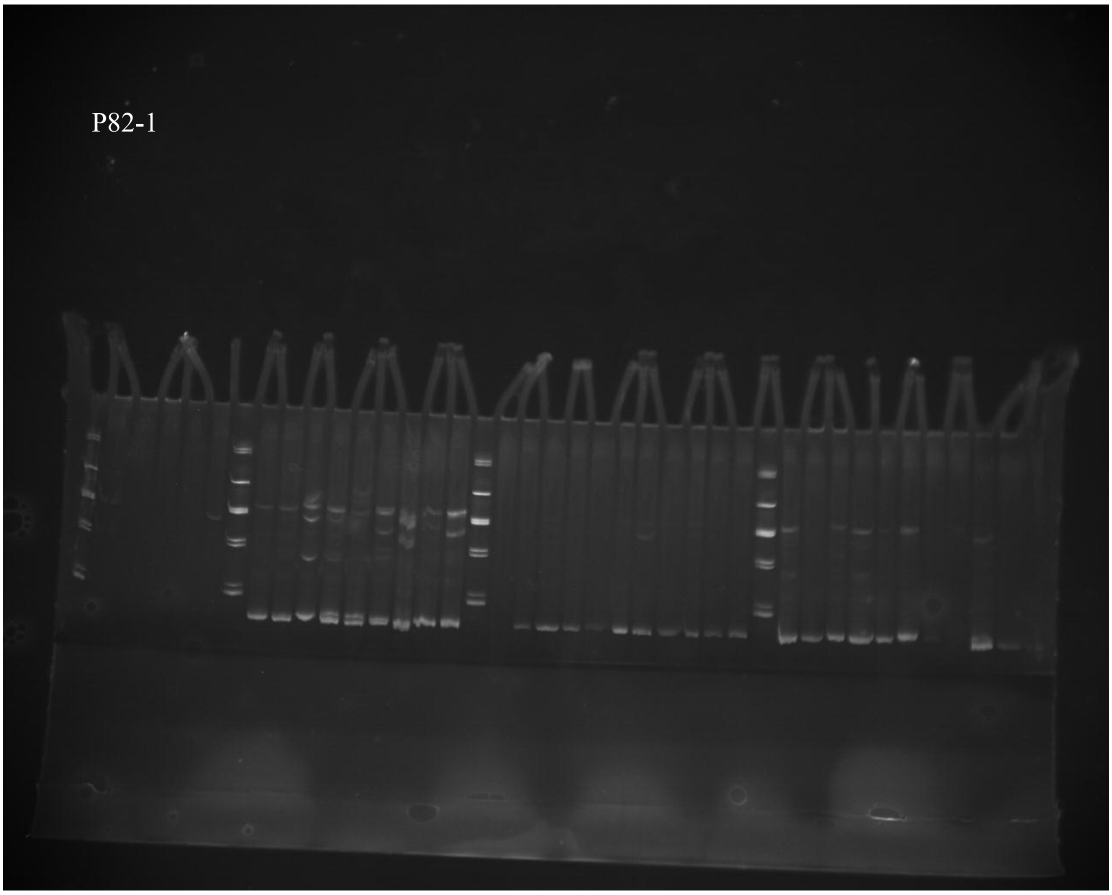
P82-1

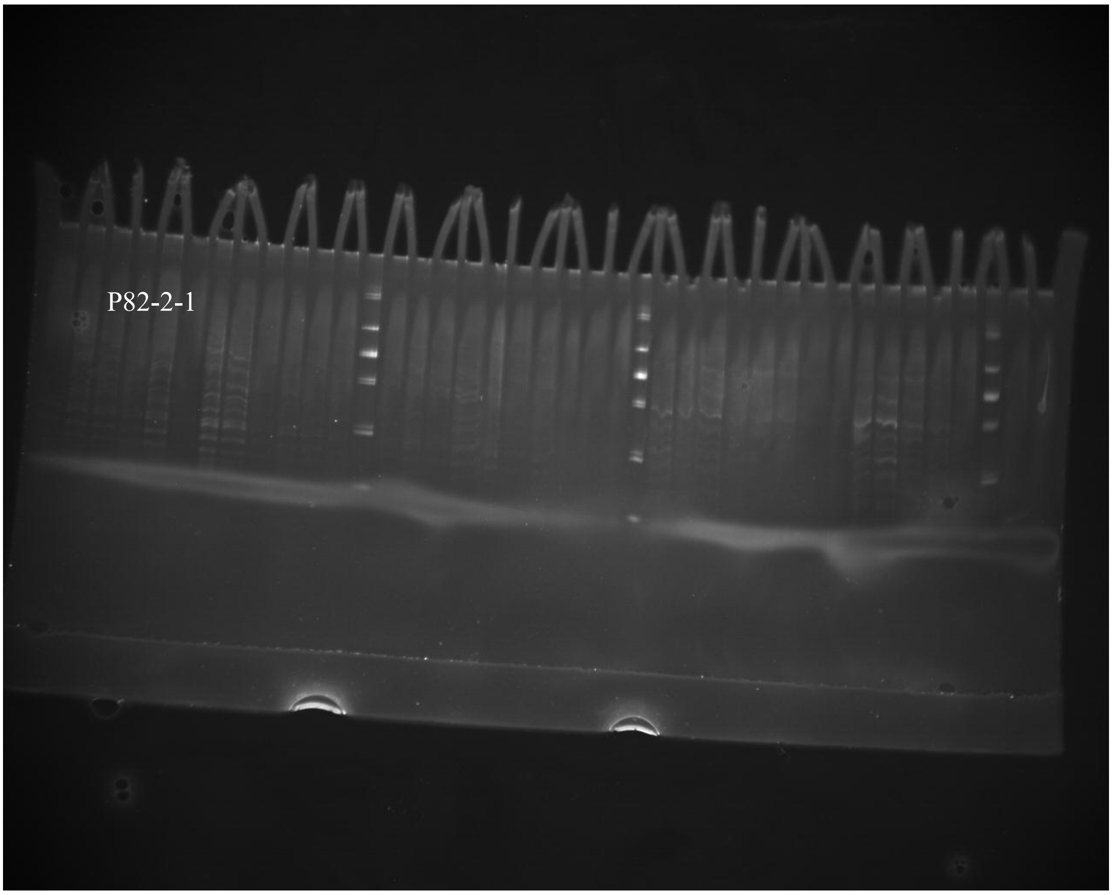
P82-2-1

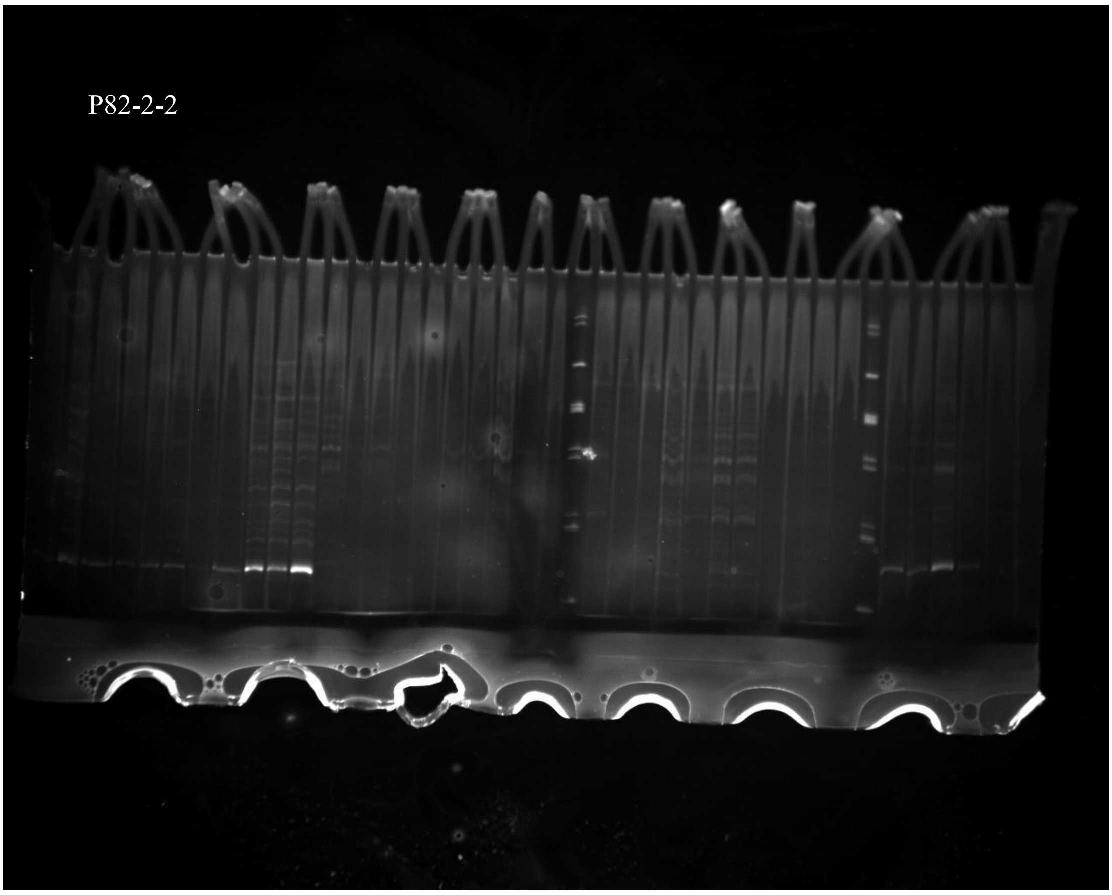
P82-2-2

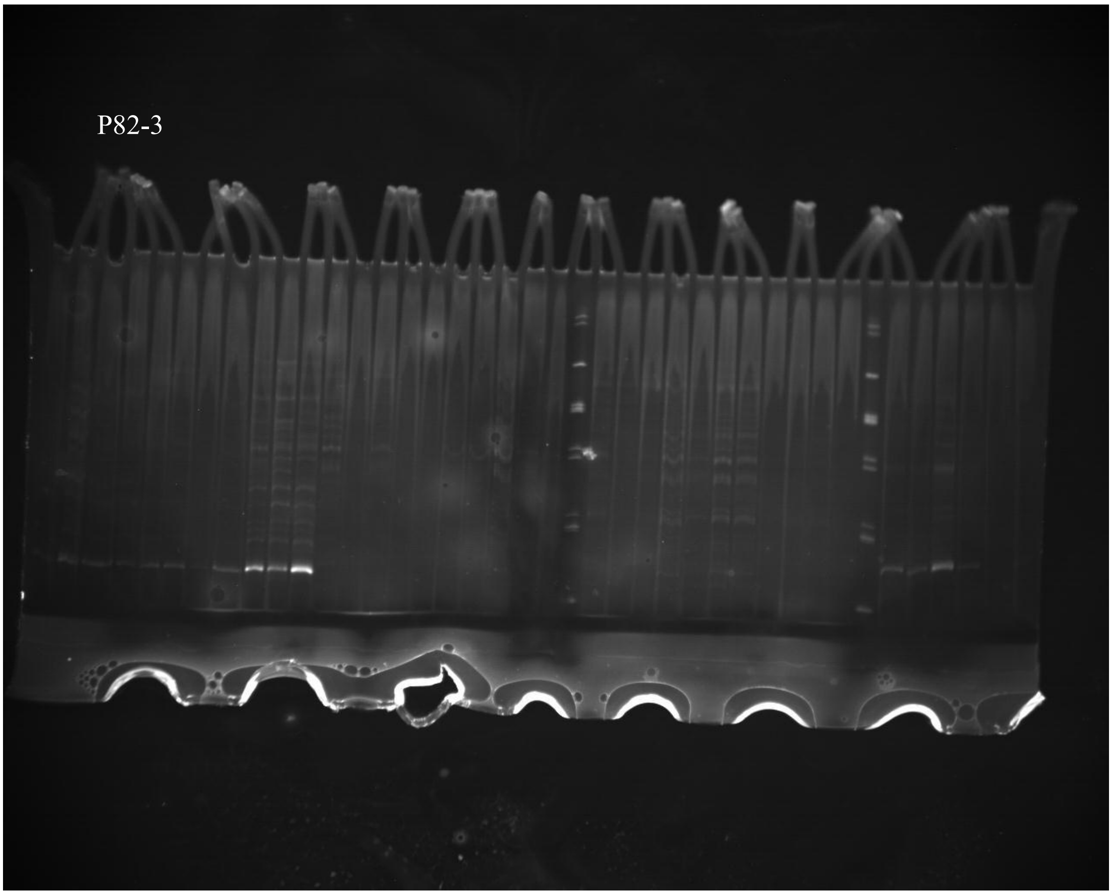
P82-3

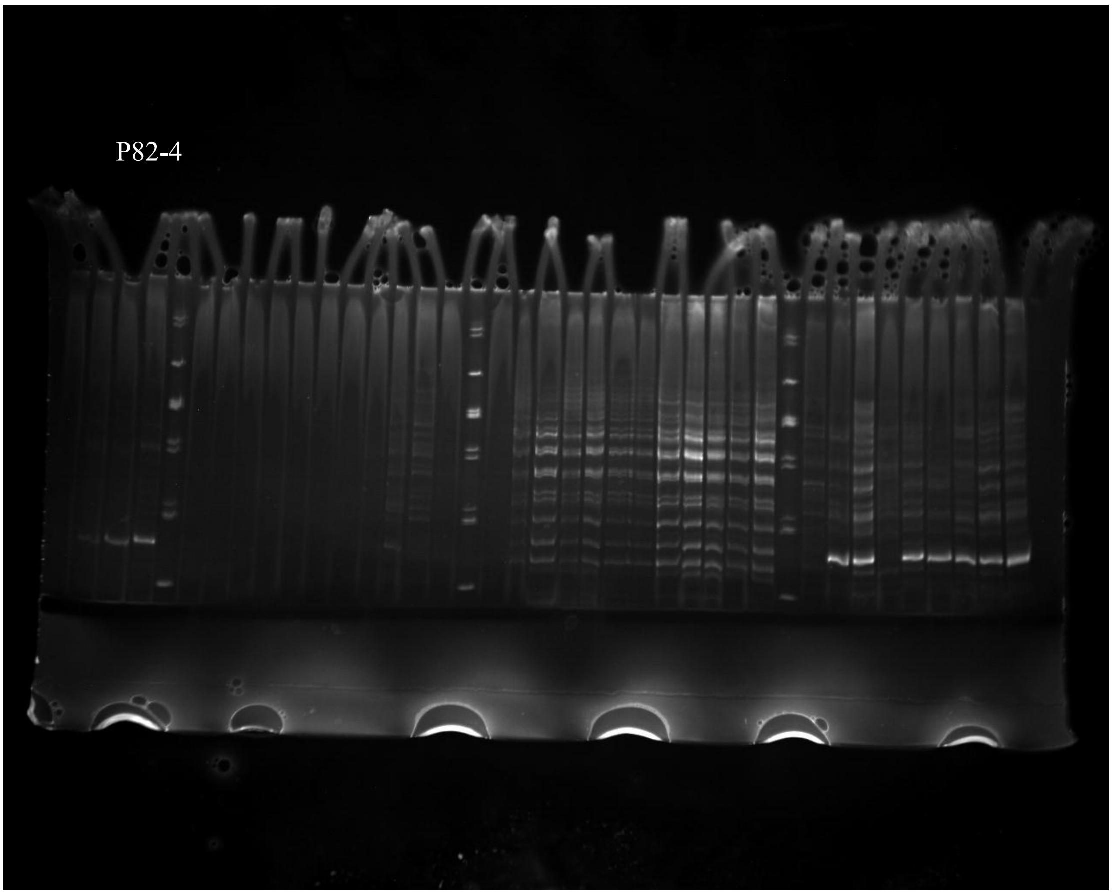
P82-4

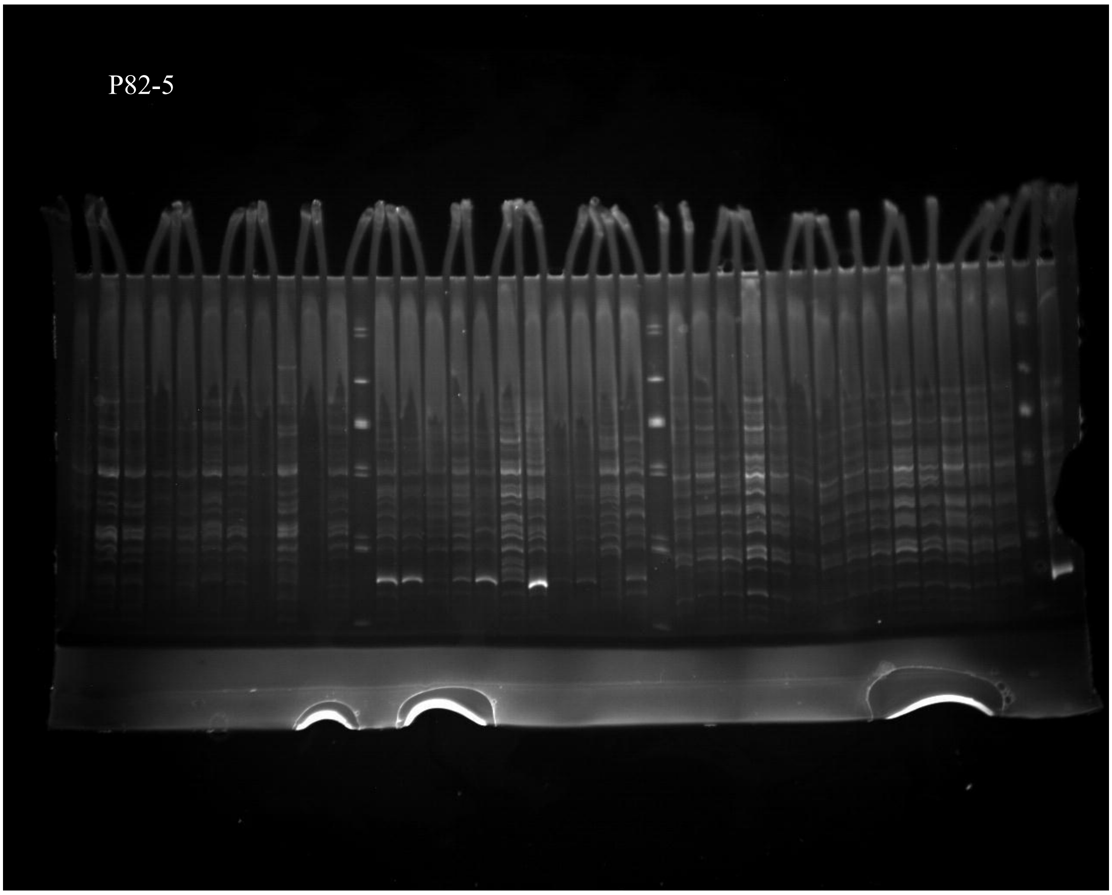
P82-5

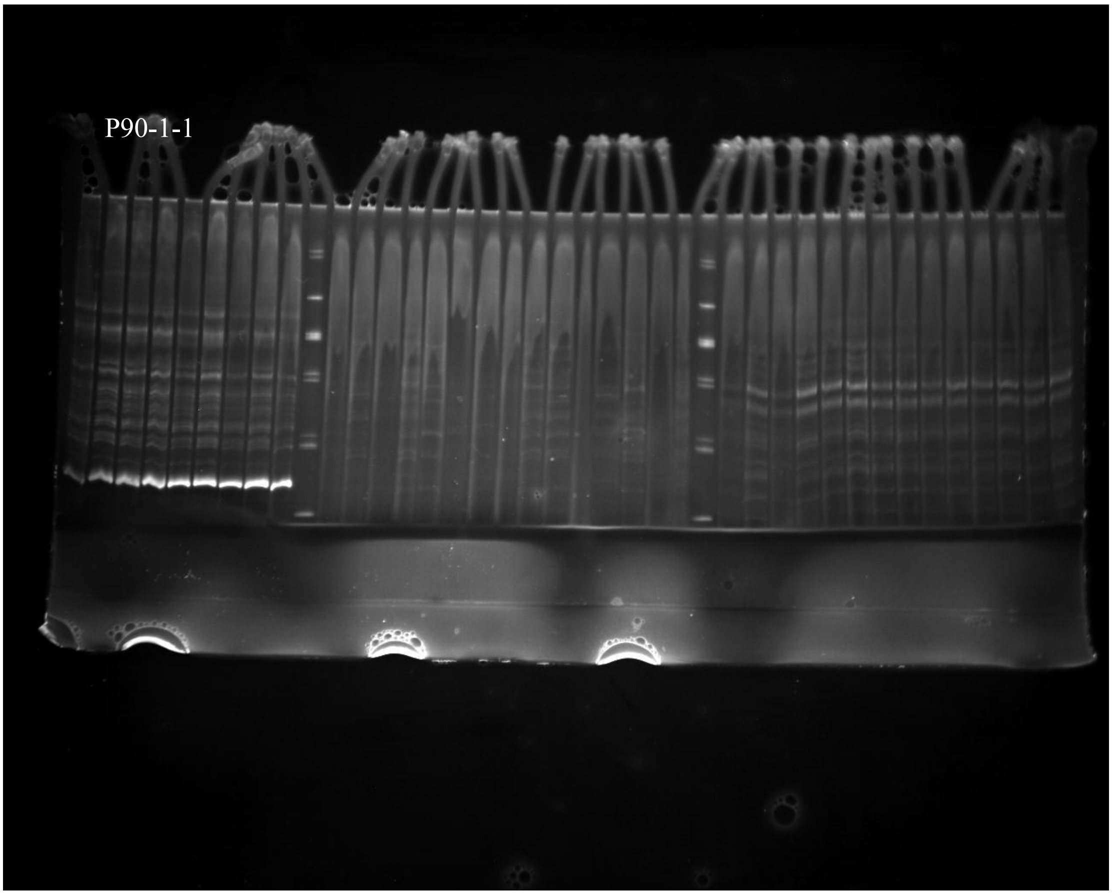
P90-1-1

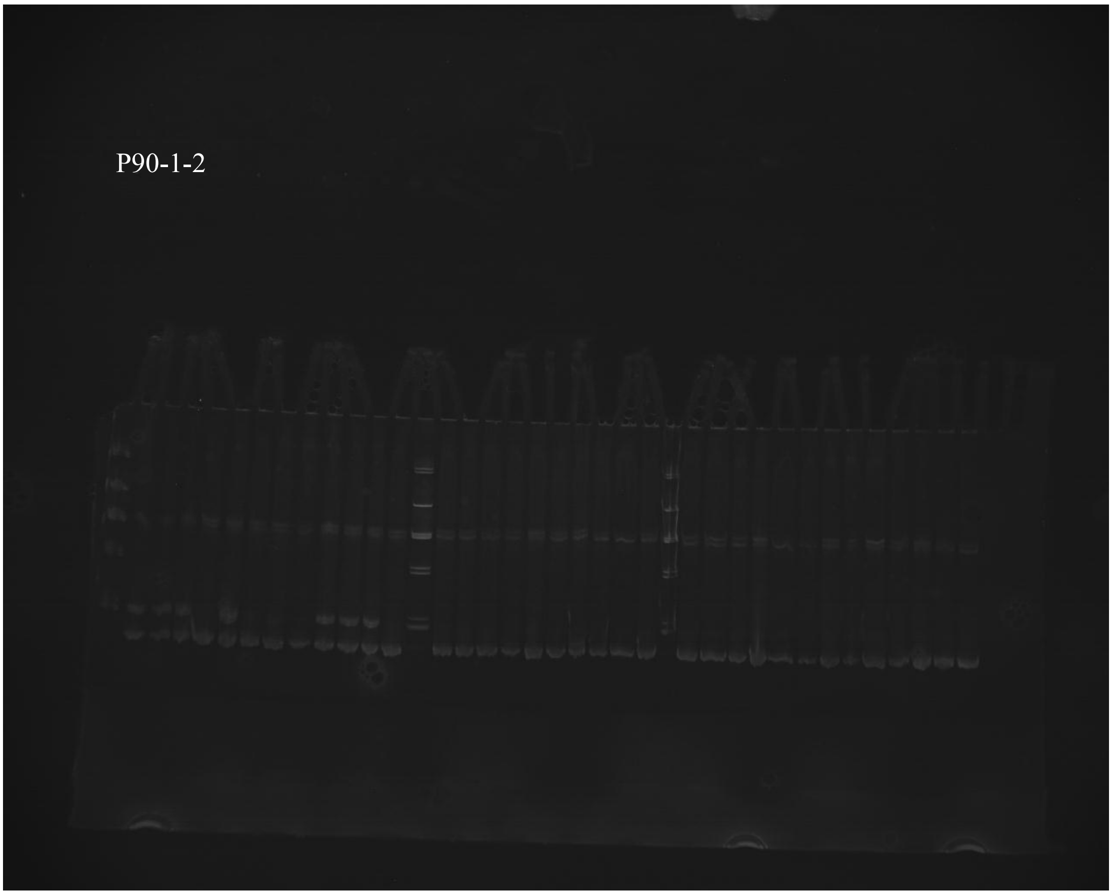
P90-1-2

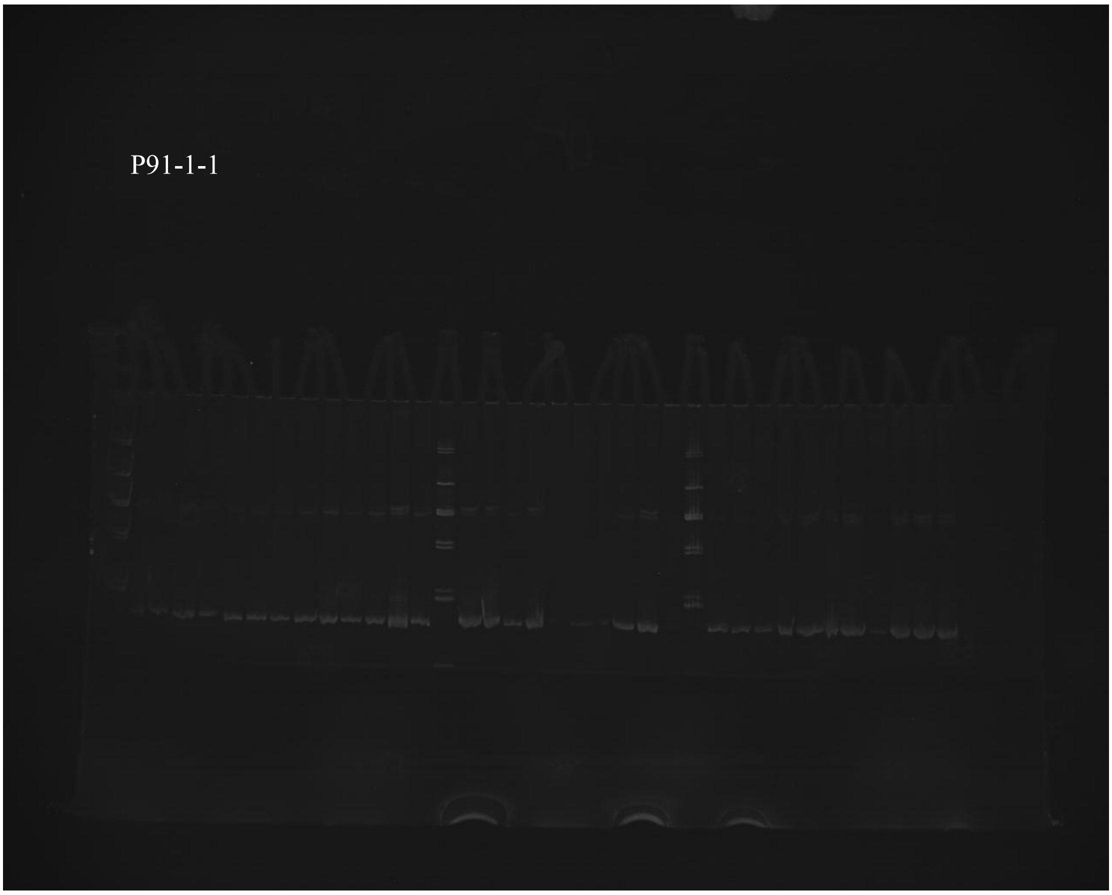
P91-1-1

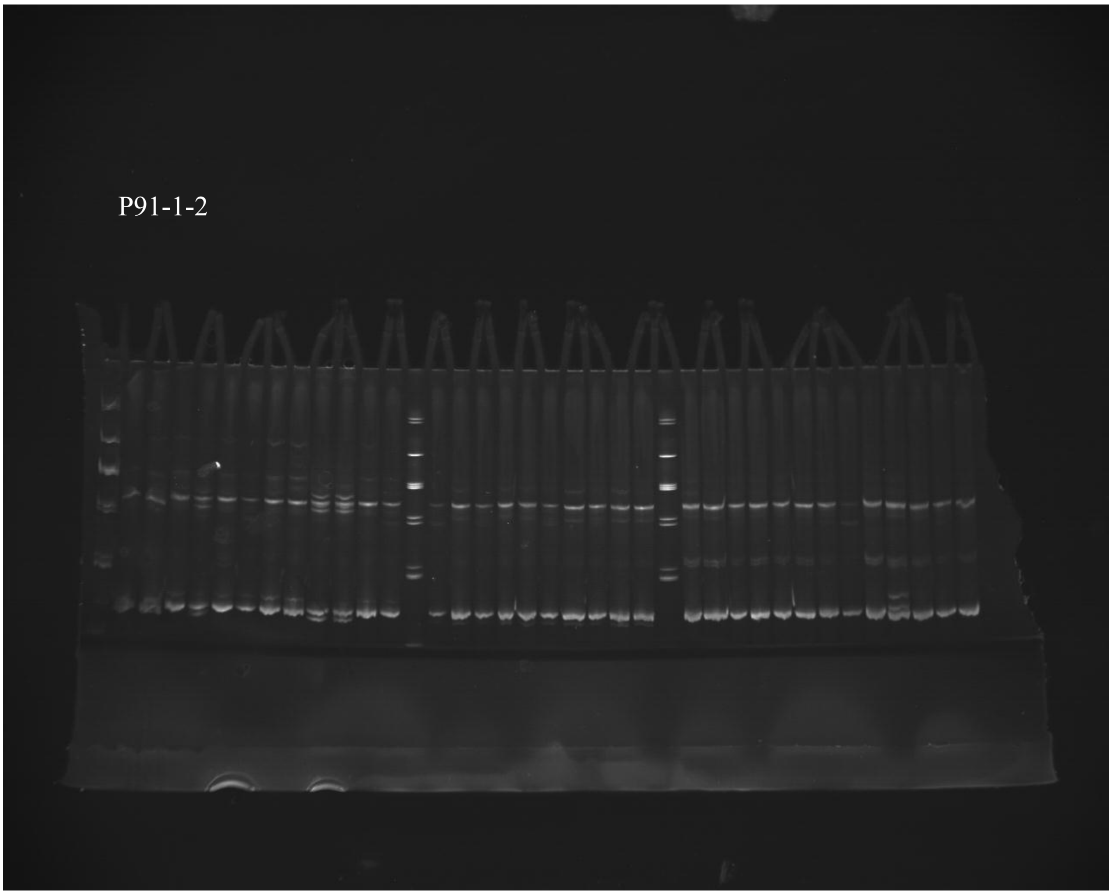
P91-1-2

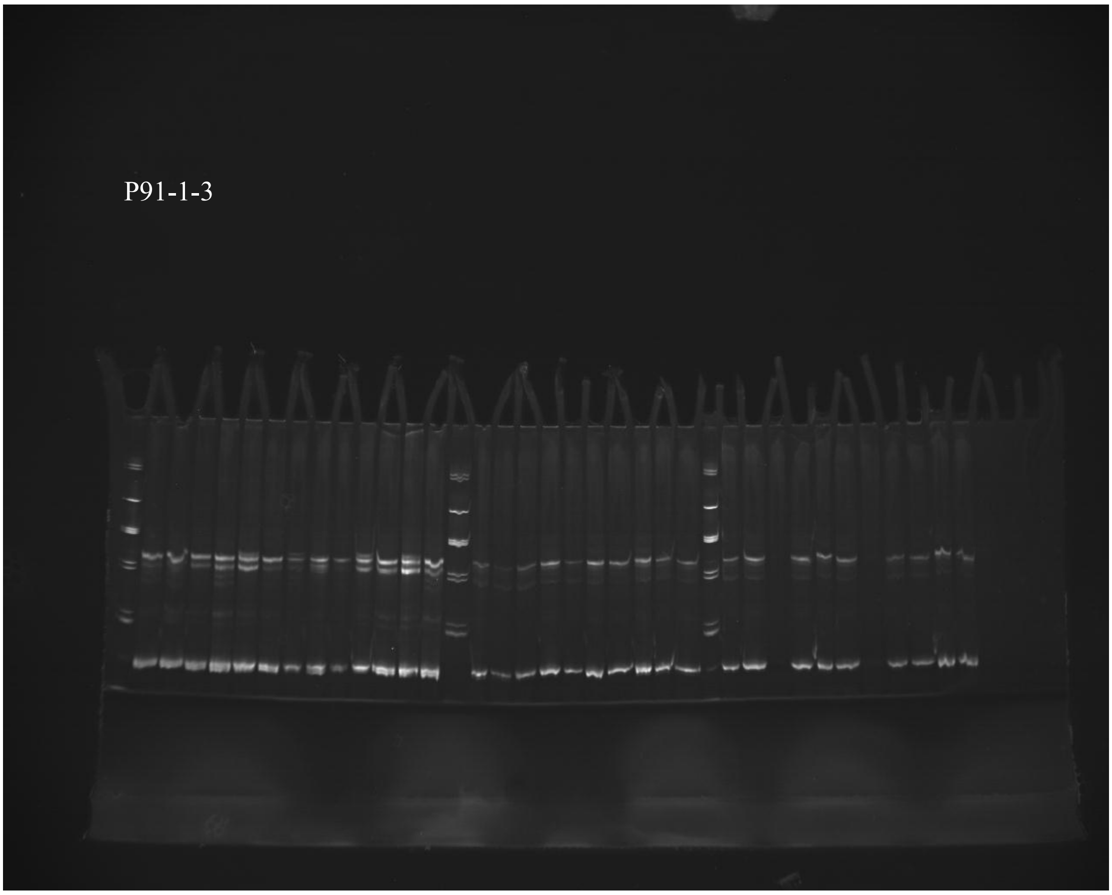
P91-1-3

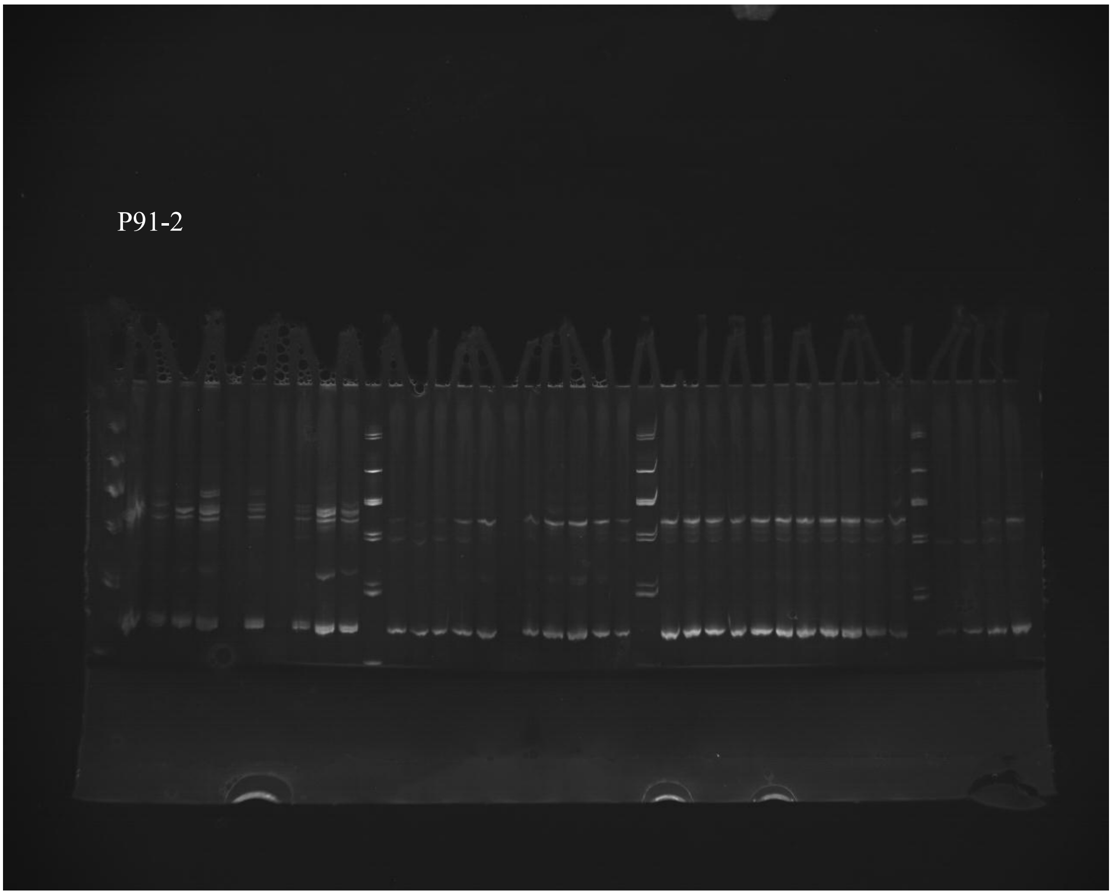
P91-2

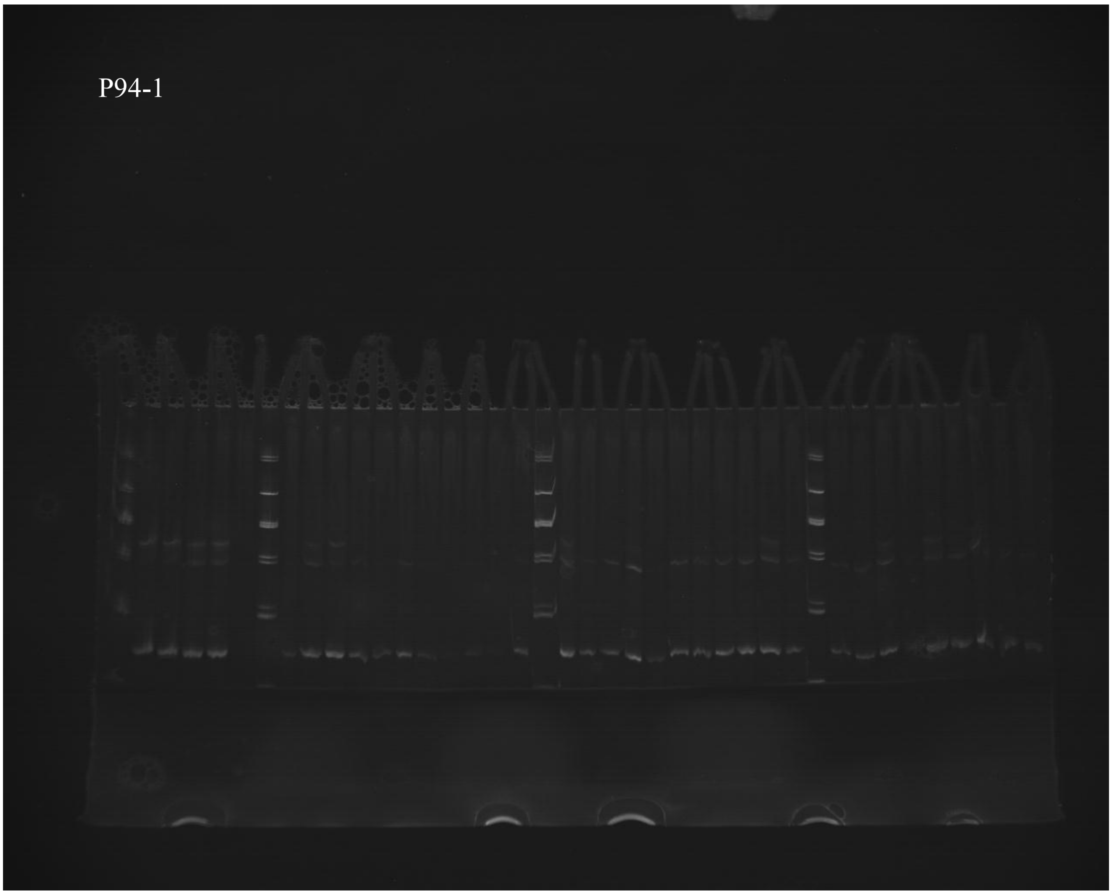
P94-1

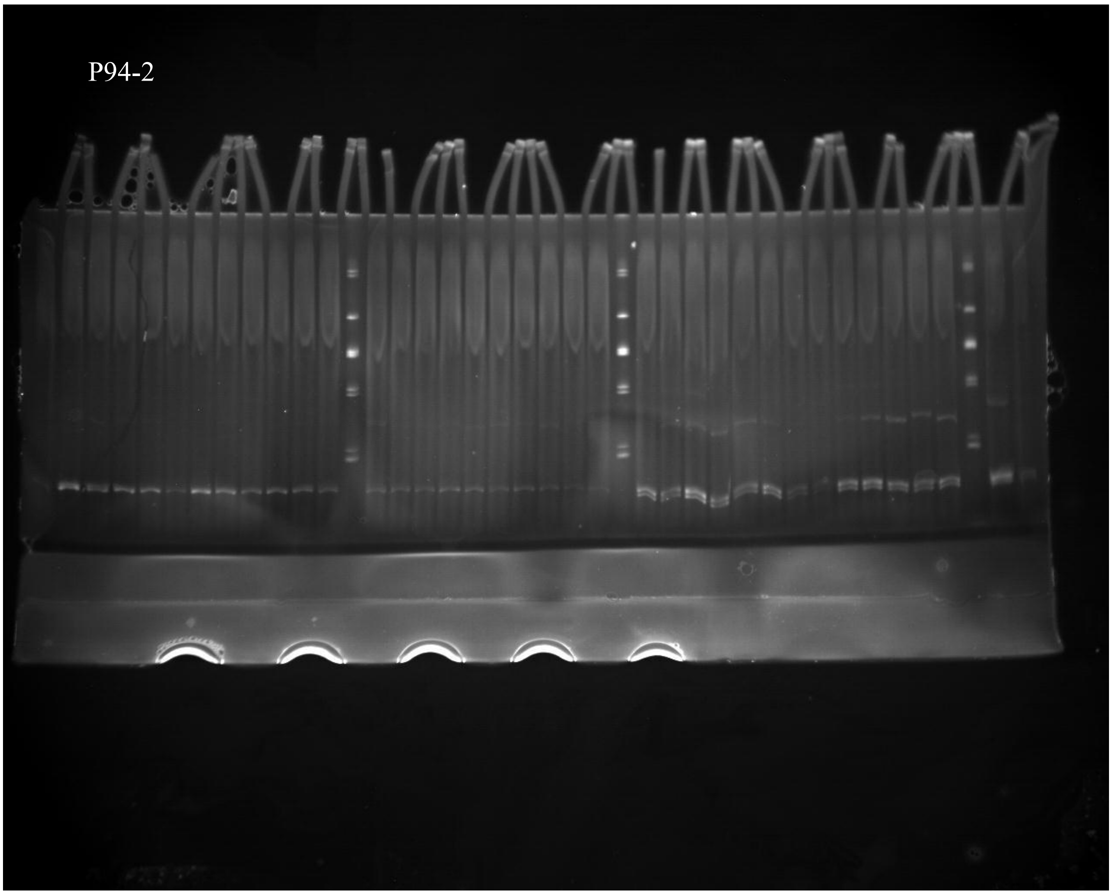
P94-2

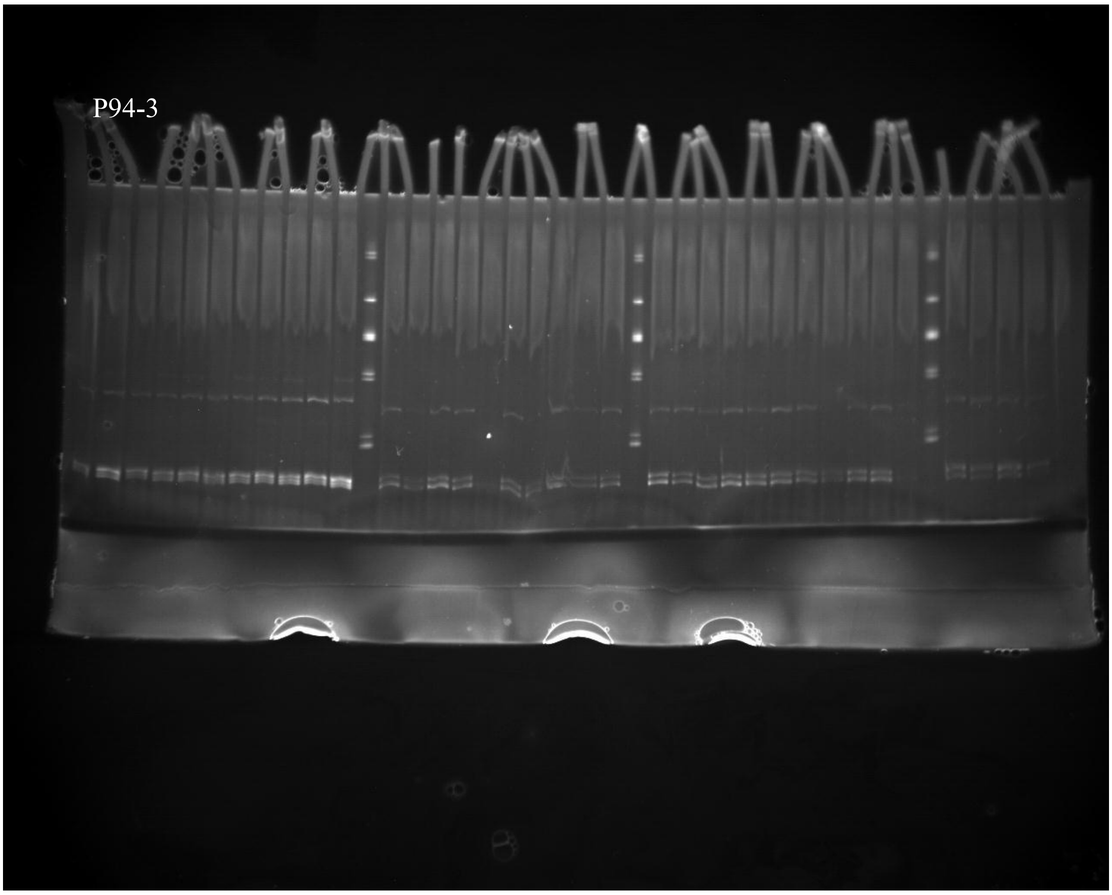
P94-3

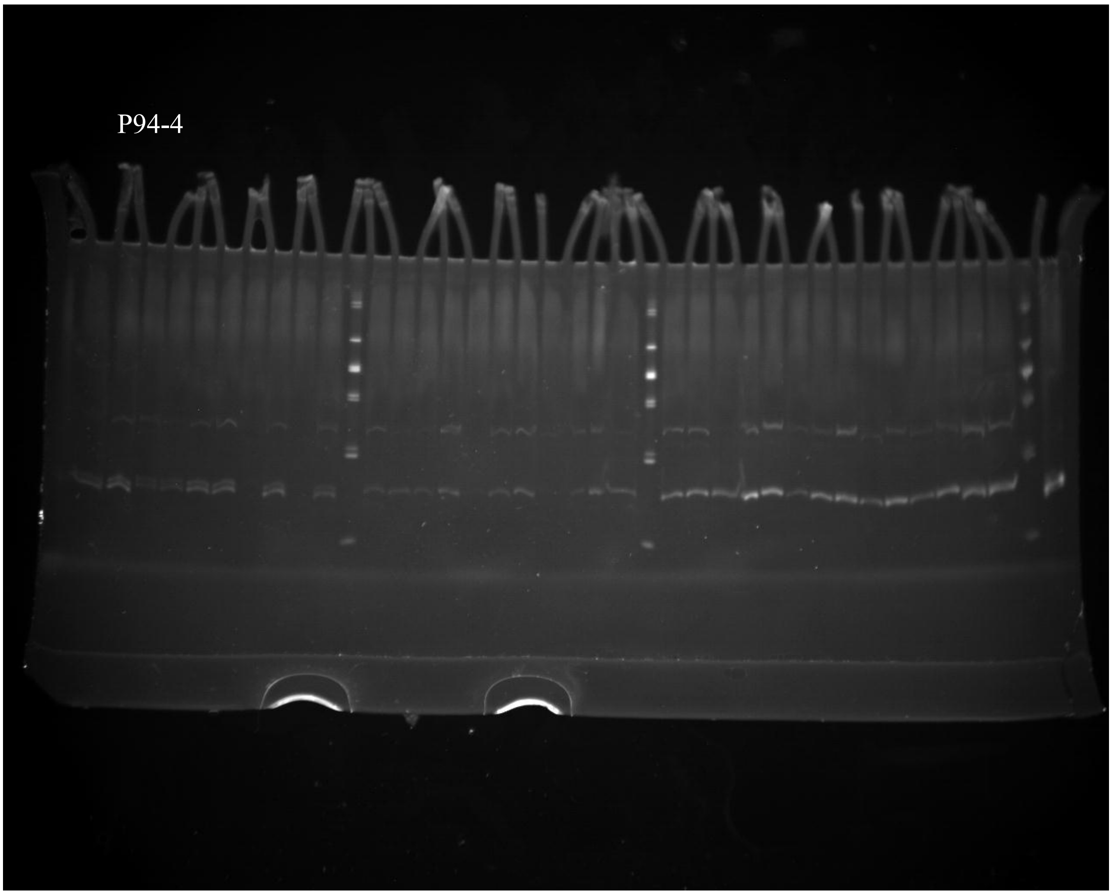
P94-4

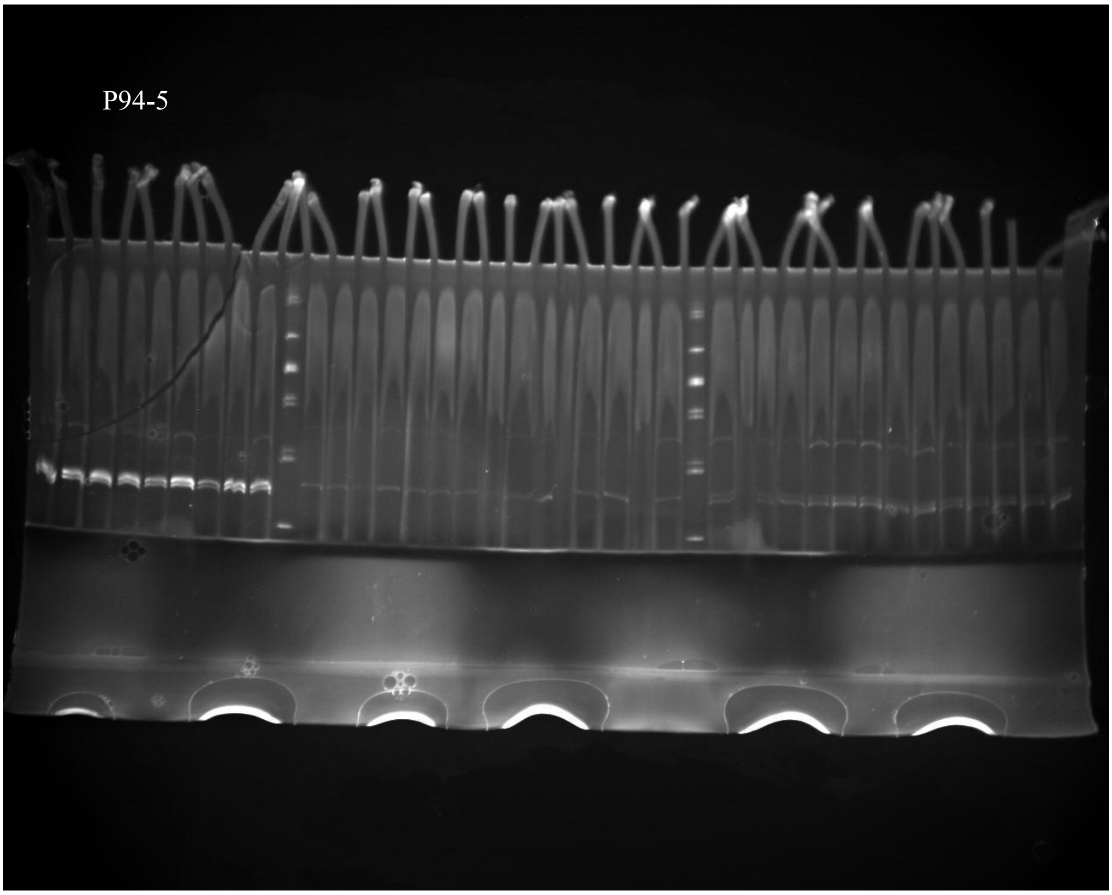

Figure S1. Amplification results of other primers
