# Supplementary material for: Assessment of Population Genetic Diversity of Medicinal Meconopsis integrifolia (Maxim.) Franch. Using Newly Developed SSR Markers
Source: Plants (Basel). 2024 Sep 12;13(18):2561. doi: 10.3390/plants13182561 (PMC11435270; doi:10.3390/plants13182561)
Supplement: Supplementary file 1 [file plants-13-02561-s001.zip › Figure S2.pdf]

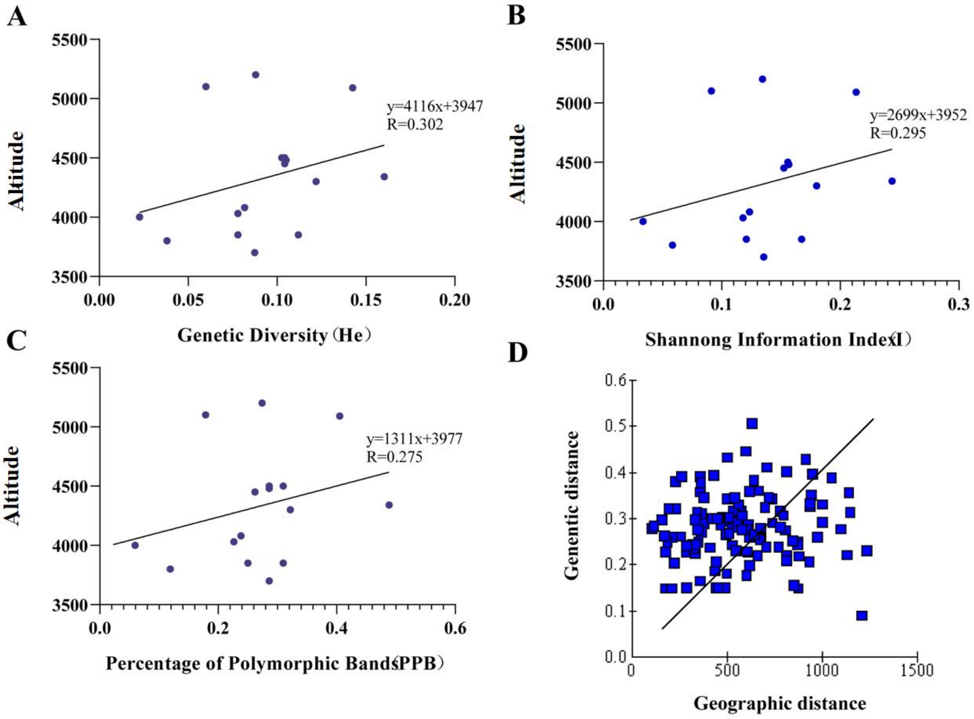

Figure S2: Correlation analysis between different indicators sing SSR analysis.(A) Correlation between Altitude and genetic diversity (He); (B) Correlation between Altitude and Shannon information index (I); (C) Correlation between Altitude and Percentage of Polymorphic Bands (PPB); (D) Correlation between Nei's genetic distance and geographic distance.
